# Methylation data imputation performances under different representations and missingness patterns

**DOI:** 10.1186/s12859-020-03592-5

**Published:** 2020-06-29

**Authors:** Pietro Di Lena, Claudia Sala, Andrea Prodi, Christine Nardini

**Affiliations:** 1 Department of Computer Science and Engineering, University of Bologna, Mura Anteo Zamboni 7, Bologna, Italy; 2 Department of Physics and Astronomy, University of Bologna, Viale Berti Pichat 6/2, Bologna, Italy; 3 Smart Cities Living Lab, ISOF CNR, Via P. Gobetti, 101, Bologna, Italy; 4 Istituto per le Applicazioni del Calcolo Mauro Picone, CNR, Via dei Taurini, 19, Roma, Italy

**Keywords:** Imputation, DNA methylation, M-value, *β*-value, Missing data mechanisms, MCAR, MAR, MNAR

## Abstract

**Background:**

High-throughput technologies enable the cost-effective collection and analysis of DNA methylation data throughout the human genome. This naturally entails missing values management that can complicate the analysis of the data. Several general and specific imputation methods are suitable for DNA methylation data. However, there are no detailed studies of their performances under different missing data mechanisms –(completely) at random or not- and different representations of DNA methylation levels (*β* and *M*-value).

**Results:**

We make an extensive analysis of the imputation performances of seven imputation methods on simulated *missing completely at random* (MCAR), *missing at random* (MAR) and *missing not at random* (MNAR) methylation data. We further consider imputation performances on the popular *β*- and *M*-value representations of methylation levels. Overall, *β*-values enable better imputation performances than *M*-values. Imputation accuracy is lower for mid-range *β*-values, while it is generally more accurate for values at the extremes of the *β*-value range. The MAR values distribution is on the average more dense in the mid-range in comparison to the expected *β*-value distribution. As a consequence, MAR values are on average harder to impute.

**Conclusions:**

The results of the analysis provide guidelines for the most suitable imputation approaches for DNA methylation data under different representations of DNA methylation levels and different missing data mechanisms.

## Background

Epigenomics is currently a very active research area aiming to shed light on the modifications in gene expression that are both independent from DNA mutations and still inheritable (mitotically and meiotically) [1, 2].

We focus here on DNA methylation, involving the covalent addition of a methyl group to the 5’-carbon cytosine in dinucleotide cytosine phosphate guanine (CpG dinucleotide). The relevance of DNA methylation spans several domains in biology, from embryonic development [3, 4] to physiological ageing, cancer [5–7] and shaping of the immune system [8], including vaccination [9]. For these reasons, a deep understanding of the fittest metrics and statistics to model methylations’ activity is important to offer a reliable assessment of its role as a potential biomarker.

Further, methylation data are stable and reproducible and offer a large amount of publicly available data, thanks to the cost-effectiveness of methylation arrays (Illumina Human Infinium Beadchips 27k, 450k and now 850k). This publicly available abundance of data, in turn, enables meta-analyses to advance discovery, thanks to numerous (ad hoc) preprocessing approaches [10].

Infinium assays utilize a pair of probes to measure the intensities of the methylated and unmethylated alleles at each CpG site. The methylation value is then computed based on the measured intensities of this pair of probes, across all cells of the sampled tissue. Two metrics are defined to indicate methylation levels: the *β*-value (ranging from 0 to 1), and the *M*-value (ranging from −*∞* to *∞*). The relationship between the two representations is a logit trasformation [11]. *β*-values at the extreme of their range (i.e. close to 0 and 1) have been shown to suffer from severe heteroscedasticity [11], differently from *M*-values. However, despite the desirable homoskedasticity of the *M*-value, in particular for differential analyses [12], *β*-value remains the predominantly used metric owing to its intuitive biological interpretation, and it is recommended by array producers [11]. As a result, both metrics are used across the literature, mostly depending on the background of the investigators.

Experimental methylation data often contain multiple missing values, that can affect downstream analyses. Examples include epigenetic clocks that estimate biological age from small sets of pre-selected age-correlated CpG sites [13–16], recently proven to be highly sensitive to small perturbations of methylation levels [17], as well as more general purpose differential analyses. Hence, accurate imputation of missing data is required for improving the quality of DNA methylation downstream analysis.

Missing data can be organized into three classes [18]: i) *missing completely at random* (MCAR) values, if the probability of being missing is totally independent of both the observed and unobserved variables; ii) *missing at random* (MAR) values, if the probability of a value of being missing does not depend on the value itself but may depend on the observed variables; iii) *missing not at random* (MNAR), if the probability of being missing depends on the missing value itself. When dealing with missing values, MNAR mechanisms are usually considered *not ignorable* since the imputation process needs to model explicitly the missing data mechanism in order to avoid biased estimations [18]. On the contrary, MCAR and MAR mechanisms are considered *ignorable* and are often used as underlying assumption of most imputation methods. However, there is no assessed statistical way to detect the specific missingness mechanism in the data [19], and assumptions need to be made based on the knowledge of the specific data and its sources of acquisition. Unfortunately, to the best of our knowledge, there is no study that addresses the missingness patterns in DNA methylation data generated with Illumina Beadchips. As such, our work will comprise the study of all three patterns.

We recently introduced a novel regression-based imputation method, *methyLImp*, specifically designed for methylation data, and compared its performances, under the MCAR assumption, with six other general purpose imputation methods [17], namely: i) two mean-value imputation approaches, the basic *mean* imputation approach and *impute.knn* [20]; ii) three iterative soft-thresholding approaches, *softImpute* [21], *imputePCA* [22] and *SVDmiss* [23]; iii) one regression-based approach, *missForest* [24]. Here we further extend the assessment and comparison of imputation performances of the seven benchmarked methods (see “Benchmark imputation software” section) on the same benchmark set of DNA methylation data used in [17], which includes 58 datasets with healthy and disease samples on a variety of different tissues and ages (see “Benchmark data” section). In particular, we extend the performance comparison in two directions: i) by considering *β*- and *M*-values data representations of the DNA methylation levels; ii) by explicitly considering and simulating the three different types of missing data mechanisms, a characterization still missing in literature for DNA methylation data (see “Missing values simulation procedure” section for details). In particular, we model MCAR values as missing values that are a direct consequence of random errors in experimental measurements, MAR values as consequence of CpG-specific probes that are more likely to fail to capture the target sequences, and MNAR values as missingness patterns that depend on the specific methylation level. In this latter case, we consider separately three different ranges of methylation levels: *low-range MNAR* missing values, for which we assign a higher probability of being missing to values in the [0,0.2]*β*-value range, *mid-range MNAR* for which we assign a higher probability of being missing to values in the [0.4,0.6] range, and *high-range MNAR* that cover the [0.8,1]*β*-value range.

The results of this extensive analysis highlight issues and limitations of DNA methylation data imputation, and provide suggestions for the most appropriate imputation approaches. In particular, as already noticed in [17], our tests show that mid-range *β*-values are harder to impute than *β*-values at the extremes (i.e. close to 0 and 1). Sampled MAR values appear to be more compressed in the mid-range and less in the lower-range than the expected *β*-value distribution. The negative consequence of such a scenario is that methylation levels of CpGs that frequently present missing values are harder to impute accurately. Furthermore, in principle one could expect the (more homoscedastic) *M*-values to be more suitable for imputation than the (more heteroscedastic) *β*-values, at least for regression-based imputation methods. However, contrary to this expectation, *β*-values appear to be the most suitable representation for methylation level imputations. Remarkably, these results hold true irrespective of the specific imputation method, although regression-based method have, on the average, better performances.

## Results

We compare the imputation accuracy of seven benchmarked methods on simulated missing values with respect to the *M*-value and *β*-value measures and with respect to MCAR, MAR and MNAR simulated missing values (see “Missing values simulation procedure” section for details and rationale about the sampling procedure). For performance comparison, the same simulated missing values have been imputed independently by first using the *M*-value and then the *β*-value representation of the data. In order to directly compare *M*- and *β*-value imputation performances, all imputed *M*-values have been converted into *β*-values before evaluation. The amount of simulated missing values introduced in each test is equal to 3% of the size of the dataset, which corresponds to the average number of real missing values observed in our benchmark set (see Table 1). Imputation performance are measured using two metrics (see “Evaluation metrics” section): *mean absolute error* (MAE) and *root mean square error* (RMSE). Since *β*-values are limited in the [0,1] range, both metrics are also limited in [0,1], where a value close to 0 means (almost) perfect imputation.

**Table 1:** Benchmark datasets

ID	GEO ID	Tissue	Disease status	# Samples	# Missing values (21k)	% Missing values (21k)
D1	GSE32146	Colon mucosa	Crohn’s disease	10	175	0.08%
D2	GSE32146	Colon mucosa	Ulcerative colitis	5	161	0.15%
D3	GSE32146	Colon	Normal	10	171	0.08%
D4	GSE32148	Blood	Normal	19	325	0.08%
D5	GSE40005	Blood	Normal	12	324	0.13%
D6	GSE42921	Colon mucosa	Crohn’s disease	5	192	0.18%
D7	GSE42921	Colon mucosa	Ulcerative colitis	6	331	0.26%
D8	GSE42921	Colon	Normal	12	874	0.34%
D9	GSE43091	Liver	Cancer	50	1,980	0.19%
D10	GSE43091	Liver	Normal	4	125	0.15%
D11	GSE44684	Cerebellum	Normal	6	67	0.05%
D12	GSE49393	Prefrontal Cortex	Normal	25	54,000	10.11%
D13	GSE51388	Blood	Normal	60	292,200	22.79%
D14	GSE52113	Blood	Normal	24	0	0.00%
D15	GSE53051	Breast	Cancer	14	0	0.00%
D16	GSE53051	Colon	Cancer	35	0	0.00%
D17	GSE53051	Colon, Pancreas	Normal	9	0	0.00%
D18	GSE53051	Lung	Cancer	9	0	0.00%
D19	GSE53051	Pancreas	Cancer	29	0	0.00%
D20	GSE53051	Thyroid	Cancer	70	0	0.00%
D21	GSE53162	Brain, Cerebellum, Prefrontal Cortex	Normal	21	0	0.00%
D22	GSE53740	Blood	Normal	165	0	0.00%
D23	GSE57360	Brain	Normal	5	0	0.00%
D24	GSE61151	Blood	Normal	184	7,544	0.19%
D25	GSE61257	Adipose	Non-alcoholic fatty liver disease (NAFLD)	8	88	0.05%
D26	GSE61257	Adipose	Non-alcoholic steatohepatitis (NASH)	9	142	0.07%
D27	GSE61257	Adipose	Normal	15	241	0.08%
D28	GSE61258	Liver	Non-alcoholic fatty liver disease (NAFLD)	14	370	0.12%
D29	GSE61258	Liver	Non-alcoholic steatohepatitis (NASH)	7	218	0.15%
D30	GSE61258	Liver	Normal	32	966	0.14%
D31	GSE61258	Liver	Primary biliary cholangitis (PBC)	12	251	0.10%
D32	GSE61258	Liver	Primary sclerosing cholangitis (PSC)	14	352	0.12%
D33	GSE61259	Muscle	Non-alcoholic fatty liver disease (NAFLD)	9	90	0.05%
D34	GSE61259	Muscle	Non-alcoholic steatohepatitis (NASH)	7	49	0.03%
D35	GSE61259	Muscle	Normal	10	96	0.04%
D36	GSE61380	Brain	Normal	15	2,4671	7.70%
D37	GSE62003	Blood	Normal	35	0	0.00%
D38	GSE64495	Blood	Normal	106	32	0.00%
D39	GSE67477	Liver	Cancer	6	461	0.36%
D40	GSE67484	Liver, Intestine-Small	Normal	4	45	0.05%
D41	GSE69502	Brain, Spinal Cord	Normal	20	37,781	8.84%
D42	GSE71955	Blood	Normal	62	260,245	19.64%
D43	GSE73103	Blood	Normal	268	1,005,268	17.55%
D44	GSE73747	Brain	Normal	9	7,069	3.68%
D45	GSE79122	Brain	Normal	7	99	0.07%
D46	GSE80970	Prefrontal Cortex	Normal	68	1,324	0.09%
D47	GSE82218	Blood	Normal	25	398	0.07%
D48	GSE84003	Blood	Normal	6	275	0.21%
D49	GSE88821	Colon, Rectum	Cancer	63	36,995	2.75%
D50	GSE88821	Colon, Rectum	Normal	8	4,680	2.74%
D51	GSE88821	Liver	Cancer	4	2,349	2.75%
D52	GSE89093	Blood	Normal	46	65,044	6.62%
D53	GSE89472	Blood	Normal	5	245	0.23%
D54	GSE89702	Cerebellum	Normal	17	49,572	13.65%
D55	GSE89703	Hippocampus	Normal	13	37,557	13.52%
D56	GSE89705	Putamen	Normal	17	49,215	13.55%
D57	GSE89706	Putamen	Normal	28	78,736	13.16%
D58	GSE97362	Blood	Normal	123	2,333	0.09%

The full range of tests is run, for computational efficiency reasons (see “Methods” section), for all datasets on the CpGs in the intersection between the 27k and 450k Human Beadchips (Type I probes). A reduced number of tests on the complete 450k benchmark data (in Additional file 3) show that there are no relevant differences between imputation accuracy on complete (450k) and restricted (21k) datasets.

We use the Wilcoxon signed-rank test to detect statistically significant difference between MAE and RMSE performances (see “Wilcoxon-testing procedure to assess statistically significantly better performances” section for more details). In particular, we use the Wicoxon test to assess whether a single method performance is statistically significantly better on the *β*-value or *M*-value representation (the best results are marked with ^∗^ in the report tables). We also use the Wilcoxon test to asses whether there are best performing methods for some missingness mechanism (the best methods, if any, are highlighted in bold in the report tables). In this latter case, we define best performing methods as those whose performances are never statistically significantly worse than any other method.

### Imputation of MCAR values

The average imputation performances on healthy and disease samples under the MCAR assumption are summarized in Table 2a and b, respectively. Due to the well-known methylation heterogeneity in disease samples (e.g. tumour) [25, 26], the imputation accuracy is consistently lower in disease than in control (healthy) samples, independently of the specific imputation method. This confirms the results already reported in [17] for the specific subset of (353) CpGs used in Horvath’s epigenetic clock [15].

**Table 2:** MCAR missing values

	MAE		RMSE	
Method	*M*-value	B-value	*M*-value	B-value
(a) Healthy datasets				
mean	0.030 ±0.001^∗^	0.030 ±0.001	0.051 ±0.001	0.050 ±0.001^∗^
impute.knn	0.039 ±0.007^∗^	0.059 ±0.012	0.079 ±0.015^∗^	0.112 ±0.019
softImpute	0.031 ±0.002	0.032 ±0.006^∗^	0.055 ±0.004	0.059 ±0.017^∗^
imputePCA	0.025 ±0.001^∗^	0.025 ±0.001	0.045 ±0.001	0.043 ±0.001^∗^
SVDmiss	0.035 ±0.001	0.027 ±0.001^∗^	0.063 ±0.002	0.048 ±0.002^∗^
missForest	0.026 ±0.001	0.026 ±0.001^∗^	0.044 ±0.003	0.043 ±0.002^∗^
methyLImp	0.029 ±0.001	0.025 ±0.001^∗^	0.050 ±0.002	0.047 ±0.002^∗^
(b) Disease datasets				
mean	0.048 ±0.001^∗^	0.048 ±0.001	0.080 ±0.002	0.079 ±0.002^∗^
impute.knn	0.059 ±0.008^∗^	0.082 ±0.013	0.107 ±0.014^∗^	0.142 ±0.018
softImpute	0.050 ±0.004	0.051 ±0.010^∗^	0.084 ±0.007	0.091 ±0.026^∗^
imputePCA	0.041 ±0.001^∗^	0.041 ±0.001	0.072 ±0.002	0.070 ±0.002^∗^
SVDmiss	0.055 ±0.001	0.045 ±0.001^∗^	0.093 ±0.002	0.080 ±0.003^∗^
missForest	0.042 ±0.001^∗^	0.042 ±0.001	0.071 ±0.002	0.070 ±0.002^∗^
methyLImp	0.043 ±0.001	0.037 ±0.001^∗^	0.074 ±0.002	0.066 ±0.002^∗^

Average Mean Average Error (MAE) and Root Mean Square Error (RMSE) imputation performance ± standard deviation. For each method, the ^∗^ symbol indicates the measure (either *M*-value or *β*-value) for which the Wilcoxon signed-rank test *p*-value is <0.05. Best results per metric with respect to the Wilcoxon signed-rank test are highlighted in bold

Furthermore, the regression-based imputation methods are the best performing among the benchmarked ones. In particular, according to the Wilcoxon signed-rank test, the imputation performances of *methyLImp* on *β*-values are never worse than those of the other methods on both *β* and *M*-values.

Note that for healthy samples, the average performances of *imputePCA* and *missForest* with respect to the RMSE metric are better than those of *methyLImp*, although the Wilcoxon test detects statistically significant differences in favour of the latter (Table 2).

This behaviour can be explained by observing the detailed results per dataset in Additional file 1. In short, 3% MCAR missing values in large datasets imply that there is at least one missing value for almost every CpG. Since *methyLImp* uses only complete observations for regression, this implies that in large datasets the regression is done on the average on just few tens of variables (see Table 2 in Additional file 2), which are usually not enough to build an appropriate regression model. This is the reason why *methyLImp*’s performance on the larger datasets (e.g. D22-GSE53740, D24-GSE61151, D38-GSE64495 in Additional file 1) are much worse than those of the baseline *mean* approach. However, since Wilcoxon test draws statistical inference from the rank sum instead of the mean, the average *methyLImp* performances are still detected as significantly better than those of the other methods. Anyway, we remark that in real methylation datasets such situation is quite unlikely since we typically have thousands of completely observed variables (see Table 1 in Additional file 2).

**Fig. 1:**
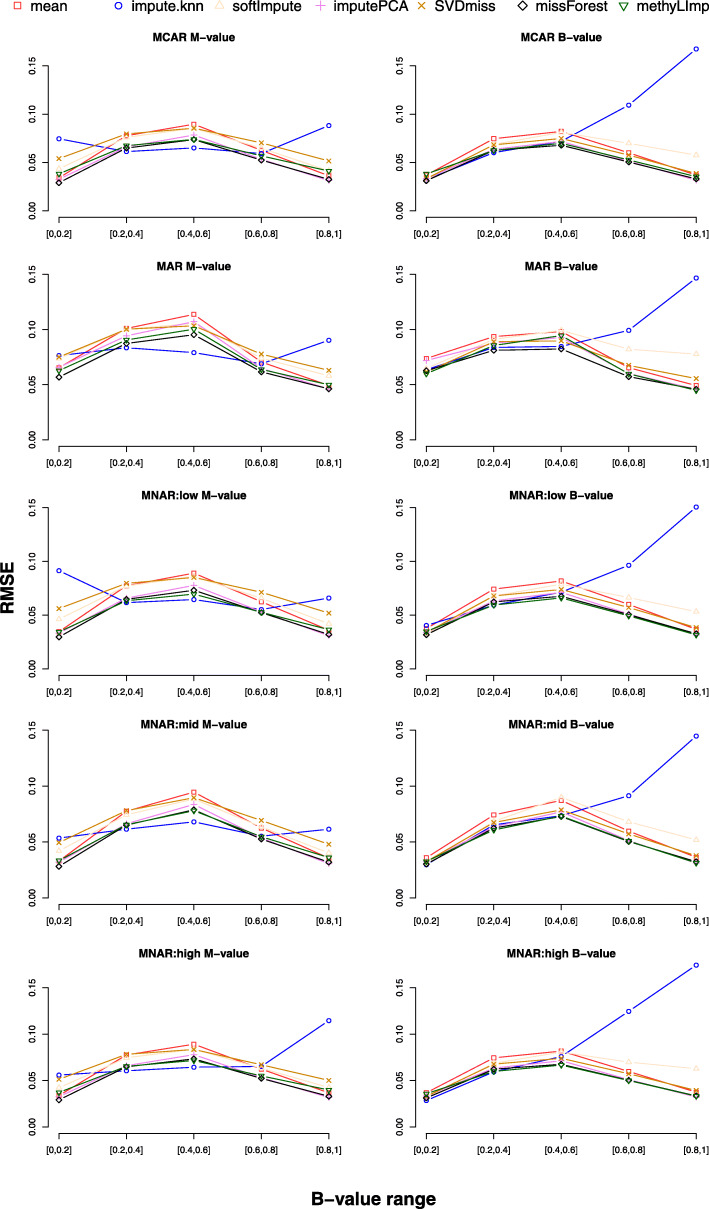
Healthy datasets. Average RMSE with respect to *β*-value range. Average RMSE for *M*-value and *β*-value imputation with respect to different *β*-value ranges and with respect to the MCAR, MAR, MNAR (low, mid, high) missing data mechanisms

We can also notice that *methyLImp, SVDmiss* and *softImpute* benefit from the *β*-value representation, *impute.knn* is definitely more accurate on the *M*-value representation, while there is no clear preference for the remaining methods (MCAR tests in Tables 2a and b). The accuracy of *impute.knn* (originally designed for gene expression data imputation) on high-range values is clearly affected by the *β*-value representation (average RMSD w.r.t. *β*-value range in Fig. 1). On the contrary, *impute.knn* performances are more uniform for the *M*-value representation, although still not satisfactory in comparison to those of the other methods, highlighting once more the peculiarity of epigenetic versus transcriptional signals. The same trend is visible also in the disease datasets (Figure 4 in Additional file 1).

To conclude, we can notice that, except for *impute.knn*, imputation errors are not equally distributed over the range of *β*-values, being smaller at the extremes (average RMSD w.r.t. *β*-value range in Fig. 1). As already pointed out in [17], this can be explained by the well-known heteroscedasticity of *β*-values [12]. The behaviour of the *mean* approach is a clear evidence of such variability at the extremes.

### Imputation of MAR values

**Fig. 2:**
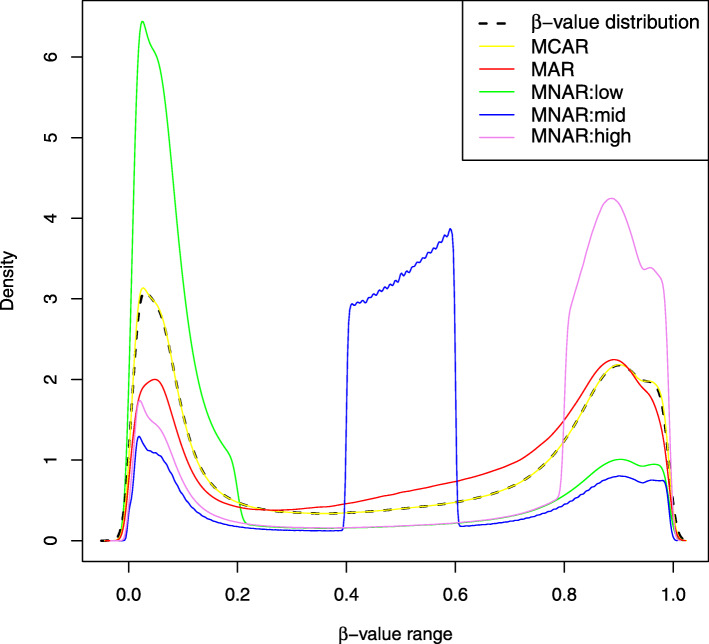
*β*-value distributions of different missingness mechanisms. Comparison of the *β*-value distribution against the distribution of simulated MCAR, MAR and MNAR missing values

**Fig. 3:**
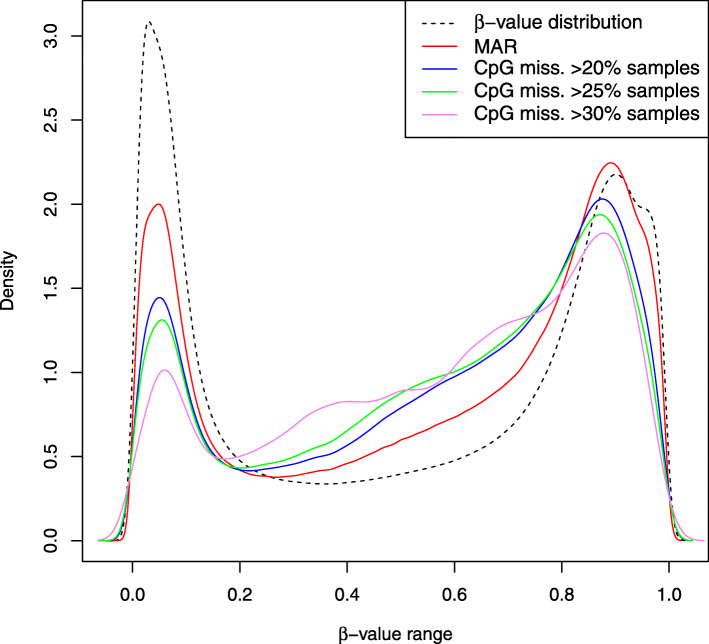
*β*-value distributions of CpGs with frequently missing values. Comparison of the *β*-value distribution against the *β*-value distribution of CpGs with missing values on >20*%*,>25*%*,>30*%* samples. MAR simulated distribution included

The average imputation performances on healthy and disease samples under the MAR assumption are summarized in Table 3a and b, respectively. We notice that the overall imputation accuracy of MAR missing values is significantly lower than that on MCAR values. This is very likely a consequence of the sampled MAR *β*-value distribution, which is shifted more toward the middle-range than the expected *β*-value distribution (Figs. 2 and 3). As discussed above, middle-range *β*-values are harder to impute due to their higher variability (see, for example, the *mean* performance) and, thus, the overall imputation accuracy for MAR missing values is, not surprisingly, lower. Since the MAR missing values have been sampled by using real data probability distribution, the average results in Table 3a and b (MAR tests) are probably a better indication of the expected imputation accuracies than the results on MCAR missing values.

**Table 3:** MAR missing values

	MAE		RMSE	
Method	*M*-value	B-value	*M*-value	B-value
(a) Healthy datasets				
mean	0.041 ±0.001	0.040 ±0.001^∗^	0.073 ±0.002	0.070 ±0.001^∗^
impute.knn	0.043 ±0.004^∗^	0.061 ±0.009	0.082 ±0.009^∗^	0.110 ±0.015
softImpute	0.042 ±0.002^∗^	0.043 ±0.007	0.077 ±0.005	0.082 ±0.017^∗^
imputePCA	0.037 ±0.001	0.036 ±0.001^∗^	0.069 ±0.002	0.066 ±0.002^∗^
SVDmiss	0.043 ±0.001	0.036 ±0.001^∗^	0.079 ±0.003	0.067 ±0.002^∗^
missForest	0.035 ±0.001	0.035 ±0.001^∗^	0.064 ±0.002	0.061 ±0.002^∗^
methyLImp	0.037 ±0.001	0.033 ±0.001^∗^	0.068 ±0.002	0.063 ±0.002^∗^
(b) Disease datasets				
mean	0.060 ±0.001	0.060 ±0.001^∗^	0.101 ±0.002	0.097 ±0.002^∗^
impute.knn	0.067 ±0.005^∗^	0.087 ±0.010	0.115 ±0.009^∗^	0.144 ±0.014
softImpute	0.062 ±0.003	0.065 ±0.011	0.106 ±0.006^∗^	0.116 ±0.026
imputePCA	0.054 ±0.001	0.053 ±0.001^∗^	0.095 ±0.002	0.090 ±0.002^∗^
SVDmiss	0.067 ±0.001	0.057 ±0.001^∗^	0.114 ±0.003	0.104 ±0.004^∗^
missForest	0.053 ±0.001	0.053 ±0.001^∗^	0.093 ±0.002	0.088 ±0.002^∗^
methyLImp	0.053 ±0.001	0.049 ±0.001^∗^	0.092 ±0.002	0.089 ±0.002^∗^

Average Mean Average Error (MAE) and Root Mean Square Error (RMSE) imputation performance ± standard deviation. For each method, the ^∗^ symbol indicates the measure (either *M*-value or *β*-value) for which the Wilcoxon signed-rank test *p*-value is <0.05. Best results per metric with respect to the Wilcoxon signed-rank test are highlighted in bold

Although the average imputation error is larger for MAR missing values, we can notice the same general performance trend, as observed in the MCAR tests: i) *β*-value imputation is generally more accurate than *M*-value imputation with the exception of *impute.knn*; ii) regression-based methods are on the average the best performing; iii) imputation accuracy is significantly better on healthy samples than on disease samples.

### Imputation of MNAR values

The average imputation performances on healthy and disease samples under the low-range MNAR assumption are summarized in Table 4a and b, respectively, those on the mid-range MNAR assumption in Table 5a and b, and those on the high-range MNAR assumption in Table 6a and b.

**Table 4:** MNAR:low missing values

	MAE		RMSE	
Method	*M*-value	B-value	*M*-value	B-value
(a) Healthy datasets				
mean	0.022 ±0.001^∗^	0.023 ±0.001	0.043 ±0.001^∗^	0.044 ±0.001
impute.knn	0.041 ±0.012	0.033 ±0.006^∗^	0.086 ±0.021	0.077 ±0.014^∗^
softImpute	0.026 ±0.002	0.023 ±0.003	0.052 ±0.006	0.046 ±0.010^∗^
imputePCA	0.019 ±0.001^∗^	0.020 ±0.001	0.039 ±0.002^∗^	0.039 ±0.001
SVDmiss	0.029 ±0.001	0.021 ±0.001^∗^	0.061 ±0.003	0.041 ±0.002^∗^
missForest	0.019 ±0.001^∗^	0.020 ±0.001	0.037 ±0.002^∗^	0.038 ±0.001
methyLImp	0.022 ±0.001^∗^	0.019 ±0.001	0.040 ±0.002^∗^	0.039 ±0.002
(b) Disease datasets				
mean	0.036 ±0.001^∗^	0.037 ±0.001	0.068 ±0.002^∗^	0.069 ±0.002
impute.knn	0.063 ±0.014	0.048 ±0.008^∗^	0.120 ±0.020	0.102 ±0.015^∗^
softImpute	0.040 ±0.005	0.036 ±0.004^∗^	0.076 ±0.010	0.072 ±0.013^∗^
imputePCA	0.031 ±0.001^∗^	0.032 ±0.001	0.061 ±0.002^∗^	0.062 ±0.002
SVDmiss	0.047 ±0.001	0.035 ±0.001^∗^	0.089 ±0.003	0.070 ±0.003^∗^
missForest	0.031 ±0.001^∗^	0.032 ±0.001	0.060 ±0.002^∗^	0.061 ±0.002
methyLImp	0.032 ±0.001	0.028 ±0.001^∗^	0.063 ±0.003	0.058 ±0.002^∗^

Average Mean Average Error (MAE) and Root Mean Square Error (RMSE) imputation performance ± standard deviation. For each method, the ^∗^ symbol indicates the measure (either *M*-value or *β*-value) for which the Wilcoxon signed-rank test *p*-value is <0.05. Best results per metric with respect to the Wilcoxon signed-rank test are highlighted in bold

**Table 5:** MNAR:mid missing values

	MAE		RMSE	
Method	*M*-value	B-value	*M*-value	B-value
(a) Healthy datasets				
mean	0.053 ±0.001	0.051 ±0.001^∗^	0.082 ±0.001	0.076 ±0.001^∗^
impute.knn	0.041 ±0.002^∗^	0.050 ±0.004	0.067 ±0.005^∗^	0.085 ±0.010
softImpute	0.051 ±0.001	0.050 ±0.006^∗^	0.078 ±0.003	0.080 ±0.012^∗^
imputePCA	0.045 ±0.001	0.043 ±0.001^∗^	0.072 ±0.001	0.067 ±0.001^∗^
SVDmiss	0.052 ±0.001	0.043 ±0.001^∗^	0.081 ±0.002	0.069 ±0.002^∗^
missForest	0.044 ±0.001	0.042 ±0.001^∗^	0.068 ±0.001	0.064 ±0.001^∗^
methyLImp	0.044 ±0.001	0.040 ±0.001^∗^	0.068 ±0.001	0.064 ±0.001^∗^
(b) Disease datasets				
mean	0.076 ±0.001	0.072 ±0.001^∗^	0.109 ±0.001	0.101 ±0.001^∗^
impute.knn	0.060 ±0.002^∗^	0.073 ±0.006	0.091 ±0.005^∗^	0.116 ±0.010
softImpute	0.075 ±0.002	0.072 ±0.010^∗^	0.108 ±0.003	0.111 ±0.021^∗^
imputePCA	0.066 ±0.001	0.062 ±0.001^∗^	0.098 ±0.001	0.091 ±0.001^∗^
SVDmiss	0.075 ±0.001	0.064 ±0.001^∗^	0.112 ±0.002	0.100 ±0.002^∗^
missForest	0.066 ±0.001	0.062 ±0.001^∗^	0.098 ±0.001	0.090 ±0.001^∗^
methyLImp	0.065 ±0.001	0.057 ±0.001^∗^	0.095 ±0.002	0.088 ±0.001^∗^

Average Mean Average Error (MAE) and Root Mean Square Error (RMSE) imputation performance ± standard deviation. For each method, the ^∗^ symbol indicates the measure (either *M*-value or *β*-value) for which the Wilcoxon signed-rank test *p*-value is <0.05. Best results per metric with respect to the Wilcoxon signed-rank test are highlighted in bold

**Table 6:** MNAR:high missing values

	MAE		RMSE	
	*M*-value	B-value	*M*-value	B-value
(a) Healthy datasets				
mean	0.026 ±0.001^∗^	0.026 ±0.001	0.044 ±0.001^∗^	0.044 ±0.001
impute.knn	0.054 ±0.013^∗^	0.092 ±0.020	0.103 ±0.022^∗^	0.152 ±0.023
softImpute	0.028 ±0.002^∗^	0.033 ±0.010	0.049 ±0.005^∗^	0.063 ±0.026
imputePCA	0.022 ±0.001^∗^	0.022 ±0.001	0.039 ±0.001	0.038 ±0.001
SVDmiss	0.032 ±0.001	0.025 ±0.001^∗^	0.056 ±0.004	0.043 ±0.002^∗^
missForest	0.023 ±0.001^∗^	0.023 ±0.001	0.039 ±0.001	0.038 ±0.001^∗^
methyLImp	0.027 ±0.001	0.022 ±0.001^∗^	0.044 ±0.001	0.039 ±0.002^∗^
(b) Disease datasets				
mean	0.041 ±0.001^∗^	0.043 ±0.001	0.069 ±0.002^∗^	0.069 ±0.002
impute.knn	0.085 ±0.017^∗^	0.134 ±0.025	0.148 ±0.023^∗^	0.203 ±0.024
softImpute	0.044 ±0.005^∗^	0.053 ±0.018	0.075 ±0.009^∗^	0.098 ±0.043
imputePCA	0.035 ±0.001^∗^	0.036 ±0.001	0.061 ±0.002^∗^	0.061 ±0.002
SVDmiss	0.050 ±0.001	0.041 ±0.001^∗^	0.084 ±0.002	0.073 ±0.003^∗^
missForest	0.036 ±0.001^∗^	0.038 ±0.001	0.062 ±0.002^∗^	0.062 ±0.001
methyLImp	0.037 ±0.001	0.032 ±0.001^∗^	0.062 ±0.002	0.057 ±0.002^∗^

Average Mean Average Error (MAE) and Root Mean Square Error (RMSE) imputation performance ± standard deviation. For each method, the ^∗^ symbol indicates the measure (either *M*-value or *β*-value) for which the Wilcoxon signed-rank test *p*-value is <0.05. Best results per metric with respect to the Wilcoxon signed-rank test are highlighted in bold

The imputation performances on low-range and high-range MNAR values are statistically significantly better than those on MCAR values, while the imputation performances on mid-range MNAR values are even worse than those on MAR. This behaviour can again be explained as a consequence of the standard deviations of the *β*-values being compressed in the low and high ranges. In fact, we remark that *β*-values at the extreme of the range (either close to one or zero) correspond to situations where all or none of the copies of the CpG sites are methylated, indicating a very robust biological condition, which seems easier to predict than conditions where methylation status is diversified across cells. As a further evidence, the plots related to the MCAR assumption are basically indistinguishable from those related to the (low-range, mid-range and high-range) MNAR assumption (Fig. 1). The good performances shown in Table 4a and b (low-range MNAR tests) are thus only a consequence of the fact that a high number of missing values are in the low-range (see also Fig. 2). The same argument can be used for the results in Table 6a and b (high-range MNAR tests). On the contrary, the worse MAE and RMSE performances are a consequence of the high number of missing values in the mid-range (Tables 6a, b and Figure 2). We also remark that, although the MNAR tests may appear purely theoretical and not related to a real-word cases, they can still give us a picture of what we should expect in an extreme scenario. In particular, the take home message here is that, if for some reason we can assume MNAR missing values in DNA methylation data, we need to model explicitly such missing mechanism only if they are of type mid-range MNAR. All other cases can be considered as *ignorable*.

To conclude, note that in Table 4 (low-range MNAR tests on healthy samples) there are several good average performances and no method is highlighted in bold, which means that it is not possible to unambiguously identify the best performing method. Anyway, a closer look at the results of the Wilcoxon test shows that methyLImp and imputePCA perform better than the remaining approaches (data not shown). Also, differently from the other cases, we can see that *M*-value imputation are more accurate than *β*-value imputation according to the Wilcoxon test (low-range MNAR tests in Table 4a and b). On the other hand, except for this specific case, we can observe the same general performance trend previously discussed for both the MAR and MCAR tests.

## Discussion

In this work we have covered three different types of missing data mechanisms for DNA methylation data, represented with the two popular *M* and *β*-value representations, and we have compared the performances of seven computationally efficient imputation methods that are available under the popular R framework. The analysis essentially provides three general pieces of information.

1. Missing values lying in the mid-range methylation level are harder to impute than missing values close to the extremes of the range, i.e. values indicating that (almost) all the copies of the CpG site are uniformly methylated or uniformly non methylated. This is very likely a consequence of the higher variance of the methylation values in the intermediate ranges. Such scenario can have a deep impact in terms of performance expectations, if we assume that in our data a large number of missing values are of type MNAR and, in particular, lie in the *β*-value mid-range. In this case, imputation approaches for DNA methylation data need to model explicitly such missingness pattern. Unfortunately, we do not have any evidence assessing whether this is true or not in real DNA methylation data.
2. Since *M*-values show lower heteroscedasticity than *β*-values, we would expect overall better imputation performances on *M*-values than on *β*-values, at least for those imputation approaches that rely on linear models. However, despite this desirable statistical properties of the *M*-value representation, there is no immediate benefit in *M*-value imputation.
3. Methylation levels of CpGs that come with a higher probability of having a missing value (i.e. MAR type missing values) are generally harder to impute accurately. This seems to be a consequence of the *β*-value distributions of such (*highly missing*) CpGs, which are, again, more compressed into the mid-range in comparison to the expected *β*-value distribution. Due to such statistics, we can speculate that MAR missigness mechanisms need to be assumed for DNA methylation data. It is however hard to quantify the amount of MAR missing values in the data, since these cannot be easily distinguished from MCAR missing values. Furthermore, we can observe highly variable percentages of missing values in DNA methylation datasets (see Table 1), making it hard to even determine how many missing values in general we should expect in real data. However, we can suggest that DNA methylation applications that rely on *highly missing* CpGs (easily identified by statistical analysis of available data) need to expect imputation accuracy lower that average for such CpGs. Also in this case, *M*-value imputation does not offer any benefit.

These three general indications hold independently from the specific imputation method adopted. On the other hand, some methods seem to be more suitable than others for DNA methylation data imputation. In particular, in terms of imputation accuracy, performance comparison shows that, among the benchmarked approaches, the regression-based methods (i.e. *methyLimp* and *missForest*) are the best performing ones. In particular, the overall results in Table 7, averaged over all 57 (healthy and disease) benchmark datasets and over all missing value mechanisms, show that *methyLImp* is statistically significantly best performing, closely followed by *missForest* and *imputePCA*. More generally, as it can be seen in Tables 2a, b, 3a, b, 4a, b, 5a, b, and 6a, b, (all missingness models tests), *methyLImp* is statistically significantly better performing with respect to all types of missingness mechanisms. However, we remark that the *methyLImp* approach is not suitable on DNA methylation datasets that have a limited number of variables (CpGs) with complete observations, although this is unlikely to happen in real methylation data (see statistics in Table 2 in Additional File 2). It is not completely trivial to quantify the minimum amount of complete CpGs needed by methyLImp to achieve good performance, since sample size has also some influence in regression accuracy. However, by observing the performances in our tests, we can empirically state that at least few thousands of complete observations are needed to achieve a good level of imputation accuracy.

**Table 7:** Global imputation performances across all datasets (healthy and disease) and all missingness mechanisms

	MAE		RMSE	
Method	*M*-value	B-value	*M*-value	B-value
mean	0.041 ±0.001^∗^	0.040 ±0.001	0.068 ±0.001	0.066 ±0.001^∗^
impute.knn	0.052 ±0.008^∗^	0.068 ±0.011	0.095 ±0.014^∗^	0.119 ±0.016
softImpute	0.042 ±0.003	0.043 ±0.008^∗^	0.072 ±0.006	0.077 ±0.020^∗^
imputePCA	0.035 ±0.001	0.035 ±0.001^∗^	0.062 ±0.002	0.059 ±0.001^∗^
SVDmiss	0.046 ±0.001	0.037 ±0.001^∗^	0.079 ±0.003	0.065 ±0.002^∗^
missForest	0.036 ±0.001	0.036 ±0.001^∗^	0.061 ±0.002	0.059 ±0.002^∗^
methyLImp	0.037 ±0.001	0.033 ±0.001^∗^	0.062 ±0.002	0.058 ±0.002^∗^

For each method, the ^∗^ symbol indicates the measure (either *M*-value or *β*-value) for which the Wilcoxon signed-rank test *p*-value is <0.05. Best results per metric with respect to the Wilcoxon signed-rank test are highlighted in bold

Furthermore, data imputation is generally more accurate when DNA methylation levels are expressed as *β*-values. This holds true essentially for all benchmarked methods but *impute.knn*, which undoubtedly benefits more from the *M*-value representation. However, even on *M*-values, the general performances of *impute.knn* are often less accurate than those of the baseline *mean* approach. This leads us to conclude that *impute.knn*, although a good general imputation method, is not suitable for DNA methylation data imputation.

All these results hold for the 21k CpGs in the intersection between the 27k and the 450k Human Beadchips. It is thus natural to ask whether there is a significant difference between imputation performances on complete datasets (i.e. 450k Human Beadchips data, which include both Type I and Type II probes) and their 21k restriction (which include Type I probes only). Due to the large computational times required to process complete datasets, for comparison purposes we performed only a restricted number of tests (see Additional file 3). There are some clearly visible trends in such comparison tests:

- *impute.knn* performances are significantly lower on the complete datasets, irrespectively of the missingness model (MCAR, MAR, low/mid/high-range MNAR), data representation (*M*-value or *β*-value) and metric used for performance assessment (MAE or RMSE).
- For the remaining methods, MAE and RMSE do not highlight a clear improvment or worsening of the results when using the complete or the restricted datasets. Wilcoxon’s test shows that better performances with MAR missingness model are achieved on the complete datasets and with MNAR:mid on the restricted datasets. However, the absolute differences obtained with the two types of datasets are negligible in all cases (in the order of 10^−3^), i.e. biologically unimportant *per se* [27] and irrelevant with respect to downstream computations [17].

As a general conclusion, these comparisons indicate that the imputation performances on the restricted datasets can be considered as representative of the imputation performances on the complete datasets.

To conclude, in terms of computational resources, the mean-value based approaches are the best performing, both in terms of running time and memory requirements, while the regression-based approaches are the more demanding in terms of running time. In particular, the average running time and memory requirements summarized in Table 8 show that *SVDmiss* and *missForest* are the two most demanding methods in terms of memory usage and computation time, respectively. Both methods are virtually unusable for multiple imputations on complete DNA methylation datasets.

**Table 8:** Average time and memory usage

Method	Avg time	Avg RAM
mean	<1s	27MB
impute.knn	2s	81MB
softImpute	<1s	74MB
imputePCA	19s	204MB
SVDmiss	2m	4GB
missForest	18h	280MB
methyLImp	21m	129MB

## Conclusions

In conclusion, the consolidated and manufacturer encouraged practice to use *β*-value seems appropriate for DNA methylation data imputation. The choice of the best imputation method is somewhat more subtle and depends essentially on the available computational resources and the amount of missing values. Independently of the expected missingness mechanisms, regression-based methods provide on average more accurate estimates of the missing values. However, imputations with regression methods in the presence of limited computational resources can be a rather challenging task. In such cases, the simple *mean* approach can surprisingly be a better choice than more sophisticated methods.

## Methods

### Benchmark data

Benchmark datasets are taken from our previous study [17]. In short, we analysed 58 datasets from healthy (37 datasets, overall 1495 samples) and diseased (21 datasets, overall 386 samples) individuals on a variety of different tissues and ages. All the samples are from Illumina 450k Human Beadchip platform (GPL13534 in GEO) and have been obtained from the NCBI database Gene Expression Omnibus (GEO, [28]), see Table 1 for details.

The 450k Human Beadchips incorporates two different chemical assays, Type I and Type II probes, which exhibit different technical characteristics. As already done in [17], due to the high computational time required by some imputation methods, we pre-filtered all datasets in order to consider only the methylation sites in the intersection between the Illumina 27k and 450k Human Beadchips (approximatively 21k sites), all of Type I. In order to assess whether the 21k restriction is representative for the whole 450k Human Beadchip, we performed a reduced (owing to the computational costs of such tests) number of tests on the complete 450k benchmark data (Additional file 3).

### Benchmark imputation software

Benchmark imputation tools include: *mean, impute.knn, SVDmiss, softImpute, ImputePCA, missForest, methyLImp*, selected according to the following criteria: i) representative of the major imputation techniques described in literature; ii) requiring limited computational resources; iii) available as R implementations. The benchmarked methods can be roughly classified into three groups:

- **mean-value imputation approaches**: average observed values.
  - *mean*: replaces the missing value of a variable by averaging all the known values for that variable. This is the baseline imputation method for continuous variables. We use our own R implementation.
  - *impute.knn* [20]: replaces a missing element for a variable by averaging the non-missing values of its nearest neighbours. Originally designed for gene expression data imputation. We use the implementation available in the R ’impute’ package [29].
- **iterative soft-thresholding approaches**: replace missing values with some initial guess and then iteratively update, up to convergence, the missing elements with values generated by low-rank approximation of the input matrix.
  - *SVDmiss* [23]: uses soft-thresholding singular value decomposition (SVD) of the input matrix. General purpose imputation algorithm (tested on air pollution data) for continuous variables. We use the implementation available in the R ’SpatioTemporal’ package [30].
  - *softImpute* [21]: uses soft-thresholding singular value decomposition (SVD) of the input matrix. General purpose imputation algorithm (tested on artificial data) for continuous variables. We use the implementation available in the R ’softImpute’ package [31].
  - *imputePCA* [22]: implements a low-rank approximation version of the iterative principal component analysis (PCA) algorithm. General purpose imputation algorithm for continuous variables. We use the implementation available in the R ’missMDA’ package [32].
- **regression-based imputation approaches**: build a regression model from observed data.
  - *missForest* [24]: builds random forests regression trees. General purpose imputation algorithm (tested on a variety of biological datasets) that can deal with both continuous and categorical variables. We use the implementation available in the R ’missForest’ package [33].
  - *methyLImp* [17]: builds a linear model with observed data. Specifically designed for methylation data. Rationale of the approach: exploits the high degree of inter-sample correlations of methylation levels. We use the implementation available in the R ’methyLImp’ package [34].

The running times and memory requirements of the benchmarked tools are quite different among different classes (see Table 8). In general, the mean-based approaches are less demanding than the regression-based approaches. There are, however, also some within-classes differences. For example, all soft-thresholding methods require matrices decomposition (in particular, both *softImpue* and *SVDmiss* use SVD decomposition), which can be expected to be computationally intensive on large matrices. However, given the high variability of computational performances observed within such class, we conclude that computational performances are mostly affected by the specific implementation and not by the approach itself.

In our tests we used the R implementations of the benchmarked methods with default parameters and we did not make any prior assumption about the missingness patterns in the data. Furthermore, since there are situations that fail to produce imputation results, the general strategy was to ignore those values in performance evaluation. We remark that values that could not be imputed are quite rare in our tests, thus ignoring them does not significantly affect performance scores. In the following we review in detail the specific limitations of each imputation method and the rationale we consequently adopted for input pre-processing.

- *mean*: trivially, it cannot impute a missing CpG value if such CpG value is missing in all samples.
- *impute.knn*: same limitations as above for the *mean* approach. There may be other cases but these are not documented. When a value cannot be imputed it is set by default to zero. We tested *impute.knn* performances by removing all the *zero imputations* without noticing any dramatic improvement in performance accuracy. Thus, since it is quite complex to detect zero values that represent a *failed imputation* (the implementation does not state explicitly which are these values), we decided to ignore this problem. Such cases, overall, rarely occur into the set of tested CpGs.
- *SVDmiss*: same limitations as above for the *mean* approach. By default, in such situation the method stops without performing imputation. We preprocessed the input matrix by removing all completely missing columns.
- *imputePCA*: does not perform imputation if one column (observation) of the matrix has zero variance (after excluding missing values). We preprocessed the input matrix by removing all zero variance columns.
- *softImpute*: exactly the same behaviour as *mean*.
- *missForest*: can apparently perform imputation in all possible situations, including the case of matrices with entirely missing observation for one variable (CpG). However, surprisingly, it does not perform imputation on matrices containing a variable with only one observed value. We preprocessed the input matrix by removing all such variables.
- *methyLImp*: cannot impute values in the same situation as described for the *mean* approach. Furthermore, it cannot perform any imputation if the matrix has at least one missing value per column (never occurred in our tests).

### Definition of *β*-value and *M*-value

The Illumina Infinium Assay [35] utilizes a pair of probes to measure the intensities of the methylated and unmethylated alleles at each CpG site. The methylation level is then estimated by measuring the intensities of this pair of probes, across all cells in the sample tissue. The two measures commonly used to quantify methylation levels are: *β*-value and *M*-value.

The *β*-value is defined as the ratio between the methylated probe intensity and the overall intensity of both methylated and unmethylated alleles. Following the notation in [12], the *β*-value for an *i*-th interrogated CpG site is defined as:

1 $$ \beta_{i} = \frac{\max\left(x_{i}^{meth},0\right)}{\max\left(x_{i}^{meth},0\right)+\max\left(x_{i}^{unmeth},0\right) + \alpha}  $$

where $x_{i}^{meth}$ and $x_{i}^{unmeth}$ are the intensities measured by the *i*-th methylated and unmethylated probes, respectively, and *α* is a constant offset (by default, *α*=100) used to regularize the *β*-value when both $x_{i}^{meth}$ and $x_{i}^{unmeth}$ intensities are low. By definition, *β*-values can range between 0 and 1. A *β*-value equal to zero implies that all the copies of the CpG site in the sample are completely unmethylated, while a *β*-value equal to one indicates that all the copies are methylated.

The *M*-value is defined as the log2 ratio between the intensities of methylated and unmethylated probes:

2 $$ M_{i} = \log_{2} \left(\frac{\max\left(x_{i}^{meth},0\right)+\alpha}{\max\left(x_{i}^{unmeth},0\right) + \alpha} \right)  $$

where the constant offset *α* (by default 1) prevents large perturbations for small values of $x_{i}^{meth}$ and $x_{i}^{unmeth}$. The *M*-values can range from −*∞* to +*∞*.

As introduced already, the *β*-value measure provides a more intuitive interpretation of the methylation status than *M*-values and it is recommended by array producers [11]. On the other hand, the *M*-value representation has been proven more suitable for conducting differential methylation analyses, due to the severe heteroscedasticity of *β*-values for highly methylated or unmethylated CpG sites [12], i.e. the standard deviations of *β*-values are compressed in the low and high ranges and larger in the middle ranges. In [12] the authors show that for typical values of $x_{i}^{meth}$ and $x_{i}^{unmeth}$ the offsets *α* in Eqs. (1) and (2) have negligible effect on both the *β*-value and *M*-value measures. Thus, by simply ignoring the *α* offsets, the relationship between the *β*-value and *M*-value measures is a logit transformation.

3 $$ \beta_{i} = \frac{2^{M_{i}}}{2^{M_{i}}+1}  $$

and

4 $$ M_{i} = \log_{2} \left(\frac{\beta_{i}}{1-\beta_{i}}\right)  $$

### Missing values simulation procedure

Given the DNA methylation datasets in Table 1, we randomly added 3% missing values under the MCAR, MAR or MNAR missingness assumptions. The 3% amount has been chosen by considering the average percentage of missing values in our benchmark set (see Table 1). Figure 2 compares the *β*-value distribution in our 57 benchmark sets against the distribution of simulated missing *β*-values under different missingness assumptions. The exact missing value simulation procedure for each missingness model and the rationale behind it is detailed in the following.

**MCAR**. We assume that MCAR missing values are the direct consequence of random errors in experimental measurements, hence we simply randomly select 3% CpG sites from the DNA methylation data matrix. Figure 2 shows that, as expected, the *β*-values distribution of artificially introduced MCAR values coincides perfectly with the *β*-values distribution in the benchmark sets.

**MAR**. We assume that MAR missing values are the effect of CpG-specific probes that are more likely to fail to capture the target sequences. That is, the missing value probability depends on the (observed) CpG site but not on its (unobserved) methylation level. Indeed, statistical analysis of missing data in our benchmark set shows that there exist CpGs with a higher probability of observing missing values. For instance, on the 21k dataset restriction, 11% CpGs have missing values on more than 20% samples, 7% on more than 25% samples and 1% on more than 30% (observed frequencies). Using this observation as a starting point, we estimated from the observed frequencies in our benchmark data the probability for a CpG-specific value of being missing and used such probability distribution to randomly sample 3% positions in the DNA methylation matrix. Figure 2 shows the distribution of the sampled MAR values in comparison to the *β*-value distribution on the entire dataset. MAR missing values are slightly more dense in the [0.4,0.8] interval and slightly less in [0,0.2]. Such trend is more evident in Fig. 3, where we consider the *β*-values distribution of CpGs with frequently missing values. In Fig. 3, it is clear that *β*-values of CpGs that are more likely to present missing values are more concentrated in the [0.4,0.8] interval and much less in the [0,0.2] interval in comparison to both the *β*-value and sampled MAR distributions.

**MNAR**. We assume that MNAR missing values are a consequence of the methylation level. In order to efficiently simulate MNAR missing values we limited our investigation to three different ranges of methylation values, visibly distinct: low-range with *β*-value between 0 and 0.2 (MNAR:low), mid-range with *β*-value between 0.4 and 0.6 (MNAR:mid) and high-range with *β*-value between 0.8 and 1 (MNAR:high). For each range, we randomly sampled 3% positions in the DNA methylation data matrix assuming 70% missing probability for the chosen range. For instance, a sampled position in the low-range MNAR model has 70% probability of being in the [0,0.2]*β*-value range. The *β*-value distributions of such MNAR models are clearly distinguishable in Fig. 2. Although 70% missing probability may seem unrealistic, we remark that in practice we do not have any strong evidence of MNAR missing data in DNA methylation datasets, thus the chosen probability, as well as the chosen ranges, are adopted to test imputation performances in extreme MNAR scenarios.

For comparison purposes, the same artificially introduced missing values have been imputed both in the *β*-value and *M*-value representations. For a more robust performance assessment, we repeated 100 times the artificial introduction of missing values for each missingness model. Thus, for each dataset our tests required a total of 1000 imputations, 500 with respect to *β*-values and 500 with respect to *M*-values.

### Evaluation metrics

Performance evaluation has been done based on two accuracy measures.

The **RMSE** (Root Mean Square Error) metric measures the difference between the predicted/estimated, *P*, and true values, *T*.

$$RMSE(P,T) = \sqrt{\frac{\sum\limits_{i=1}^{n}(P_{i}-T_{i})^{2}}{n}} $$

The **MAE** (Mean Absolute Error) metric measures the absolute difference between the predicted and true values.

$$MAE(P,T) = \frac{\sum\limits_{i=1}^{n}\lvert P_{i}-T_{i} \rvert}{n} $$

Recall that, by Jensen’s inequality RMSE ≥ MAE. Although the two metrics seem almost equivalent, they are in fact complementary. Indeed, the RMSE metric is more suitable for performance ranking, since it gives higher weight than MAE to large errors, which are particularly undesirable in DNA methylation data imputation. On the other hand, MAE provides a more immediate interpretation of the results in comparison to RMSE. In particular, MAE gives an indication on the average error to be expected on the imputed value.

### Wilcoxon-testing procedure to assess statistically significantly better performances

We use the Wilcoxon signed-rank test [36] to assess whether there is a statistically significant difference between the performances of a pair of methods. The Wilcoxon signed-rank test is a nonparametric statistical hypothesis test that can be used to compare two related (i.e. paired) samples to assess whether their population mean ranks differ. It is an alternative to the paired Student’s t-test when it is not possible to assume that the distribution of the differences between two samples is normal. As nearly all rank tests, the Wilcoxon test is not transitive. We use the Wilcoxon test to assess the statistical significance of two distinct comparisons:

1. **intra-method comparison**: we compare the performances of the same imputation method on *β*-value vs *M*-value representations of the data, in order to detect whether there is a better performing data representation method-wise. The statistically significant results with better performance are marked with ^∗^ in the result tables.
2. **inter-method comparison**: we compare the performances of two distinct methods, on either *β*-value or *M*-value representations of the data. Table-wise, we highlight in bold the results of the method with the best performance. This was identified as the method whose performance is always statistically significantly higher than or comparable to that of other methods. We remark that, in many cases, when comparing two performances the Wilcoxon test does not detect a statistically significant difference. Thus, there is no case in which (table-wise) a performance is detected as statistically significantly better than all other performances. Therefore, we *relaxed* the definition of *best performance* by defining it as the performance that is never statistically significantly worse within a table.

Both in comparisons 1 and 2, the Wilcoxon test has been applied separately on MAE and RMSE. In order to compute the Wilcoxon signed-rank test we used the (paired) average (MAE or RMSE) performances of the imputation methods on the one hundred repetitions of the imputation tests. Additionally, we performed a *p*-value adjustment for multiple comparisons with the Benjamini-Hochberg (BH) procedure.

We remark that, since the Wilcoxon test draws statistical inference from the rank sum instead of the mean, it can happen that a performance is detected as statistically significantly better than another although the average performances of the former are worse than those of the latter. This typically happens when there are just few *outliers* that affect the overall average performance score but not the rank sum test.

We further remark that, since the Wilcoxon test is not transitive, when we use it to asses a comparison between multiple methods there can be cases where no method can be termed as best performing, even with our relaxed definition (one example is performance assessment in Table 4).

## Supplementary information

**Additional file 1** Detailed imputation results per dataset.

**Additional file 2** Dataset statistics.

**Additional file 3** Performance comparison between complete (450k) and restricted (21k) datasets.

## Data Availability

The datasets generated and/or analysed during the current study are available in the GEO repository https://www.ncbi.nlm.nih.gov/geo/. The GEO IDs of the datasets are available in Table 1.

## Abbreviations

CpG: 5’–C–phosphate–G–3’
MCAR: Missing completely at random
MAR: Missing at random
MNAR: Missing not at random
MAE: Mean absolute error
RMSE: Root mean square error
