# Supplementary material for: Methylation data imputation performances under different representations and missingness patterns
Source: BMC Bioinformatics. 2020 Jun 29;21:268. doi: 10.1186/s12859-020-03592-5 (PMC7325236; doi:10.1186/s12859-020-03592-5)
Supplement: Supplementary file 1 — Additional file 1 Detailed imputation results per dataset. [file 12859_2020_3592_MOESM1_ESM.pdf]

Performance report

1 Results: global performances

1.1 Healthy control samples

Method	Avg time (sec)	Avg RAM (Mb)
mean	< 1	34
softImpute	1	84
imputePCA	24	222
impute.knn	2	93
SVDmiss	153	3965
methyLImp	1796	132
missForest	88564	301

Table 1: Healthy samples. Average time and memory usage.

Table 2: Healthy samples. Global imputation performances.

	MAE		RMSE	
Method	M-value	B-value	M-value	B-value
mean	0.034±0.001	0.034±0.001*	0.059±0.001	0.057±0.001*
impute.knn	0.044±0.008*	0.059±0.010	0.083±0.014*	0.107±0.016
softImpute	0.036±0.002	0.036±0.006*	0.062±0.005	0.066±0.016*
imputePCA	0.030±0.001	0.029±0.001*	0.053±0.001	0.051±0.001*
SVDmiss	0.038±0.001	0.031±0.002*	0.068±0.003	0.054±0.002*
missForest	0.029±0.002	0.029±0.002*	0.051±0.002	0.049±0.001*
methyLImp	0.032±0.002	**0.028±0.002***	0.054±0.002	**0.050±0.002***

Table 3: Healthy samples. Imputation performance on **MCAR** type missing values.

	MAE		RMSE	
Method	M-value	B-value	M-value	B-value
mean	0.030±0.001*	0.030±0.001	0.051±0.001	0.050±0.001*
impute.knn	0.039±0.007*	0.059±0.012	0.079±0.015*	0.112±0.019
softImpute	0.031±0.002	0.032±0.006*	0.055±0.004	0.059±0.017*
imputePCA	0.025±0.001*	0.025±0.001	0.045±0.001	0.043±0.001*
SVDmiss	0.035±0.001	0.027±0.001*	0.063±0.002	0.048±0.002*
missForest	0.026±0.001	0.026±0.001*	0.044±0.003	0.043±0.002*
methyLImp	0.029±0.001	**0.025±0.001***	0.050±0.002	**0.047±0.002***

Table 4: Healthy samples. Imputation performance on **MAR** type missing values.

	MAE		RMSE	
Method	M-value	B-value	M-value	B-value
mean	0.041±0.001	0.040±0.001*	0.073±0.002	0.070±0.001*
impute.knn	0.043±0.004*	0.061±0.009	0.082±0.009*	0.110±0.015
softImpute	0.042±0.002*	0.043±0.007	0.077±0.005	0.082±0.017*
imputePCA	0.037±0.001	0.036±0.001*	0.069±0.002	0.066±0.002*
SVDmiss	0.043±0.001	0.036±0.001*	0.079±0.003	0.067±0.002*
missForest	0.035±0.001	0.035±0.001*	0.064±0.002	**0.061±0.002***
methyLImp	0.037±0.001	**0.033±0.001***	0.068±0.002	**0.063±0.002***

Table 5: Healthy samples. Imputation performance on **MNAR:low** type missing values.

	MAE		RMSE	
Method	M-value	B-value	M-value	B-value
mean	0.022±0.001*	0.023±0.001	0.043±0.001*	0.044±0.001
impute.knn	0.041±0.012	0.033±0.006*	0.086±0.021	0.077±0.014*
softImpute	0.026±0.002	0.023±0.003	0.052±0.006	0.046±0.010*
imputePCA	0.019±0.001*	0.020±0.001	0.039±0.002*	0.039±0.001
SVDmiss	0.029±0.001	0.021±0.001*	0.061±0.003	0.041±0.002*
missForest	0.019±0.001*	0.020±0.001	0.037±0.002*	0.038±0.001
methyLImp	0.022±0.001*	0.019±0.001	0.040±0.002*	0.039±0.002

Table 6: Healthy samples. Imputation performance on **MNAR:mid** type missing values.

	MAE		RMSE	
Method	M-value	B-value	M-value	B-value
mean	0.053±0.001	0.051±0.001*	0.082±0.001	0.076±0.001*
impute.knn	0.041±0.002*	0.050±0.004	0.067±0.005*	0.085±0.010
softImpute	0.051±0.001	0.050±0.006*	0.078±0.003	0.080±0.012*
imputePCA	0.045±0.001	0.043±0.001*	0.072±0.001	0.067±0.001*
SVDmiss	0.052±0.001	0.043±0.001*	0.081±0.002	0.069±0.002*
missForest	0.044±0.001	0.042±0.001*	0.068±0.001	0.064±0.001*
methyLImp	0.044±0.001	**0.040±0.001***	0.068±0.001	**0.064±0.001***

Table 7: Healthy samples. Imputation performance on **MNAR:high** type missing values.

	MAE		RMSE	
Method	M-value	B-value	M-value	B-value
mean	0.026±0.001*	0.026±0.001	0.044±0.001*	0.044±0.001
impute.knn	0.054±0.013*	0.092±0.020	0.103±0.022*	0.152±0.023
softImpute	0.028±0.002*	0.033±0.010	0.049±0.005*	0.063±0.026
imputePCA	0.022±0.001*	0.022±0.001	0.039±0.001	0.038±0.001
SVDmiss	0.032±0.001	0.025±0.001*	0.056±0.004	0.043±0.002*
missForest	0.023±0.001*	0.023±0.001	0.039±0.001	0.038±0.001*
methyLImp	0.027±0.001	**0.022±0.001***	0.044±0.001	**0.039±0.002***

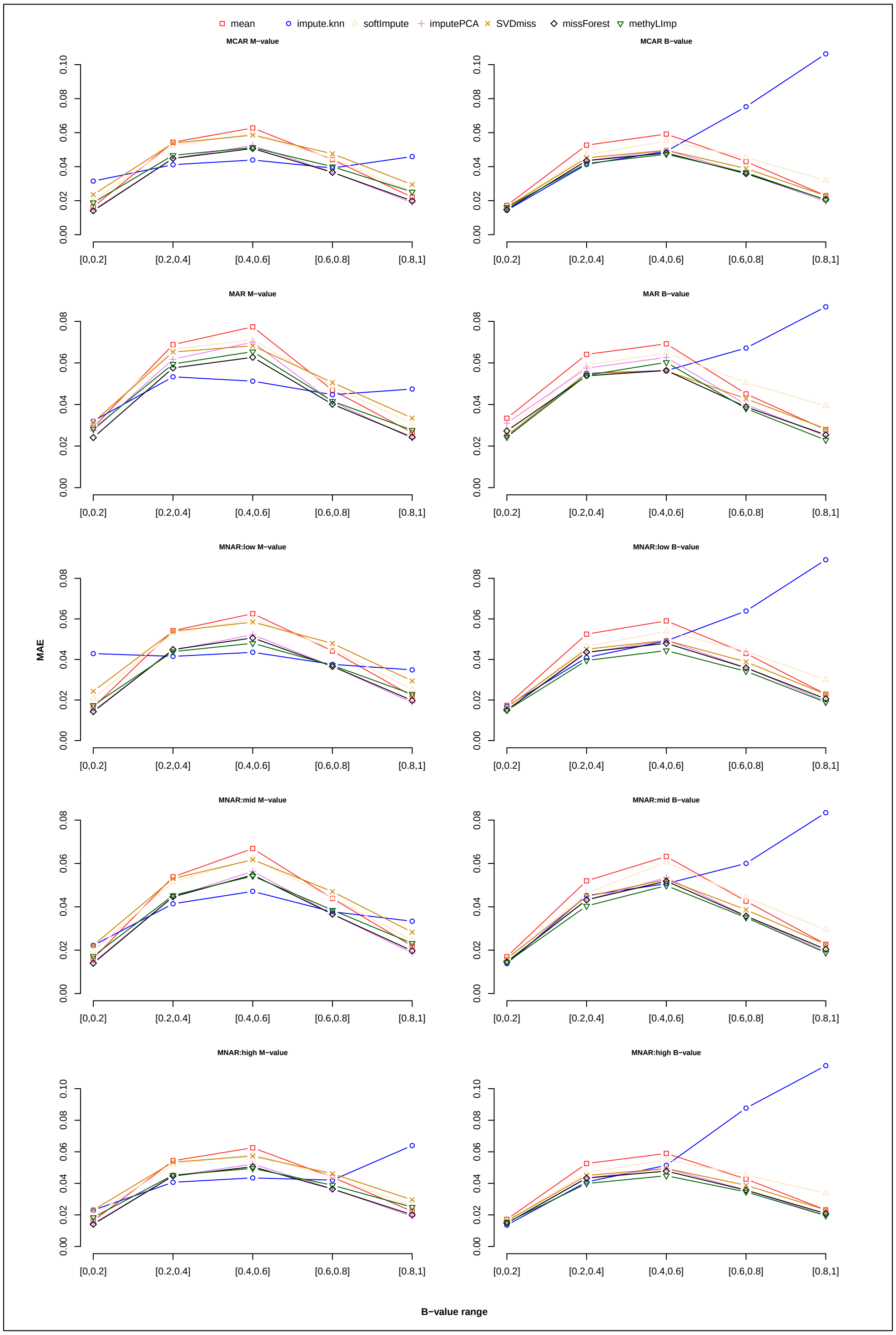

Figure 1: Healthy samples. MAE imputation performances with respect to B-value range.

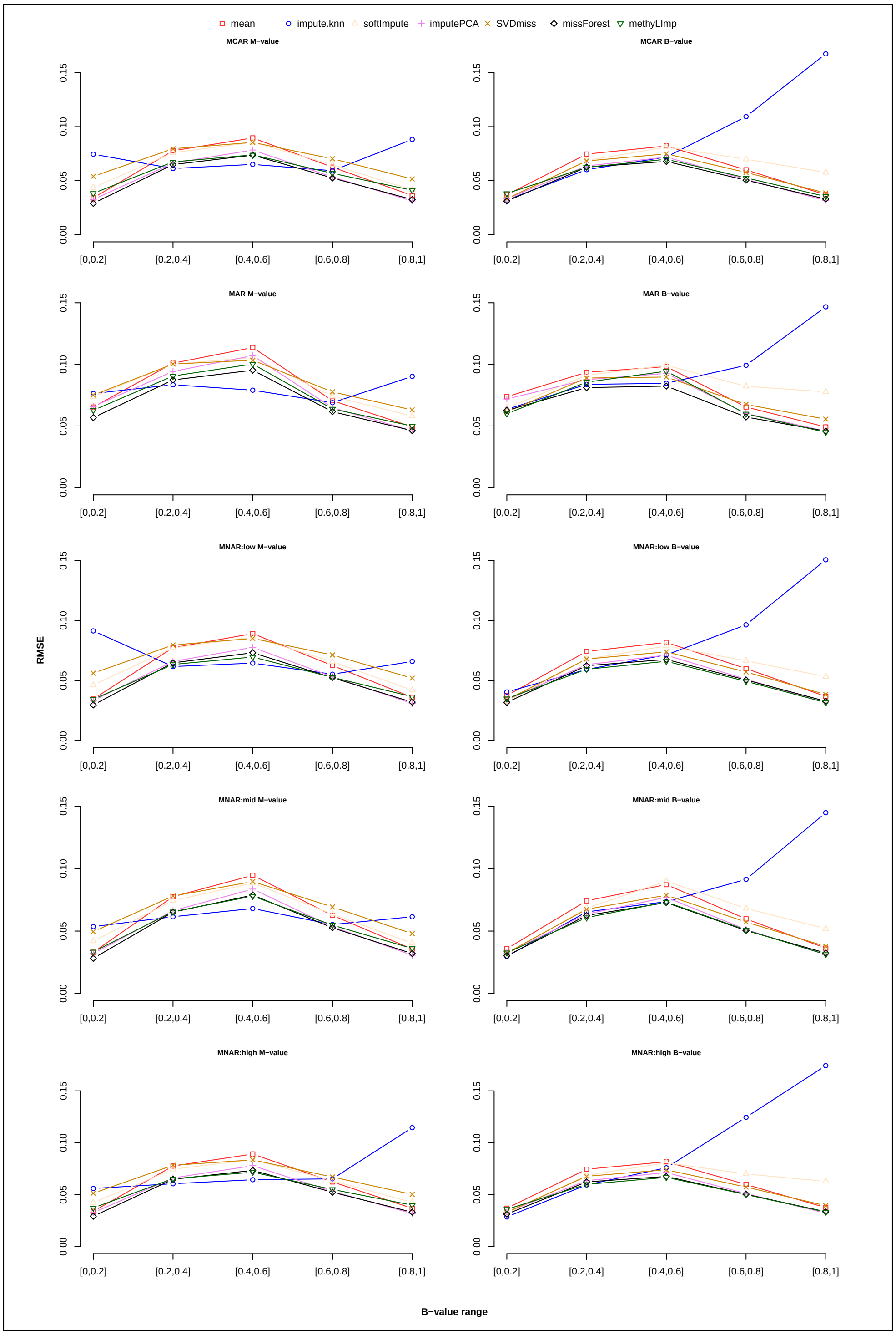

Figure 2: Healthy samples. RMSE imputation performances with respect to B-value range.

1.2 Disease case samples

Method	Avg time (sec)	Avg RAM (Mb)
mean	< 1	15
softImpute	< 1	57
imputePCA	9	173
impute.knn	1	62
SVDmiss	60	4077
methyLImp	357	123
missForest	25170	242

Table 8: Disease samples. Average time and memory usage.

Table 9: Disease samples. Global imputation performances.

	MAE		RMSE	
Method	M-value	B-value	M-value	B-value
mean	0.052±0.002*	0.052±0.002	0.085±0.002	0.083±0.002*
impute.knn	0.067±0.009*	0.085±0.012	0.116±0.014*	0.141±0.016
softImpute	0.054±0.004	0.055±0.011*	0.090±0.007	0.098±0.026*
imputePCA	0.045±0.002	0.045±0.002	0.077±0.002	0.075±0.002*
SVDmiss	0.059±0.001	0.048±0.002*	0.098±0.002	0.085±0.003*
missForest	0.046±0.002*	0.045±0.002	0.077±0.002	0.074±0.002*
methyLImp	0.046±0.002	**0.041±0.002***	0.077±0.002	**0.072±0.002***

Table 10: Disease samples. Imputation performance on **MCAR** type missing values.

	MAE		RMSE	
Method	M-value	B-value	M-value	B-value
mean	0.048±0.001*	0.048±0.001	0.080±0.002	0.079±0.002*
impute.knn	0.059±0.008*	0.082±0.013	0.107±0.014*	0.142±0.018
softImpute	0.050±0.004	0.051±0.010*	0.084±0.007	0.091±0.026*
imputePCA	0.041±0.001*	0.041±0.001	0.072±0.002	0.070±0.002*
SVDmiss	0.055±0.001	0.045±0.001*	0.093±0.002	0.080±0.003*
missForest	0.042±0.001*	0.042±0.001	0.071±0.002	0.070±0.002*
methyLImp	0.043±0.001	**0.037±0.001***	0.074±0.002	**0.066±0.002***

Table 11: Disease samples. Imputation performance on **MAR** type missing values.

	MAE		RMSE	
Method	M-value	B-value	M-value	B-value
mean	0.060±0.001	0.060±0.001*	0.101±0.002	0.097±0.002*
impute.knn	0.067±0.005*	0.087±0.010	0.115±0.009*	0.144±0.014
softImpute	0.062±0.003	0.065±0.011	0.106±0.006*	0.116±0.026
imputePCA	0.054±0.001	0.053±0.001*	0.095±0.002	**0.090±0.002***
SVDmiss	0.067±0.001	0.057±0.001*	0.114±0.003	0.104±0.004*
missForest	0.053±0.001	0.053±0.001*	0.093±0.002	0.088±0.002*
methyLImp	0.053±0.001	**0.049±0.001***	0.092±0.002	**0.089±0.002***

Table 12: Disease samples. Imputation performance on **MNAR:low** type missing values.

	MAE		RMSE	
Method	M-value	B-value	M-value	B-value
mean	0.036±0.001*	0.037±0.001	0.068±0.002*	0.069±0.002
impute.knn	0.063±0.014	0.048±0.008*	0.120±0.020	0.102±0.015*
softImpute	0.040±0.005	0.036±0.004*	0.076±0.010	0.072±0.013*
imputePCA	0.031±0.001*	0.032±0.001	0.061±0.002*	0.062±0.002
SVDmiss	0.047±0.001	0.035±0.001*	0.089±0.003	0.070±0.003*
missForest	0.031±0.001*	0.032±0.001	0.060±0.002*	0.061±0.002
methyLImp	0.032±0.001	**0.028±0.001***	0.063±0.003	**0.058±0.002***

Table 13: Disease samples. Imputation performance on **MNAR:mid** type missing values.

	MAE		RMSE	
Method	M-value	B-value	M-value	B-value
mean	0.076±0.001	0.072±0.001*	0.109±0.001	0.101±0.001*
impute.knn	0.060±0.002*	0.073±0.006	0.091±0.005*	0.116±0.010
softImpute	0.075±0.002	0.072±0.010*	0.108±0.003	0.111±0.021*
imputePCA	0.066±0.001	0.062±0.001*	0.098±0.001	0.091±0.001*
SVDmiss	0.075±0.001	0.064±0.001*	0.112±0.002	0.100±0.002*
missForest	0.066±0.001	0.062±0.001*	0.098±0.001	0.090±0.001*
methyLImp	0.065±0.001	**0.057±0.001***	0.095±0.002	**0.088±0.001***

Table 14: Disease samples. Imputation performance on **MNAR:high** type missing values.

	MAE		RMSE	
Method	M-value	B-value	M-value	B-value
mean	0.041±0.001*	0.043±0.001	0.069±0.002*	0.069±0.002
impute.knn	0.085±0.017*	0.134±0.025	0.148±0.023*	0.203±0.024
softImpute	0.044±0.005*	0.053±0.018	0.075±0.009*	0.098±0.043
imputePCA	0.035±0.001*	0.036±0.001	0.061±0.002*	0.061±0.002
SVDmiss	0.050±0.001	0.041±0.001*	0.084±0.002	0.073±0.003*
missForest	0.036±0.001*	0.038±0.001	0.062±0.002*	0.062±0.001
methyLImp	0.037±0.001	**0.032±0.001***	0.062±0.002	**0.057±0.002***

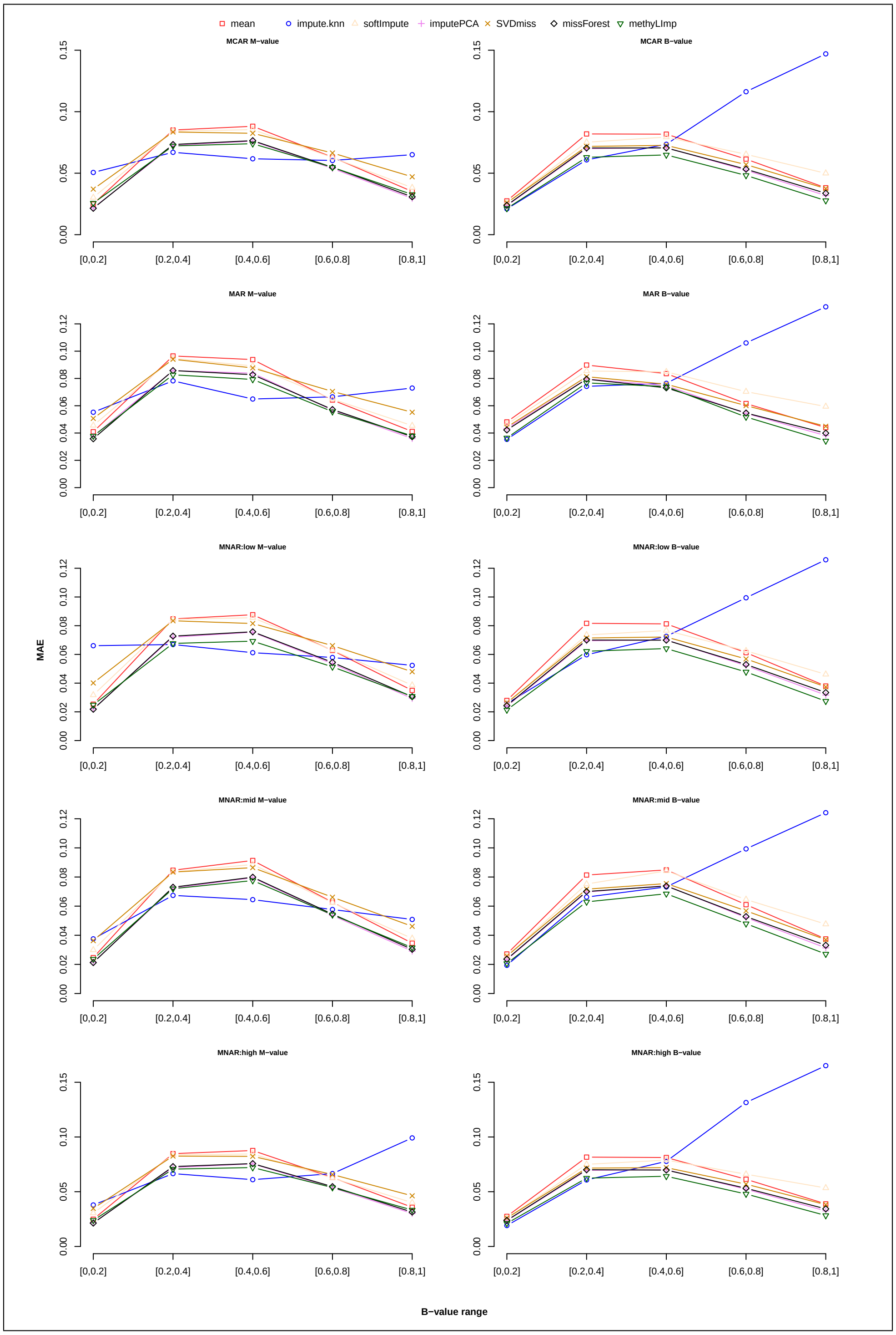

Figure 3: Disease samples. MAE imputation performances with respect to B-value range.

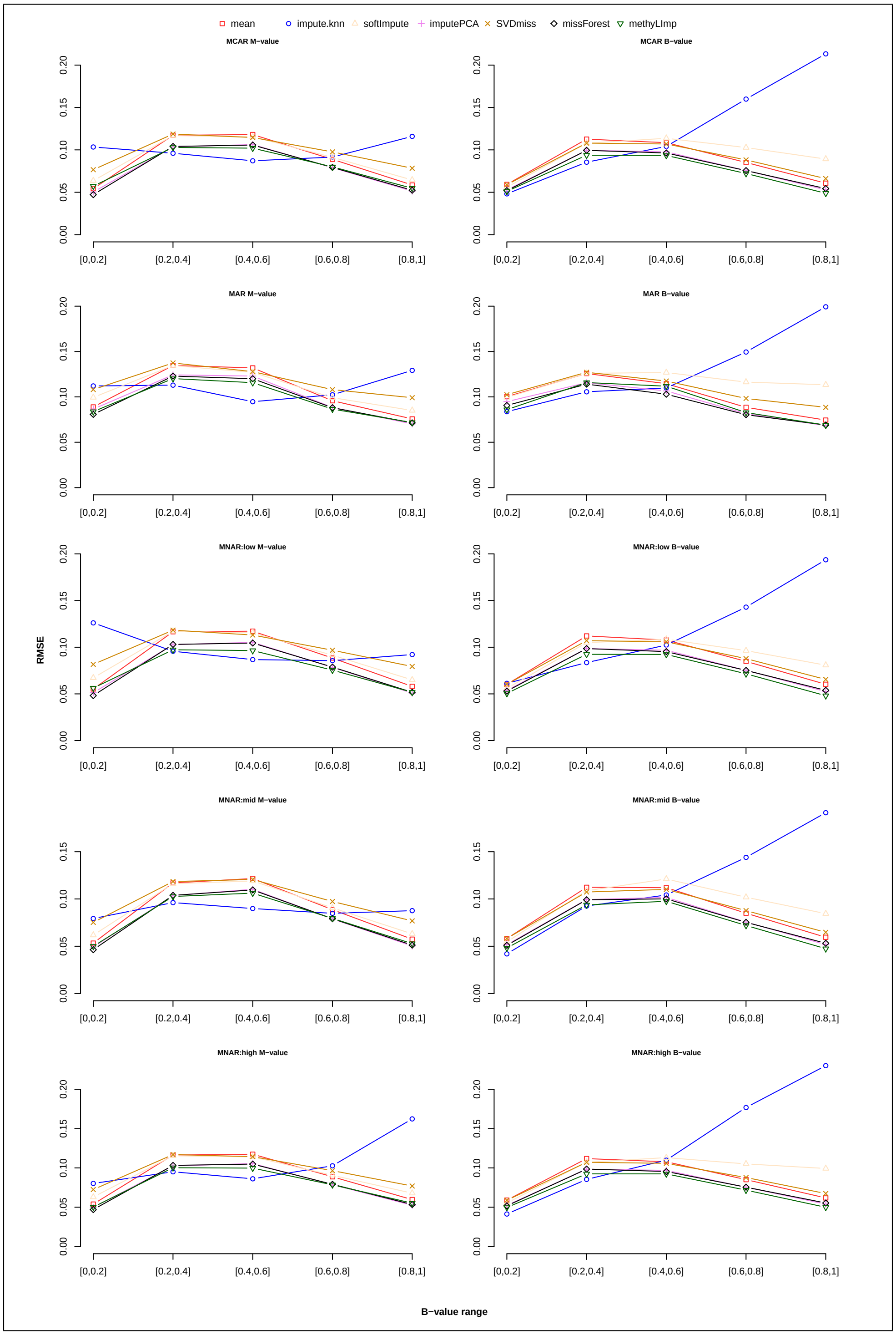

Figure 4: Disease samples. RMSE imputation performances with respect to B-value range.

2 Results: dataset-specific performances

2.1 GSE32146 (D1) - Colon mucosa - Crohn's disease - 10 samples

Method	Avg time (sec)	Avg RAM (Mb)
mean	< 1	9
softImpute	< 1	43
imputePCA	7	155
impute.knn	< 1	49
SVDmiss	18	3929
methyLImp	7	120
missForest	2978	285

Table 15: Dataset GSE32146 (D1). Average time and memory usage.

Table 16: Dataset GSE32146 (D1). Imputation performance on **MCAR** type missing values.

	MAE		RMSE	
Method	M-value	B-value	M-value	B-value
mean	0.030±0.001*	0.030±0.001	0.051±0.001	0.050±0.001*
softImpute	0.029±0.001	0.028±0.009*	0.048±0.001	0.051±0.027*
impute.knn	0.040±0.009*	0.064±0.015	0.083±0.020*	0.125±0.023
imputePCA	0.026±0.001	0.026±0.001	0.046±0.002	0.045±0.002*
SVDmiss	0.027±0.001	0.027±0.001*	0.046±0.002	0.046±0.002*
missForest	0.027±0.001*	0.027±0.001	0.046±0.001	0.046±0.001*
methyLImp	**0.024±0.001***	0.024±0.001	**0.040±0.002**	**0.040±0.002**

Table 17: Dataset GSE32146 (D1). Imputation performance on **MAR** type missing values.

	MAE		RMSE	
Method	M-value	B-value	M-value	B-value
mean	0.046±0.001	0.045±0.001*	0.080±0.002	0.078±0.002*
softImpute	0.042±0.003*	0.045±0.007	0.076±0.010*	0.085±0.020
impute.knn	0.044±0.005*	0.062±0.010	0.082±0.012*	0.116±0.016
imputePCA	0.043±0.001	0.043±0.001*	0.080±0.002	0.078±0.002*
SVDmiss	0.038±0.001*	0.038±0.001	0.070±0.002*	0.071±0.003
missForest	0.043±0.001	0.043±0.001*	0.078±0.002	0.075±0.002*
methyLImp	**0.038±0.001***	0.038±0.001	**0.068±0.002***	0.069±0.002

Table 18: Dataset GSE32146 (D1). Imputation performance on **MNAR:low** type missing values.

	MAE		RMSE	
Method	M-value	B-value	M-value	B-value
mean	0.022±0.001*	0.022±0.001	0.042±0.002*	0.044±0.002
softImpute	0.021±0.001	0.021±0.001*	0.038±0.002*	0.043±0.003
impute.knn	0.046±0.018	0.034±0.008*	0.105±0.033	0.083±0.019*
imputePCA	0.019±0.001*	0.020±0.001	0.040±0.002*	0.042±0.002
SVDmiss	0.020±0.001*	0.020±0.001	0.038±0.002	0.038±0.002
missForest	0.020±0.001*	0.020±0.001	0.039±0.002*	0.041±0.002
methyLImp	**0.017±0.001***	0.017±0.001	**0.033±0.002***	0.033±0.002

Table 19: Dataset GSE32146 (D1). Imputation performance on **MNAR:mid** type missing values.

	MAE		RMSE	
Method	M-value	B-value	M-value	B-value
mean	0.050±0.001	0.048±0.001*	0.075±0.001	0.070±0.001*
softImpute	0.048±0.001	0.041±0.002*	0.069±0.001	0.064±0.002*
impute.knn	**0.037±0.002***	0.044±0.005	0.059±0.006*	0.077±0.012
imputePCA	0.042±0.001	0.040±0.001*	0.068±0.001	0.063±0.001*
SVDmiss	0.043±0.001	0.040±0.001*	0.065±0.001	0.064±0.002*
missForest	0.045±0.001	0.043±0.001*	0.070±0.001	0.064±0.001*
methyLImp	0.038±0.001	**0.037±0.001***	0.058±0.001	**0.057±0.001***

Table 20: Dataset GSE32146 (D1). Imputation performance on **MNAR:high** type missing values.

	MAE		RMSE	
Method	M-value	B-value	M-value	B-value
mean	0.024±0.001*	0.025±0.001	0.040±0.001*	0.041±0.001
softImpute	0.024±0.001	0.026±0.013*	0.039±0.001*	0.047±0.038
impute.knn	0.067±0.023*	0.113±0.029	0.138±0.037*	0.192±0.032
imputePCA	0.021±0.001*	0.021±0.001	0.036±0.001*	0.037±0.001
SVDmiss	0.023±0.001*	0.024±0.001	0.040±0.002*	0.041±0.002
missForest	0.022±0.001*	0.023±0.001	0.037±0.001*	0.038±0.001
methyLImp	**0.020±0.001***	0.020±0.001	**0.034±0.001***	0.034±0.001

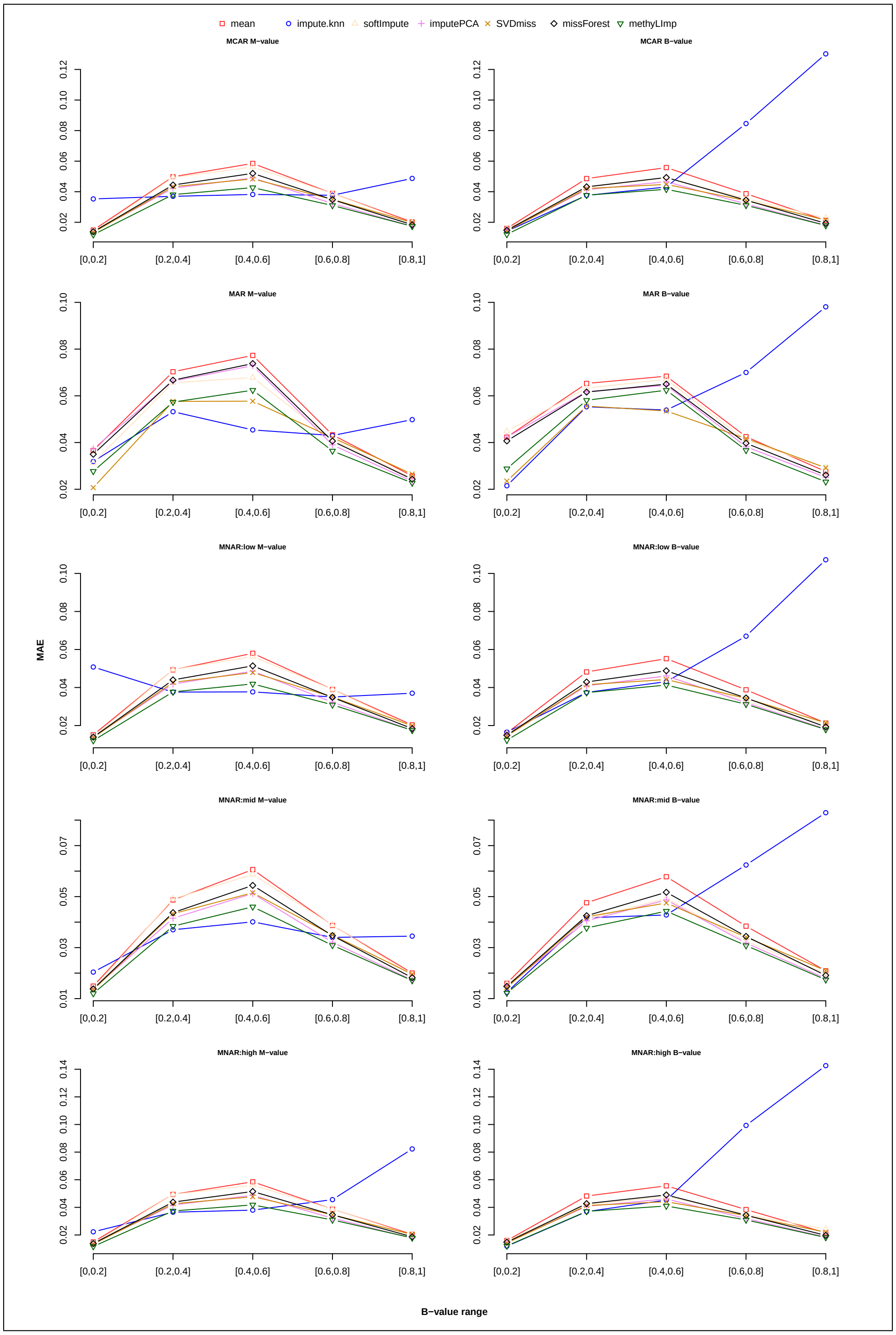

Figure 5: Dataset GSE32146 (D1). MAE imputation performances with respect to B-value range.

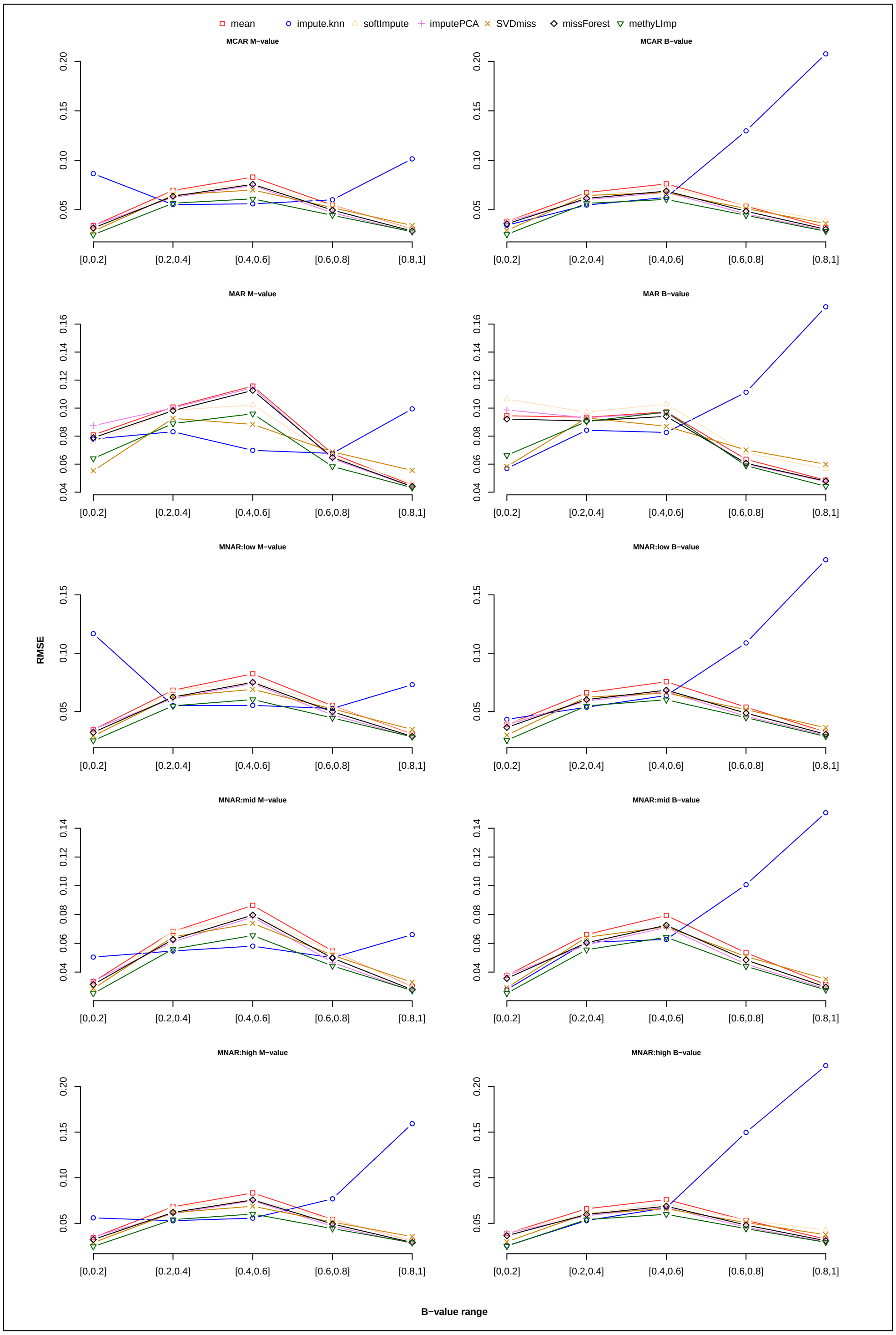

Figure 6: Dataset GSE32146 (D1). RMSE imputation performances with respect to B-value range.

2.2 GSE32146 (D2) - Colon mucosa - Ulcerative colitis - 5 samples

Method	Avg time (sec)	Avg RAM (Mb)
mean	< 1	4
softImpute	< 1	41
imputePCA	7	142
impute.knn	< 1	42
SVDmiss	10	3726
methyLImp	2	124
missForest	300	250

Table 21: Dataset GSE32146 (D2). Average time and memory usage.

Table 22: Dataset GSE32146 (D2). Imputation performance on **MCAR** type missing values.

	MAE		RMSE	
Method	M-value	B-value	M-value	B-value
mean	0.037±0.001	0.037±0.001	0.057±0.002	0.057±0.002*
softImpute	0.037±0.010*	0.065±0.040	0.060±0.020*	0.127±0.095
impute.knn	0.070±0.015*	0.112±0.025	0.125±0.024*	0.178±0.028
imputePCA	0.034±0.001*	0.034±0.001	0.053±0.002	0.053±0.002*
SVDmiss	0.070±0.002	0.038±0.001*	0.105±0.003	0.061±0.003*
missForest	0.035±0.001*	0.035±0.001	0.055±0.002	0.054±0.002*
methyLImp	**0.031±0.001***	0.032±0.001	**0.049±0.002***	0.049±0.002

Table 23: Dataset GSE32146 (D2). Imputation performance on **MAR** type missing values.

	MAE		RMSE	
Method	M-value	B-value	M-value	B-value
mean	0.055±0.001*	0.056±0.001	0.089±0.003	0.089±0.002*
softImpute	0.051±0.003*	0.094±0.042	0.089±0.007*	0.180±0.086
impute.knn	0.082±0.011*	0.135±0.021	0.136±0.015*	0.199±0.020
imputePCA	0.050±0.001*	0.050±0.001	0.082±0.003	0.081±0.002*
SVDmiss	0.083±0.002	0.052±0.001*	0.123±0.003	0.091±0.003*
missForest	0.052±0.001*	0.052±0.001	0.084±0.002	0.084±0.002*
methyLImp	**0.045±0.001***	0.048±0.002	**0.076±0.003***	0.080±0.004

Table 24: Dataset GSE32146 (D2). Imputation performance on **MNAR:low** type missing values.

	MAE		RMSE	
Method	M-value	B-value	M-value	B-value
mean	0.026±0.001*	0.026±0.001	0.045±0.002*	0.047±0.002
softImpute	0.031±0.016*	0.033±0.015	0.056±0.032*	0.066±0.045
impute.knn	0.101±0.029	0.067±0.019*	0.173±0.032	0.136±0.027*
imputePCA	0.024±0.001*	0.025±0.001	0.043±0.002*	0.044±0.002
SVDmiss	0.079±0.003	0.029±0.001*	0.125±0.004	0.051±0.003*
missForest	0.025±0.001*	0.025±0.001	0.043±0.002*	0.045±0.002
methyLImp	**0.022±0.001***	0.023±0.001	**0.039±0.002***	0.039±0.002

Table 25: Dataset GSE32146 (D2). Imputation performance on **MNAR:mid** type missing values.

	MAE		RMSE	
Method	M-value	B-value	M-value	B-value
mean	0.055±0.001	0.054±0.001*	0.080±0.002	0.077±0.002*
softImpute	0.056±0.001*	0.078±0.034	0.080±0.002*	0.130±0.069
impute.knn	0.049±0.003*	0.070±0.008	0.073±0.007*	0.117±0.014
imputePCA	0.051±0.001	0.050±0.001*	0.076±0.002	0.072±0.002*
SVDmiss	0.065±0.001	0.050±0.001*	0.094±0.002	0.075±0.002*
missForest	0.052±0.001	0.051±0.001*	0.077±0.002	0.073±0.002*
methyLImp	0.049±0.001	**0.047±0.002***	0.071±0.002	**0.069±0.003***

Table 26: Dataset GSE32146 (D2). Imputation performance on **MNAR:high** type missing values.

	MAE		RMSE	
Method	M-value	B-value	M-value	B-value
mean	0.036±0.001*	0.036±0.001	0.052±0.002*	0.053±0.002
softImpute	0.034±0.016*	0.079±0.063	0.058±0.030*	0.157±0.130
impute.knn	0.176±0.027*	0.228±0.035	0.255±0.025*	0.290±0.026
imputePCA	0.032±0.001*	0.033±0.001	0.048±0.002*	0.049±0.001
SVDmiss	0.074±0.001	0.035±0.001*	0.106±0.002	0.058±0.003*
missForest	0.034±0.001*	0.035±0.001	0.050±0.002*	0.051±0.002
methyLImp	**0.029±0.001***	0.030±0.001	**0.044±0.002***	0.045±0.002

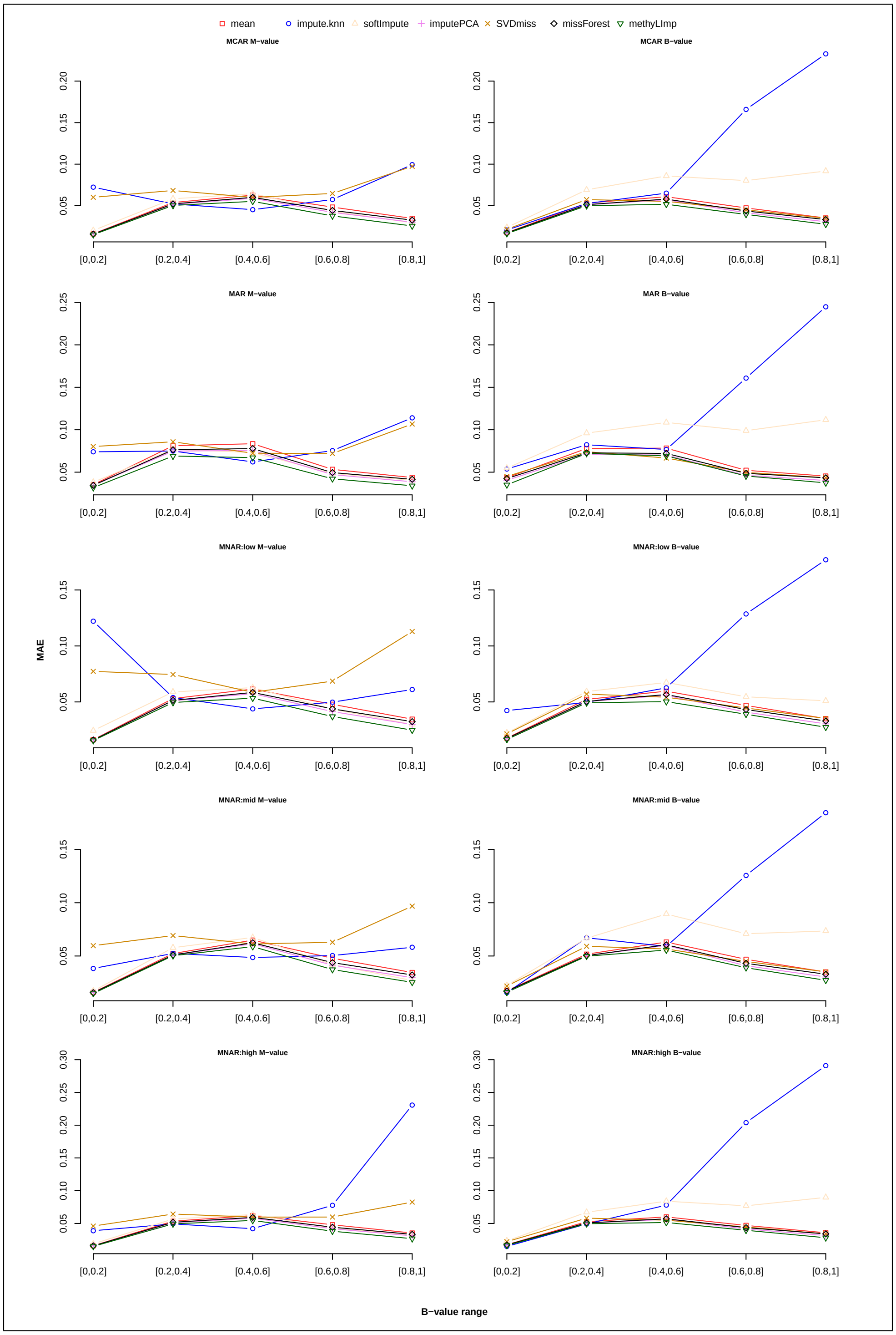

Figure 7: Dataset GSE32146 (D2). MAE imputation performances with respect to B-value range.

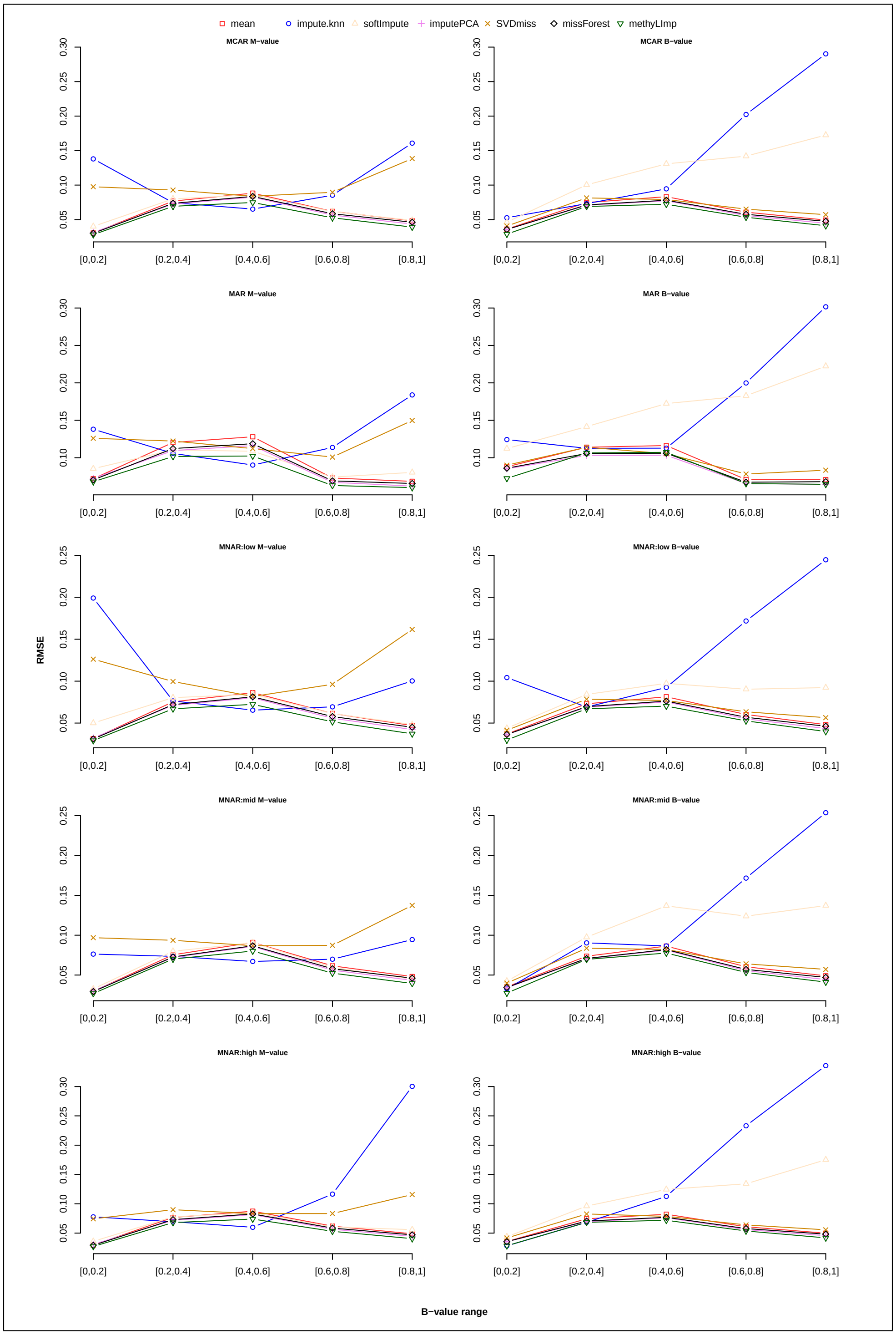

Figure 8: Dataset GSE32146 (D2). RMSE imputation performances with respect to B-value range.

2.3 GSE32146 (D3) - Colon - Normal - 10 samples

Method	Avg time (sec)	Avg RAM (Mb)
mean	< 1	9
softImpute	< 1	41
imputePCA	7	155
impute.knn	< 1	49
SVDmiss	18	3929
methyLImp	7	118
missForest	2924	287

Table 27: Dataset GSE32146 (D3). Average time and memory usage.

Table 28: Dataset GSE32146 (D3). Imputation performance on **MCAR** type missing values.

	MAE		RMSE	
Method	M-value	B-value	M-value	B-value
mean	0.026±0.001	0.026±0.001	0.045±0.001	0.044±0.001*
softImpute	0.025±0.001	0.025±0.004*	0.040±0.001	0.041±0.011*
impute.knn	0.040±0.009*	0.062±0.016	0.087±0.020*	0.123±0.024
imputePCA	0.023±0.001	0.023±0.001*	0.041±0.001	0.040±0.001*
SVDmiss	0.024±0.001*	0.024±0.001	0.041±0.002*	0.042±0.002
missForest	0.024±0.001	0.024±0.001	0.042±0.001	0.041±0.001*
methyLImp	0.022±0.001	**0.022±0.001***	**0.037±0.001**	**0.037±0.001**

Table 29: Dataset GSE32146 (D3). Imputation performance on **MAR** type missing values.

	MAE		RMSE	
Method	M-value	B-value	M-value	B-value
mean	0.043±0.001	0.042±0.001*	0.078±0.002	0.076±0.002*
softImpute	**0.034±0.001***	0.037±0.008	**0.060±0.002***	0.065±0.020
impute.knn	0.041±0.005*	0.061±0.011	0.080±0.012*	0.113±0.017
imputePCA	0.040±0.001	0.039±0.001*	0.075±0.002	0.073±0.002*
SVDmiss	0.035±0.001*	0.036±0.001	0.067±0.003*	0.070±0.003
missForest	0.041±0.001	0.040±0.001*	0.075±0.002	0.073±0.002*
methyLImp	0.036±0.001	0.035±0.001*	0.067±0.003	0.066±0.003*

Table 30: Dataset GSE32146 (D3). Imputation performance on **MNAR:low** type missing values.

	MAE		RMSE	
Method	M-value	B-value	M-value	B-value
mean	0.020±0.001*	0.020±0.001	0.039±0.002*	0.041±0.002
softImpute	0.019±0.001*	0.019±0.001	0.033±0.002*	0.034±0.002
impute.knn	0.050±0.017	0.032±0.008*	0.113±0.028	0.079±0.020*
imputePCA	0.018±0.001*	0.018±0.001	0.036±0.002*	0.038±0.002
SVDmiss	0.018±0.001*	0.019±0.001	0.034±0.002*	0.035±0.002
missForest	0.018±0.001*	0.019±0.001	0.036±0.002*	0.038±0.002
methyLImp	**0.016±0.001***	0.017±0.001	**0.031±0.002***	0.031±0.002

Table 31: Dataset GSE32146 (D3). Imputation performance on **MNAR:mid** type missing values.

	MAE		RMSE	
Method	M-value	B-value	M-value	B-value
mean	0.042±0.001	0.041±0.001*	0.068±0.001	0.062±0.001*
softImpute	0.039±0.001	0.039±0.009*	0.058±0.001	0.059±0.024*
impute.knn	**0.034±0.002***	0.042±0.005	0.055±0.006*	0.074±0.012
imputePCA	0.037±0.001	0.036±0.001*	0.062±0.001	0.057±0.001*
SVDmiss	0.036±0.001	0.036±0.001*	0.057±0.001*	0.058±0.002
missForest	0.040±0.001	0.038±0.001*	0.063±0.001	0.058±0.001*
methyLImp	0.035±0.001	**0.034±0.001***	0.054±0.001	**0.053±0.001***

Table 32: Dataset GSE32146 (D3). Imputation performance on **MNAR:high** type missing values.

	MAE		RMSE	
Method	M-value	B-value	M-value	B-value
mean	0.022±0.001*	0.022±0.001	0.037±0.001*	0.037±0.001
softImpute	0.022±0.001*	0.026±0.016	0.035±0.001*	0.046±0.049
impute.knn	0.065±0.023*	0.114±0.029	0.136±0.037*	0.193±0.030
imputePCA	0.019±0.001*	0.020±0.001	0.034±0.002*	0.034±0.001
SVDmiss	0.022±0.001*	0.022±0.001	0.037±0.002*	0.038±0.002
missForest	0.020±0.001*	0.021±0.001	0.034±0.001*	0.035±0.001
methyLImp	0.019±0.001	**0.019±0.001***	**0.032±0.002**	**0.032±0.002**

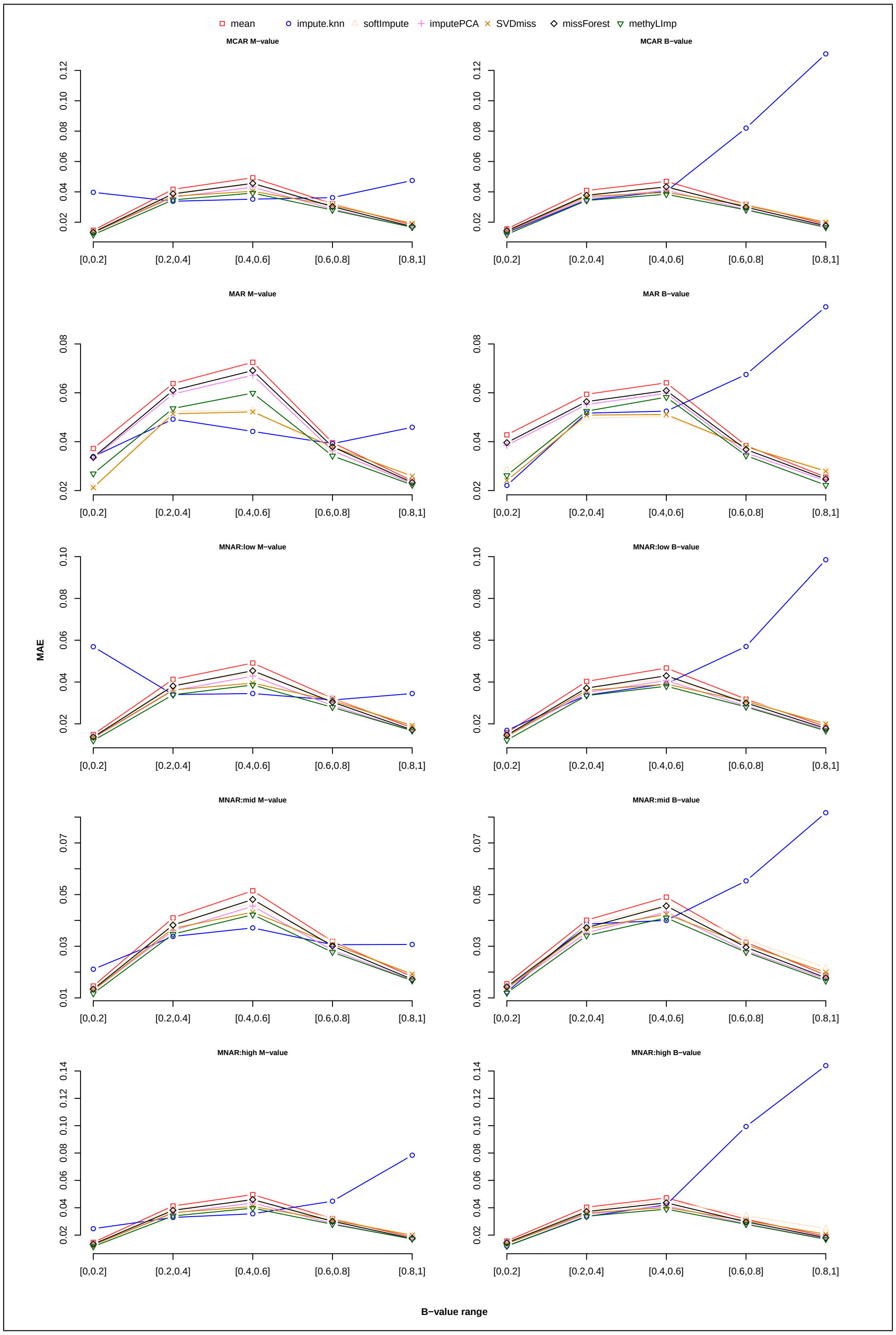

Figure 9: Dataset GSE32146 (D3). MAE imputation performances with respect to B-value range.

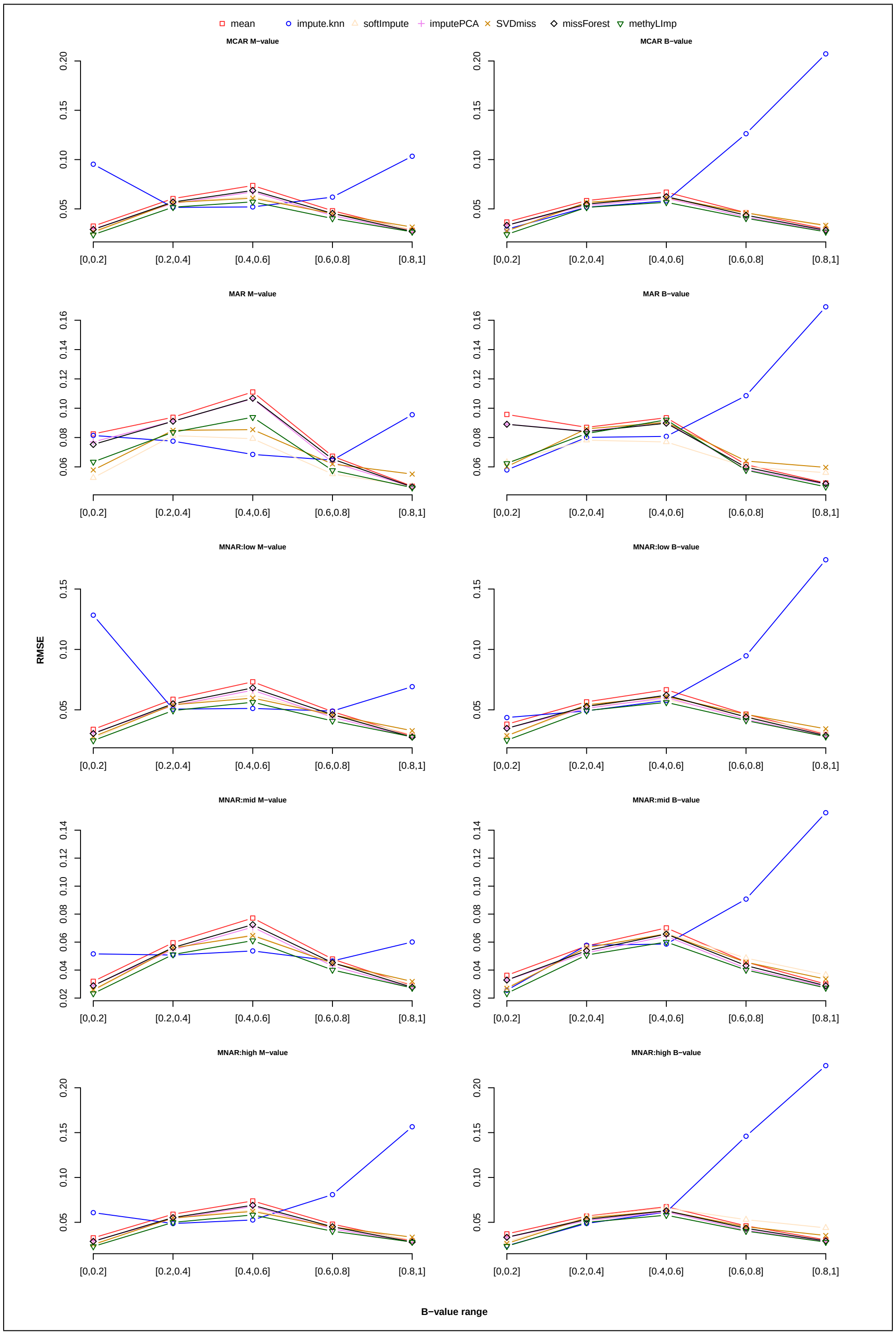

Figure 10: Dataset GSE32146 (D3). RMSE imputation performances with respect to B-value range.

2.4 GSE32148 (D4) - Blood - Normal - 19 samples

Method	Avg time (sec)	Avg RAM (Mb)
mean	< 1	16
softImpute	< 1	55
imputePCA	7	174
impute.knn	1	64
SVDmiss	73	4051
methyLImp	65	100
missForest	13317	139

Table 33: Dataset GSE32148 (D4). Average time and memory usage.

Table 34: Dataset GSE32148 (D4). Imputation performance on **MCAR** type missing values.

	MAE		RMSE	
Method	M-value	B-value	M-value	B-value
mean	0.022±0.001	0.022±0.001*	0.043±0.001	0.042±0.001*
softImpute	0.020±0.001	0.019±0.001*	0.038±0.001	0.037±0.001*
impute.knn	0.031±0.007*	0.046±0.012	0.080±0.017*	0.105±0.020
imputePCA	0.019±0.001	0.019±0.001	0.039±0.001	0.038±0.001*
SVDmiss	0.017±0.001*	0.017±0.001	0.033±0.001	0.033±0.001*
missForest	0.017±0.001*	0.018±0.001	0.033±0.001	0.033±0.001
methyLImp	**0.016±0.001***	0.017±0.001	**0.032±0.001***	0.032±0.001

Table 35: Dataset GSE32148 (D4). Imputation performance on **MAR** type missing values.

	MAE		RMSE	
Method	M-value	B-value	M-value	B-value
mean	0.039±0.001	0.038±0.001*	0.074±0.001	0.072±0.001*
softImpute	0.030±0.001*	0.035±0.001	0.059±0.003*	0.069±0.003
impute.knn	0.032±0.003*	0.038±0.006	0.071±0.009*	0.083±0.012
imputePCA	0.037±0.001	0.037±0.001*	0.074±0.001	0.072±0.001*
SVDmiss	**0.025±0.001***	0.026±0.001	0.056±0.002	**0.055±0.002***
missForest	0.029±0.001*	0.030±0.001	0.057±0.001	0.056±0.001*
methyLImp	0.032±0.001	0.031±0.001*	0.065±0.003	0.064±0.002*

Table 36: Dataset GSE32148 (D4). Imputation performance on **MNAR:low** type missing values.

	MAE		RMSE	
Method	M-value	B-value	M-value	B-value
mean	0.017±0.001*	0.017±0.001	0.036±0.001*	0.038±0.001
softImpute	0.015±0.001*	0.015±0.001	0.031±0.001*	0.033±0.001
impute.knn	0.024±0.008	0.023±0.004	0.071±0.022	0.065±0.013*
imputePCA	0.015±0.001*	0.015±0.001	0.034±0.001*	0.036±0.001
SVDmiss	0.022±0.007	0.013±0.001*	0.056±0.021	0.028±0.001*
missForest	0.013±0.001*	0.014±0.001	0.027±0.001*	0.028±0.001
methyLImp	**0.013±0.001***	0.013±0.001	**0.027±0.001***	0.027±0.001

Table 37: Dataset GSE32148 (D4). Imputation performance on **MNAR:mid** type missing values.

	MAE		RMSE	
Method	M-value	B-value	M-value	B-value
mean	0.050±0.001	0.047±0.001*	0.084±0.001	0.075±0.001*
softImpute	0.043±0.001	0.039±0.001*	0.068±0.002	0.065±0.001*
impute.knn	0.034±0.002*	0.037±0.003	0.061±0.006*	0.069±0.008
imputePCA	0.042±0.001	0.039±0.001*	0.077±0.001	0.068±0.001*
SVDmiss	0.032±0.001	**0.031±0.001***	0.055±0.001	**0.054±0.001***
missForest	0.037±0.001	0.035±0.001*	0.061±0.001	0.056±0.001*
methyLImp	0.034±0.001	0.033±0.001*	0.057±0.001	0.056±0.001*

Table 38: Dataset GSE32148 (D4). Imputation performance on **MNAR:high** type missing values.

	MAE		RMSE	
Method	M-value	B-value	M-value	B-value
mean	0.018±0.001*	0.018±0.001	0.035±0.001*	0.035±0.001
softImpute	0.017±0.001	0.017±0.001*	0.032±0.001	0.031±0.001*
impute.knn	0.035±0.010*	0.061±0.015	0.090±0.022*	0.131±0.021
imputePCA	0.016±0.001*	0.016±0.001	0.032±0.001*	0.032±0.001
SVDmiss	0.015±0.001*	0.015±0.001	0.029±0.001*	0.029±0.001
missForest	0.015±0.001*	0.015±0.001	**0.027±0.001***	0.028±0.001
methyLImp	**0.014±0.001***	0.015±0.001	**0.028±0.001***	0.028±0.001

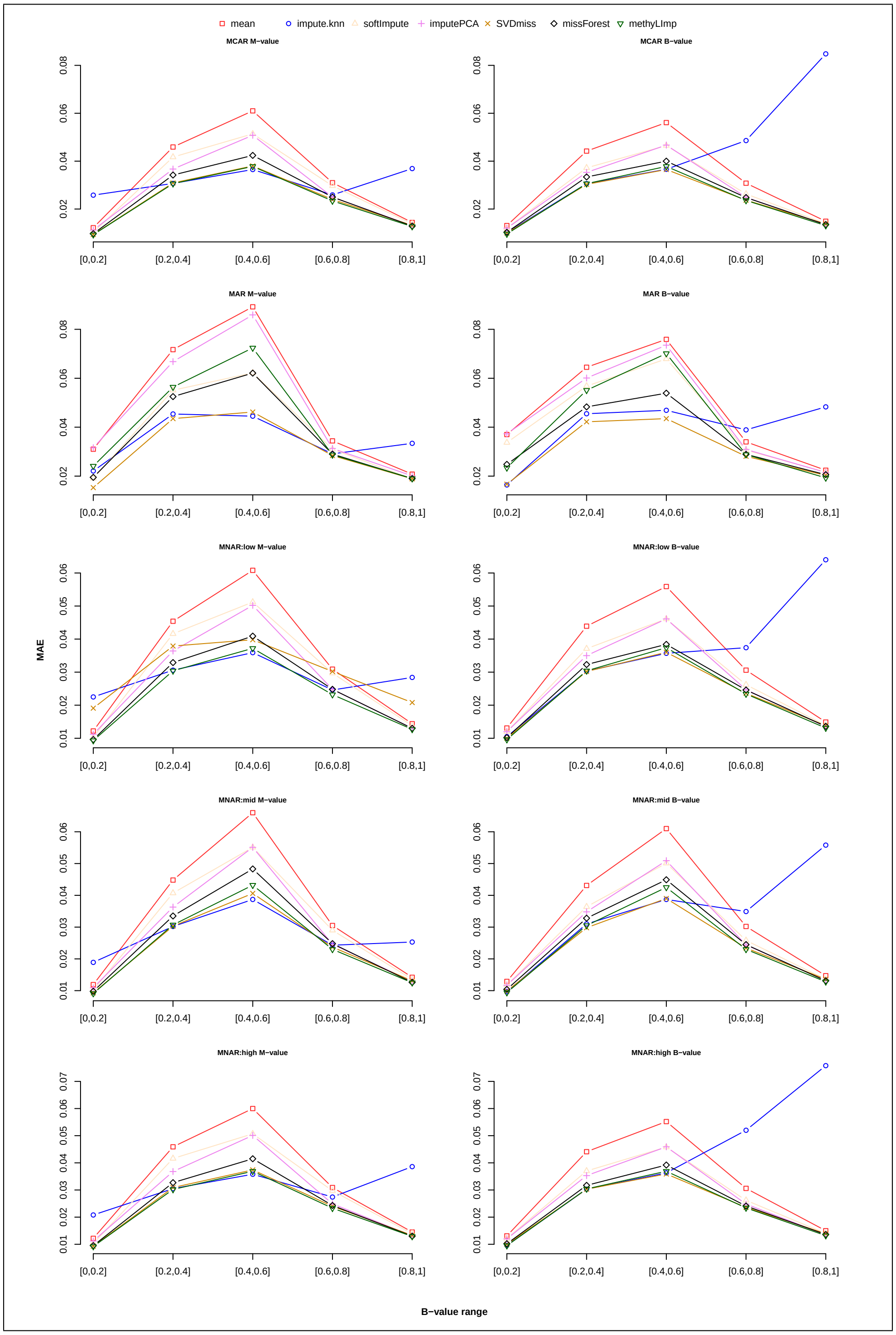

Figure 11: Dataset GSE32148 (D4). MAE imputation performances with respect to B-value range.

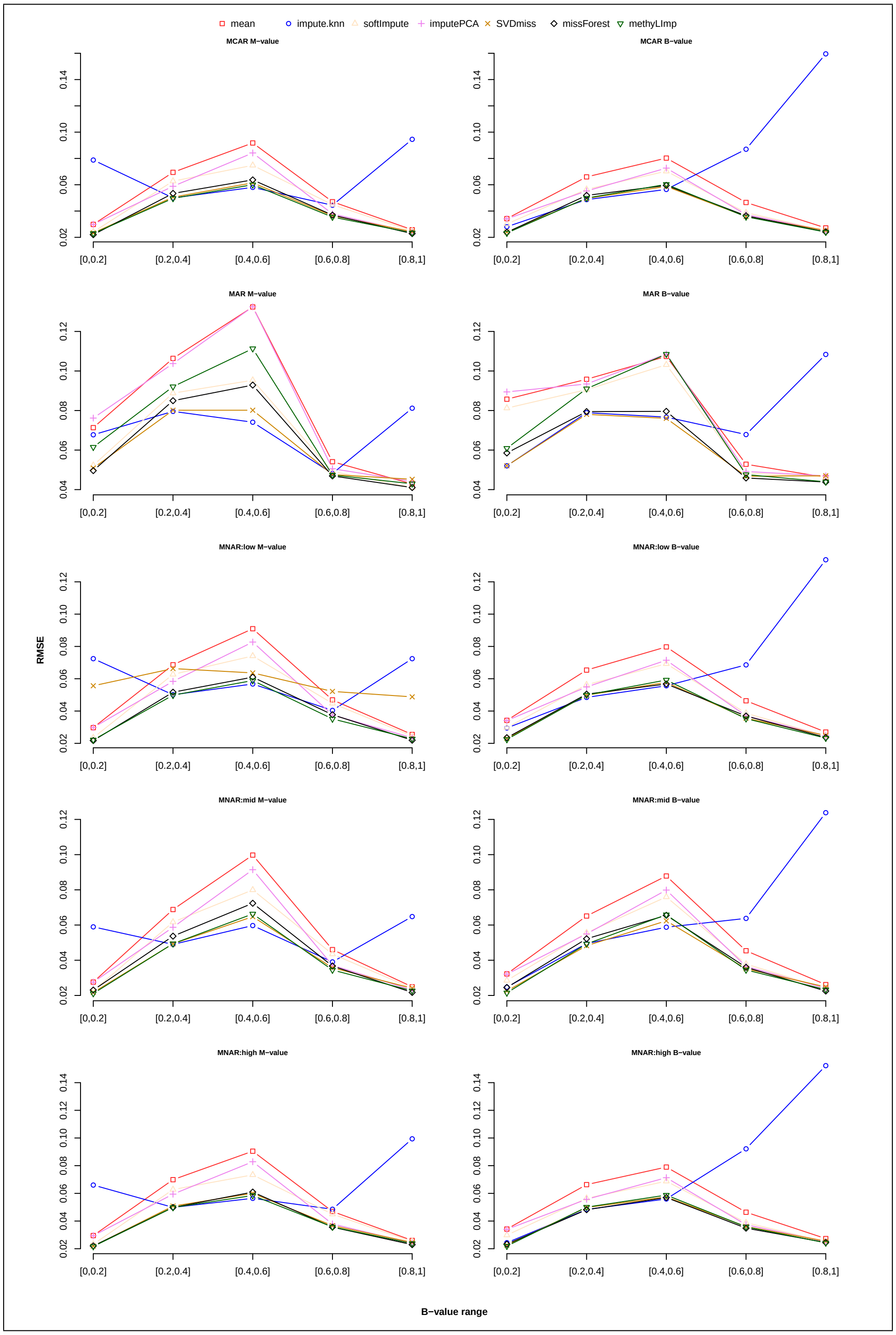

Figure 12: Dataset GSE32148 (D4). RMSE imputation performances with respect to B-value range.

2.5 GSE40005 (D5) - Blood - Normal - 12 samples

Method	Avg time (sec)	Avg RAM (Mb)
mean	< 1	10
softImpute	< 1	50
imputePCA	8	154
impute.knn	< 1	52
SVDmiss	27	4013
methyLImp	13	112
missForest	5170	288

Table 39: Dataset GSE40005 (D5). Average time and memory usage.

Table 40: Dataset GSE40005 (D5). Imputation performance on **MCAR** type missing values.

	MAE		RMSE	
Method	M-value	B-value	M-value	B-value
mean	0.028±0.001*	0.028±0.001	0.051±0.002	0.050±0.001*
softImpute	0.037±0.001	0.028±0.002*	0.070±0.004	0.048±0.007*
impute.knn	0.038±0.007*	0.066±0.016	0.082±0.018*	0.132±0.025
imputePCA	0.026±0.001	0.026±0.001*	0.049±0.002	0.048±0.001*
SVDmiss	0.043±0.001	0.030±0.001*	0.084±0.004	0.056±0.002*
missForest	0.027±0.001	0.027±0.001*	0.049±0.002	**0.047±0.001***
methyLImp	0.030±0.001	**0.026±0.001***	0.051±0.002	**0.047±0.002***

Table 41: Dataset GSE40005 (D5). Imputation performance on **MAR** type missing values.

	MAE		RMSE	
Method	M-value	B-value	M-value	B-value
mean	0.046±0.001	0.045±0.001*	0.086±0.002	0.080±0.002*
softImpute	0.054±0.002	**0.043±0.006***	0.100±0.004	**0.079±0.015***
impute.knn	0.046±0.004*	0.063±0.011	0.091±0.010*	0.121±0.018
imputePCA	0.044±0.001	0.044±0.001*	0.085±0.002	0.080±0.002*
SVDmiss	0.060±0.001	0.041±0.001*	0.111±0.003	0.083±0.003*
missForest	0.044±0.001	0.043±0.001*	0.083±0.002	**0.077±0.002***
methyLImp	0.046±0.001	**0.041±0.001***	0.084±0.002	0.079±0.003*

Table 42: Dataset GSE40005 (D5). Imputation performance on **MNAR:low** type missing values.

	MAE		RMSE	
Method	M-value	B-value	M-value	B-value
mean	0.021±0.001*	0.022±0.001	0.044±0.002*	0.045±0.002
softImpute	0.030±0.002	0.021±0.001*	0.070±0.006	**0.041±0.002***
impute.knn	0.035±0.012	0.033±0.008	0.083±0.026	0.084±0.020
imputePCA	0.020±0.001*	0.021±0.001	0.043±0.002*	0.044±0.002
SVDmiss	0.036±0.001	0.022±0.001*	0.089±0.006	0.047±0.002*
missForest	0.020±0.001*	0.021±0.001	0.041±0.002*	0.042±0.002
methyLImp	0.023±0.001	**0.020±0.001***	0.043±0.002	0.043±0.003

Table 43: Dataset GSE40005 (D5). Imputation performance on **MNAR:mid** type missing values.

	MAE		RMSE	
Method	M-value	B-value	M-value	B-value
mean	0.054±0.001	0.052±0.001*	0.088±0.001	0.080±0.001*
softImpute	0.063±0.002	0.052±0.008*	0.102±0.004	0.084±0.021*
impute.knn	**0.045±0.002***	0.052±0.006	**0.073±0.005***	0.093±0.014
imputePCA	0.051±0.001	0.048±0.001*	0.085±0.001	0.077±0.001*
SVDmiss	0.072±0.002	0.051±0.001*	0.113±0.003	0.082±0.002*
missForest	0.052±0.001	0.049±0.001*	0.084±0.001	0.075±0.001*
methyLImp	0.054±0.001	0.047±0.001*	0.085±0.001	0.075±0.001*

Table 44: Dataset GSE40005 (D5). Imputation performance on **MNAR:high** type missing values.

	MAE		RMSE	
Method	M-value	B-value	M-value	B-value
mean	0.024±0.001*	0.025±0.001	0.043±0.002	0.042±0.001*
softImpute	0.031±0.001	0.027±0.010*	0.058±0.005	0.048±0.032*
impute.knn	0.052±0.015*	0.105±0.027	0.112±0.031*	0.184±0.031
imputePCA	0.023±0.001*	0.023±0.001	0.041±0.002	0.040±0.001*
SVDmiss	0.037±0.001	0.027±0.001*	0.069±0.003	0.050±0.003*
missForest	0.023±0.001*	0.024±0.001	0.041±0.002	0.040±0.001*
methyLImp	0.027±0.001	**0.022±0.001***	0.044±0.002	**0.040±0.002***

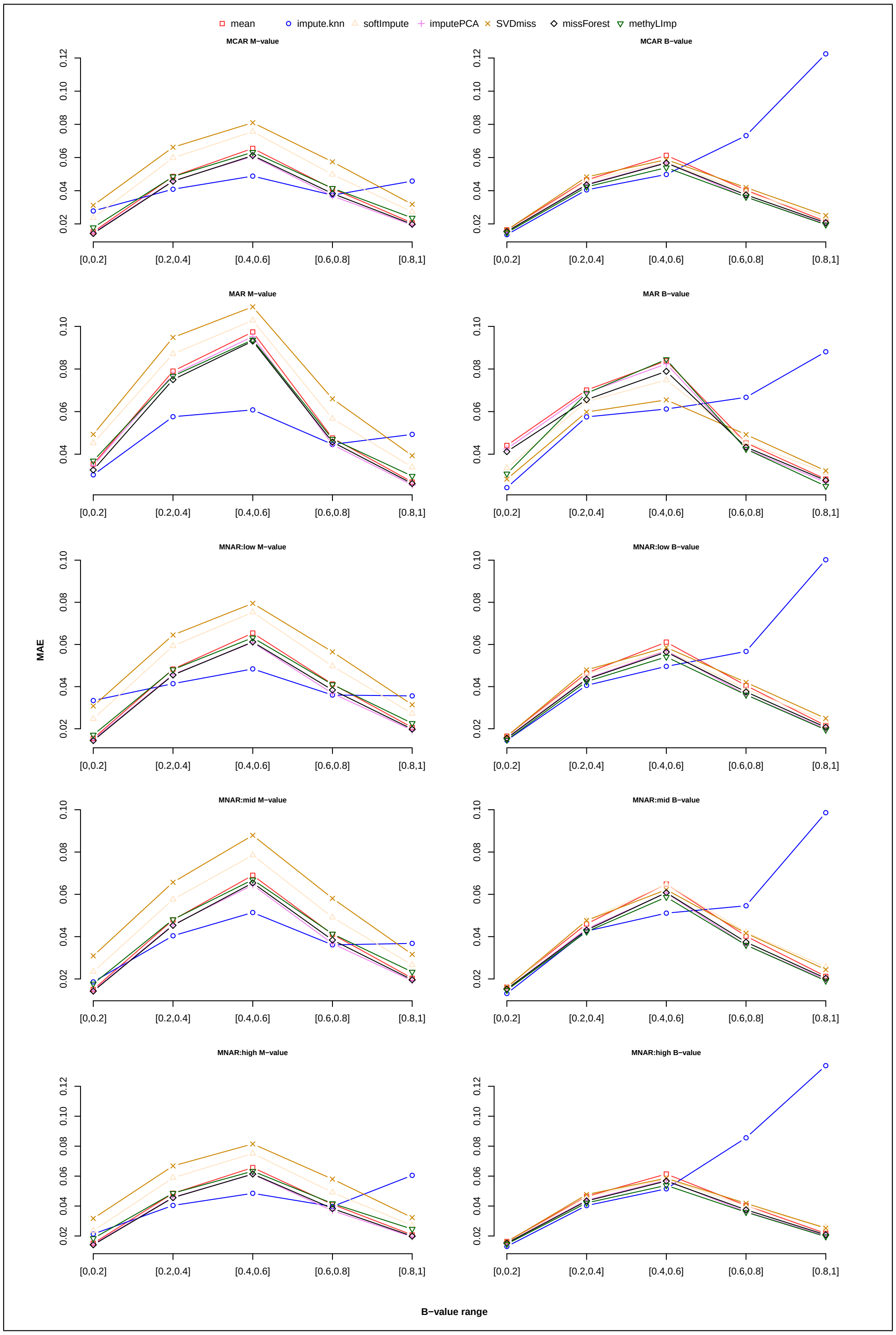

Figure 13: Dataset GSE40005 (D5). MAE imputation performances with respect to B-value range.

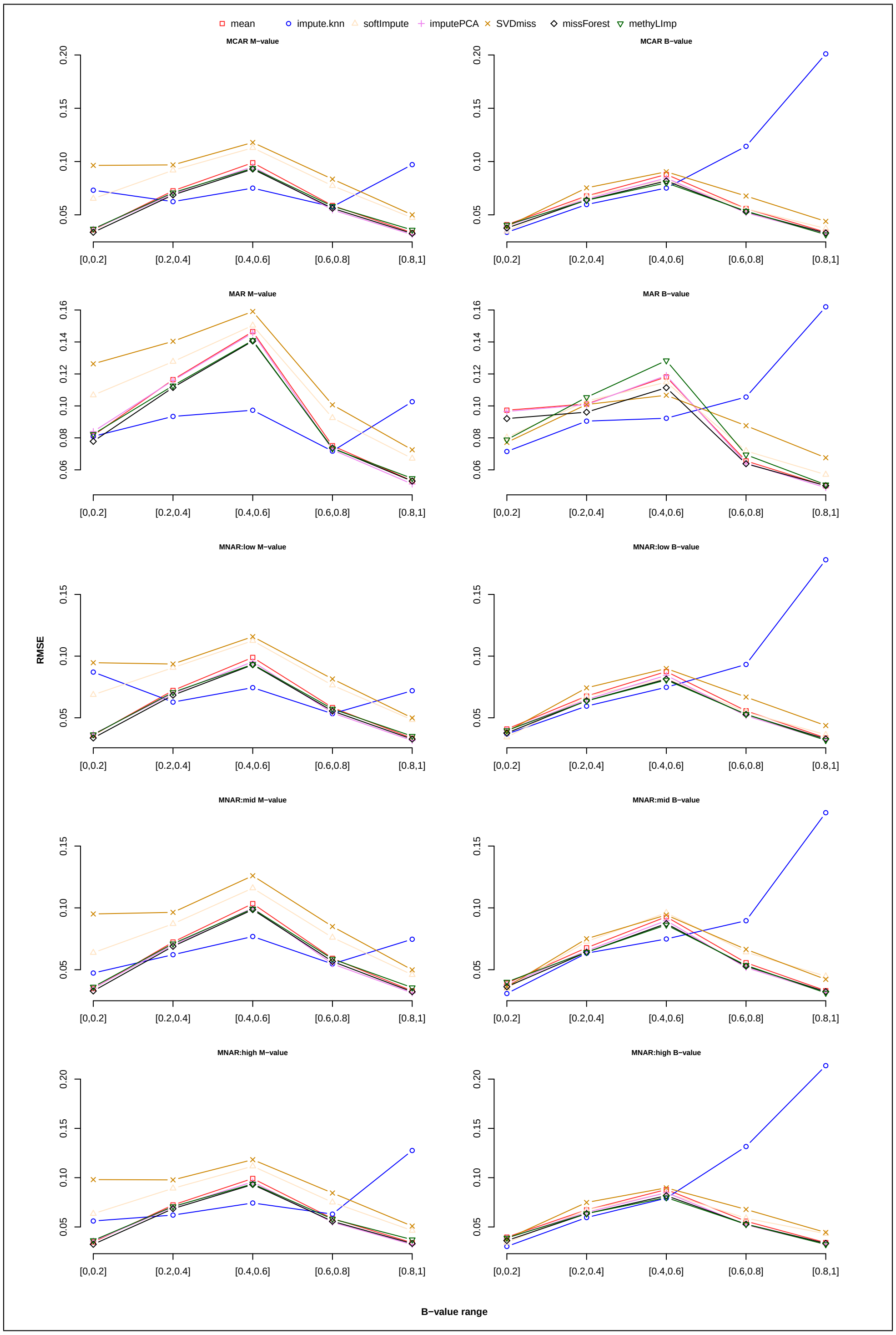

Figure 14: Dataset GSE40005 (D5). RMSE imputation performances with respect to B-value range.

2.6 GSE42921 (D6) - Colon mucosa - Crohn's disease - 5 samples

Method	Avg time (sec)	Avg RAM (Mb)
mean	< 1	4
softImpute	< 1	41
imputePCA	5	141
impute.knn	< 1	42
SVDmiss	10	3726
methyLImp	2	117
missForest	156	266

Table 45: Dataset GSE42921 (D6). Average time and memory usage.

Table 46: Dataset GSE42921 (D6). Imputation performance on **MCAR** type missing values.

	MAE		RMSE	
Method	M-value	B-value	M-value	B-value
mean	0.029±0.001	0.029±0.001*	0.053±0.002	0.051±0.002*
softImpute	0.032±0.001*	0.057±0.041	0.055±0.004*	0.118±0.099
impute.knn	0.080±0.023*	0.134±0.024	0.144±0.033*	0.214±0.024
imputePCA	0.028±0.001	0.028±0.001*	0.052±0.002	0.051±0.002*
SVDmiss	0.049±0.001	0.046±0.002*	0.082±0.003	0.083±0.006
missForest	0.029±0.001	0.029±0.001*	0.052±0.002	0.051±0.002*
methyLImp	0.028±0.001	**0.028±0.001***	0.049±0.002	**0.048±0.002***

Table 47: Dataset GSE42921 (D6). Imputation performance on **MAR** type missing values.

	MAE		RMSE	
Method	M-value	B-value	M-value	B-value
mean	0.049±0.001	0.048±0.001*	0.093±0.003	0.090±0.003*
softImpute	0.051±0.003*	0.084±0.041	0.094±0.006*	0.166±0.086
impute.knn	0.090±0.014*	0.149±0.025	0.153±0.019*	0.222±0.024
imputePCA	0.049±0.001	0.048±0.001*	0.093±0.003	0.089±0.003*
SVDmiss	0.065±0.002	0.059±0.002*	0.112±0.003	0.112±0.005
missForest	0.049±0.001	0.048±0.001*	0.093±0.003	0.089±0.003*
methyLImp	**0.045±0.001***	0.045±0.001	**0.085±0.003***	0.085±0.003

Table 48: Dataset GSE42921 (D6). Imputation performance on **MNAR:low** type missing values.

	MAE		RMSE	
Method	M-value	B-value	M-value	B-value
mean	0.021±0.001*	0.021±0.001	0.046±0.003*	0.048±0.003
softImpute	0.026±0.017	0.029±0.017	0.054±0.035	0.064±0.053
impute.knn	0.110±0.036	0.077±0.025*	0.186±0.039	0.159±0.033*
imputePCA	0.021±0.001*	0.021±0.001	0.045±0.003*	0.046±0.003
SVDmiss	0.037±0.001	0.033±0.001*	0.068±0.003	0.068±0.005
missForest	0.021±0.001*	0.021±0.001	0.046±0.003*	0.047±0.003
methyLImp	**0.020±0.001***	0.020±0.001	**0.041±0.003**	**0.041±0.003**

Table 49: Dataset GSE42921 (D6). Imputation performance on **MNAR:mid** type missing values.

	MAE		RMSE	
Method	M-value	B-value	M-value	B-value
mean	0.050±0.001	0.048±0.001*	0.081±0.002	0.075±0.002*
softImpute	0.051±0.001*	0.076±0.040	0.076±0.002*	0.129±0.082
impute.knn	0.051±0.005*	0.085±0.013	0.079±0.011*	0.148±0.022
imputePCA	0.050±0.001	0.048±0.001*	0.081±0.002	0.075±0.002*
SVDmiss	0.075±0.001	0.067±0.002*	0.109±0.002	0.108±0.004*
missForest	0.050±0.001	0.048±0.001*	0.081±0.002	0.075±0.002*
methyLImp	0.048±0.001	**0.047±0.001***	0.074±0.002	**0.073±0.002***

Table 50: Dataset GSE42921 (D6). Imputation performance on **MNAR:high** type missing values.

	MAE		RMSE	
Method	M-value	B-value	M-value	B-value
mean	0.024±0.001*	0.024±0.001	0.043±0.002	0.043±0.002*
softImpute	0.031±0.014*	0.079±0.066	0.057±0.033*	0.165±0.146
impute.knn	0.162±0.038*	0.224±0.041	0.245±0.037*	0.294±0.031
imputePCA	**0.023±0.001***	0.024±0.001	0.043±0.002	0.042±0.002*
SVDmiss	0.041±0.001	0.041±0.001	0.071±0.003*	0.076±0.005
missForest	0.024±0.001*	0.024±0.001	0.043±0.002	0.043±0.002*
methyLImp	0.024±0.001	0.024±0.001*	0.042±0.003	**0.042±0.003***

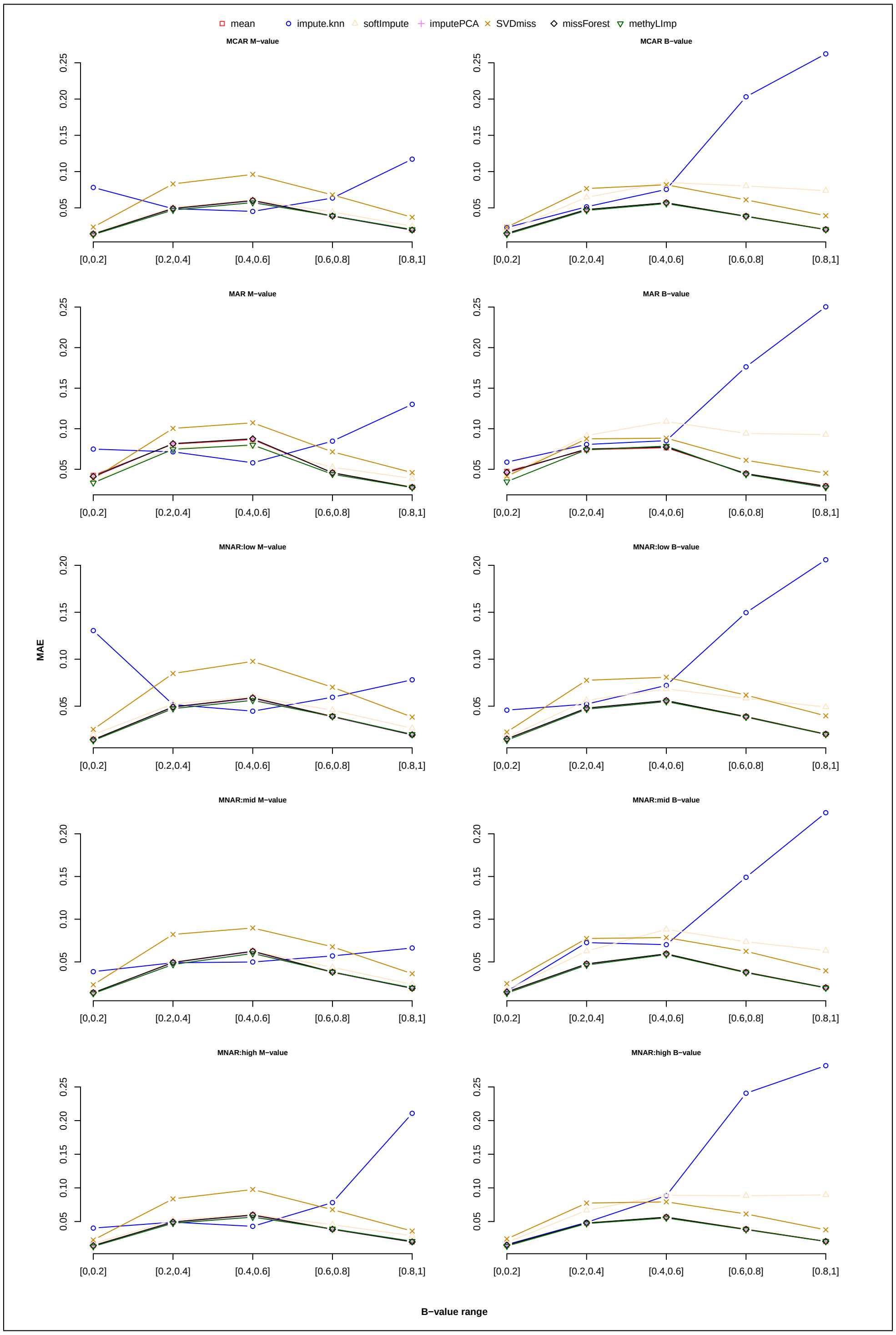

Figure 15: Dataset GSE42921 (D6). MAE imputation performances with respect to B-value range.

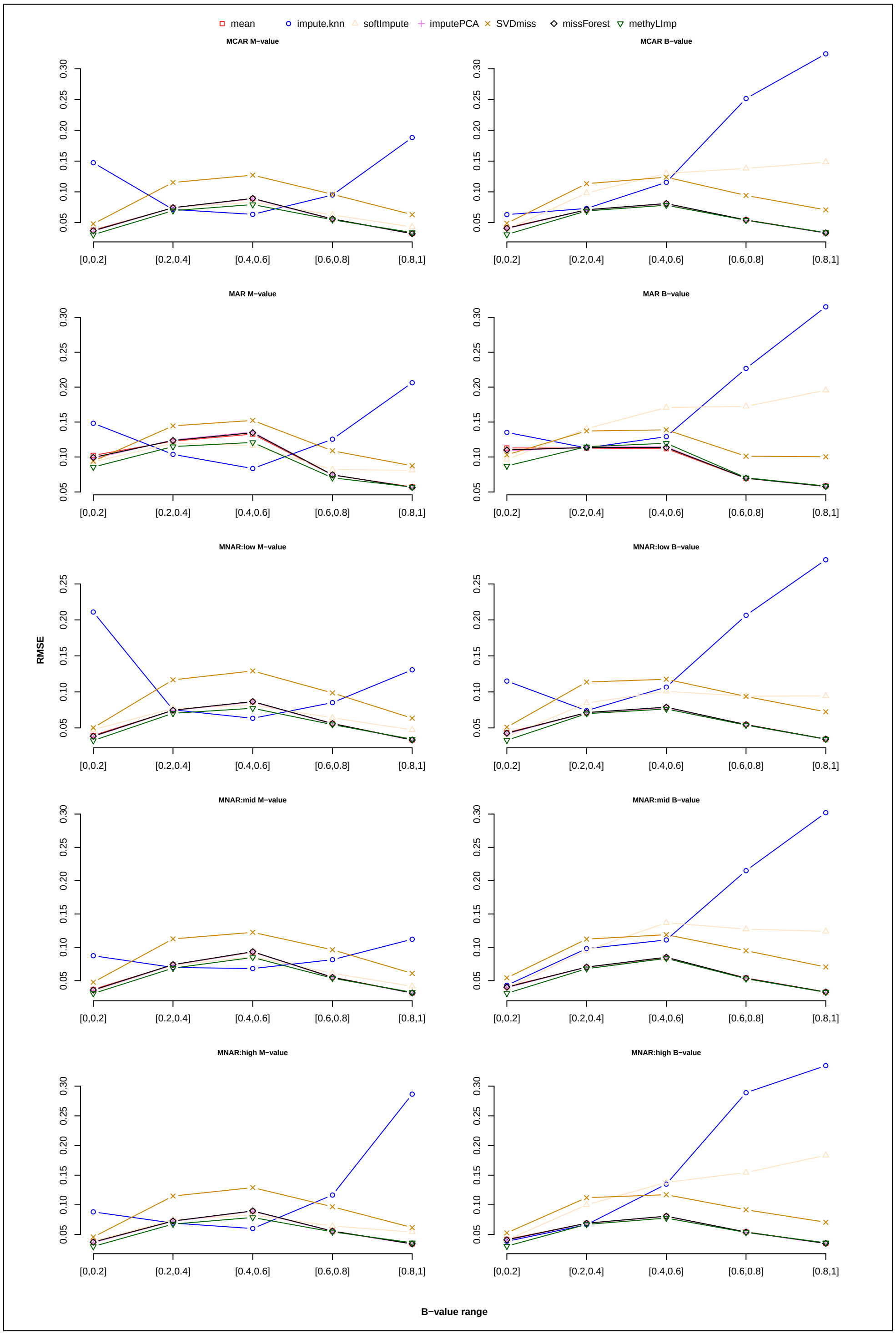

Figure 16: Dataset GSE42921 (D6). RMSE imputation performances with respect to B-value range.

2.7 GSE42921 (D7) - Colon mucosa - Ulcerative colitis - 6 samples

Method	Avg time (sec)	Avg RAM (Mb)
mean	< 1	5
softImpute	< 1	41
imputePCA	8	141
impute.knn	< 1	41
SVDmiss	11	3767
methyLImp	3	121
missForest	511	251

Table 51: Dataset GSE42921 (D7). Average time and memory usage.

Table 52: Dataset GSE42921 (D7). Imputation performance on **MCAR** type missing values.

	MAE		RMSE	
Method	M-value	B-value	M-value	B-value
mean	0.034±0.001	0.034±0.001*	0.055±0.002	0.055±0.002*
softImpute	0.030±0.001	0.039±0.025	0.050±0.002*	0.075±0.065
impute.knn	0.060±0.015*	0.102±0.023	0.118±0.026*	0.177±0.026
imputePCA	0.027±0.001	0.027±0.001*	0.047±0.002	0.046±0.002*
SVDmiss	0.049±0.001	0.036±0.001*	0.080±0.002	0.066±0.004*
missForest	0.031±0.001	0.031±0.001	0.051±0.002	0.050±0.002*
methyLImp	0.027±0.001	**0.026±0.001***	0.046±0.002	**0.045±0.002***

Table 53: Dataset GSE42921 (D7). Imputation performance on **MAR** type missing values.

	MAE		RMSE	
Method	M-value	B-value	M-value	B-value
mean	0.053±0.001*	0.053±0.001	0.090±0.002	0.089±0.002*
softImpute	0.040±0.001*	0.061±0.033	0.073±0.004*	0.121±0.078
impute.knn	0.069±0.010*	0.113±0.017	0.124±0.016*	0.185±0.019
imputePCA	0.042±0.001	0.042±0.001*	0.077±0.003	0.075±0.003*
SVDmiss	0.065±0.001	0.050±0.001*	0.106±0.003	0.100±0.005*
missForest	0.048±0.001	0.048±0.001	0.083±0.003	0.081±0.002*
methyLImp	**0.040±0.001***	0.041±0.001	**0.072±0.003***	0.076±0.003

Table 54: Dataset GSE42921 (D7). Imputation performance on **MNAR:low** type missing values.

	MAE		RMSE	
Method	M-value	B-value	M-value	B-value
mean	0.023±0.001*	0.024±0.001	0.045±0.002*	0.047±0.002
softImpute	0.022±0.001*	0.024±0.010	0.042±0.003*	0.050±0.032
impute.knn	0.077±0.026	0.055±0.016*	0.149±0.035	0.126±0.027*
imputePCA	0.020±0.001*	0.020±0.001	0.039±0.002*	0.040±0.002
SVDmiss	0.050±0.005	0.028±0.001*	0.085±0.007	0.057±0.004*
missForest	0.022±0.001*	0.022±0.001	0.042±0.002*	0.044±0.002
methyLImp	**0.020±0.001***	0.020±0.001	**0.037±0.002***	0.038±0.002

Table 55: Dataset GSE42921 (D7). Imputation performance on **MNAR:mid** type missing values.

	MAE		RMSE	
Method	M-value	B-value	M-value	B-value
mean	0.053±0.001	0.052±0.001*	0.081±0.002	0.076±0.002*
softImpute	0.049±0.001	0.052±0.024*	0.073±0.002	0.085±0.053
impute.knn	**0.041±0.003***	0.066±0.008	0.067±0.009*	0.121±0.015
imputePCA	0.044±0.001	0.042±0.001*	0.069±0.002	0.064±0.001*
SVDmiss	0.053±0.001	0.048±0.001*	0.082±0.002*	0.082±0.003
missForest	0.049±0.001	0.048±0.001*	0.075±0.002	0.070±0.001*
methyLImp	0.044±0.001	0.041±0.001*	0.068±0.002	**0.064±0.002***

Table 56: Dataset GSE42921 (D7). Imputation performance on **MNAR:high** type missing values.

	MAE		RMSE	
Method	M-value	B-value	M-value	B-value
mean	0.030±0.001*	0.030±0.001	0.047±0.002*	0.048±0.002
softImpute	0.025±0.001*	0.057±0.057	0.044±0.004*	0.117±0.133
impute.knn	0.135±0.034*	0.184±0.034	0.219±0.036*	0.261±0.028
imputePCA	0.024±0.001*	0.024±0.001	0.039±0.002*	0.040±0.002
SVDmiss	0.050±0.001	0.034±0.001*	0.080±0.002	0.063±0.005*
missForest	0.027±0.001*	0.028±0.001	0.043±0.002*	0.044±0.002
methyLImp	0.024±0.001	**0.023±0.001***	**0.039±0.002**	**0.039±0.002**

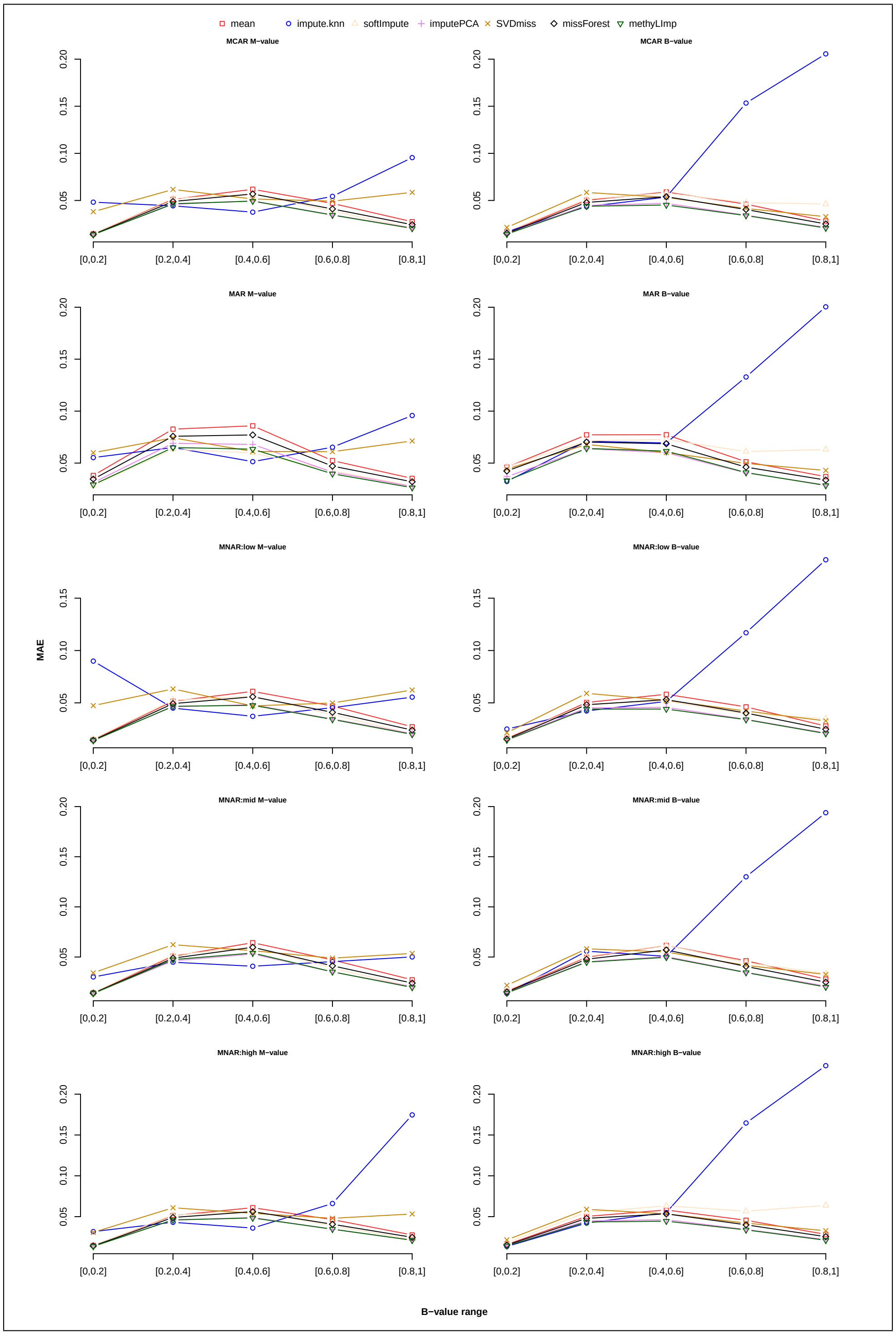

Figure 17: Dataset GSE42921 (D7). MAE imputation performances with respect to B-value range.

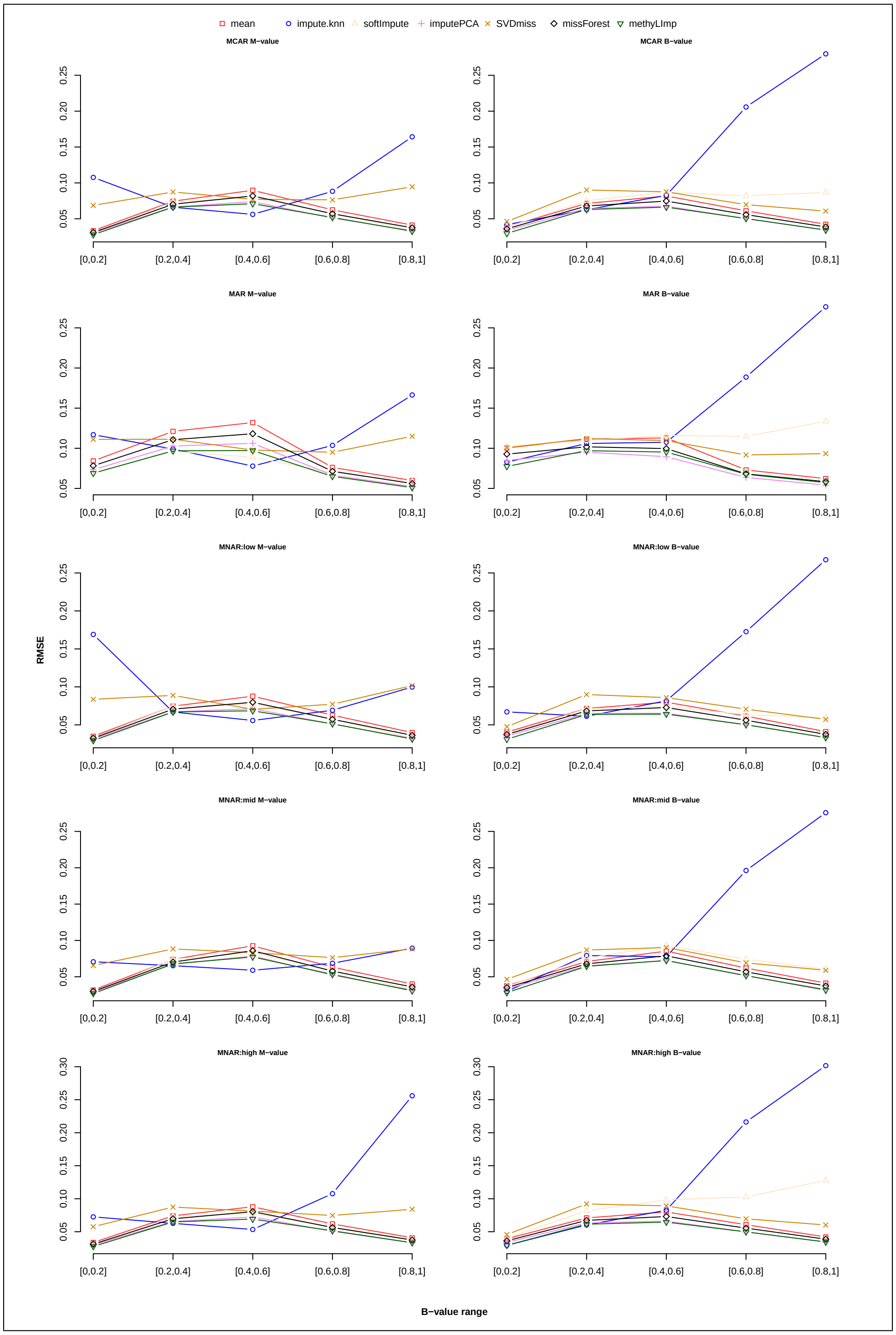

Figure 18: Dataset GSE42921 (D7). RMSE imputation performances with respect to B-value range.

2.8 GSE42921 (D8) - Colon - Normal - 12 samples

Method	Avg time (sec)	Avg RAM (Mb)
mean	< 1	10
softImpute	< 1	41
imputePCA	5	154
impute.knn	< 1	49
SVDmiss	25	4007
methyLImp	13	113
missForest	6647	101

Table 57: Dataset GSE42921 (D8). Average time and memory usage.

Table 58: Dataset GSE42921 (D8). Imputation performance on **MCAR** type missing values.

	MAE		RMSE	
Method	M-value	B-value	M-value	B-value
mean	0.022±0.001	0.022±0.001*	0.042±0.001	0.041±0.001*
softImpute	0.021±0.001*	0.021±0.001	0.037±0.002	0.036±0.002*
impute.knn	0.037±0.009*	0.056±0.013	0.088±0.021*	0.121±0.022
imputePCA	0.021±0.001	0.021±0.001*	0.041±0.001	0.040±0.001*
SVDmiss	0.021±0.001*	0.022±0.001	0.039±0.002*	0.040±0.002
missForest	0.021±0.001	0.021±0.001*	0.039±0.001	0.039±0.001*
methyLImp	0.020±0.001	**0.020±0.001***	**0.035±0.002**	**0.035±0.002**

Table 59: Dataset GSE42921 (D8). Imputation performance on **MAR** type missing values.

	MAE		RMSE	
Method	M-value	B-value	M-value	B-value
mean	0.041±0.001	0.039±0.001*	0.079±0.002	0.076±0.002*
softImpute	**0.029±0.001***	0.032±0.009	**0.059±0.002**	**0.063±0.025**
impute.knn	0.040±0.005*	0.054±0.008	0.084±0.013*	0.110±0.014
imputePCA	0.040±0.001	0.039±0.001*	0.078±0.002	0.075±0.002*
SVDmiss	0.032±0.001*	0.033±0.001	0.068±0.003*	0.070±0.003
missForest	0.039±0.001	0.038±0.001*	0.075±0.002	0.072±0.002*
methyLImp	0.034±0.001	0.033±0.001*	0.065±0.002	0.064±0.002*

Table 60: Dataset GSE42921 (D8). Imputation performance on **MNAR:low** type missing values.

	MAE		RMSE	
Method	M-value	B-value	M-value	B-value
mean	0.017±0.001*	0.017±0.001	0.038±0.002*	0.040±0.002
softImpute	0.016±0.001*	0.016±0.001	0.031±0.002*	0.031±0.002
impute.knn	0.039±0.014	0.030±0.006*	0.099±0.028	0.082±0.016*
imputePCA	0.017±0.001*	0.017±0.001	0.037±0.002*	0.038±0.002
SVDmiss	0.016±0.001*	0.017±0.001	0.033±0.002*	0.034±0.003
missForest	0.016±0.001*	0.017±0.001	0.035±0.002*	0.036±0.002
methyLImp	**0.015±0.001***	0.015±0.001	**0.030±0.002**	**0.030±0.002**

Table 61: Dataset GSE42921 (D8). Imputation performance on **MNAR:mid** type missing values.

	MAE		RMSE	
Method	M-value	B-value	M-value	B-value
mean	0.038±0.001	0.036±0.001*	0.065±0.001	0.057±0.001*
softImpute	0.033±0.001	0.033±0.001*	0.051±0.001	0.050±0.002*
impute.knn	0.032±0.002*	0.039±0.004	0.057±0.008*	0.076±0.012
imputePCA	0.036±0.001	0.034±0.001*	0.063±0.002	0.055±0.001*
SVDmiss	0.033±0.001	0.032±0.001*	0.054±0.001	0.055±0.002
missForest	0.036±0.001	0.034±0.001*	0.060±0.001	0.053±0.001*
methyLImp	0.032±0.001	**0.031±0.001***	0.051±0.001	**0.050±0.001***

Table 62: Dataset GSE42921 (D8). Imputation performance on **MNAR:high** type missing values.

	MAE		RMSE	
Method	M-value	B-value	M-value	B-value
mean	0.018±0.001*	0.019±0.001	0.034±0.001	0.034±0.001
softImpute	0.018±0.001*	0.019±0.007	0.032±0.002*	0.035±0.023
impute.knn	0.052±0.018*	0.091±0.024	0.121±0.035*	0.172±0.028
imputePCA	0.018±0.001*	0.018±0.001	0.034±0.002	0.034±0.001
SVDmiss	0.019±0.001*	0.020±0.001	0.036±0.002*	0.037±0.003
missForest	0.018±0.001*	0.018±0.001	0.033±0.001*	0.033±0.001
methyLImp	**0.017±0.001***	0.017±0.001	**0.031±0.002***	0.031±0.002

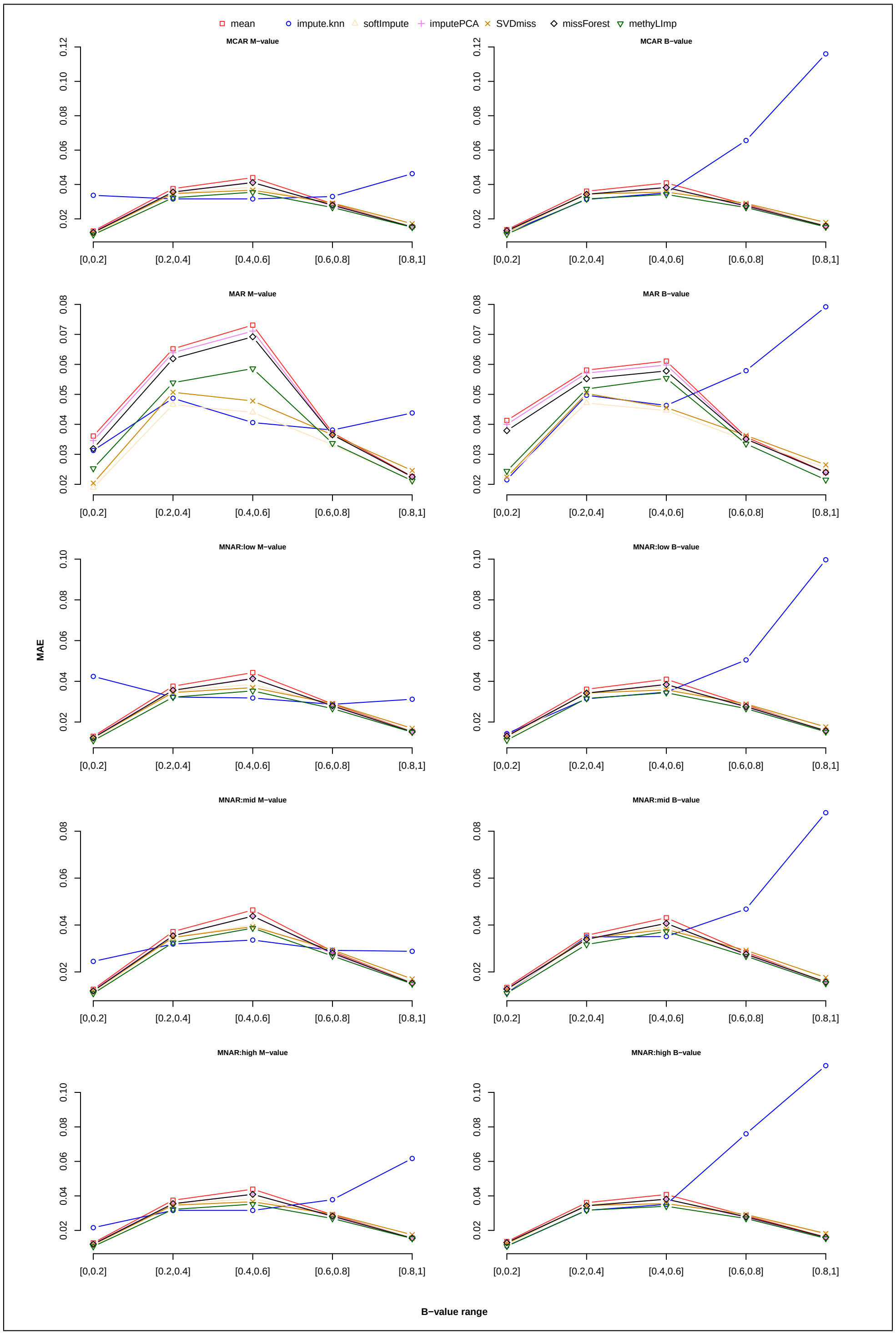

Figure 19: Dataset GSE42921 (D8). MAE imputation performances with respect to B-value range.

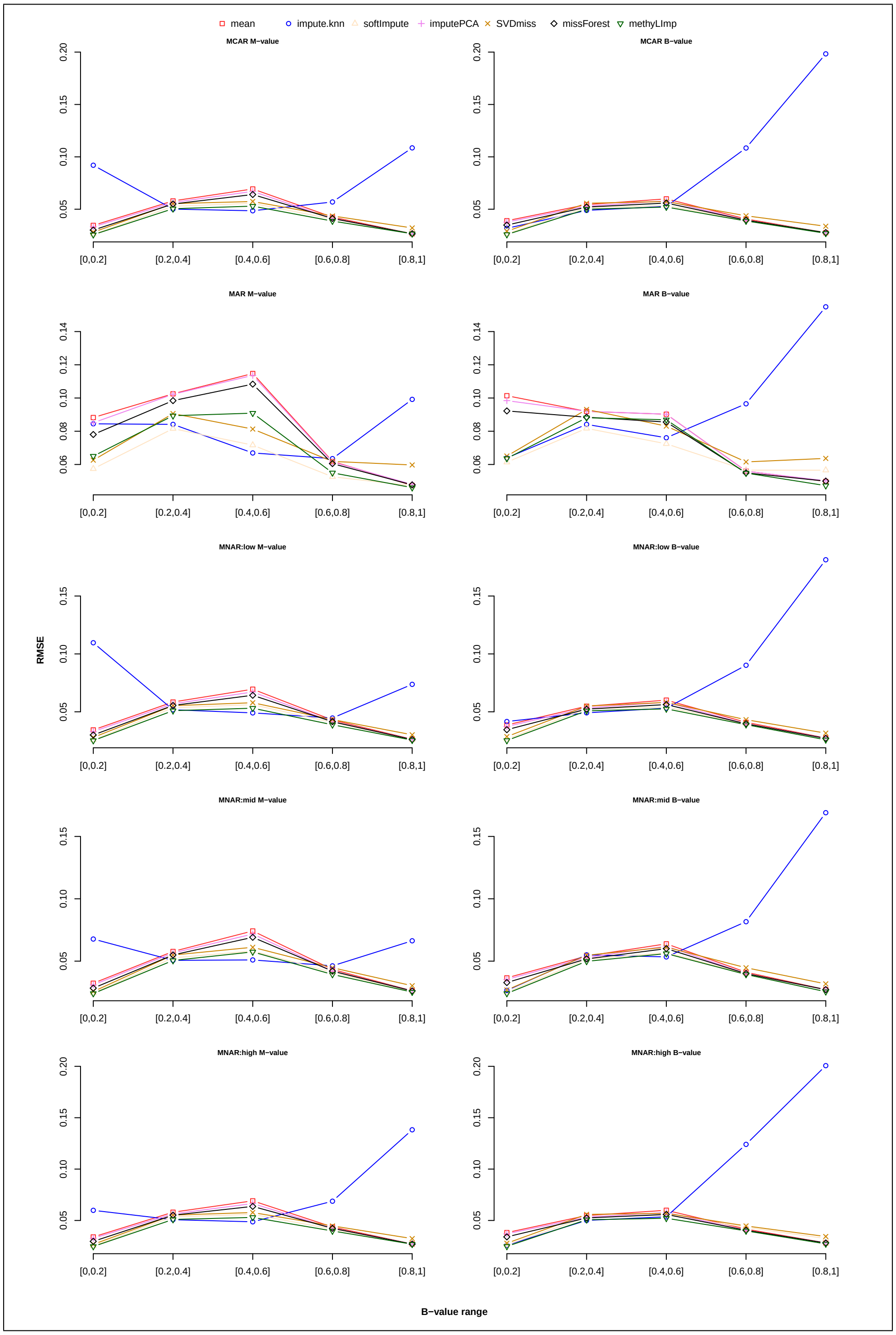

Figure 20: Dataset GSE42921 (D8). RMSE imputation performances with respect to B-value range.

2.9 GSE43091 (D9) - Liver - Cancer - 50 samples

Method	Avg time (sec)	Avg RAM (Mb)
mean	< 1	42
softImpute	1	106
imputePCA	16	257
impute.knn	2	113
SVDmiss	194	4965
methyLImp	1364	129
missForest	104702	300

Table 63: Dataset GSE43091 (D9). Average time and memory usage.

Table 64: Dataset GSE43091 (D9). Imputation performance on **MCAR** type missing values.

	MAE		RMSE	
Method	M-value	B-value	M-value	B-value
mean	0.050±0.001	0.050±0.001*	0.085±0.001	0.082±0.001*
softImpute	0.053±0.001	0.046±0.001*	0.086±0.001	0.076±0.001*
impute.knn	**0.043±0.001***	0.046±0.002	0.075±0.001*	0.081±0.005
imputePCA	0.046±0.001	0.046±0.001*	0.081±0.001	0.076±0.001*
SVDmiss	0.058±0.001	0.045±0.001*	0.100±0.002	0.074±0.001*
missForest	0.044±0.001*	0.044±0.001	0.076±0.001	**0.073±0.001***
methyLImp	0.058±0.001	0.044±0.001*	0.100±0.002	0.079±0.001*

Table 65: Dataset GSE43091 (D9). Imputation performance on **MAR** type missing values.

	MAE		RMSE	
Method	M-value	B-value	M-value	B-value
mean	0.066±0.001	0.063±0.001*	0.110±0.001	0.099±0.001*
softImpute	0.068±0.001	0.059±0.001*	0.111±0.001	0.094±0.001*
impute.knn	0.055±0.001*	0.056±0.002	0.097±0.001	0.094±0.005*
imputePCA	0.062±0.001	0.058±0.001*	0.108±0.001	0.095±0.001*
SVDmiss	0.073±0.001	0.055±0.001*	0.123±0.001	0.090±0.001*
missForest	0.056±0.001	**0.054±0.001***	0.099±0.002	**0.086±0.001***
methyLImp	0.066±0.001	0.058±0.001*	0.112±0.001	0.104±0.001*

Table 66: Dataset GSE43091 (D9). Imputation performance on **MNAR:low** type missing values.

	MAE		RMSE	
Method	M-value	B-value	M-value	B-value
mean	0.035±0.001*	0.037±0.001	0.067±0.001*	0.068±0.001
softImpute	0.040±0.001	0.034±0.001*	0.071±0.001	0.064±0.001*
impute.knn	**0.030±0.001***	0.033±0.001	**0.059±0.001***	0.065±0.003
imputePCA	0.034±0.001*	0.034±0.001	0.069±0.001	0.065±0.001*
SVDmiss	0.044±0.001	0.033±0.001*	0.090±0.003	0.062±0.001*
missForest	0.031±0.001*	0.033±0.001	0.060±0.001*	0.060±0.001
methyLImp	0.043±0.002	0.033±0.001*	0.095±0.005	0.069±0.002*

Table 67: Dataset GSE43091 (D9). Imputation performance on **MNAR:mid** type missing values.

	MAE		RMSE	
Method	M-value	B-value	M-value	B-value
mean	0.083±0.001	0.078±0.001*	0.121±0.001	0.110±0.001*
softImpute	0.084±0.001	0.070±0.001*	0.120±0.001	0.100±0.001*
impute.knn	0.069±0.001	**0.066±0.001***	0.103±0.001	0.098±0.002*
imputePCA	0.076±0.001	0.071±0.001*	0.112±0.001	0.101±0.001*
SVDmiss	0.089±0.001	0.068±0.001*	0.129±0.001	0.099±0.001*
missForest	0.071±0.001	**0.066±0.001***	0.107±0.001	**0.096±0.001***
methyLImp	0.086±0.001	0.070±0.001*	0.123±0.001	0.106±0.001*

Table 68: Dataset GSE43091 (D9). Imputation performance on **MNAR:high** type missing values.

	MAE		RMSE	
Method	M-value	B-value	M-value	B-value
mean	0.042±0.001*	0.044±0.001	0.072±0.001	0.071±0.001*
softImpute	0.045±0.001	0.041±0.001*	0.074±0.001	0.066±0.001*
impute.knn	**0.036±0.001***	0.044±0.004	0.065±0.002*	0.081±0.010
imputePCA	0.038±0.001*	0.040±0.001	0.068±0.001	0.066±0.001*
SVDmiss	0.049±0.001	0.040±0.001*	0.088±0.002	0.065±0.001*
missForest	0.037±0.001*	0.039±0.001	0.065±0.001	**0.064±0.001***
methyLImp	0.045±0.001	0.037±0.001*	0.076±0.001	0.066±0.001*

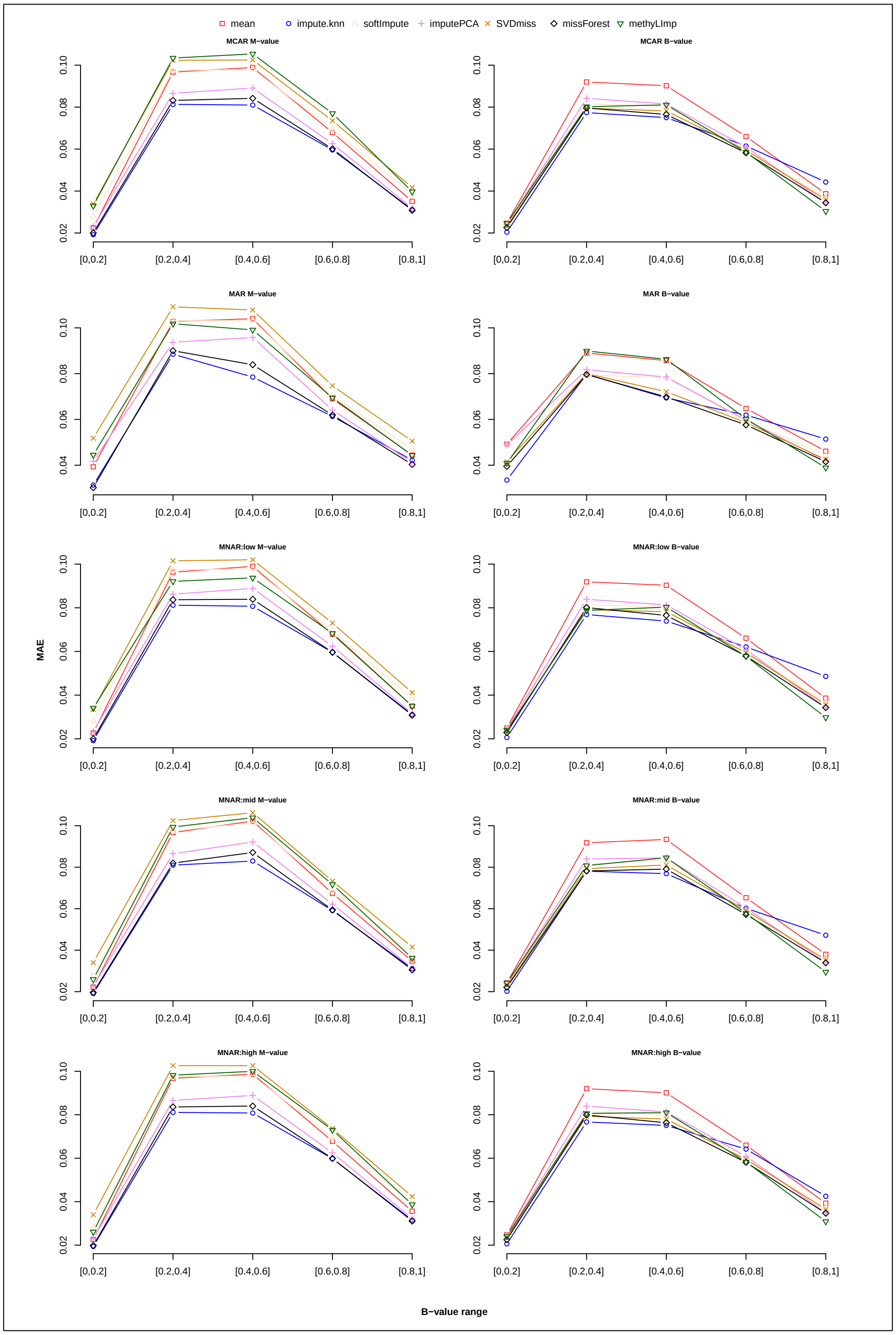

Figure 21: Dataset GSE43091 (D9). MAE imputation performances with respect to B-value range.

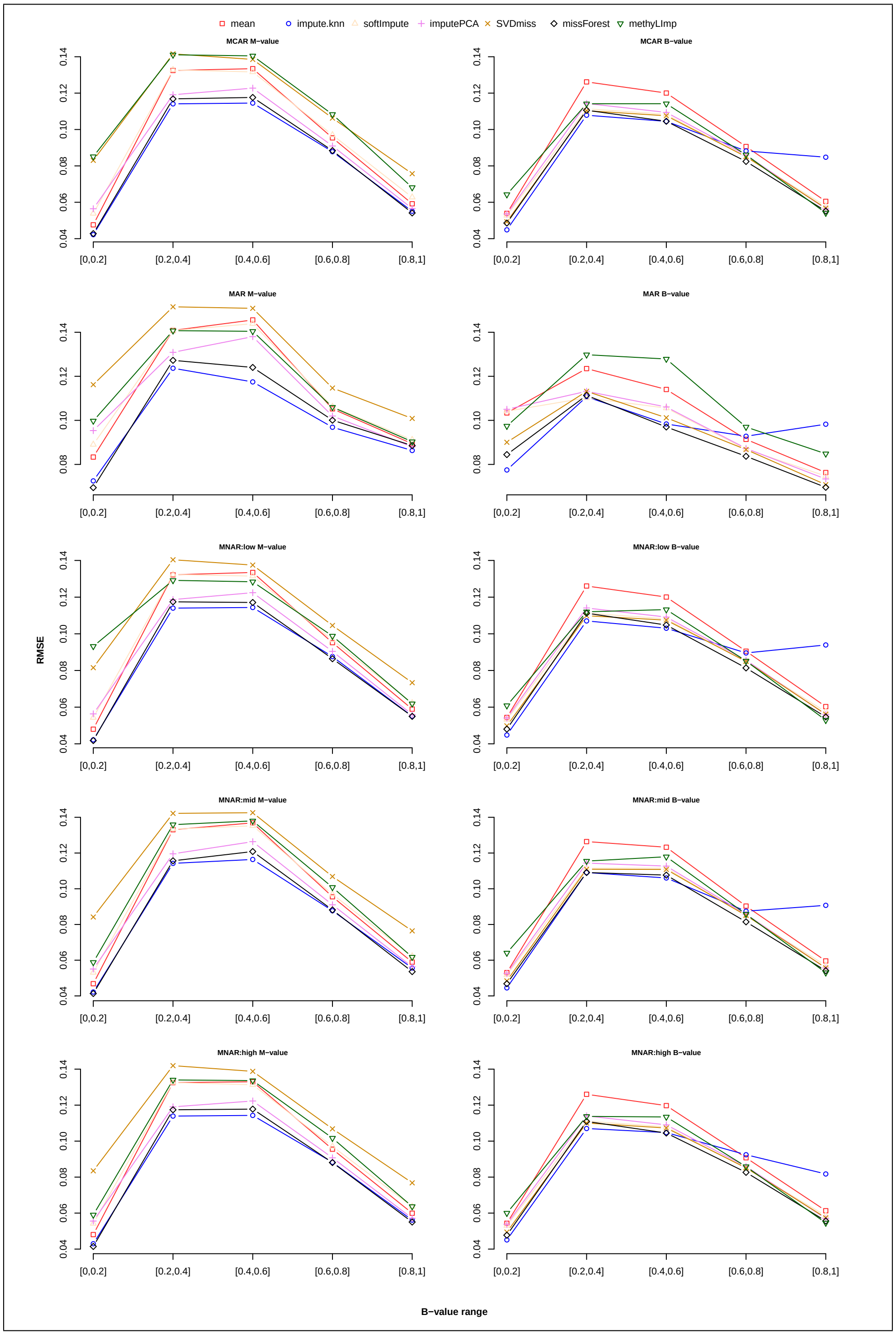

Figure 22: Dataset GSE43091 (D9). RMSE imputation performances with respect to B-value range.

2.10 GSE43091 (D10) - Liver - Normal - 4 samples

Method	Avg time (sec)	Avg RAM (Mb)
mean	< 1	4
softImpute	< 1	42
imputePCA	5	142
impute.knn	< 1	42
SVDmiss	10	3500
methyLImp	1	115
missForest	103	260

Table 69: Dataset GSE43091 (D10). Average time and memory usage.

Table 70: Dataset GSE43091 (D10). Imputation performance on **MCAR** type missing values.

	MAE		RMSE	
Method	M-value	B-value	M-value	B-value
mean	0.027±0.001	0.027±0.001*	0.044±0.002	0.043±0.002*
softImpute	0.069±0.023*	0.119±0.051	0.130±0.047*	0.244±0.116
impute.knn	0.088±0.023*	0.153±0.037	0.156±0.031*	0.227±0.040
imputePCA	0.027±0.001	0.027±0.001*	0.044±0.002	0.043±0.002*
SVDmiss	0.050±0.001	**0.027±0.001***	0.064±0.002	**0.042±0.002***
missForest	0.027±0.001	0.027±0.001*	0.044±0.002	0.043±0.002*
methyLImp	0.043±0.001	0.027±0.001*	0.057±0.002	0.044±0.002*

Table 71: Dataset GSE43091 (D10). Imputation performance on **MAR** type missing values.

	MAE		RMSE	
Method	M-value	B-value	M-value	B-value
mean	0.036±0.001	0.036±0.001*	0.067±0.004	0.064±0.003*
softImpute	0.086±0.023*	0.136±0.046	0.159±0.042*	0.265±0.091
impute.knn	0.088±0.019*	0.159±0.026	0.151±0.027*	0.228±0.026
imputePCA	0.035±0.001	**0.035±0.001***	0.065±0.004	**0.062±0.003***
SVDmiss	0.055±0.001	0.035±0.001*	0.080±0.003	0.063±0.003*
missForest	0.035±0.001	0.035±0.001*	0.065±0.004	0.062±0.003*
methyLImp	0.049±0.001	0.035±0.001*	0.073±0.003	0.065±0.004*

Table 72: Dataset GSE43091 (D10). Imputation performance on **MNAR:low** type missing values.

	MAE		RMSE	
Method	M-value	B-value	M-value	B-value
mean	0.021±0.001	0.021±0.001	0.037±0.003	0.037±0.003
softImpute	0.070±0.025	0.068±0.026	0.150±0.050	0.167±0.075
impute.knn	0.110±0.042	0.092±0.023*	0.180±0.047	0.177±0.030
imputePCA	0.020±0.001*	0.020±0.001	0.037±0.003	0.037±0.003
SVDmiss	0.053±0.003	**0.020±0.001***	0.068±0.003	**0.036±0.003***
missForest	0.021±0.001	0.021±0.001	0.037±0.003	0.037±0.003
methyLImp	0.040±0.001	0.021±0.001*	0.054±0.002	0.037±0.003*

Table 73: Dataset GSE43091 (D10). Imputation performance on **MNAR:mid** type missing values.

	MAE		RMSE	
Method	M-value	B-value	M-value	B-value
mean	0.042±0.001	0.041±0.001*	0.063±0.002	0.061±0.002*
softImpute	0.066±0.011*	0.133±0.038	0.107±0.025*	0.234±0.072
impute.knn	0.052±0.005*	0.084±0.013	0.081±0.012*	0.145±0.021
imputePCA	0.041±0.001	0.040±0.001*	0.062±0.002	0.060±0.002*
SVDmiss	0.051±0.001	**0.040±0.001***	0.071±0.002	**0.060±0.002***
missForest	0.042±0.001	0.041±0.001*	0.063±0.002	0.061±0.002*
methyLImp	0.043±0.001	0.041±0.001*	0.061±0.002*	0.063±0.002

Table 74: Dataset GSE43091 (D10). Imputation performance on **MNAR:high** type missing values.

	MAE		RMSE	
Method	M-value	B-value	M-value	B-value
mean	0.025±0.001*	0.025±0.001	0.039±0.003	0.039±0.002*
softImpute	0.071±0.029*	0.153±0.062	0.130±0.051*	0.303±0.125
impute.knn	0.192±0.040*	0.238±0.055	0.275±0.039*	0.298±0.048
imputePCA	0.024±0.001*	0.024±0.001	0.039±0.003	0.038±0.002*
SVDmiss	0.047±0.001	0.025±0.001*	0.059±0.002	**0.038±0.002***
missForest	0.025±0.001*	0.025±0.001	0.039±0.003	0.039±0.002*
methyLImp	0.045±0.001	**0.024±0.001***	0.058±0.002	0.039±0.003*

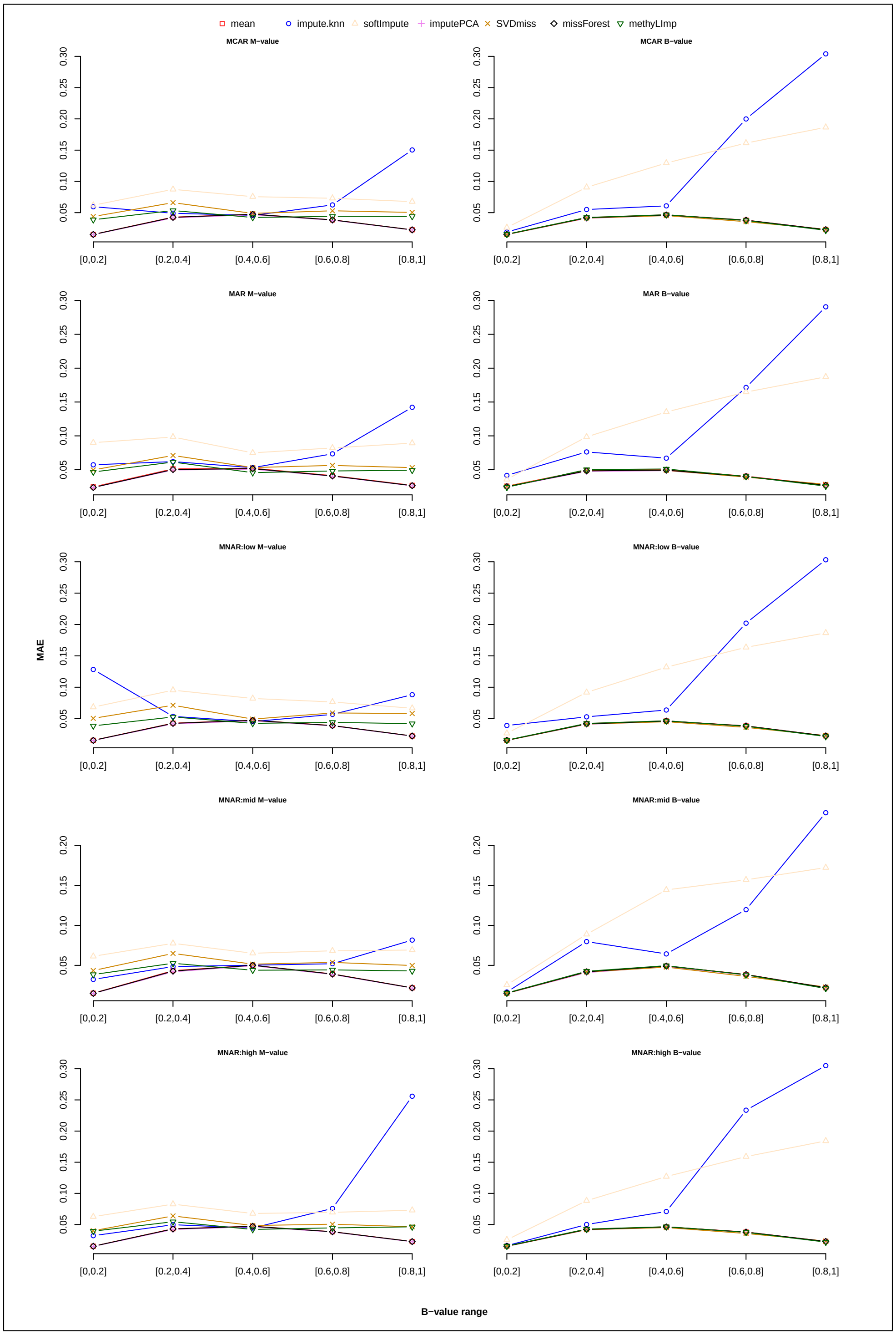

Figure 23: Dataset GSE43091 (D10). MAE imputation performances with respect to B-value range.

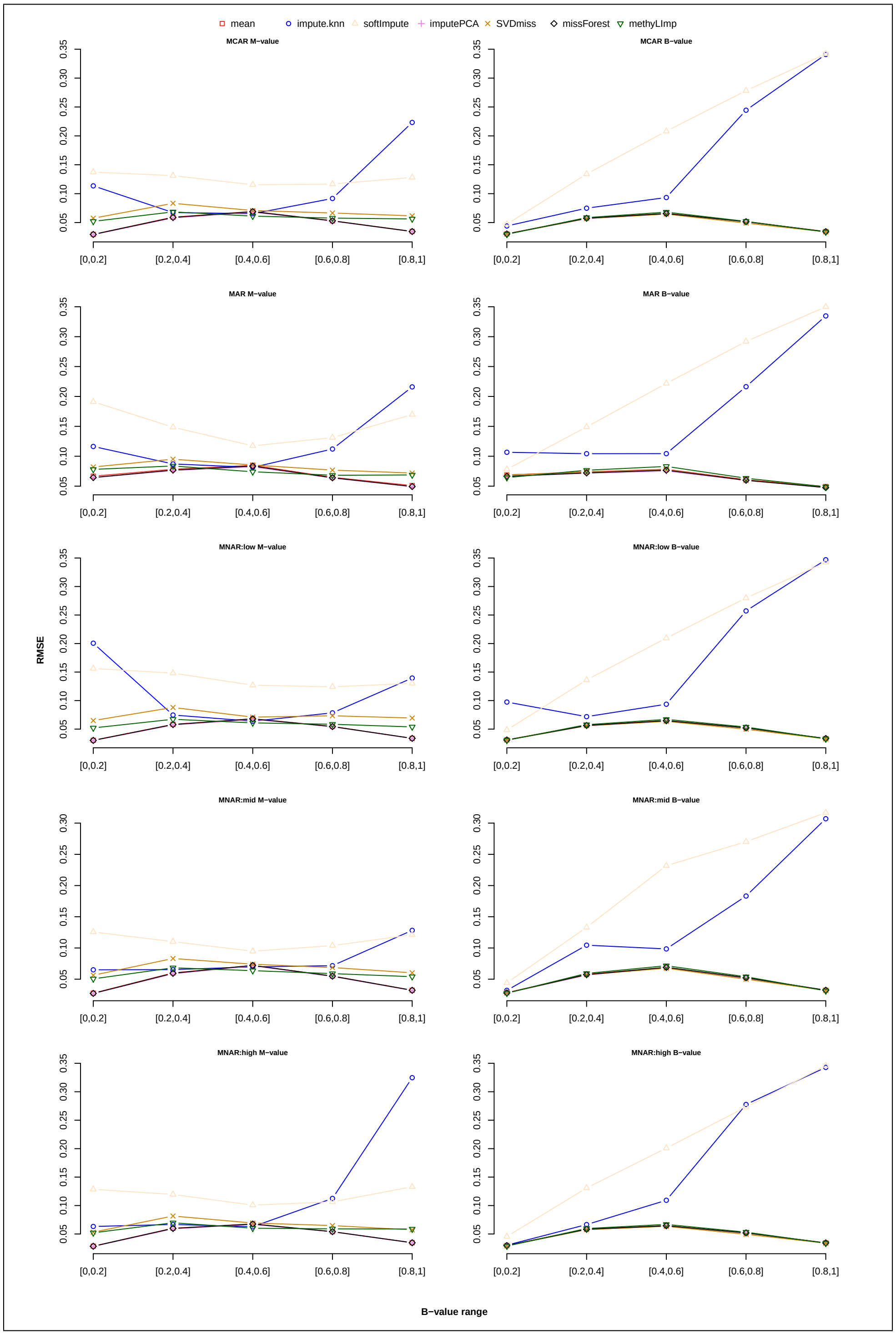

Figure 24: Dataset GSE43091 (D10). RMSE imputation performances with respect to B-value range.

2.11 GSE44684 (D11) - Cerebellum - Normal - 6 samples

Method	Avg time (sec)	Avg RAM (Mb)
mean	< 1	5
softImpute	< 1	41
imputePCA	7	142
impute.knn	< 1	41
SVDmiss	11	3767
methyLImp	2	118
missForest	474	253

Table 75: Dataset GSE44684 (D11). Average time and memory usage.

Table 76: Dataset GSE44684 (D11). Imputation performance on **MCAR** type missing values.

	MAE		RMSE	
Method	M-value	B-value	M-value	B-value
mean	0.048±0.001	0.048±0.001	0.085±0.002	0.084±0.002*
softImpute	0.030±0.001*	0.032±0.002	0.058±0.003*	0.066±0.010
impute.knn	0.071±0.016*	0.099±0.021	0.134±0.024*	0.172±0.026
imputePCA	0.037±0.001*	0.037±0.001	0.065±0.002	0.065±0.002
SVDmiss	0.033±0.001*	0.036±0.001	0.057±0.003*	0.062±0.004
missForest	0.041±0.001*	0.041±0.001	0.072±0.002	0.071±0.002*
methyLImp	**0.027±0.001***	0.029±0.001	**0.049±0.003***	0.050±0.003

Table 77: Dataset GSE44684 (D11). Imputation performance on **MAR** type missing values.

	MAE		RMSE	
Method	M-value	B-value	M-value	B-value
mean	0.062±0.001	0.062±0.001	0.105±0.002	0.103±0.002*
softImpute	0.057±0.003*	0.064±0.005	0.109±0.006*	0.128±0.016
impute.knn	0.073±0.008*	0.101±0.019	0.129±0.014*	0.167±0.025
imputePCA	0.056±0.001*	0.056±0.001	0.098±0.002	0.097±0.002*
SVDmiss	0.051±0.001*	0.054±0.001	0.093±0.004*	0.101±0.005
missForest	0.058±0.001*	0.058±0.001	0.099±0.002	0.098±0.002*
methyLImp	**0.046±0.001***	0.050±0.001	**0.088±0.003***	0.091±0.003

Table 78: Dataset GSE44684 (D11). Imputation performance on **MNAR:low** type missing values.

	MAE		RMSE	
Method	M-value	B-value	M-value	B-value
mean	0.036±0.001*	0.037±0.001	0.071±0.003*	0.073±0.002
softImpute	0.025±0.002*	0.026±0.001	0.055±0.008	0.054±0.005
impute.knn	0.095±0.030	0.058±0.017*	0.170±0.035	0.124±0.027*
imputePCA	0.028±0.001*	0.029±0.001	0.054±0.002*	0.057±0.002
SVDmiss	0.026±0.001*	0.033±0.001	0.049±0.003*	0.055±0.004
missForest	0.031±0.001*	0.032±0.001	0.060±0.002*	0.062±0.002
methyLImp	**0.021±0.001***	0.024±0.001	**0.041±0.003***	0.042±0.003

Table 79: Dataset GSE44684 (D11). Imputation performance on **MNAR:mid** type missing values.

	MAE		RMSE	
Method	M-value	B-value	M-value	B-value
mean	0.090±0.001	0.085±0.001*	0.130±0.002	0.121±0.002*
softImpute	0.056±0.002*	0.063±0.004	0.095±0.003*	0.112±0.012
impute.knn	0.054±0.003*	0.073±0.007	**0.086±0.007***	0.119±0.012
imputePCA	0.071±0.001	0.068±0.001*	0.108±0.002	0.101±0.002*
SVDmiss	0.056±0.001	0.056±0.001*	0.089±0.003*	0.093±0.004
missForest	0.078±0.001	0.074±0.001*	0.116±0.002	0.107±0.002*
methyLImp	**0.053±0.001***	0.053±0.001	**0.086±0.002***	0.086±0.003

Table 80: Dataset GSE44684 (D11). Imputation performance on **MNAR:high** type missing values.

	MAE		RMSE	
Method	M-value	B-value	M-value	B-value
mean	0.040±0.001*	0.041±0.001	0.073±0.002*	0.074±0.002
softImpute	0.027±0.002*	0.031±0.004	0.054±0.006*	0.069±0.022
impute.knn	0.121±0.031*	0.196±0.038	0.205±0.035*	0.271±0.031
imputePCA	0.031±0.001*	0.032±0.001	0.054±0.002*	0.056±0.002
SVDmiss	0.030±0.001*	0.032±0.001	0.053±0.003*	0.059±0.004
missForest	0.034±0.001*	0.035±0.001	0.061±0.002*	0.062±0.002
methyLImp	**0.024±0.001***	0.027±0.001	**0.043±0.003***	0.045±0.002

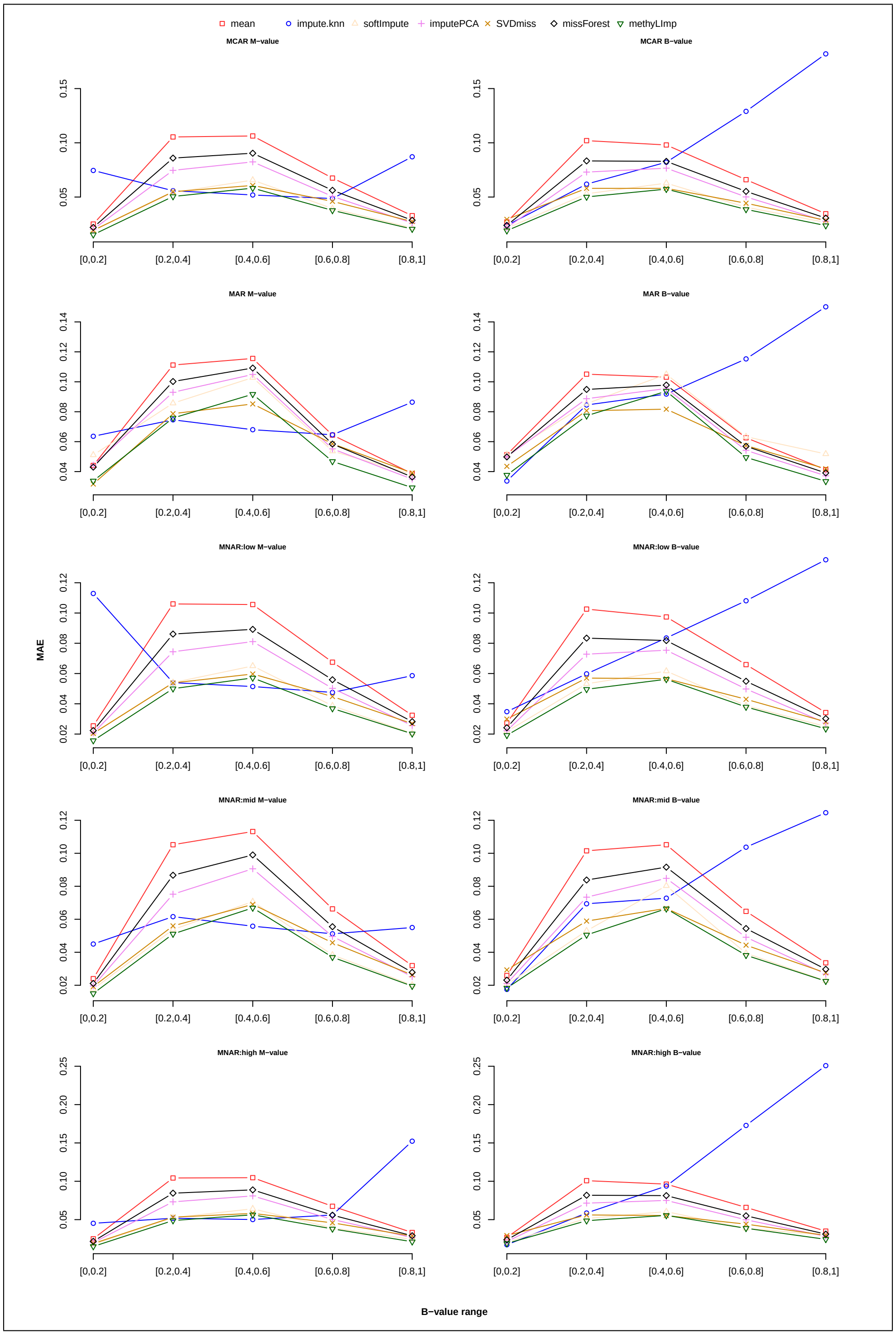

Figure 25: Dataset GSE44684 (D11). MAE imputation performances with respect to B-value range.

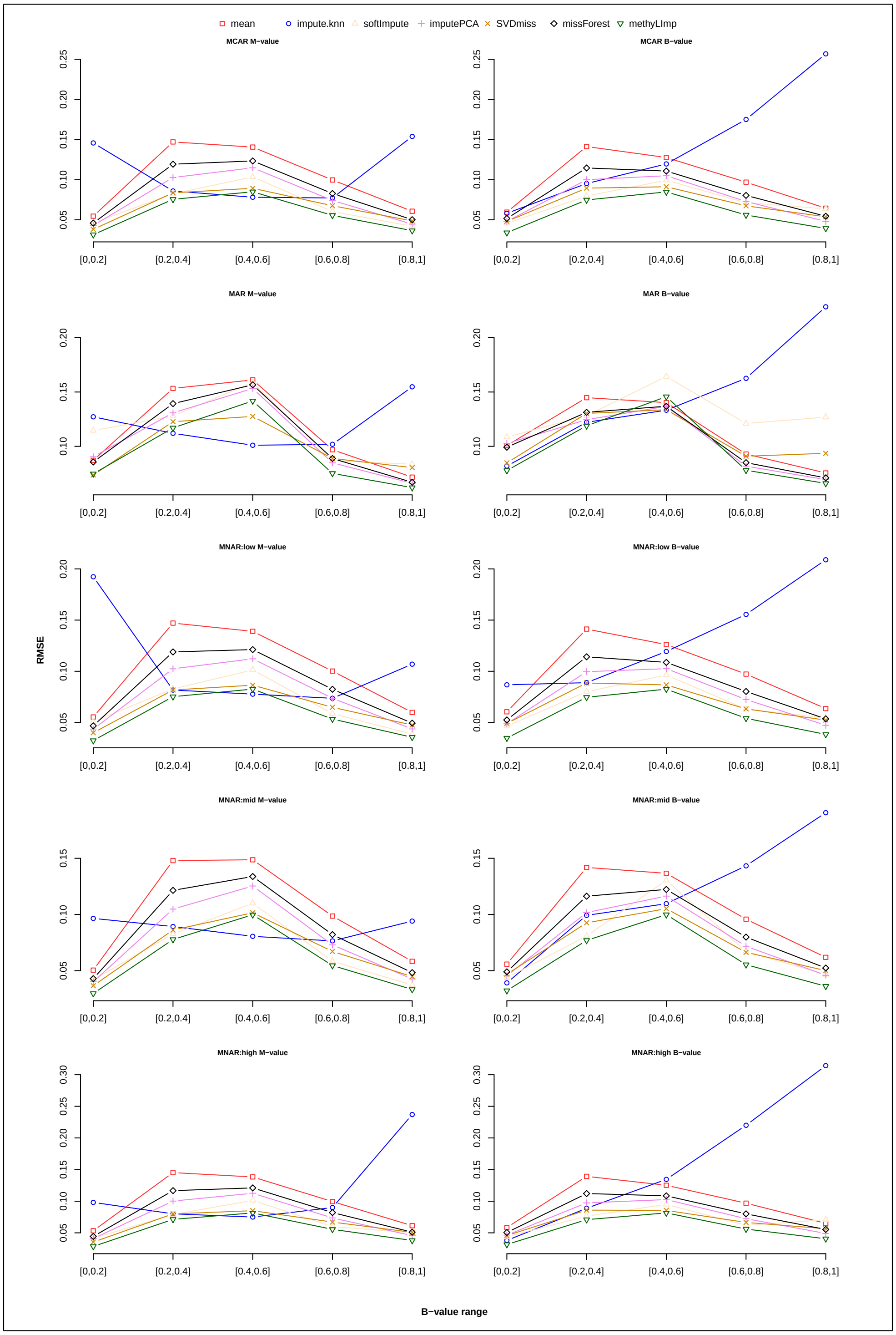

Figure 26: Dataset GSE44684 (D11). RMSE imputation performances with respect to B-value range.

2.12 GSE49393 (D12) - Prefrontal Cortex - Normal - 25 samples

Method	Avg time (sec)	Avg RAM (Mb)
mean	< 1	22
softImpute	< 1	59
imputePCA	8	188
impute.knn	1	68
SVDmiss	63	3527
methyLImp	126	101
missForest	14967	148

Table 81: Dataset GSE49393 (D12). Average time and memory usage.

Table 82: Dataset GSE49393 (D12). Imputation performance on **MCAR** type missing values.

	MAE		RMSE	
Method	M-value	B-value	M-value	B-value
mean	0.020±0.001*	0.020±0.001	0.034±0.001	0.033±0.001*
softImpute	0.020±0.001	0.017±0.001*	0.032±0.001	0.027±0.001*
impute.knn	0.021±0.004*	0.029±0.007	0.050±0.014*	0.071±0.016
imputePCA	0.016±0.001*	0.016±0.001	0.029±0.001	0.028±0.001*
SVDmiss	0.019±0.001	0.015±0.001*	0.031±0.001	0.023±0.001*
missForest	0.016±0.001	0.015±0.001*	0.025±0.001	0.023±0.001*
methyLImp	0.015±0.001	**0.015±0.001***	**0.023±0.001***	0.024±0.001

Table 83: Dataset GSE49393 (D12). Imputation performance on **MAR** type missing values.

	MAE		RMSE	
Method	M-value	B-value	M-value	B-value
mean	0.032±0.001*	0.033±0.001	0.061±0.002	0.057±0.001*
softImpute	0.030±0.001	0.028±0.001*	0.051±0.001	0.051±0.002
impute.knn	0.022±0.002*	0.028±0.004	0.043±0.008*	0.059±0.012
imputePCA	0.029±0.001*	0.030±0.001	0.059±0.002	0.056±0.001*
SVDmiss	0.029±0.001	**0.018±0.001***	0.052±0.003	**0.031±0.001***
missForest	0.022±0.001	0.021±0.001*	0.043±0.002	0.033±0.001*
methyLImp	0.026±0.001	0.025±0.001*	0.051±0.004	0.052±0.003

Table 84: Dataset GSE49393 (D12). Imputation performance on **MNAR:low** type missing values.

	MAE		RMSE	
Method	M-value	B-value	M-value	B-value
mean	0.014±0.001*	0.015±0.001	0.026±0.001*	0.028±0.001
softImpute	0.014±0.001	0.013±0.001*	0.026±0.001	0.023±0.001*
impute.knn	0.017±0.004	0.016±0.003	0.045±0.017	0.042±0.012
imputePCA	0.012±0.001*	0.013±0.001	0.023±0.001*	0.024±0.001
SVDmiss	0.014±0.001	0.012±0.001*	0.026±0.003	0.018±0.001*
missForest	0.012±0.001	0.012±0.001	0.020±0.001	0.019±0.001*
methyLImp	0.011±0.001	**0.011±0.001***	**0.018±0.001***	0.019±0.001

Table 85: Dataset GSE49393 (D12). Imputation performance on **MNAR:mid** type missing values.

	MAE		RMSE	
Method	M-value	B-value	M-value	B-value
mean	0.033±0.001	0.032±0.001*	0.055±0.001	0.050±0.001*
softImpute	0.032±0.001	0.026±0.001*	0.049±0.001	0.041±0.001*
impute.knn	0.023±0.001*	0.025±0.002	0.039±0.005*	0.045±0.008
imputePCA	0.026±0.001	0.025±0.001*	0.048±0.001	0.043±0.001*
SVDmiss	0.031±0.002	**0.021±0.001***	0.051±0.004	**0.031±0.001***
missForest	0.024±0.001	0.023±0.001*	0.040±0.001	0.033±0.001*
methyLImp	0.022±0.001	0.022±0.001*	0.033±0.001*	0.036±0.001

Table 86: Dataset GSE49393 (D12). Imputation performance on **MNAR:high** type missing values.

	MAE		RMSE	
Method	M-value	B-value	M-value	B-value
mean	0.016±0.001*	0.017±0.001	0.027±0.001	0.027±0.001
softImpute	0.017±0.001	0.015±0.001*	0.027±0.001	0.022±0.001*
impute.knn	0.021±0.005*	0.044±0.011	0.055±0.019*	0.105±0.020
imputePCA	0.014±0.001*	0.014±0.001	0.023±0.001	0.023±0.001
SVDmiss	0.016±0.001	0.014±0.001*	0.026±0.001	0.020±0.001*
missForest	0.014±0.001*	0.014±0.001	0.021±0.001	0.020±0.001*
methyLImp	0.014±0.001	**0.013±0.001***	**0.020±0.001***	0.020±0.001

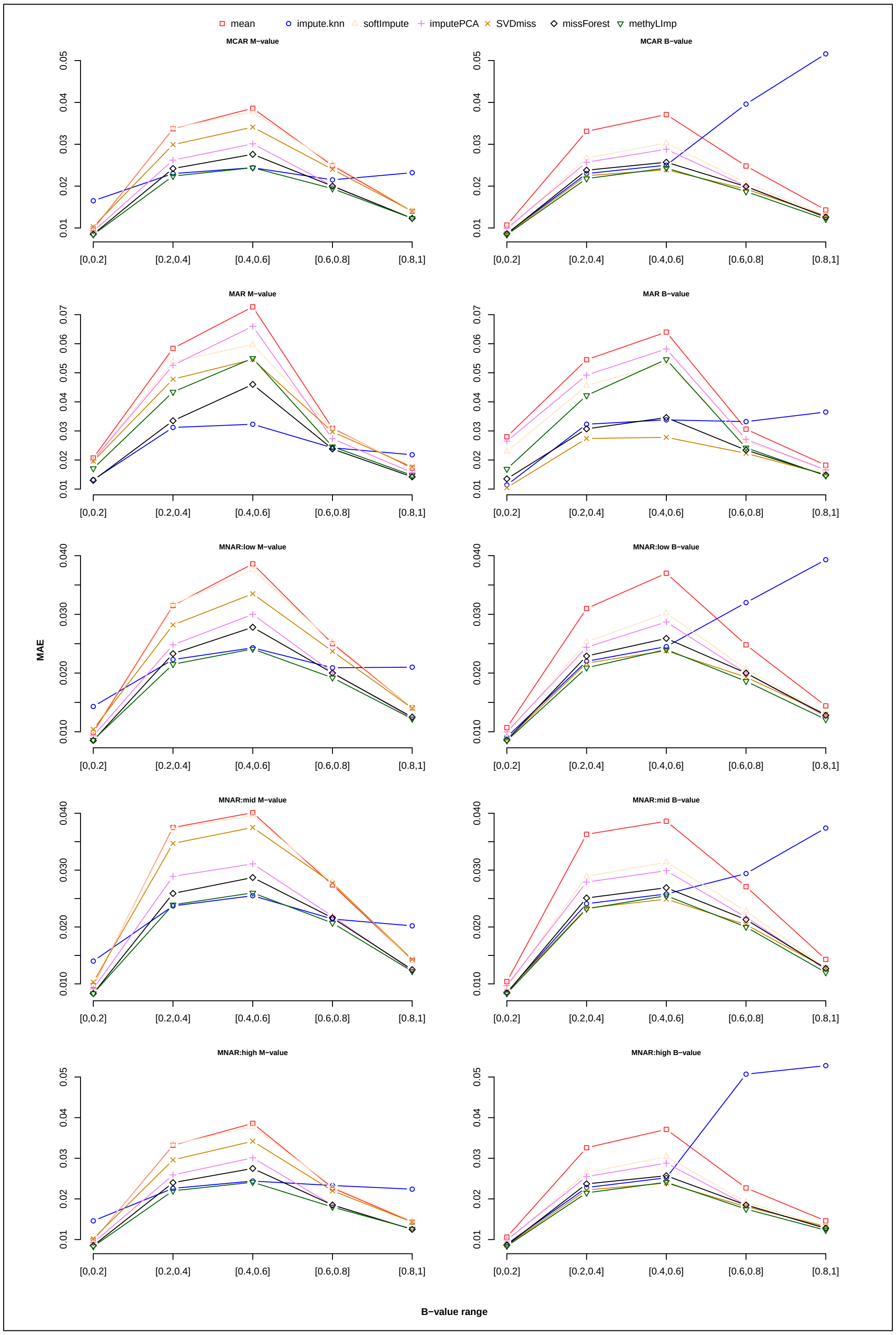

Figure 27: Dataset GSE49393 (D12). MAE imputation performances with respect to B-value range.

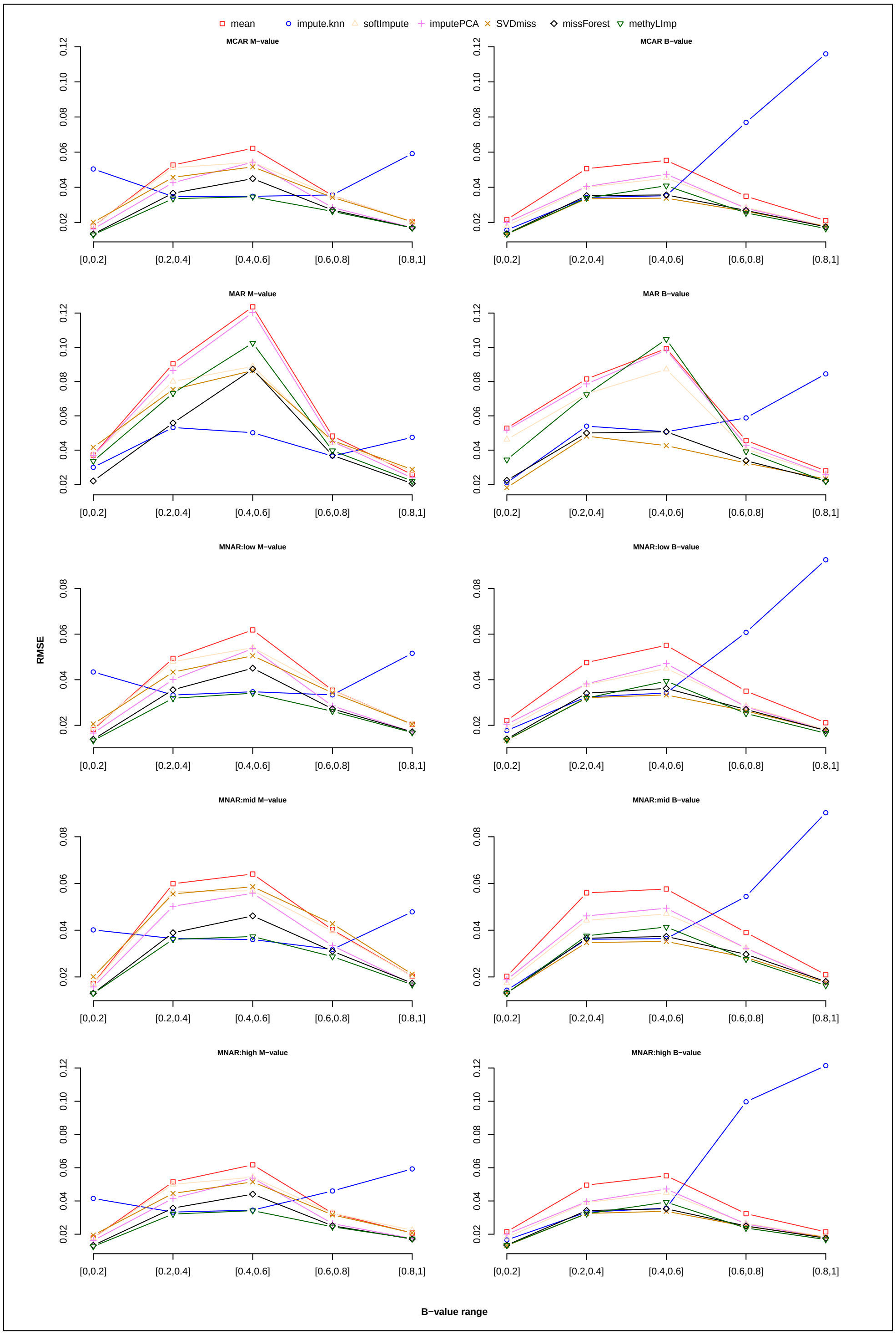

Figure 28: Dataset GSE49393 (D12). RMSE imputation performances with respect to B-value range.

2.13 GSE51388 (D13) - Blood - Normal - 60 samples

Method	Avg time (sec)	Avg RAM (Mb)
mean	< 1	56
softImpute	1	109
imputePCA	111	258
impute.knn	2	123
SVDmiss	157	3246
methyLImp	902	128
missForest	52391	290

Table 87: Dataset GSE51388 (D13). Average time and memory usage.

Table 88: Dataset GSE51388 (D13). Imputation performance on **MCAR** type missing values.

	MAE		RMSE	
Method	M-value	B-value	M-value	B-value
mean	0.018±0.001*	0.018±0.001	0.031±0.001	0.031±0.001*
softImpute	0.017±0.001*	0.017±0.001	0.030±0.001*	0.030±0.001
impute.knn	0.020±0.001*	0.024±0.003	0.051±0.003*	0.064±0.008
imputePCA	0.016±0.001*	0.016±0.001	0.028±0.001	0.027±0.001*
SVDmiss	0.015±0.001	0.015±0.001*	0.026±0.001	0.025±0.001*
missForest	0.014±0.001*	0.014±0.001	0.023±0.001	0.023±0.001*
methyLImp	**0.013±0.001***	0.013±0.001	0.019±0.001	**0.019±0.001***

Table 89: Dataset GSE51388 (D13). Imputation performance on **MAR** type missing values.

	MAE		RMSE	
Method	M-value	B-value	M-value	B-value
mean	0.023±0.001*	0.024±0.001	0.048±0.001	0.047±0.001*
softImpute	0.023±0.001*	0.023±0.001	0.047±0.001	0.047±0.001*
impute.knn	0.030±0.001*	0.033±0.002	0.075±0.003*	0.083±0.008
imputePCA	0.021±0.001*	0.022±0.001	0.045±0.001	0.045±0.001*
SVDmiss	0.020±0.001	0.020±0.001	0.042±0.001	0.041±0.001*
missForest	0.018±0.001*	0.019±0.001	0.037±0.001	0.037±0.001
methyLImp	0.015±0.001	**0.015±0.001***	0.027±0.001	**0.024±0.001***

Table 90: Dataset GSE51388 (D13). Imputation performance on **MNAR:low** type missing values.

	MAE		RMSE	
Method	M-value	B-value	M-value	B-value
mean	0.015±0.001*	0.015±0.001	0.027±0.001*	0.028±0.001
softImpute	0.014±0.001*	0.014±0.001	0.026±0.001*	0.027±0.001
impute.knn	0.018±0.001*	0.021±0.001	0.056±0.002*	0.065±0.004
imputePCA	0.013±0.001*	0.013±0.001	0.024±0.001*	0.024±0.001
SVDmiss	0.012±0.001*	0.013±0.001	0.023±0.001	0.023±0.001
missForest	0.012±0.001*	0.012±0.001	0.021±0.001*	0.021±0.001
methyLImp	**0.011±0.001***	0.011±0.001	0.017±0.001	**0.016±0.001***

Table 91: Dataset GSE51388 (D13). Imputation performance on **MNAR:mid** type missing values.

	MAE		RMSE	
Method	M-value	B-value	M-value	B-value
mean	0.033±0.001	0.033±0.001*	0.053±0.001	0.051±0.001*
softImpute	0.032±0.001	0.032±0.001*	0.052±0.001	0.050±0.001*
impute.knn	0.029±0.001*	0.031±0.001	0.052±0.001*	0.059±0.005
imputePCA	0.029±0.001	0.028±0.001*	0.047±0.001	0.045±0.001*
SVDmiss	0.028±0.001	0.025±0.001*	0.045±0.001	0.040±0.001*
missForest	0.025±0.001	0.025±0.001*	0.040±0.001	0.038±0.001*
methyLImp	0.021±0.001	**0.019±0.001***	0.033±0.001	**0.031±0.001***

Table 92: Dataset GSE51388 (D13). Imputation performance on **MNAR:high** type missing values.

	MAE		RMSE	
Method	M-value	B-value	M-value	B-value
mean	0.015±0.001*	0.015±0.001	0.027±0.001*	0.027±0.001
softImpute	0.015±0.001*	0.015±0.001	0.026±0.001*	0.026±0.001
impute.knn	0.019±0.001*	0.023±0.003	0.050±0.002*	0.063±0.009
imputePCA	0.014±0.001*	0.014±0.001	0.024±0.001*	0.024±0.001
SVDmiss	0.013±0.001*	0.013±0.001	0.023±0.001	0.023±0.001*
missForest	0.013±0.001*	0.013±0.001	0.021±0.001	0.021±0.001
methyLImp	**0.012±0.001***	0.012±0.001	0.017±0.001	**0.017±0.001***

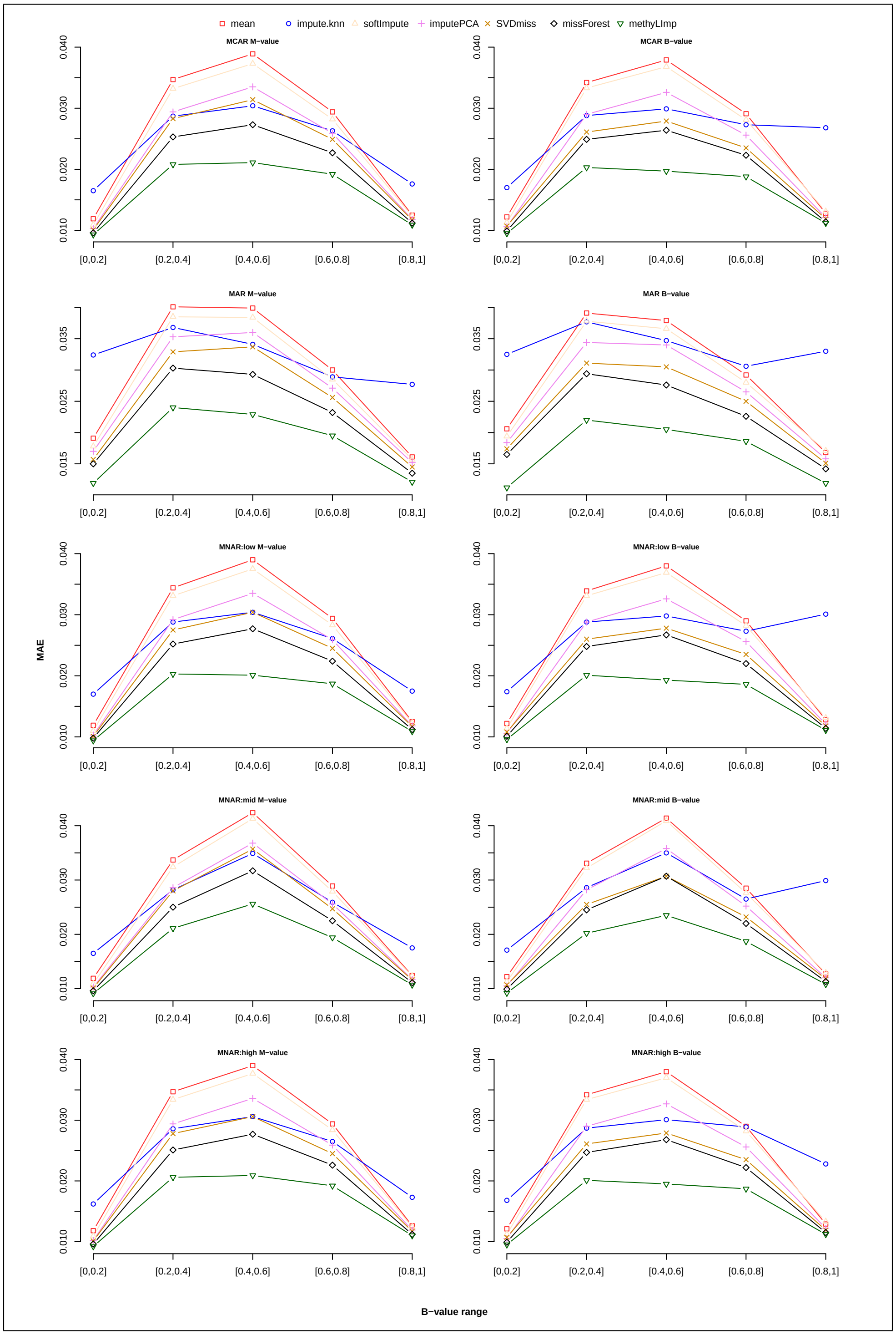

Figure 29: Dataset GSE51388 (D13). MAE imputation performances with respect to B-value range.

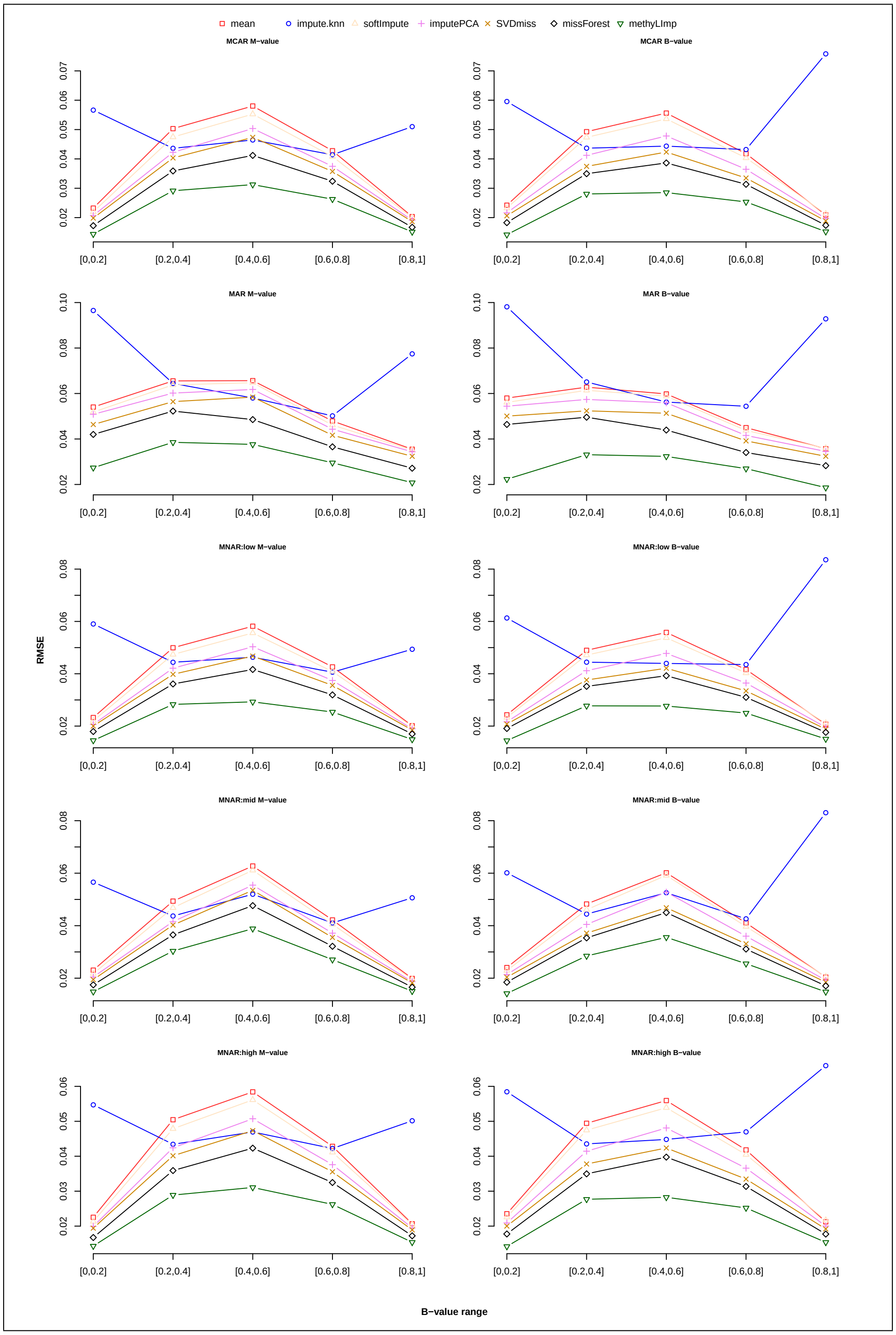

Figure 30: Dataset GSE51388 (D13). RMSE imputation performances with respect to B-value range.

2.14 GSE52113 (D14) - Blood - Normal - 24 samples

Method	Avg time (sec)	Avg RAM (Mb)
mean	< 1	20
softImpute	< 1	58
imputePCA	8	188
impute.knn	1	69
SVDmiss	88	4236
methyLImp	130	101
missForest	42752	150

Table 93: Dataset GSE52113 (D14). Average time and memory usage.

Table 94: Dataset GSE52113 (D14). Imputation performance on **MCAR** type missing values.

	MAE		RMSE	
Method	M-value	B-value	M-value	B-value
mean	0.022±0.001*	0.022±0.001	0.041±0.001	0.040±0.001*
softImpute	0.020±0.001	0.019±0.001*	0.035±0.001	0.034±0.001*
impute.knn	0.030±0.006*	0.039±0.009	0.077±0.017*	0.094±0.017
imputePCA	0.019±0.001	0.019±0.001	0.038±0.001	0.037±0.001*
SVDmiss	0.018±0.001*	0.018±0.001	0.033±0.001	0.032±0.001*
missForest	0.018±0.001*	0.018±0.001	0.031±0.001	0.031±0.001
methyLImp	**0.016±0.001***	0.016±0.001	0.025±0.001	**0.024±0.001***

Table 95: Dataset GSE52113 (D14). Imputation performance on **MAR** type missing values.

	MAE		RMSE	
Method	M-value	B-value	M-value	B-value
mean	0.039±0.001*	0.039±0.001	0.075±0.001	0.072±0.001*
softImpute	0.027±0.001	0.027±0.001*	0.054±0.003	0.052±0.001*
impute.knn	0.031±0.003*	0.035±0.003	0.067±0.009*	0.076±0.008
imputePCA	0.036±0.001	0.035±0.001*	0.071±0.001	0.069±0.001*
SVDmiss	0.026±0.001*	0.026±0.001	0.052±0.001	0.051±0.001*
missForest	0.027±0.001*	0.027±0.001	0.052±0.001	0.051±0.001*
methyLImp	0.028±0.001	**0.025±0.001***	0.054±0.002	**0.046±0.002***

Table 96: Dataset GSE52113 (D14). Imputation performance on **MNAR:low** type missing values.

	MAE		RMSE	
Method	M-value	B-value	M-value	B-value
mean	0.018±0.001*	0.018±0.001	0.035±0.001*	0.038±0.001
softImpute	0.016±0.001*	0.016±0.001	0.030±0.001*	0.030±0.001
impute.knn	0.023±0.006	0.021±0.003	0.065±0.021	0.058±0.011*
imputePCA	0.016±0.001*	0.016±0.001	0.034±0.001*	0.035±0.001
SVDmiss	0.014±0.001*	0.015±0.001	0.028±0.001*	0.028±0.001
missForest	0.014±0.001*	0.014±0.001	0.027±0.001*	0.028±0.001
methyLImp	**0.013±0.001***	0.013±0.001	0.020±0.001	**0.020±0.001***

Table 97: Dataset GSE52113 (D14). Imputation performance on **MNAR:mid** type missing values.

	MAE		RMSE	
Method	M-value	B-value	M-value	B-value
mean	0.046±0.001	0.044±0.001*	0.081±0.001	0.072±0.001*
softImpute	0.038±0.001	0.036±0.001*	0.060±0.001	0.056±0.001*
impute.knn	0.034±0.001*	0.037±0.003	0.061±0.004*	0.067±0.007
imputePCA	0.042±0.001	0.039±0.001*	0.075±0.001	0.066±0.001*
SVDmiss	0.034±0.001	0.033±0.001*	0.057±0.001	0.054±0.001*
missForest	0.035±0.001	0.034±0.001*	0.058±0.001	0.054±0.001*
methyLImp	0.031±0.001	**0.029±0.001***	0.050±0.001	**0.046±0.001***

Table 98: Dataset GSE52113 (D14). Imputation performance on **MNAR:high** type missing values.

	MAE		RMSE	
Method	M-value	B-value	M-value	B-value
mean	0.018±0.001*	0.019±0.001	0.034±0.001*	0.034±0.001
softImpute	0.017±0.001	0.017±0.001*	0.030±0.001	0.030±0.001*
impute.knn	0.030±0.007*	0.048±0.013	0.079±0.018*	0.112±0.022
imputePCA	0.017±0.001*	0.017±0.001	0.032±0.001	0.032±0.001*
SVDmiss	0.016±0.001*	0.017±0.001	0.029±0.001*	0.029±0.001
missForest	0.015±0.001*	0.016±0.001	0.027±0.001*	0.028±0.001
methyLImp	**0.014±0.001***	0.015±0.001	0.022±0.001	**0.022±0.001***

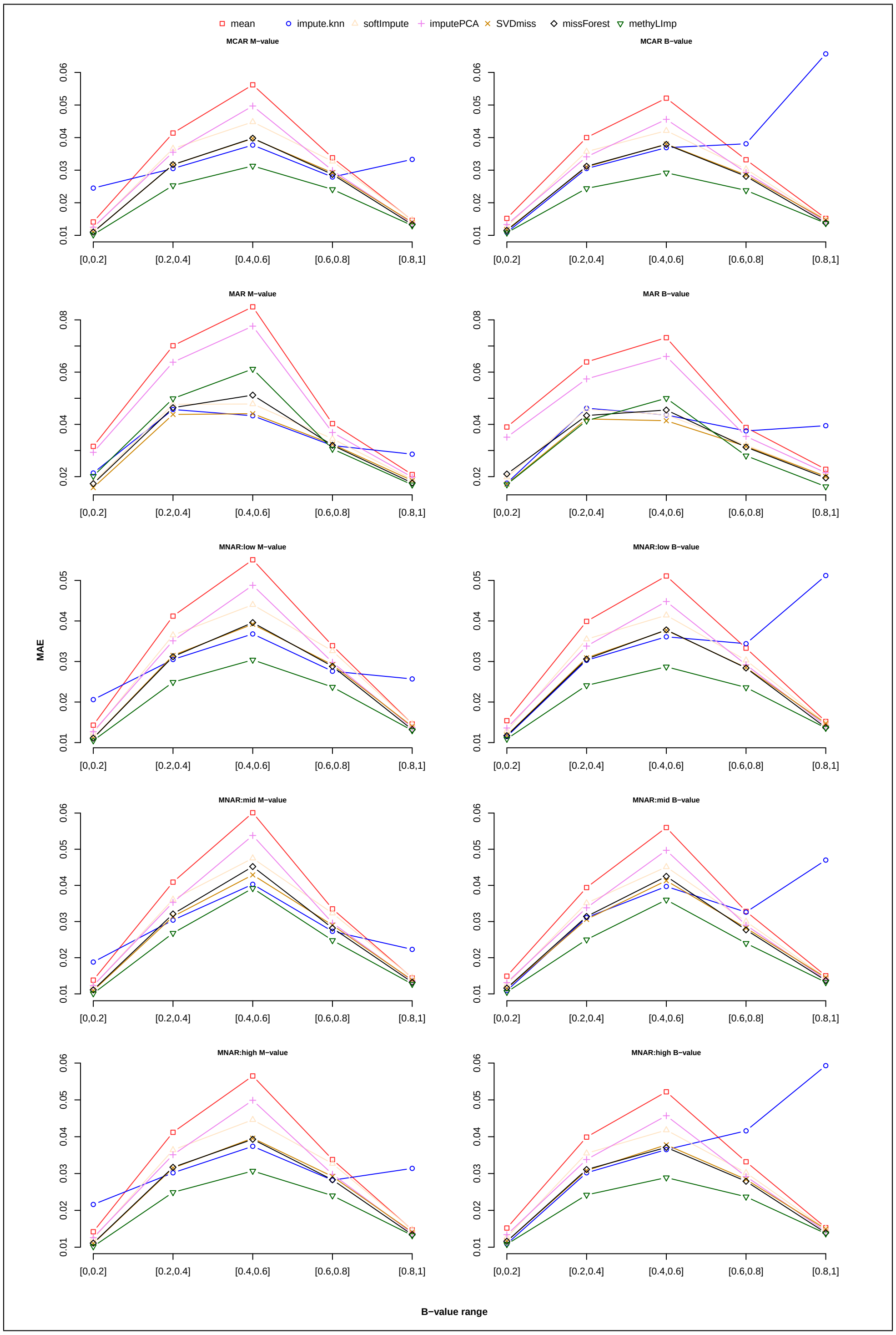

Figure 31: Dataset GSE52113 (D14). MAE imputation performances with respect to B-value range.

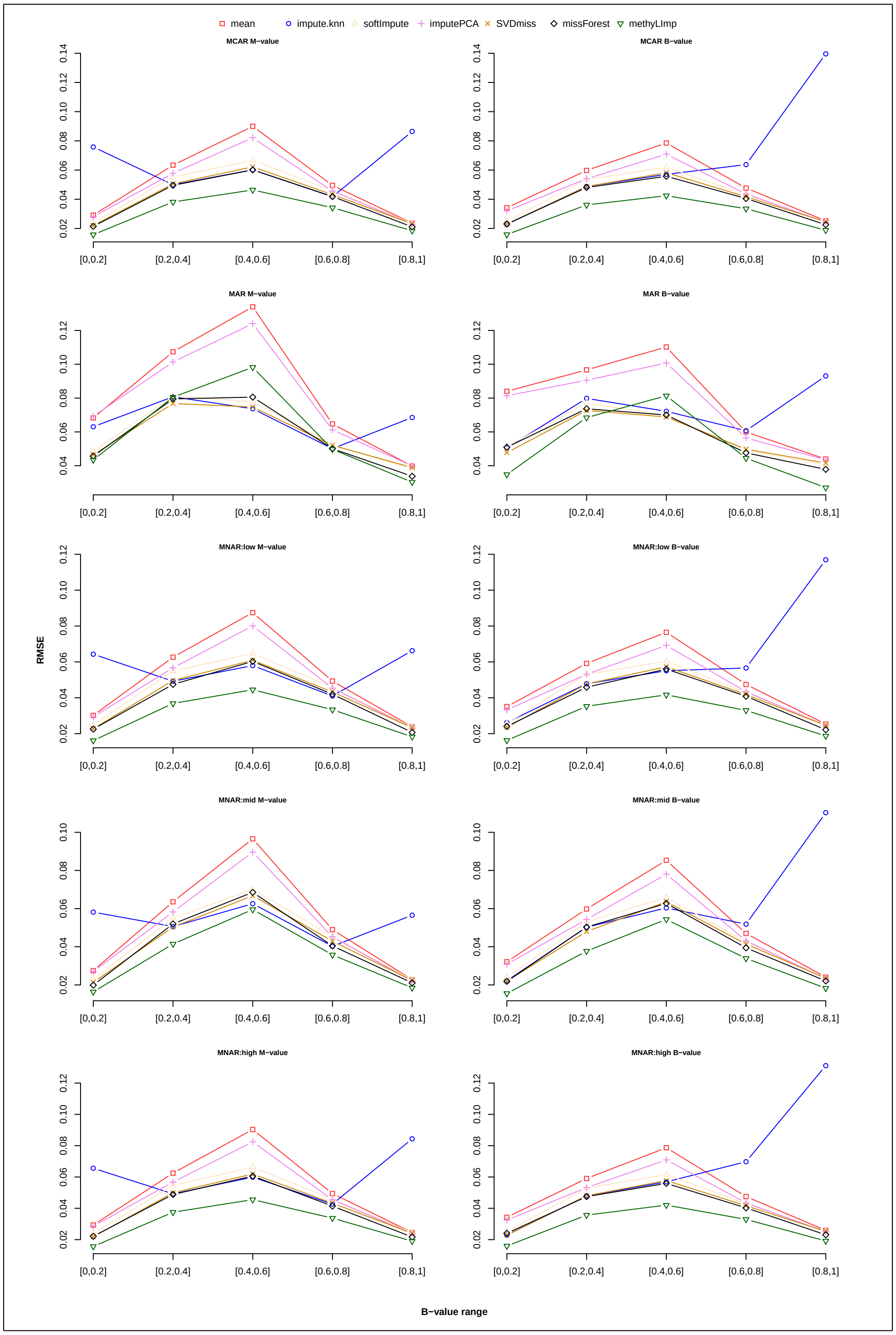

Figure 32: Dataset GSE52113 (D14). RMSE imputation performances with respect to B-value range.

2.15 GSE53051 (D15) - Breast - Cancer - 14 samples

Method	Avg time (sec)	Avg RAM (Mb)
mean	< 1	12
softImpute	1	50
imputePCA	7	162
impute.knn	< 1	51
SVDmiss	36	4094
methyLImp	19	108
missForest	8086	112

Table 99: Dataset GSE53051 (D15). Average time and memory usage.

Table 100: Dataset GSE53051 (D15). Imputation performance on **MCAR** type missing values.

	MAE		RMSE	
Method	M-value	B-value	M-value	B-value
mean	0.068±0.001*	0.068±0.001	0.112±0.002	0.109±0.001*
softImpute	0.079±0.002	0.068±0.001*	0.131±0.004	0.110±0.003*
impute.knn	0.069±0.003*	0.095±0.010	0.113±0.004*	0.157±0.016
imputePCA	0.066±0.001*	0.067±0.001	0.111±0.002	0.107±0.001*
SVDmiss	0.093±0.002	0.078±0.001*	0.158±0.004	0.140±0.003*
missForest	0.065±0.001*	0.066±0.001	0.109±0.002	0.105±0.001*
methyLImp	0.070±0.001	**0.062±0.001***	0.112±0.002	**0.105±0.002***

Table 101: Dataset GSE53051 (D15). Imputation performance on **MAR** type missing values.

	MAE		RMSE	
Method	M-value	B-value	M-value	B-value
mean	0.076±0.001	0.075±0.001*	0.123±0.002	0.115±0.001*
softImpute	0.087±0.002	0.075±0.002*	0.141±0.004	0.118±0.004*
impute.knn	0.079±0.002*	0.095±0.005	0.126±0.003*	0.151±0.009
imputePCA	0.074±0.001	0.073±0.001*	0.122±0.002	0.114±0.001*
SVDmiss	0.102±0.002	0.086±0.001*	0.166±0.003	0.148±0.003*
missForest	0.073±0.001	0.073±0.001*	0.120±0.002	**0.112±0.001***
methyLImp	0.077±0.001	**0.071±0.001***	0.122±0.002	0.118±0.002*

Table 102: Dataset GSE53051 (D15). Imputation performance on **MNAR:low** type missing values.

	MAE		RMSE	
Method	M-value	B-value	M-value	B-value
mean	0.050±0.001*	0.052±0.001	0.095±0.002*	0.096±0.002
softImpute	0.063±0.002	0.052±0.001*	0.119±0.005	0.097±0.003*
impute.knn	0.055±0.006*	0.057±0.004	0.104±0.011*	0.109±0.009
imputePCA	0.049±0.001*	0.051±0.001	0.097±0.002	0.095±0.002*
SVDmiss	0.075±0.002	0.062±0.001*	0.151±0.004	0.127±0.003*
missForest	0.048±0.001*	0.051±0.001	**0.092±0.002***	0.094±0.001
methyLImp	0.052±0.001	**0.048±0.001***	0.094±0.002*	0.096±0.002

Table 103: Dataset GSE53051 (D15). Imputation performance on **MNAR:mid** type missing values.

	MAE		RMSE	
Method	M-value	B-value	M-value	B-value
mean	0.101±0.001	0.094±0.001*	0.142±0.001	0.132±0.001*
softImpute	0.110±0.002	0.094±0.002*	0.158±0.003	0.133±0.003*
impute.knn	**0.088±0.001***	0.093±0.003	0.128±0.002*	0.136±0.005
imputePCA	0.099±0.001	0.094±0.001*	0.141±0.001	0.132±0.001*
SVDmiss	0.132±0.002	0.103±0.001*	0.190±0.002	0.162±0.003*
missForest	0.096±0.001	0.089±0.001*	0.137±0.001	**0.126±0.001***
methyLImp	0.101±0.001	**0.089±0.001***	0.142±0.001	0.129±0.001*

Table 104: Dataset GSE53051 (D15). Imputation performance on **MNAR:high** type missing values.

	MAE		RMSE	
Method	M-value	B-value	M-value	B-value
mean	0.058±0.001*	0.062±0.001	0.098±0.002	0.097±0.001*
softImpute	0.067±0.002	0.063±0.001*	0.112±0.004	0.100±0.003*
impute.knn	0.068±0.005*	0.128±0.020	0.115±0.009*	0.204±0.025
imputePCA	0.056±0.001*	0.059±0.001	0.095±0.002	0.095±0.001*
SVDmiss	0.079±0.001	0.073±0.001*	0.134±0.003	0.129±0.003*
missForest	0.056±0.001*	0.060±0.001	0.095±0.002	0.095±0.001
methyLImp	0.062±0.001	**0.053±0.001***	0.099±0.002	**0.090±0.002***

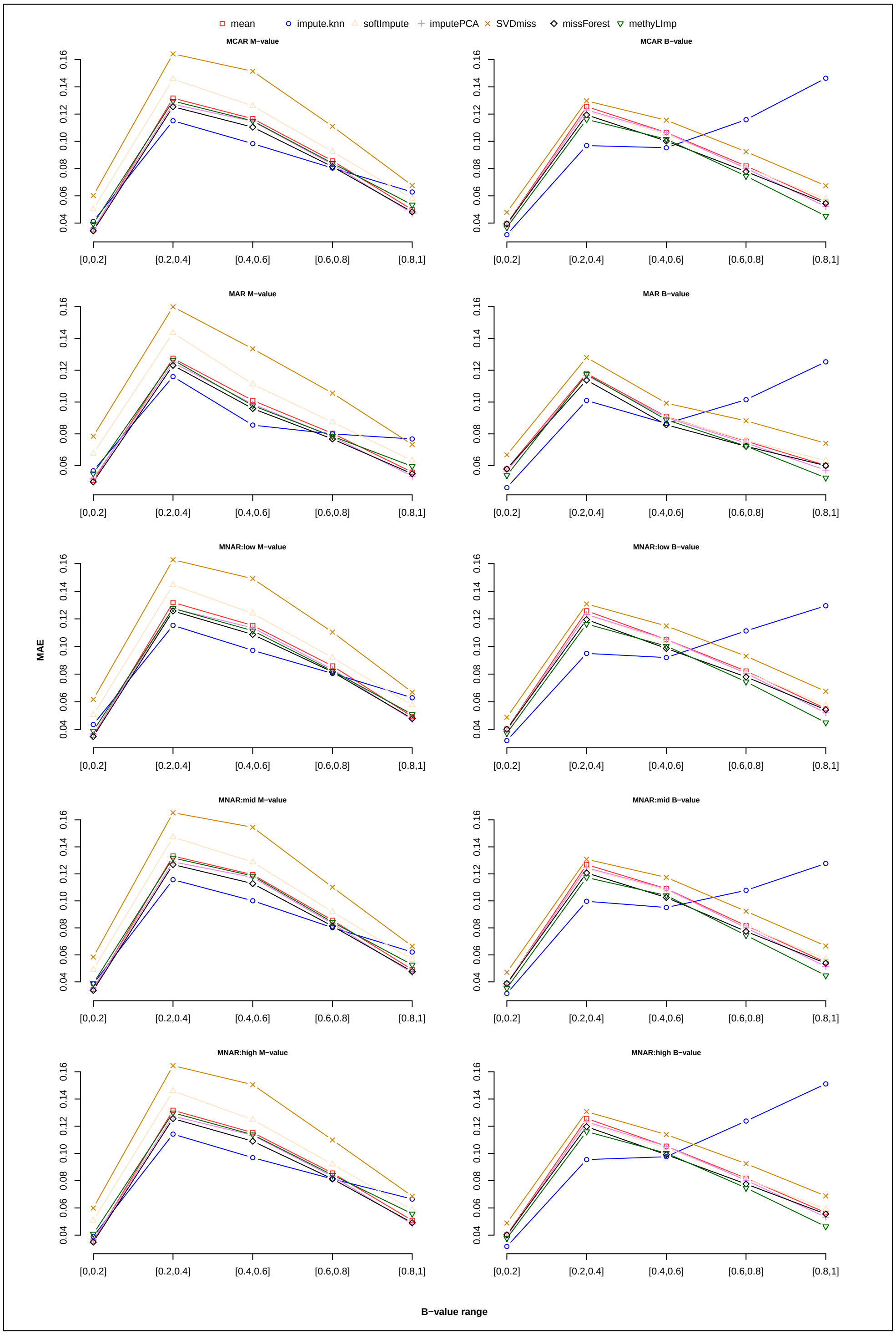

Figure 33: Dataset GSE53051 (D15). MAE imputation performances with respect to B-value range.

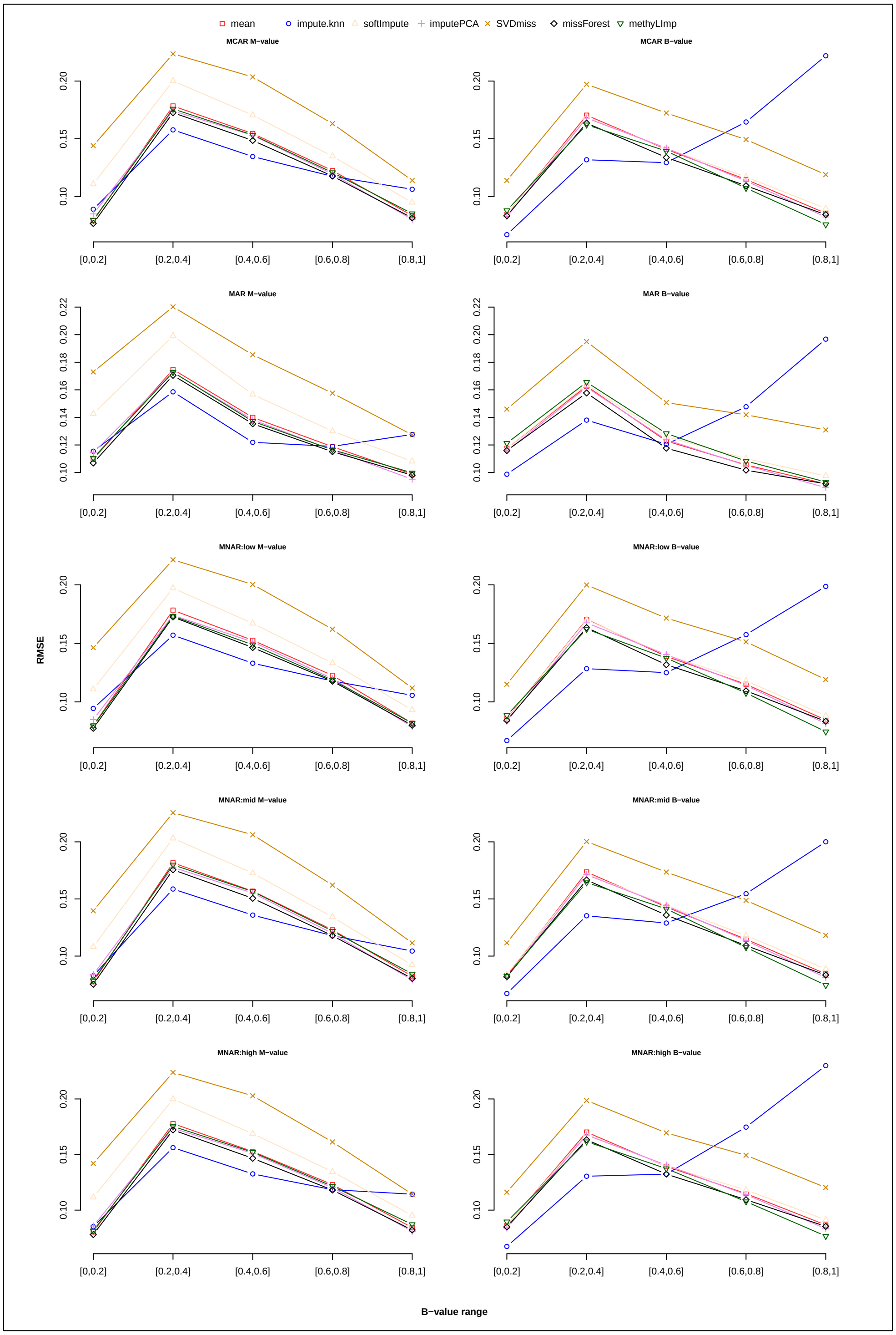

Figure 34: Dataset GSE53051 (D15). RMSE imputation performances with respect to B-value range.

2.16 GSE53051 (D16) - Colon - Cancer - 35 samples

Method	Avg time (sec)	Avg RAM (Mb)
mean	< 1	29
softImpute	1	80
imputePCA	12	207
impute.knn	1	93
SVDmiss	136	4913
methyLImp	504	121
missForest	40506	214

Table 105: Dataset GSE53051 (D16). Average time and memory usage.

Table 106: Dataset GSE53051 (D16). Imputation performance on **MCAR** type missing values.

	MAE		RMSE	
Method	M-value	B-value	M-value	B-value
mean	0.068±0.001	0.068±0.001*	0.111±0.001	0.107±0.001*
softImpute	0.077±0.001	0.061±0.001*	0.121±0.001	0.096±0.001*
impute.knn	0.056±0.001*	0.060±0.002	0.094±0.001*	0.100±0.005
imputePCA	0.061±0.001	0.059±0.001*	0.102±0.001	0.094±0.001*
SVDmiss	0.081±0.001	0.058±0.001*	0.139±0.002	0.092±0.001*
missForest	0.058±0.001	0.057±0.001	0.096±0.001	**0.091±0.001***
methyLImp	0.067±0.001	**0.055±0.001***	0.108±0.001	0.094±0.001*

Table 107: Dataset GSE53051 (D16). Imputation performance on **MAR** type missing values.

	MAE		RMSE	
Method	M-value	B-value	M-value	B-value
mean	0.082±0.001	0.081±0.001*	0.134±0.001	0.121±0.001*
softImpute	0.090±0.001	0.074±0.001*	0.141±0.002	0.113±0.001*
impute.knn	0.068±0.001	0.068±0.001	0.114±0.001	0.108±0.003*
imputePCA	0.075±0.001	0.072±0.001*	0.126±0.001	0.111±0.001*
SVDmiss	0.094±0.001	0.070±0.001*	0.157±0.002	0.110±0.001*
missForest	0.070±0.001	**0.066±0.001***	0.119±0.001	**0.102±0.001***
methyLImp	0.079±0.001	0.070±0.001*	0.130±0.001	0.122±0.001*

Table 108: Dataset GSE53051 (D16). Imputation performance on **MNAR:low** type missing values.

	MAE		RMSE	
Method	M-value	B-value	M-value	B-value
mean	0.050±0.001*	0.053±0.001	0.093±0.001*	0.095±0.001
softImpute	0.061±0.001	0.048±0.001*	0.108±0.004	0.086±0.001*
impute.knn	**0.040±0.001***	0.043±0.001	**0.078±0.002***	0.081±0.004
imputePCA	0.046±0.001*	0.046±0.001	0.088±0.001	0.084±0.001*
SVDmiss	0.065±0.001	0.045±0.001*	0.128±0.002	0.082±0.001*
missForest	0.042±0.001*	0.045±0.001	0.079±0.002*	0.081±0.001
methyLImp	0.050±0.001	0.043±0.001*	0.098±0.003	0.085±0.002*

Table 109: Dataset GSE53051 (D16). Imputation performance on **MNAR:mid** type missing values.

	MAE		RMSE	
Method	M-value	B-value	M-value	B-value
mean	0.097±0.001	0.089±0.001*	0.137±0.001	0.123±0.001*
softImpute	0.100±0.001	0.082±0.001*	0.143±0.001	0.115±0.001*
impute.knn	0.078±0.001	**0.074±0.001***	0.116±0.001	0.108±0.002*
imputePCA	0.087±0.001	0.079±0.001*	0.128±0.001	0.113±0.001*
SVDmiss	0.106±0.001	0.077±0.001*	0.156±0.001	0.110±0.001*
missForest	0.082±0.001	**0.074±0.001***	0.120±0.001	**0.105±0.001***
methyLImp	0.096±0.001	0.076±0.001*	0.137±0.001	0.114±0.001*

Table 110: Dataset GSE53051 (D16). Imputation performance on **MNAR:high** type missing values.

	MAE		RMSE	
Method	M-value	B-value	M-value	B-value
mean	0.057±0.001*	0.060±0.001	0.097±0.001	0.094±0.001*
softImpute	0.068±0.001	0.056±0.001*	0.106±0.001	0.087±0.001*
impute.knn	0.049±0.001*	0.061±0.006	0.084±0.002*	0.106±0.012
imputePCA	0.051±0.001*	0.052±0.001	0.088±0.001	0.083±0.001*
SVDmiss	0.071±0.001	0.053±0.001*	0.130±0.002	0.083±0.001*
missForest	0.049±0.001*	0.052±0.001	0.086±0.001	0.083±0.001*
methyLImp	0.056±0.001	**0.047±0.001***	0.093±0.001	**0.082±0.001***

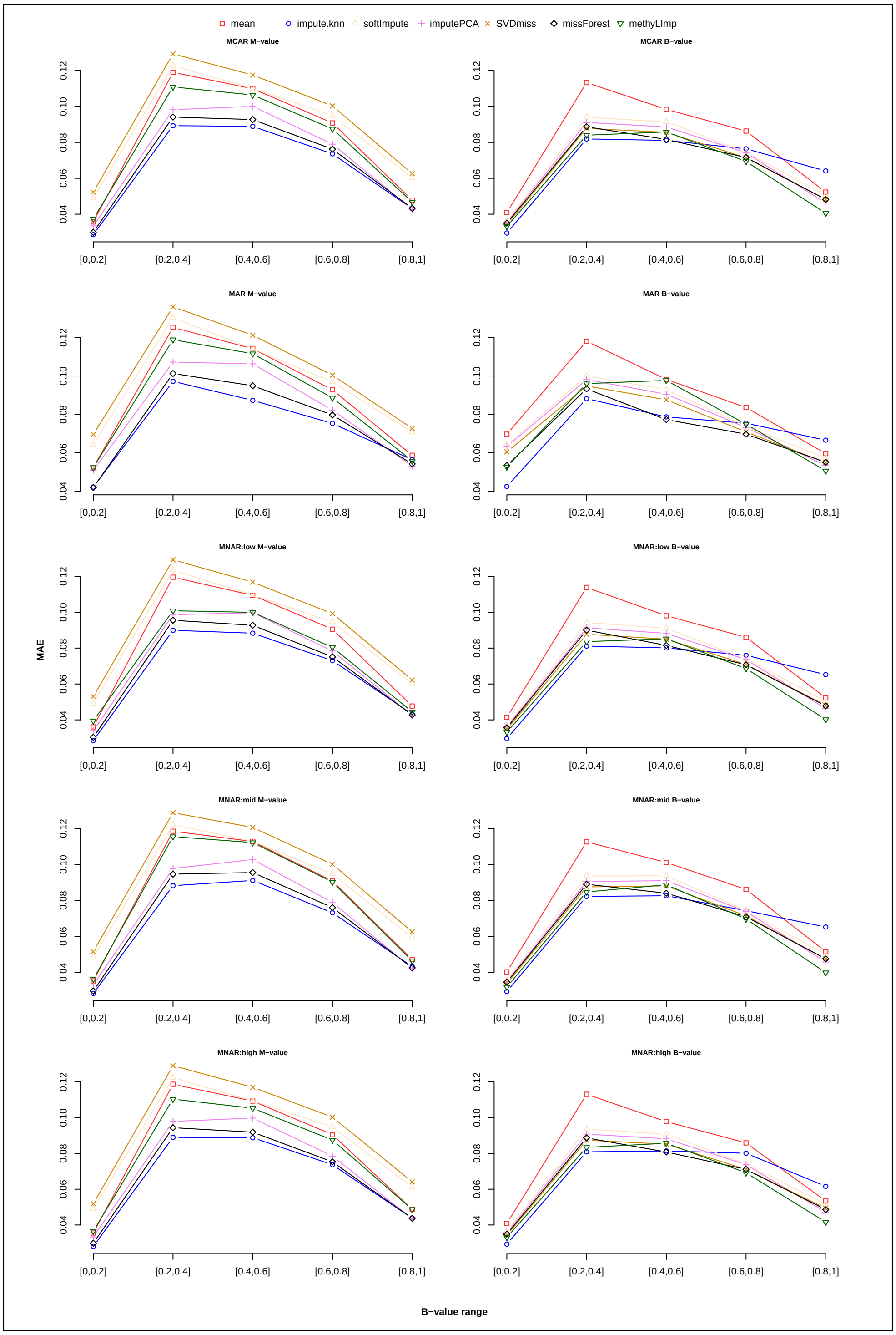

Figure 35: Dataset GSE53051 (D16). MAE imputation performances with respect to B-value range.

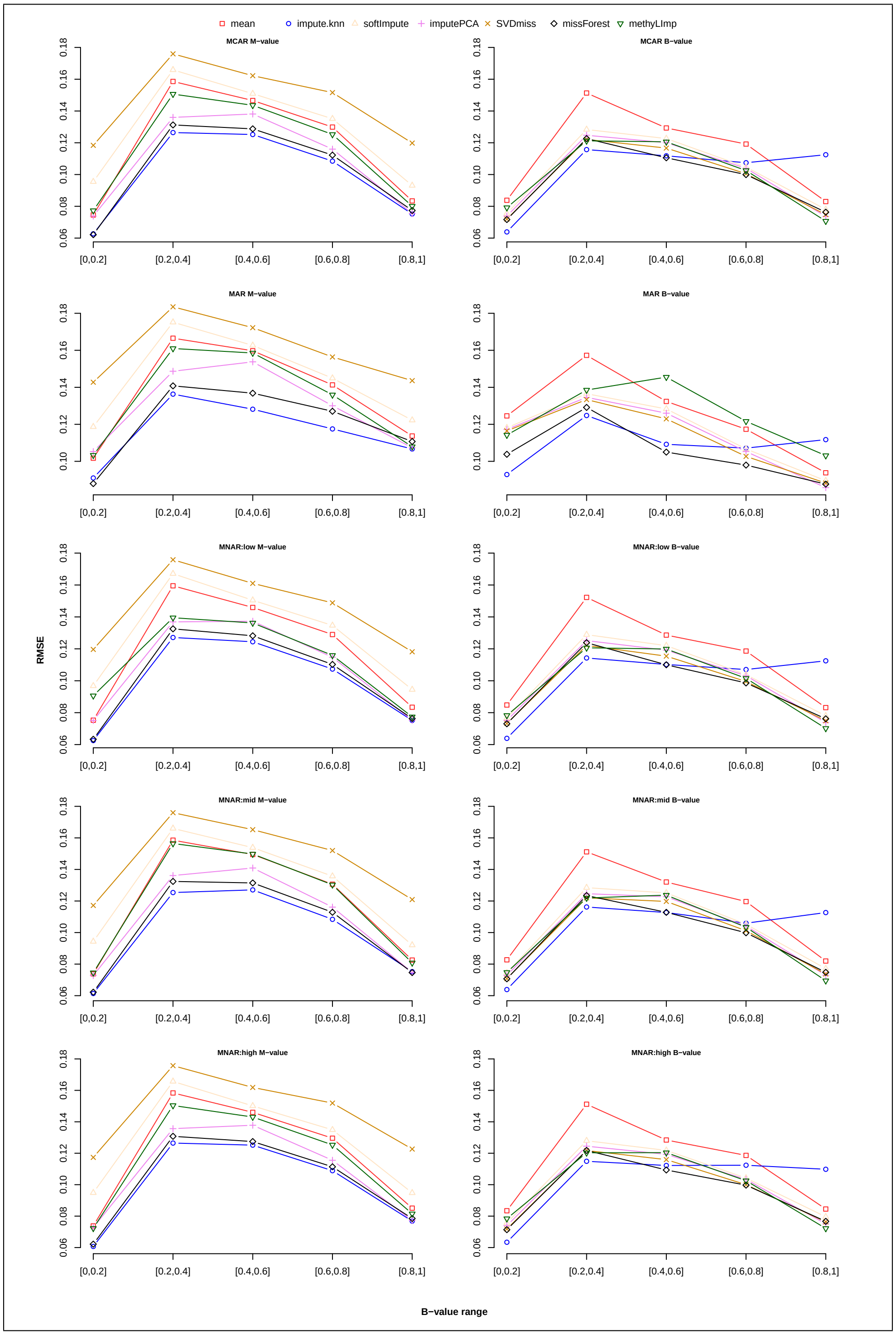

Figure 36: Dataset GSE53051 (D16). RMSE imputation performances with respect to B-value range.

2.17 GSE53051 (D17) - Colon, Pancreas - Normal - 9 samples

Method	Avg time (sec)	Avg RAM (Mb)
mean	< 1	8
softImpute	< 1	46
imputePCA	6	154
impute.knn	< 1	50
SVDmiss	16	3890
methyLImp	5	127
missForest	1986	284

Table 111: Dataset GSE53051 (D17). Average time and memory usage.

Table 112: Dataset GSE53051 (D17). Imputation performance on **MCAR** type missing values.

	MAE		RMSE	
Method	M-value	B-value	M-value	B-value
mean	0.050±0.001	0.050±0.001*	0.081±0.002	0.078±0.001*
softImpute	0.070±0.008	0.035±0.001*	0.118±0.022	0.058±0.002*
impute.knn	0.040±0.005*	0.070±0.017	0.071±0.013*	0.129±0.027
imputePCA	0.037±0.001	0.037±0.001	0.062±0.002	0.060±0.001*
SVDmiss	0.068±0.003	0.036±0.001*	0.114±0.006	**0.057±0.002***
missForest	0.040±0.001	0.040±0.001*	0.066±0.002	0.062±0.001*
methyLImp	0.051±0.001	**0.035±0.001***	0.077±0.002	0.065±0.004*

Table 113: Dataset GSE53051 (D17). Imputation performance on **MAR** type missing values.

	MAE		RMSE	
Method	M-value	B-value	M-value	B-value
mean	0.066±0.001	0.065±0.001*	0.113±0.003	0.102±0.002*
softImpute	0.084±0.007	0.055±0.001*	0.140±0.017	0.097±0.003*
impute.knn	0.055±0.002*	0.076±0.012	0.097±0.005*	0.132±0.019
imputePCA	0.055±0.001	0.055±0.001*	0.102±0.003	0.095±0.002*
SVDmiss	0.078±0.002	0.051±0.001*	0.128±0.004	**0.089±0.003***
missForest	0.058±0.001	0.057±0.001*	0.104±0.003	0.093±0.002*
methyLImp	0.065±0.001	**0.049±0.001***	0.107±0.003	0.093±0.003*

Table 114: Dataset GSE53051 (D17). Imputation performance on **MNAR:low** type missing values.

	MAE		RMSE	
Method	M-value	B-value	M-value	B-value
mean	0.037±0.001*	0.038±0.001	0.066±0.002*	0.068±0.002
softImpute	0.065±0.011	**0.028±0.001***	0.125±0.030	0.052±0.002*
impute.knn	0.044±0.013	0.035±0.005*	0.089±0.025	0.077±0.014*
imputePCA	0.028±0.001*	0.029±0.001	0.052±0.002*	0.054±0.002
SVDmiss	0.059±0.002	0.028±0.001*	0.120±0.006	**0.048±0.002***
missForest	0.030±0.001*	0.031±0.001	0.054±0.002*	0.055±0.002
methyLImp	0.039±0.001	0.029±0.001*	0.063±0.002*	0.068±0.005

Table 115: Dataset GSE53051 (D17). Imputation performance on **MNAR:mid** type missing values.

	MAE		RMSE	
Method	M-value	B-value	M-value	B-value
mean	0.083±0.001	0.079±0.001*	0.119±0.002	0.108±0.001*
softImpute	0.094±0.005	0.054±0.001*	0.136±0.011	0.081±0.002*
impute.knn	**0.048±0.001***	0.056±0.005	**0.071±0.002***	0.091±0.012
imputePCA	0.060±0.001	0.057±0.001*	0.092±0.002	0.085±0.001*
SVDmiss	0.097±0.002	0.051±0.001*	0.138±0.004	0.076±0.002*
missForest	0.066±0.001	0.062±0.001*	0.098±0.002	0.088±0.001*
methyLImp	0.078±0.001	0.051±0.001*	0.111±0.002	0.079±0.002*

Table 116: Dataset GSE53051 (D17). Imputation performance on **MNAR:high** type missing values.

	MAE		RMSE	
Method	M-value	B-value	M-value	B-value
mean	0.042±0.001*	0.043±0.001	0.070±0.002	0.067±0.001*
softImpute	0.061±0.009	0.032±0.001*	0.096±0.020	**0.050±0.001***
impute.knn	0.053±0.013*	0.128±0.031	0.107±0.029*	0.206±0.033
imputePCA	0.032±0.001*	0.033±0.001	0.054±0.002	0.052±0.001*
SVDmiss	0.059±0.002	0.034±0.001*	0.095±0.005	0.053±0.003*
missForest	0.034±0.001*	0.035±0.001	0.058±0.003	0.054±0.001*
methyLImp	0.046±0.001	**0.031±0.001***	0.070±0.002	0.056±0.003*

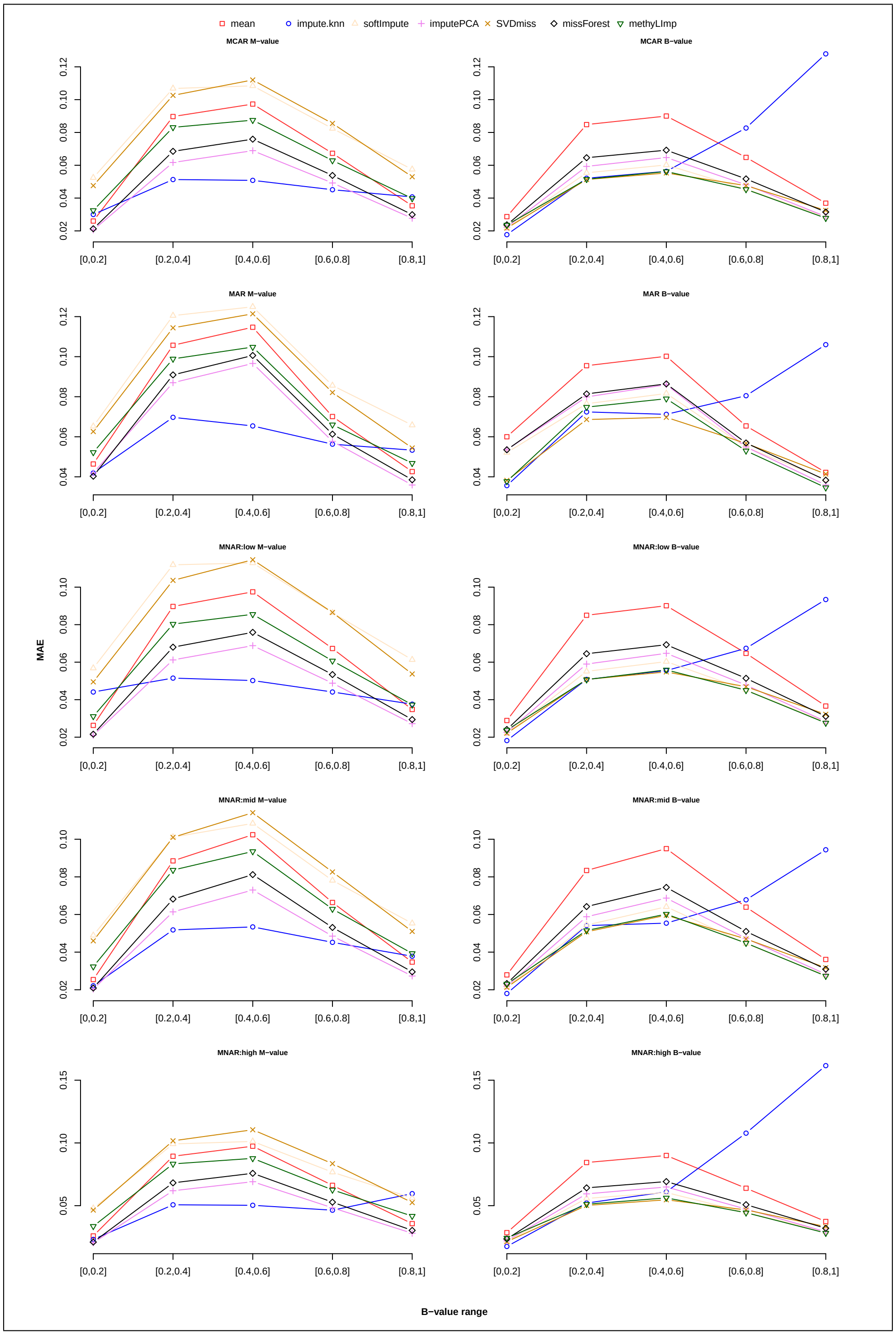

Figure 37: Dataset GSE53051 (D17). MAE imputation performances with respect to B-value range.

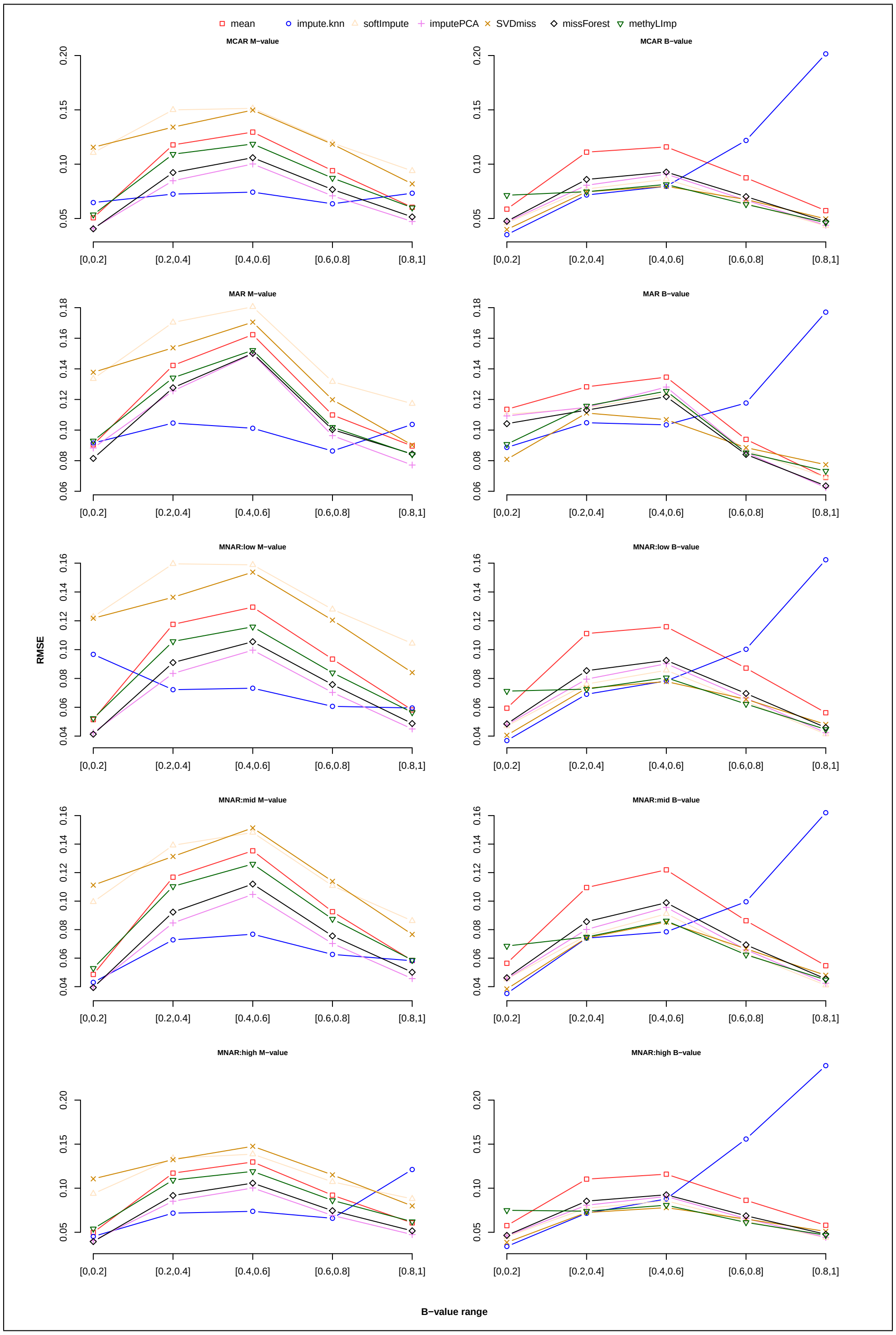

Figure 38: Dataset GSE53051 (D17). RMSE imputation performances with respect to B-value range.

2.18 GSE53051 (D18) - Lung - Cancer - 9 samples

Method	Avg time (sec)	Avg RAM (Mb)
mean	< 1	8
softImpute	< 1	45
imputePCA	7	155
impute.knn	< 1	48
SVDmiss	16	3890
methyLImp	5	130
missForest	2024	286

Table 117: Dataset GSE53051 (D18). Average time and memory usage.

Table 118: Dataset GSE53051 (D18). Imputation performance on **MCAR** type missing values.

	MAE		RMSE	
Method	M-value	B-value	M-value	B-value
mean	0.065±0.001*	0.067±0.001	0.106±0.002	0.104±0.002*
softImpute	0.078±0.002	0.073±0.004*	0.122±0.004	0.121±0.008*
impute.knn	0.072±0.005*	0.098±0.012	0.117±0.006*	0.160±0.018
imputePCA	0.061±0.001*	0.061±0.001	0.102±0.002	**0.098±0.002***
SVDmiss	0.101±0.002	0.088±0.002*	0.160±0.004*	0.165±0.005
missForest	0.062±0.001*	0.063±0.001	0.103±0.002	0.100±0.002*
methyLImp	0.070±0.001	**0.059±0.001***	0.106±0.002	0.101±0.003*

Table 119: Dataset GSE53051 (D18). Imputation performance on **MAR** type missing values.

	MAE		RMSE	
Method	M-value	B-value	M-value	B-value
mean	0.082±0.001*	0.082±0.001	0.129±0.002	0.122±0.002*
softImpute	0.095±0.002	0.091±0.006*	0.148±0.004	0.147±0.013
impute.knn	0.087±0.003*	0.103±0.008	0.136±0.004*	0.161±0.012
imputePCA	0.078±0.001	0.077±0.001*	0.126±0.002	**0.118±0.002***
SVDmiss	0.117±0.002	0.115±0.002*	0.180±0.004*	0.219±0.005
missForest	0.079±0.001	0.078±0.001*	0.127±0.002	**0.118±0.002***
methyLImp	0.085±0.001	**0.076±0.001***	0.128±0.002	0.125±0.003*

Table 120: Dataset GSE53051 (D18). Imputation performance on **MNAR:low** type missing values.

	MAE		RMSE	
Method	M-value	B-value	M-value	B-value
mean	0.045±0.001*	0.048±0.001	0.084±0.002*	0.086±0.002
softImpute	0.064±0.002	0.053±0.003*	0.115±0.005	0.102±0.007*
impute.knn	0.064±0.010	0.054±0.004*	0.115±0.015	0.106±0.008*
imputePCA	0.044±0.001*	0.046±0.001	0.084±0.002	0.083±0.002*
SVDmiss	0.084±0.002	0.070±0.002*	0.159±0.006	0.155±0.005*
missForest	**0.044±0.001***	0.047±0.001	**0.082±0.002***	0.083±0.002
methyLImp	0.053±0.001	0.044±0.001*	0.089±0.002	0.088±0.003*

Table 121: Dataset GSE53051 (D18). Imputation performance on **MNAR:mid** type missing values.

	MAE		RMSE	
Method	M-value	B-value	M-value	B-value
mean	0.105±0.001	0.100±0.001*	0.148±0.002	0.139±0.002*
softImpute	0.110±0.002	0.105±0.007*	0.153±0.003	0.155±0.016
impute.knn	**0.084±0.002***	0.095±0.005	**0.122±0.002***	0.141±0.008
imputePCA	0.098±0.001	0.093±0.001*	0.141±0.002	0.132±0.002*
SVDmiss	0.140±0.002	0.121±0.002*	0.192±0.002*	0.194±0.004
missForest	0.100±0.001	0.095±0.001*	0.144±0.002	0.134±0.002*
methyLImp	0.099±0.001	0.094±0.001*	0.137±0.002	0.136±0.002*

Table 122: Dataset GSE53051 (D18). Imputation performance on **MNAR:high** type missing values.

	MAE		RMSE	
Method	M-value	B-value	M-value	B-value
mean	0.054±0.001*	0.059±0.001	0.087±0.002*	0.089±0.001
softImpute	0.066±0.001	0.069±0.010	0.102±0.003*	0.119±0.026
impute.knn	0.078±0.008*	0.157±0.026	0.130±0.014*	0.230±0.025
imputePCA	0.050±0.001*	0.053±0.001	0.084±0.002	0.083±0.002*
SVDmiss	0.087±0.002	0.078±0.002*	0.137±0.004*	0.145±0.005
missForest	0.051±0.001*	0.055±0.001	0.084±0.002*	0.085±0.002
methyLImp	0.064±0.001	**0.048±0.001***	0.095±0.002	**0.081±0.002***

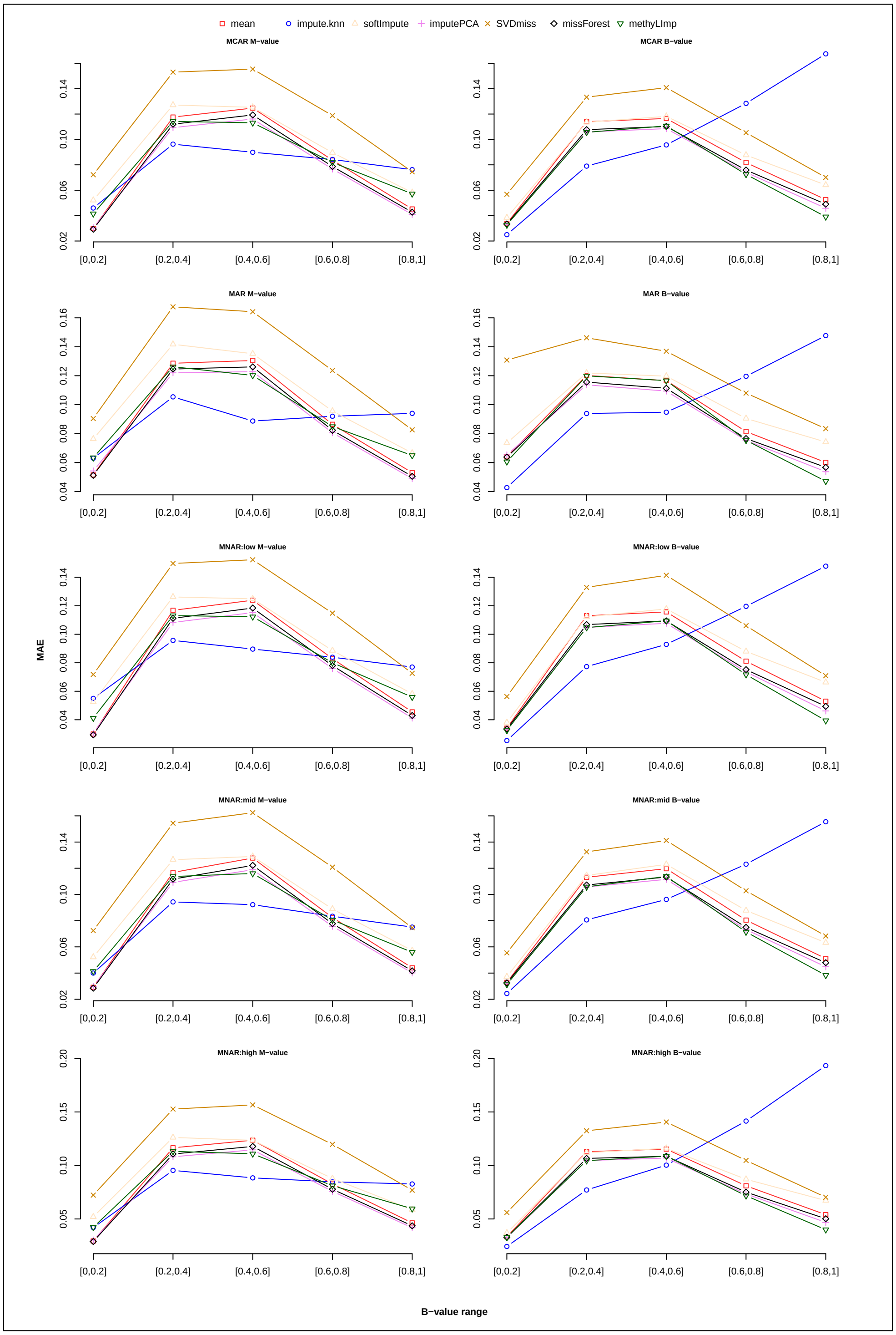

Figure 39: Dataset GSE53051 (D18). MAE imputation performances with respect to B-value range.

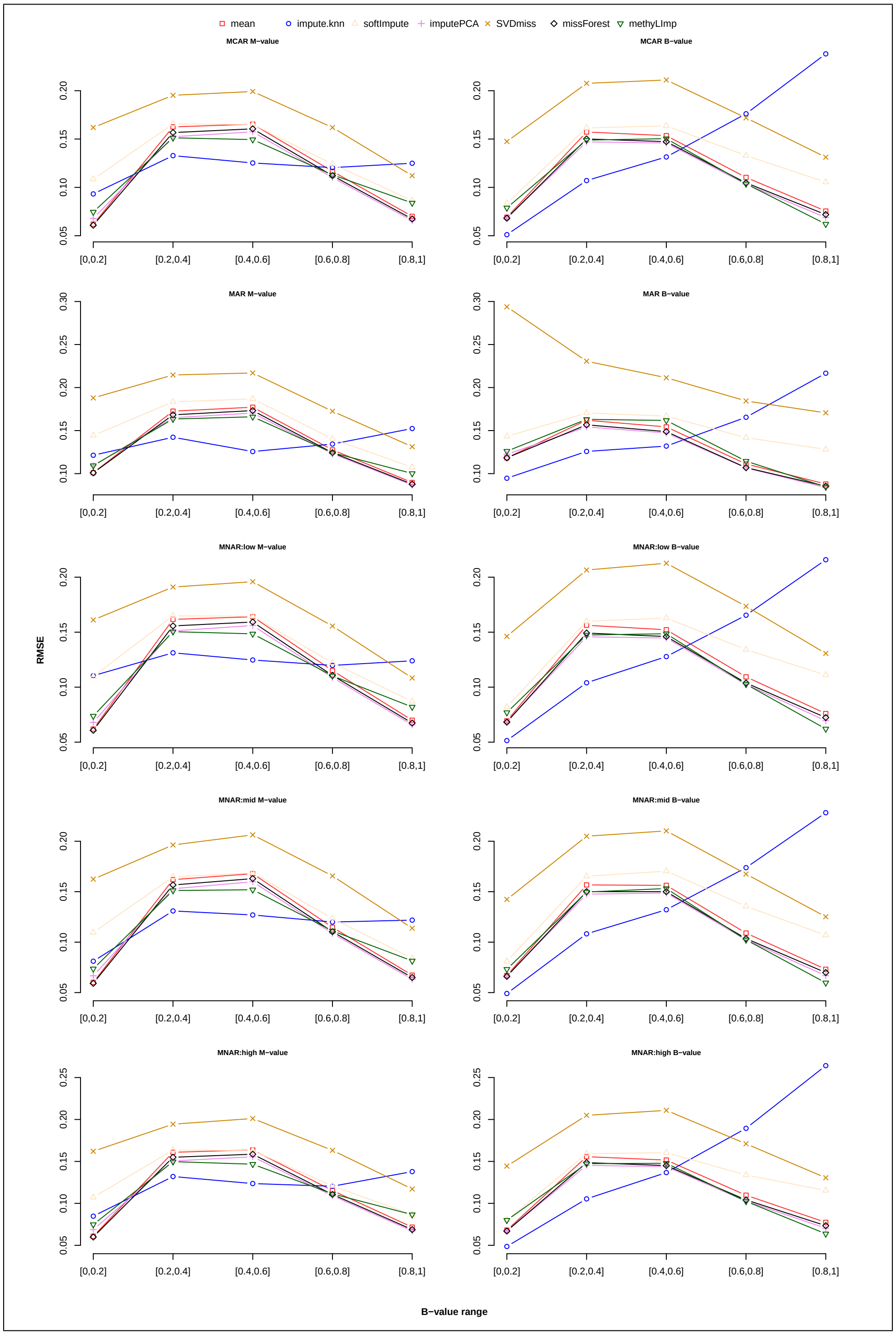

Figure 40: Dataset GSE53051 (D18). RMSE imputation performances with respect to B-value range.

2.19 GSE53051 (D19) - Pancreas - Cancer - 29 samples

Method	Avg time (sec)	Avg RAM (Mb)
mean	< 1	24
softImpute	1	77
imputePCA	10	209
impute.knn	1	80
SVDmiss	106	4705
methyLImp	243	126
missForest	31923	188

Table 123: Dataset GSE53051 (D19). Average time and memory usage.

Table 124: Dataset GSE53051 (D19). Imputation performance on **MCAR** type missing values.

	MAE		RMSE	
Method	M-value	B-value	M-value	B-value
mean	0.069±0.001*	0.070±0.001	0.113±0.001	0.109±0.001*
softImpute	0.077±0.001	0.064±0.001*	0.124±0.002	0.100±0.001*
impute.knn	0.059±0.001*	0.067±0.003	0.099±0.002*	0.110±0.006
imputePCA	0.064±0.001	0.063±0.001*	0.106±0.001	0.100±0.001*
SVDmiss	0.077±0.001	0.064±0.001*	0.128±0.002	0.106±0.001*
missForest	0.060±0.001*	0.061±0.001	0.100±0.001	**0.096±0.001***
methyLImp	0.068±0.001	**0.057±0.001***	0.108±0.001	0.099±0.002*

Table 125: Dataset GSE53051 (D19). Imputation performance on **MAR** type missing values.

	MAE		RMSE	
Method	M-value	B-value	M-value	B-value
mean	0.083±0.001	0.082±0.001*	0.134±0.001	0.123±0.001*
softImpute	0.089±0.001	0.076±0.001*	0.143±0.002	0.116±0.001*
impute.knn	0.070±0.001*	0.072±0.002	0.117±0.002	0.114±0.004*
imputePCA	0.078±0.001	0.075±0.001*	0.129±0.002	0.115±0.001*
SVDmiss	0.090±0.001	0.077±0.001*	0.148±0.002	0.125±0.002*
missForest	0.072±0.001	**0.069±0.001***	0.121±0.002	**0.106±0.001***
methyLImp	0.080±0.001	0.073±0.001*	0.130±0.002	0.126±0.002*

Table 126: Dataset GSE53051 (D19). Imputation performance on **MNAR:low** type missing values.

	MAE		RMSE	
Method	M-value	B-value	M-value	B-value
mean	0.050±0.001*	0.054±0.001	0.091±0.001*	0.094±0.001
softImpute	0.059±0.001	0.049±0.001*	0.109±0.003	0.086±0.001*
impute.knn	**0.043±0.002***	0.045±0.001	**0.081±0.004***	0.084±0.003
imputePCA	0.046±0.001*	0.048±0.001	0.087±0.001	0.086±0.001*
SVDmiss	0.061±0.001	0.050±0.001*	0.119±0.003	0.090±0.001*
missForest	**0.043±0.001***	0.047±0.001	**0.079±0.001***	0.081±0.001
methyLImp	0.050±0.001	0.044±0.001*	0.092±0.002	0.089±0.002*

Table 127: Dataset GSE53051 (D19). Imputation performance on **MNAR:mid** type missing values.

	MAE		RMSE	
Method	M-value	B-value	M-value	B-value
mean	0.108±0.001	0.100±0.001*	0.152±0.001	0.138±0.001*
softImpute	0.112±0.001	0.091±0.001*	0.159±0.002	0.127±0.001*
impute.knn	0.086±0.001	**0.083±0.001***	0.126±0.001	**0.120±0.003***
imputePCA	0.099±0.001	0.091±0.001*	0.141±0.001	0.128±0.001*
SVDmiss	0.112±0.001	0.090±0.001*	0.160±0.001	0.132±0.001*
missForest	0.092±0.001	0.085±0.001*	0.133±0.001	**0.119±0.001***
methyLImp	0.103±0.001	0.084±0.001*	0.145±0.001	0.124±0.001*

Table 128: Dataset GSE53051 (D19). Imputation performance on **MNAR:high** type missing values.

	MAE		RMSE	
Method	M-value	B-value	M-value	B-value
mean	0.059±0.001*	0.063±0.001	0.096±0.001	0.096±0.001*
softImpute	0.065±0.001	0.058±0.001*	0.105±0.002	0.090±0.001*
impute.knn	0.054±0.001*	0.073±0.007	0.090±0.002*	0.124±0.012
imputePCA	0.054±0.001*	0.057±0.001	0.091±0.001	0.089±0.001*
SVDmiss	0.066±0.001	0.060±0.001*	0.108±0.002	0.098±0.001*
missForest	0.052±0.001*	0.056±0.001	0.087±0.001	**0.086±0.001***
methyLImp	0.059±0.001	**0.050±0.001***	0.094±0.001	**0.086±0.001***

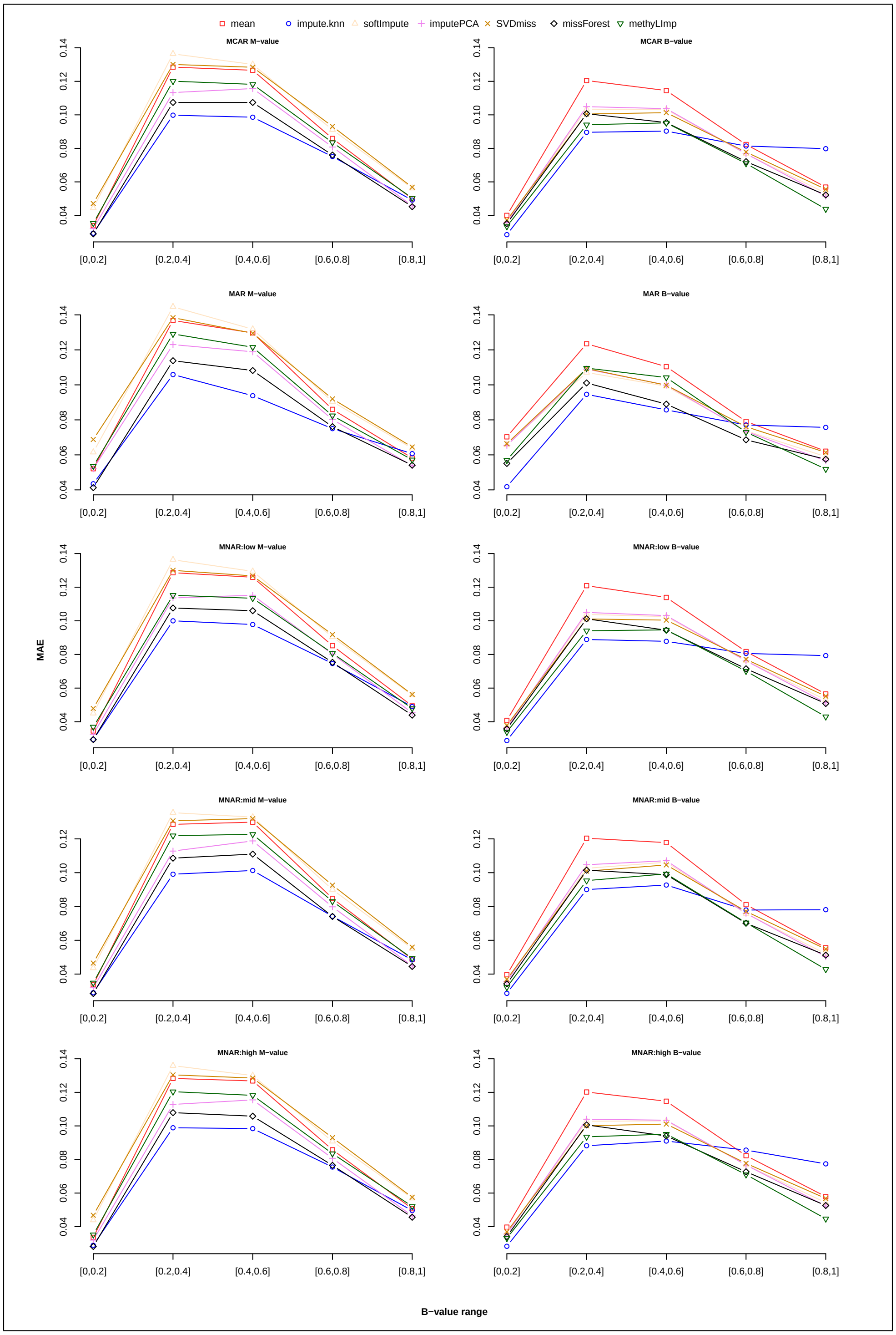

Figure 41: Dataset GSE53051 (D19). MAE imputation performances with respect to B-value range.

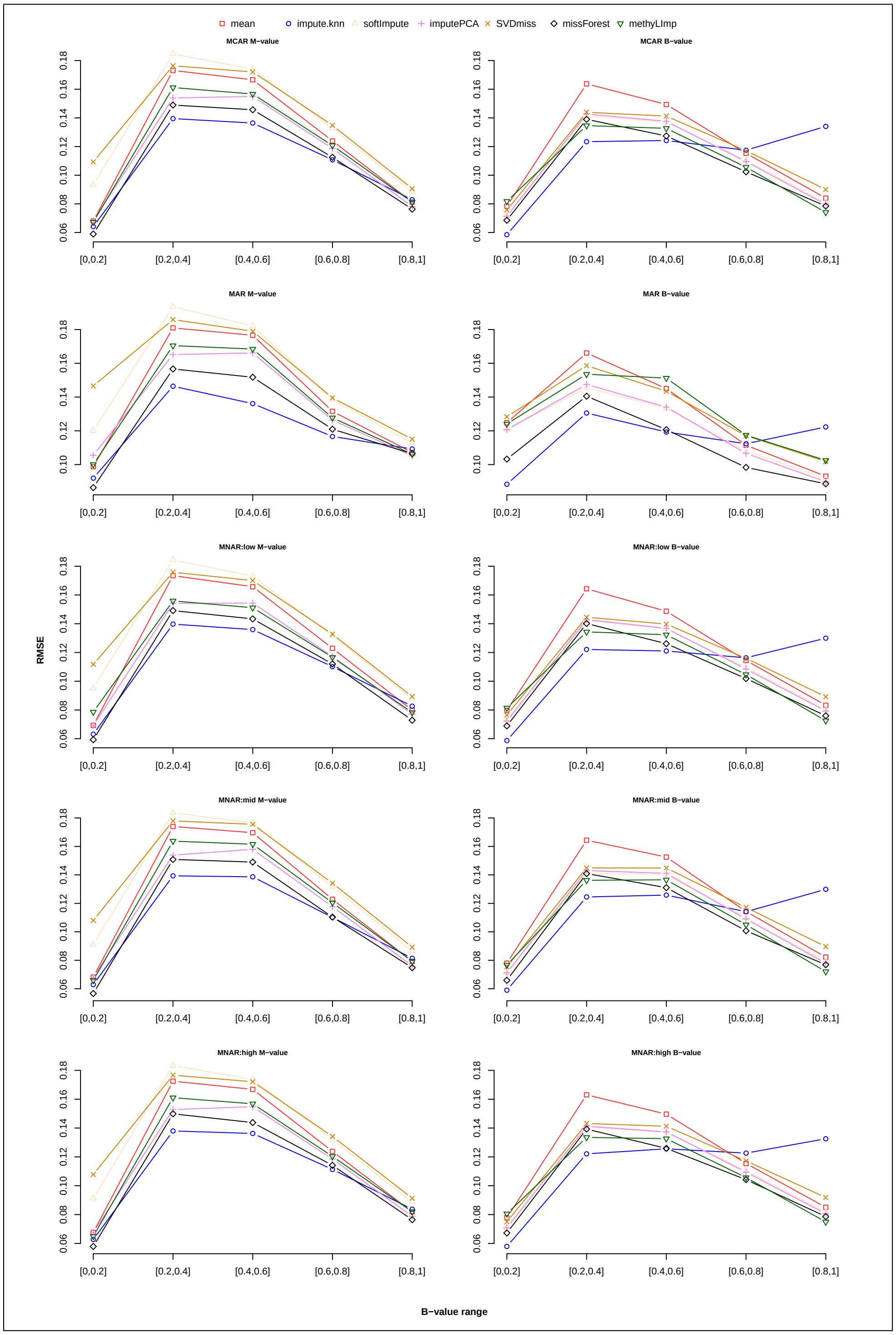

Figure 42: Dataset GSE53051 (D19). RMSE imputation performances with respect to B-value range.

2.20 GSE53051 (D20) - Thyroid - Cancer - 70 samples

Method	Avg time (sec)	Avg RAM (Mb)
mean	< 1	58
softImpute	1	128
imputePCA	20	293
impute.knn	3	150
SVDmiss	291	4800
methyLImp	3067	150
missForest	122143	399

Table 129: Dataset GSE53051 (D20). Average time and memory usage.

Table 130: Dataset GSE53051 (D20). Imputation performance on **MCAR** type missing values.

	MAE		RMSE	
Method	M-value	B-value	M-value	B-value
mean	0.054±0.001*	0.054±0.001	0.095±0.001	0.091±0.001*
softImpute	0.056±0.001	0.053±0.001*	0.096±0.001	0.089±0.001*
impute.knn	0.050±0.001*	0.053±0.001	0.090±0.001*	0.092±0.003
imputePCA	0.052±0.001	0.052±0.001*	0.093±0.001	0.089±0.001*
SVDmiss	0.057±0.001	0.051±0.001*	0.100±0.001	0.087±0.001*
missForest	**0.050±0.001***	0.051±0.001	0.089±0.001	**0.086±0.001***
methyLImp	0.074±0.002	0.052±0.001*	0.137±0.003	0.095±0.001*

Table 131: Dataset GSE53051 (D20). Imputation performance on **MAR** type missing values.

	MAE		RMSE	
Method	M-value	B-value	M-value	B-value
mean	0.069±0.001	0.067±0.001*	0.120±0.001	0.108±0.001*
softImpute	0.071±0.001	0.066±0.001*	0.121±0.001	0.108±0.001*
impute.knn	0.063±0.001*	0.064±0.001	0.111±0.001	0.109±0.005*
imputePCA	0.067±0.001	0.065±0.001*	0.119±0.001	0.107±0.001*
SVDmiss	0.070±0.001	**0.061±0.001***	0.123±0.001	0.100±0.001*
missForest	0.063±0.001	**0.061±0.001***	0.112±0.001	**0.099±0.001***
methyLImp	0.072±0.001	0.067±0.001*	0.126±0.001	0.120±0.001*

Table 132: Dataset GSE53051 (D20). Imputation performance on **MNAR:low** type missing values.

	MAE		RMSE	
Method	M-value	B-value	M-value	B-value
mean	0.040±0.001*	0.042±0.001	0.078±0.001*	0.080±0.001
softImpute	0.043±0.001	0.041±0.001*	0.081±0.001	0.078±0.001*
impute.knn	**0.037±0.001***	0.040±0.001	0.074±0.001*	0.079±0.003
imputePCA	0.039±0.001*	0.040±0.001	0.078±0.001*	0.078±0.001
SVDmiss	0.044±0.001	0.039±0.001*	0.088±0.001	0.075±0.001*
missForest	**0.037±0.001***	0.040±0.001	**0.073±0.001***	0.075±0.001
methyLImp	0.048±0.002	0.038±0.001*	0.106±0.007	0.078±0.001*

Table 133: Dataset GSE53051 (D20). Imputation performance on **MNAR:mid** type missing values.

	MAE		RMSE	
Method	M-value	B-value	M-value	B-value
mean	0.108±0.001	0.098±0.001*	0.150±0.001	0.135±0.001*
softImpute	0.108±0.001	0.095±0.001*	0.150±0.001	0.132±0.001*
impute.knn	0.097±0.001	0.092±0.001*	0.138±0.001	0.130±0.002*
imputePCA	0.103±0.001	0.094±0.001*	0.146±0.001	0.131±0.001*
SVDmiss	0.108±0.001	0.091±0.001*	0.151±0.001	0.128±0.001*
missForest	0.097±0.001	**0.089±0.001***	0.139±0.001	**0.124±0.001***
methyLImp	0.113±0.001	0.097±0.001*	0.157±0.001	0.139±0.001*

Table 134: Dataset GSE53051 (D20). Imputation performance on **MNAR:high** type missing values.

	MAE		RMSE	
Method	M-value	B-value	M-value	B-value
mean	0.045±0.001*	0.047±0.001	0.081±0.001	0.079±0.001*
softImpute	0.047±0.001	0.046±0.001*	0.083±0.001	0.077±0.001*
impute.knn	0.042±0.001*	0.046±0.002	0.077±0.001*	0.083±0.005
imputePCA	0.043±0.001*	0.045±0.001	0.079±0.001	0.076±0.001*
SVDmiss	0.047±0.001	0.044±0.001*	0.085±0.001	0.075±0.001*
missForest	**0.042±0.001***	0.044±0.001	0.077±0.001	**0.075±0.001***
methyLImp	0.051±0.001	0.042±0.001*	0.091±0.001	0.078±0.001*

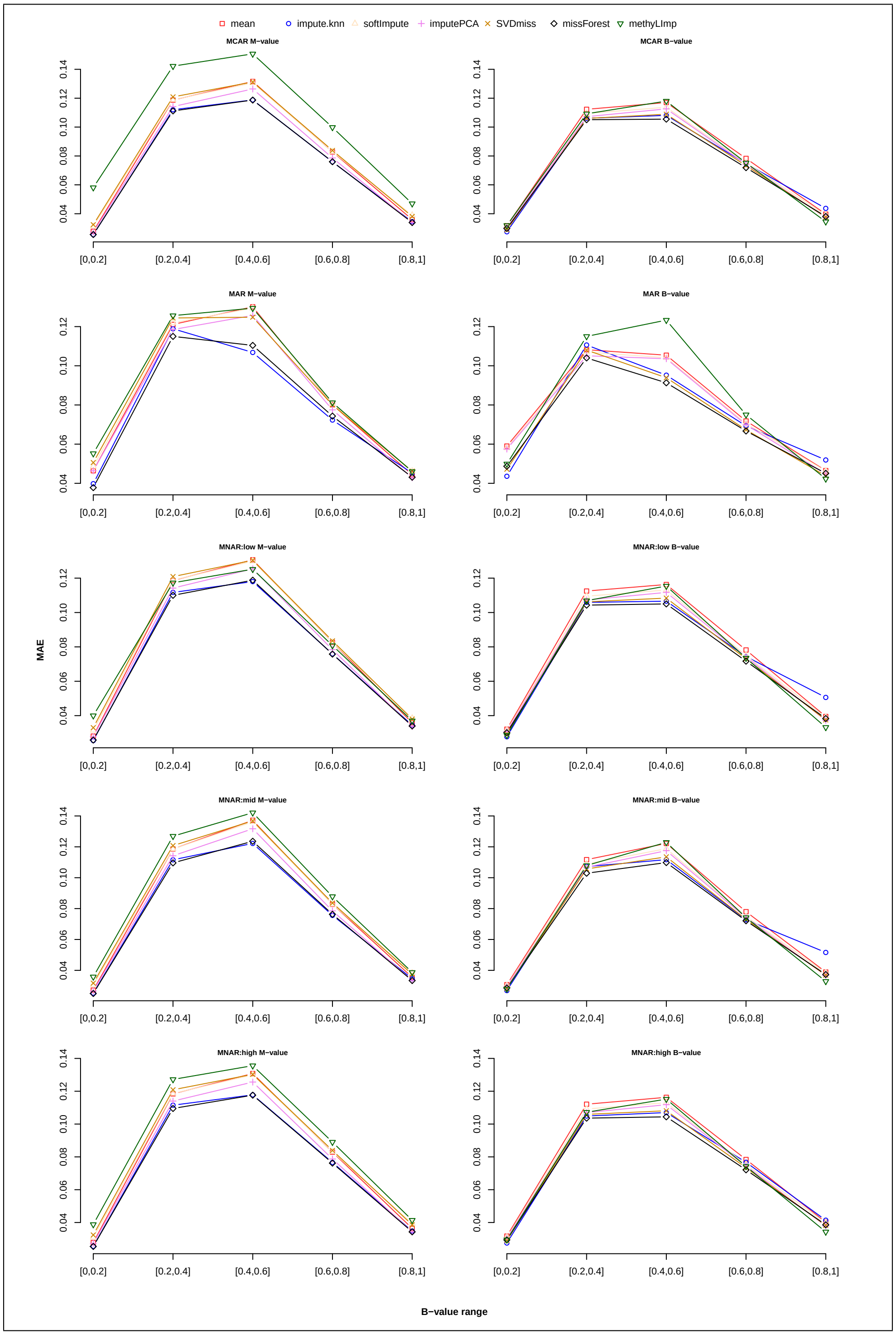

Figure 43: Dataset GSE53051 (D20). MAE imputation performances with respect to B-value range.

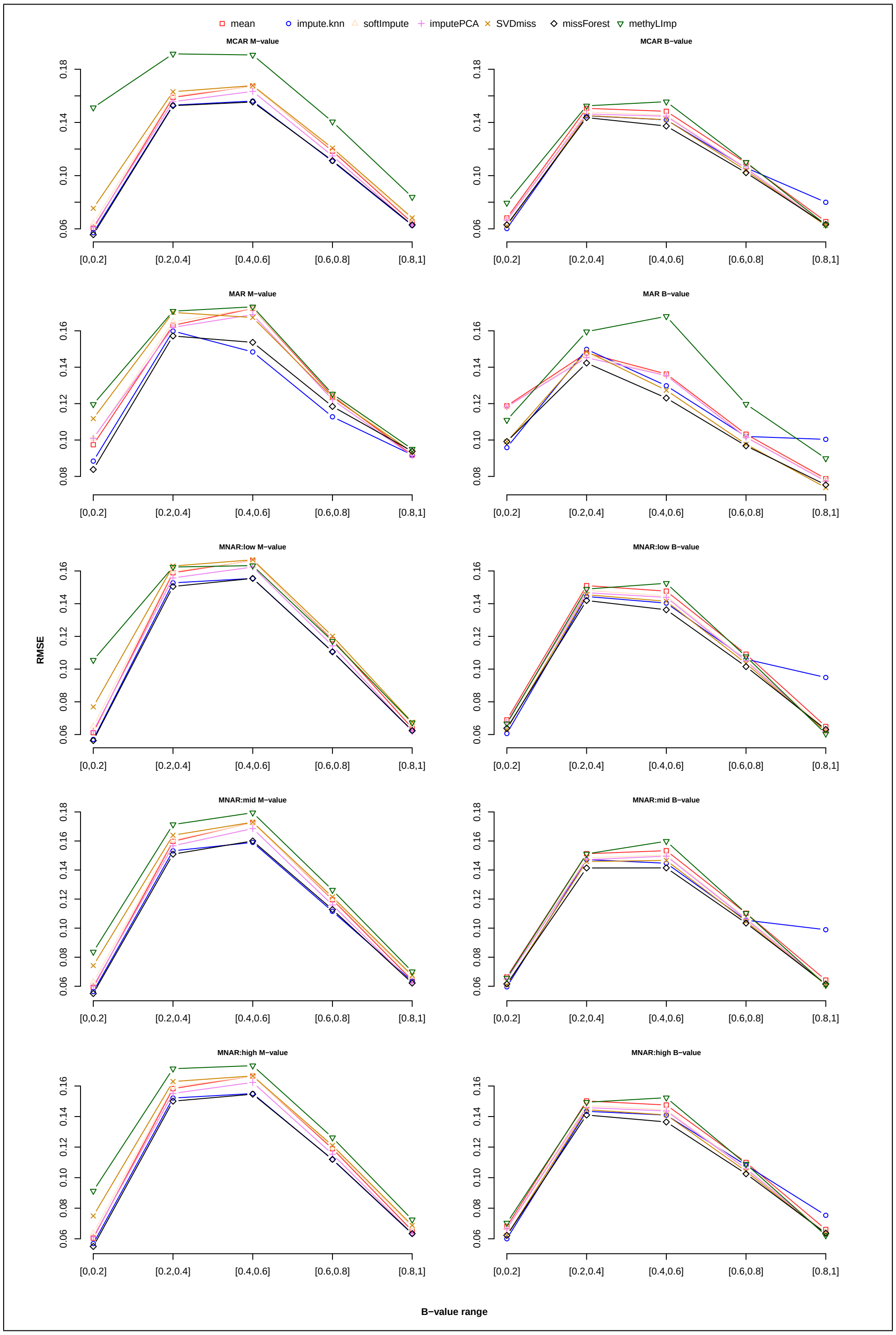

Figure 44: Dataset GSE53051 (D20). RMSE imputation performances with respect to B-value range.

2.21 GSE53162 (D21) - Brain,Cerebellum,Prefrontal Cortex - Normal - 21 samples

Method	Avg time (sec)	Avg RAM (Mb)
mean	< 1	18
softImpute	< 1	54
imputePCA	9	188
impute.knn	1	63
SVDmiss	61	4279
methyLImp	77	102
missForest	18599	144

Table 135: Dataset GSE53162 (D21). Average time and memory usage.

Table 136: Dataset GSE53162 (D21). Imputation performance on **MCAR** type missing values.

	MAE		RMSE	
Method	M-value	B-value	M-value	B-value
mean	0.042±0.001*	0.042±0.001	0.072±0.001	0.071±0.001*
softImpute	0.026±0.001*	0.026±0.001	0.043±0.001	0.043±0.001
impute.knn	0.030±0.003*	0.040±0.007	0.060±0.011*	0.084±0.016
imputePCA	0.026±0.001*	0.026±0.001	0.043±0.001*	0.043±0.001
SVDmiss	0.026±0.001*	0.026±0.001	0.043±0.001	0.043±0.001*
missForest	0.026±0.001*	0.026±0.001	0.043±0.001	0.042±0.001
methyLImp	**0.023±0.001***	0.024±0.001	0.039±0.001	**0.039±0.001***

Table 137: Dataset GSE53162 (D21). Imputation performance on **MAR** type missing values.

	MAE		RMSE	
Method	M-value	B-value	M-value	B-value
mean	0.048±0.001*	0.049±0.001	0.081±0.001	0.080±0.001*
softImpute	0.035±0.001*	0.036±0.001	0.065±0.001*	0.065±0.001
impute.knn	0.036±0.001*	0.042±0.003	**0.064±0.004***	0.079±0.008
imputePCA	0.035±0.001*	0.036±0.001	0.064±0.001	0.064±0.001*
SVDmiss	0.035±0.001*	0.036±0.001	0.064±0.001*	0.065±0.001
missForest	0.035±0.001*	0.036±0.001	0.063±0.001	**0.063±0.001***
methyLImp	**0.033±0.001***	0.034±0.001	0.063±0.001*	0.063±0.001

Table 138: Dataset GSE53162 (D21). Imputation performance on **MNAR:low** type missing values.

	MAE		RMSE	
Method	M-value	B-value	M-value	B-value
mean	0.032±0.001*	0.032±0.001	0.062±0.001*	0.063±0.001
softImpute	0.020±0.001*	0.020±0.001	0.036±0.001*	0.036±0.001
impute.knn	0.027±0.006	0.024±0.003*	0.063±0.019	0.053±0.010*
imputePCA	0.020±0.001*	0.020±0.001	0.036±0.001*	0.037±0.001
SVDmiss	0.020±0.001*	0.020±0.001	0.036±0.001*	0.037±0.001
missForest	0.020±0.001*	0.021±0.001	0.036±0.001*	0.037±0.001
methyLImp	**0.018±0.001***	0.018±0.001	**0.033±0.001***	0.033±0.001

Table 139: Dataset GSE53162 (D21). Imputation performance on **MNAR:mid** type missing values.

	MAE		RMSE	
Method	M-value	B-value	M-value	B-value
mean	0.070±0.001	0.066±0.001*	0.100±0.001	0.094±0.001*
softImpute	0.040±0.001	0.040±0.001*	0.064±0.001	0.063±0.001*
impute.knn	0.037±0.001*	0.039±0.002	0.059±0.003*	0.066±0.006
imputePCA	0.041±0.001	0.040±0.001*	0.064±0.001	0.062±0.001*
SVDmiss	0.039±0.001	0.038±0.001*	0.062±0.001	0.060±0.001*
missForest	0.040±0.001	0.038±0.001*	0.063±0.001	0.060±0.001*
methyLImp	0.036±0.001	**0.035±0.001***	0.057±0.001	**0.056±0.001***

Table 140: Dataset GSE53162 (D21). Imputation performance on **MNAR:high** type missing values.

	MAE		RMSE	
Method	M-value	B-value	M-value	B-value
mean	0.036±0.001*	0.038±0.001	0.061±0.001*	0.063±0.001
softImpute	0.023±0.001*	0.024±0.001	0.037±0.001*	0.038±0.001
impute.knn	0.027±0.004*	0.057±0.014	0.053±0.014*	0.117±0.023
imputePCA	0.023±0.001*	0.024±0.001	0.037±0.001*	0.038±0.001
SVDmiss	0.024±0.001*	0.024±0.001	0.039±0.001*	0.040±0.001
missForest	0.023±0.001*	0.024±0.001	0.037±0.001*	0.038±0.001
methyLImp	**0.022±0.001***	0.022±0.001	**0.035±0.001***	0.035±0.001

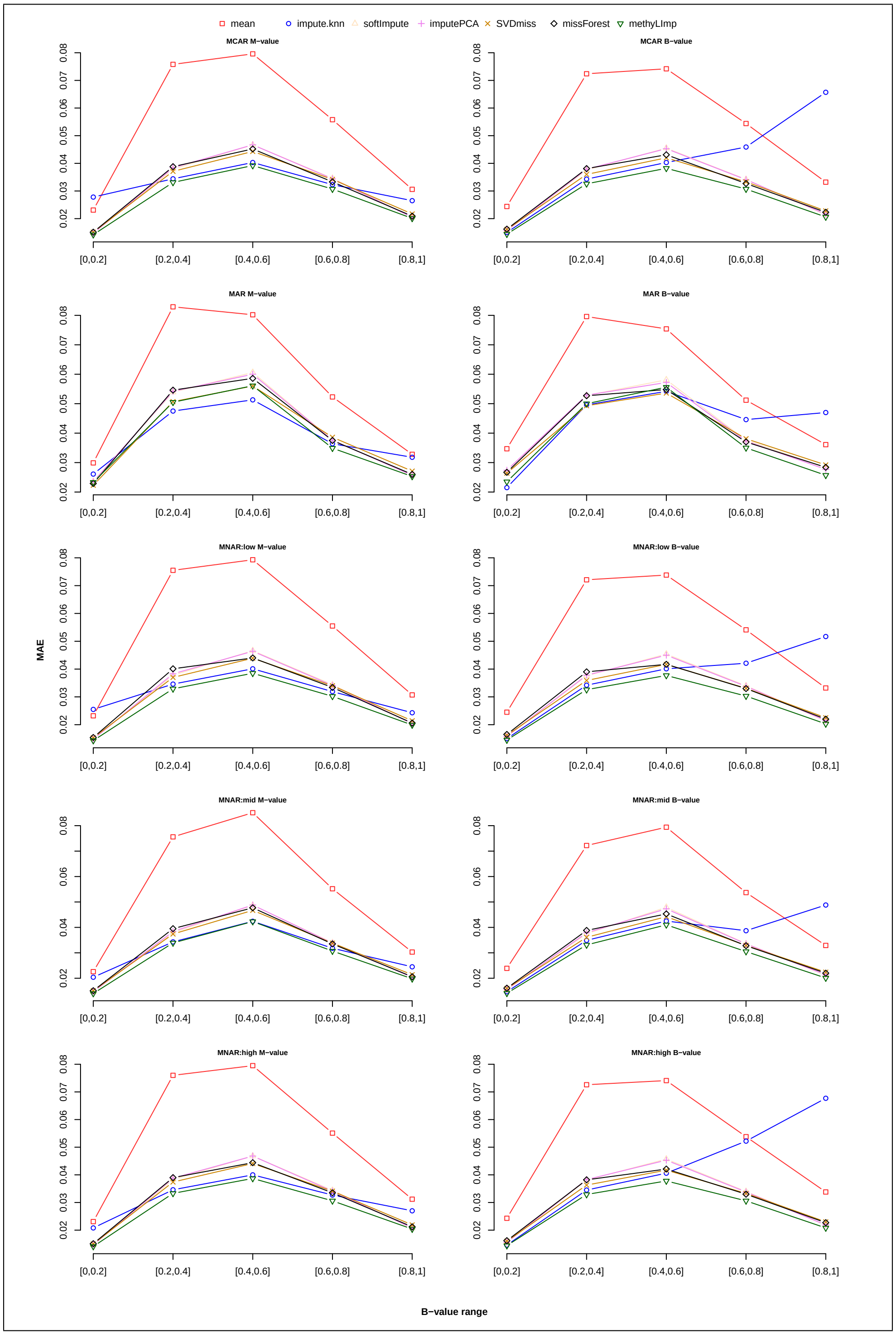

Figure 45: Dataset GSE53162 (D21). MAE imputation performances with respect to B-value range.

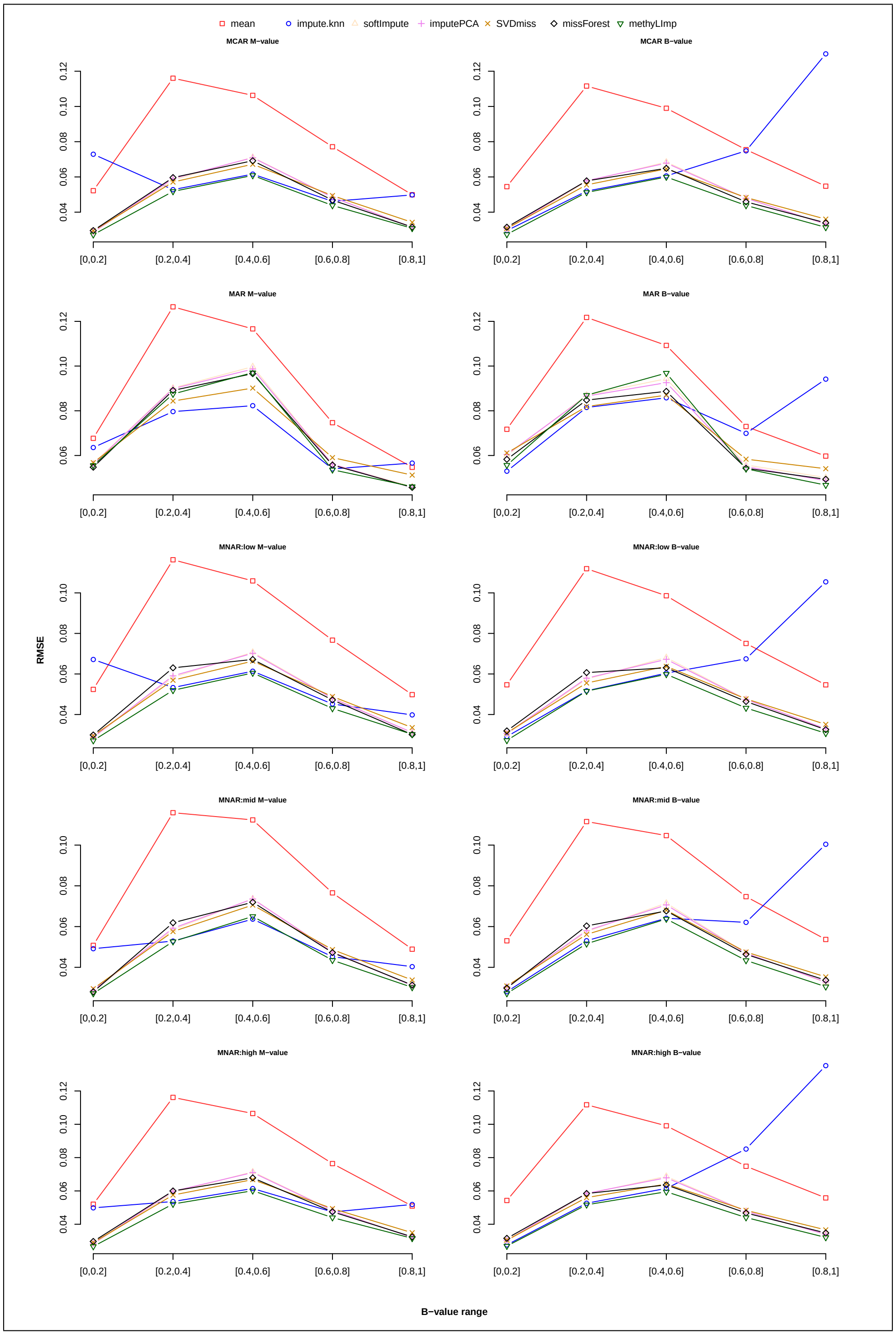

Figure 46: Dataset GSE53162 (D21). RMSE imputation performances with respect to B-value range.

2.22 GSE53740 (D22) - Blood - Normal - 165 samples

Method	Avg time (sec)	Avg RAM (Mb)
mean	< 1	134
softImpute	2	249
imputePCA	117	539
impute.knn	10	267
SVDmiss	692	5255
methyLImp	12718	253
missForest	572498	842

Table 141: Dataset GSE53740 (D22). Average time and memory usage.

Table 142: Dataset GSE53740 (D22). Imputation performance on **MCAR** type missing values.

	MAE		RMSE	
Method	M-value	B-value	M-value	B-value
mean	0.034±0.001*	0.035±0.001	0.064±0.001	0.063±0.001*
softImpute	0.031±0.001*	0.032±0.001	0.060±0.001	0.058±0.001*
impute.knn	0.029±0.001*	0.030±0.001	0.055±0.001*	0.058±0.003
imputePCA	0.031±0.001*	0.031±0.001	0.060±0.001	0.058±0.001*
SVDmiss	0.029±0.001*	0.029±0.001	0.054±0.001	0.053±0.001*
methyLImp	0.072±0.008*	0.075±0.008	0.132±0.014*	0.140±0.014
missForest	**0.028±0.001***	0.028±0.001	0.054±0.001	**0.053±0.001***

Table 143: Dataset GSE53740 (D22). Imputation performance on **MAR** type missing values.

	MAE		RMSE	
Method	M-value	B-value	M-value	B-value
mean	0.054±0.001*	0.054±0.001	0.098±0.001	0.094±0.001*
softImpute	0.050±0.001	0.049±0.002*	0.095±0.001	0.088±0.003*
impute.knn	0.040±0.001*	0.043±0.001	0.079±0.001*	0.090±0.007
imputePCA	0.050±0.001	0.050±0.001*	0.095±0.001	0.091±0.001*
SVDmiss	0.039±0.001*	0.040±0.001	0.076±0.001	0.075±0.001*
methyLImp	0.045±0.001	0.043±0.001*	0.089±0.001	0.085±0.001*
missForest	**0.038±0.001***	0.039±0.001	0.075±0.001	**0.074±0.001***

Table 144: Dataset GSE53740 (D22). Imputation performance on **MNAR:low** type missing values.

	MAE		RMSE	
Method	M-value	B-value	M-value	B-value
mean	0.027±0.001*	0.029±0.001	0.056±0.001*	0.057±0.001
softImpute	0.025±0.001*	0.027±0.001	0.053±0.001	0.052±0.001*
impute.knn	0.022±0.001*	0.024±0.001	0.047±0.001*	0.051±0.002
imputePCA	0.024±0.001*	0.026±0.001	0.052±0.001*	0.054±0.001
SVDmiss	0.022±0.001*	0.024±0.001	0.048±0.001	0.048±0.001*
methyLImp	0.025±0.001*	0.026±0.001	0.052±0.001*	0.054±0.001
missForest	**0.021±0.001***	0.022±0.001	**0.046±0.001**	**0.046±0.001**

Table 145: Dataset GSE53740 (D22). Imputation performance on **MNAR:mid** type missing values.

	MAE		RMSE	
Method	M-value	B-value	M-value	B-value
mean	0.076±0.001	0.070±0.001*	0.121±0.001	0.107±0.001*
softImpute	0.071±0.001	0.064±0.001*	0.113±0.001	0.098±0.001*
impute.knn	0.059±0.001	0.057±0.001*	0.094±0.001	0.090±0.001*
imputePCA	0.070±0.001	0.064±0.001*	0.112±0.001	0.098±0.001*
SVDmiss	0.059±0.001	0.056±0.001*	0.094±0.001	0.087±0.001*
methyLImp	0.061±0.001	0.060±0.001*	0.098±0.002	0.097±0.002*
missForest	0.058±0.001	**0.054±0.001***	0.094±0.001	**0.086±0.001***

Table 146: Dataset GSE53740 (D22). Imputation performance on **MNAR:high** type missing values.

	MAE		RMSE	
Method	M-value	B-value	M-value	B-value
mean	0.030±0.001*	0.031±0.001	0.055±0.001*	0.055±0.001
softImpute	0.027±0.001*	0.030±0.001	0.051±0.001*	0.051±0.001
impute.knn	0.025±0.001*	0.027±0.001	0.047±0.001*	0.053±0.004
imputePCA	0.027±0.001*	0.028±0.001	0.050±0.001*	0.051±0.001
SVDmiss	0.025±0.001*	0.027±0.001	0.047±0.001*	0.048±0.001
methyLImp	0.030±0.001*	0.034±0.001	0.057±0.001*	0.066±0.001
missForest	**0.024±0.001***	0.026±0.001	**0.047±0.001***	0.047±0.001

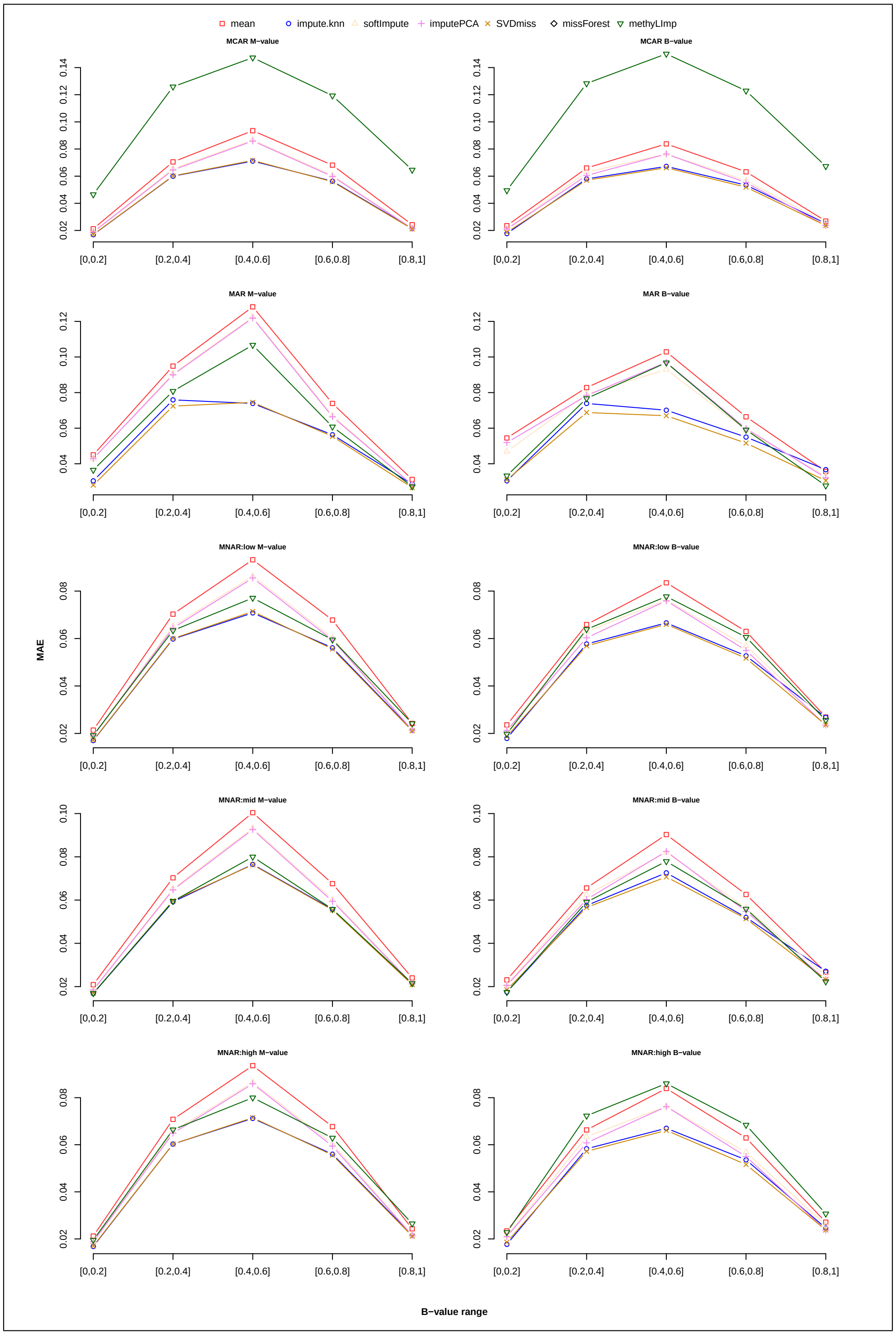

Figure 47: Dataset GSE53740 (D22). MAE imputation performances with respect to B-value range.

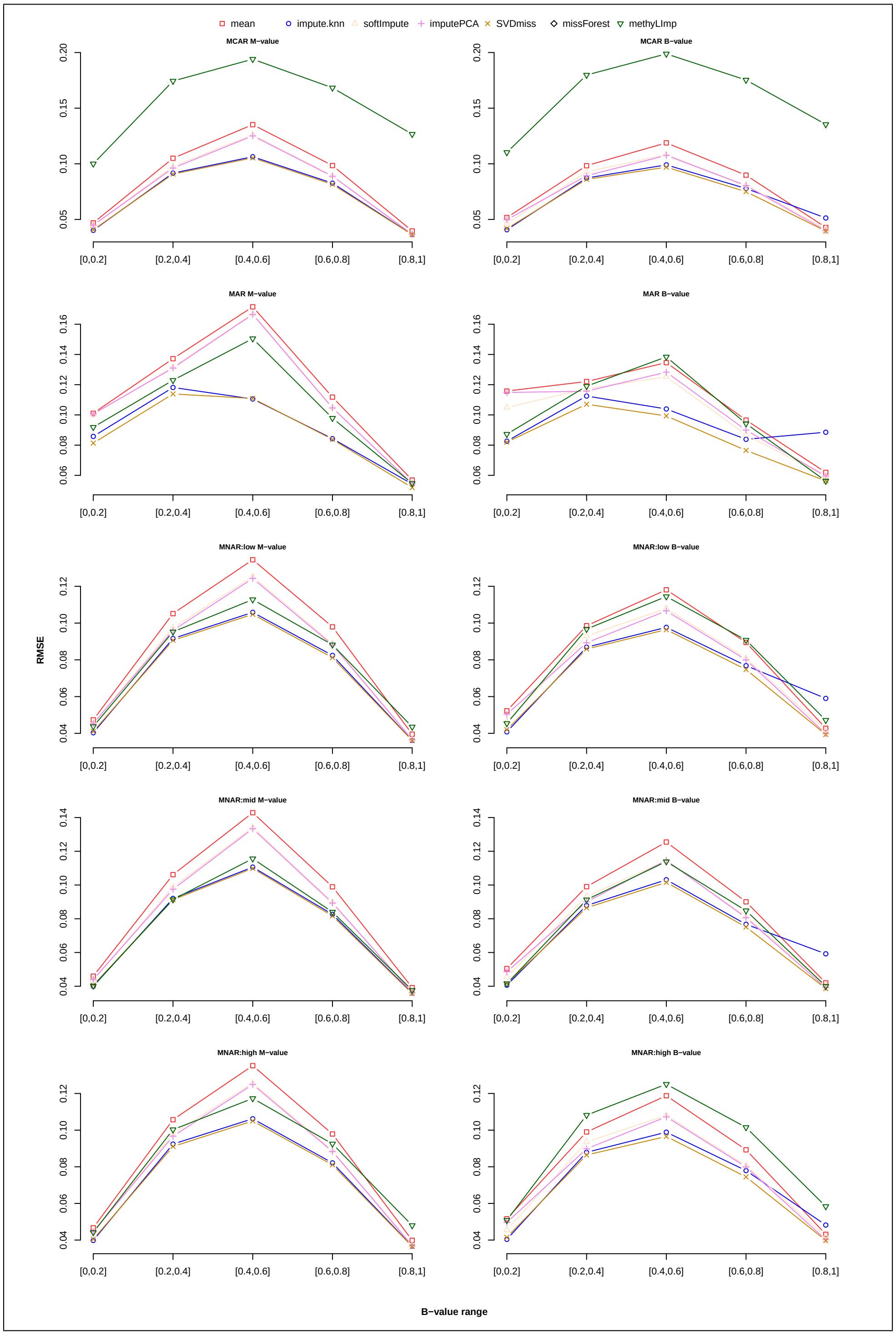

Figure 48: Dataset GSE53740 (D22). RMSE imputation performances with respect to B-value range.

2.23 GSE57360 (D23) - Brain - Normal - 5 samples

Method	Avg time (sec)	Avg RAM (Mb)
mean	< 1	4
softImpute	< 1	42
imputePCA	5	142
impute.knn	< 1	42
SVDmiss	10	3726
methyLImp	2	111
missForest	156	267

Table 147: Dataset GSE57360 (D23). Average time and memory usage.

Table 148: Dataset GSE57360 (D23). Imputation performance on **MCAR** type missing values.

	MAE		RMSE	
Method	M-value	B-value	M-value	B-value
mean	0.043±0.001	0.043±0.001*	0.070±0.002	0.068±0.002*
softImpute	0.068±0.002*	0.082±0.038	0.103±0.006*	0.156±0.088
impute.knn	0.077±0.013*	0.147±0.028	0.121±0.020*	0.227±0.030
imputePCA	0.042±0.001	0.042±0.001*	0.069±0.002	**0.067±0.002***
SVDmiss	0.064±0.002	0.056±0.001*	0.098±0.005	0.092±0.005*
missForest	0.043±0.001	0.042±0.001*	0.070±0.002	0.068±0.002*
methyLImp	0.050±0.002	**0.042±0.001***	0.075±0.002	0.068±0.003*

Table 149: Dataset GSE57360 (D23). Imputation performance on **MAR** type missing values.

	MAE		RMSE	
Method	M-value	B-value	M-value	B-value
mean	0.061±0.002	0.060±0.001*	0.106±0.003	0.100±0.003*
softImpute	0.084±0.003*	0.101±0.035	0.133±0.007*	0.187±0.072
impute.knn	0.094±0.006*	0.162±0.024	0.146±0.007*	0.234±0.025
imputePCA	0.061±0.002	0.059±0.001*	0.106±0.003	0.100±0.003*
SVDmiss	0.080±0.003	0.073±0.002*	0.128±0.005*	0.129±0.005
missForest	0.061±0.002	**0.059±0.001***	0.105±0.003	**0.099±0.003***
methyLImp	0.068±0.002	0.060±0.002*	0.109±0.003	0.106±0.004*

Table 150: Dataset GSE57360 (D23). Imputation performance on **MNAR:low** type missing values.

	MAE		RMSE	
Method	M-value	B-value	M-value	B-value
mean	0.031±0.001*	0.031±0.001	0.058±0.003*	0.059±0.003
softImpute	0.059±0.002	0.048±0.019*	0.104±0.007	0.102±0.057
impute.knn	0.099±0.030	0.079±0.016*	0.161±0.036	0.158±0.026
imputePCA	0.030±0.001*	0.031±0.001	**0.057±0.003***	0.058±0.003
SVDmiss	0.052±0.003	0.041±0.001*	0.090±0.007	0.076±0.006*
missForest	0.030±0.001*	0.031±0.001	0.057±0.003*	0.058±0.003
methyLImp	0.039±0.002	**0.030±0.001***	0.064±0.003	**0.057±0.004***

Table 151: Dataset GSE57360 (D23). Imputation performance on **MNAR:mid** type missing values.

	MAE		RMSE	
Method	M-value	B-value	M-value	B-value
mean	0.071±0.001	0.068±0.001*	0.105±0.002	0.097±0.002*
softImpute	0.087±0.003*	0.104±0.030	0.123±0.005*	0.166±0.059
impute.knn	0.068±0.002*	0.103±0.015	**0.094±0.004***	0.160±0.025
imputePCA	0.070±0.001	**0.068±0.001***	0.104±0.002	0.097±0.002*
SVDmiss	0.090±0.002	0.078±0.001*	0.126±0.003	0.116±0.004*
missForest	0.071±0.001	0.068±0.001*	0.105±0.002	0.097±0.002*
methyLImp	0.072±0.001	0.069±0.002*	0.103±0.002	0.101±0.002*

Table 152: Dataset GSE57360 (D23). Imputation performance on **MNAR:high** type missing values.

	MAE		RMSE	
Method	M-value	B-value	M-value	B-value
mean	0.039±0.001*	0.040±0.001	0.062±0.003	0.061±0.002*
softImpute	0.064±0.002	0.090±0.058	0.093±0.006*	0.172±0.128
impute.knn	0.115±0.023*	0.242±0.041	0.179±0.030*	0.310±0.032
imputePCA	**0.038±0.001***	0.038±0.001	0.061±0.003	**0.060±0.002***
SVDmiss	0.058±0.002	0.054±0.001*	0.088±0.004	0.089±0.006
missForest	0.038±0.001*	0.039±0.001	0.062±0.003	0.061±0.002*
methyLImp	0.049±0.002	0.038±0.001*	0.072±0.003	0.061±0.003*

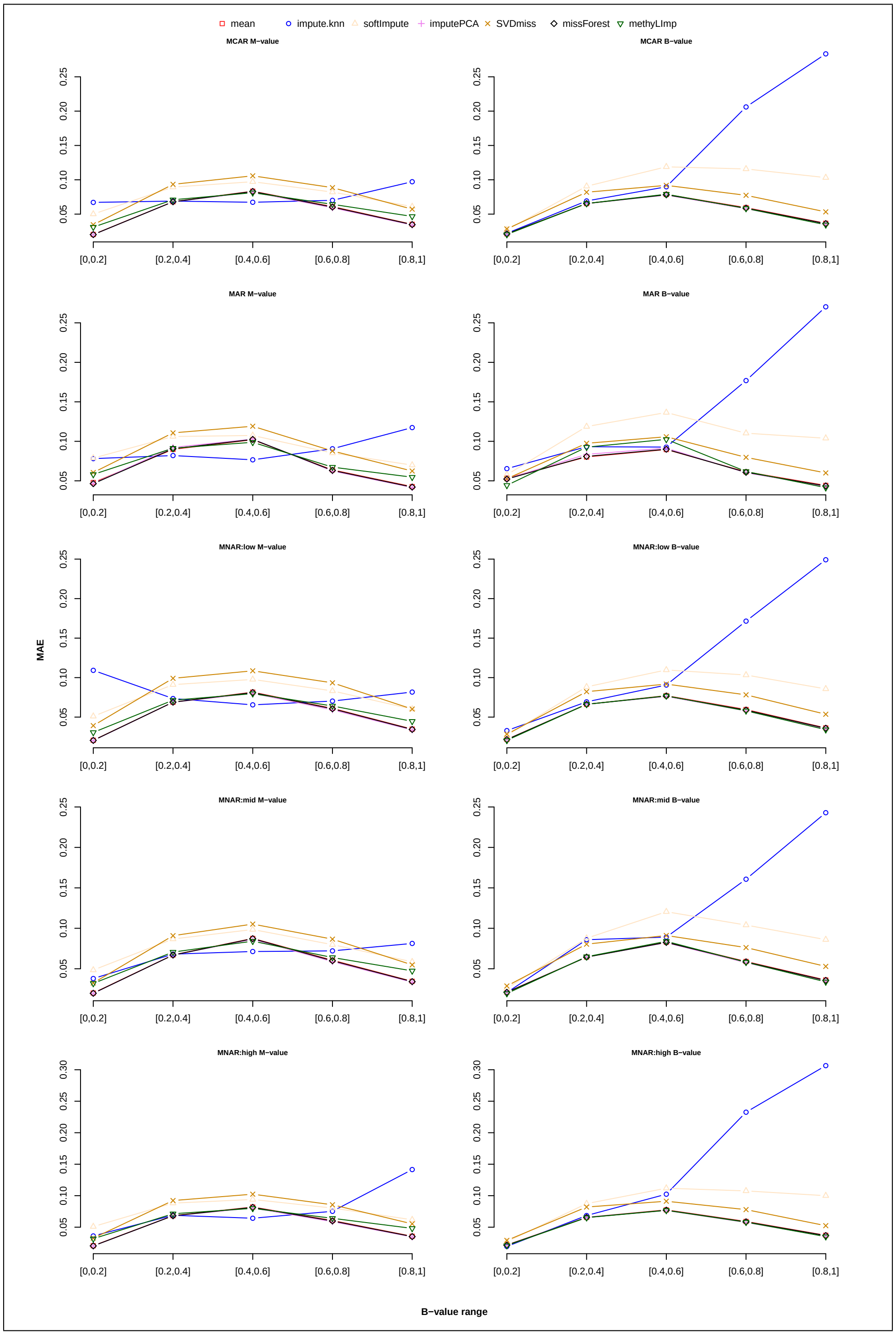

Figure 49: Dataset GSE57360 (D23). MAE imputation performances with respect to B-value range.

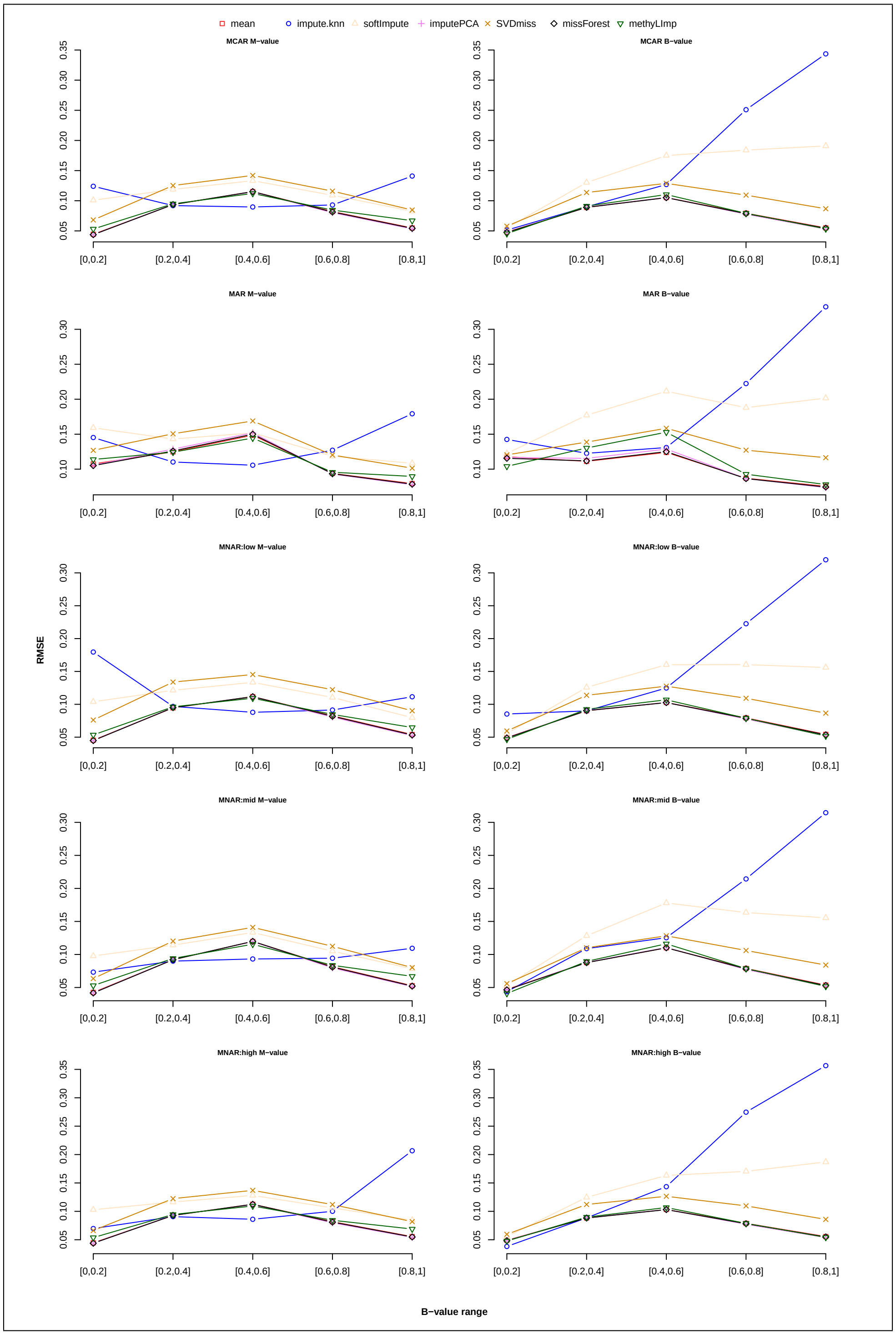

Figure 50: Dataset GSE57360 (D23). RMSE imputation performances with respect to B-value range.

2.24 GSE61151 (D24) - Blood - Normal - 184 samples

Method	Avg time (sec)	Avg RAM (Mb)
mean	< 1	148
softImpute	3	268
imputePCA	82	573
impute.knn	13	308
SVDmiss	847	5278
methyLImp	14924	275
missForest	357992	982

Table 153: Dataset GSE61151 (D24). Average time and memory usage.

Table 154: Dataset GSE61151 (D24). Imputation performance on **MCAR** type missing values.

	MAE		RMSE	
Method	M-value	B-value	M-value	B-value
mean	0.020±0.001*	0.020±0.001	0.040±0.001	0.039±0.001*
softImpute	0.020±0.001	0.020±0.001*	0.040±0.001	0.038±0.001*
impute.knn	0.020±0.001*	0.021±0.001	0.039±0.001*	0.046±0.005
imputePCA	0.019±0.001*	0.020±0.001	0.039±0.001	0.038±0.001*
SVDmiss	0.020±0.001	0.020±0.001*	0.040±0.001	0.037±0.001*
methyLImp	0.025±0.001	0.025±0.001	0.053±0.003*	0.053±0.002
missForest	**0.019±0.001***	0.019±0.001	0.038±0.001	**0.037±0.001***

Table 155: Dataset GSE61151 (D24). Imputation performance on **MAR** type missing values.

	MAE		RMSE	
Method	M-value	B-value	M-value	B-value
mean	0.028±0.001*	0.028±0.001	0.057±0.001	0.055±0.001*
softImpute	0.028±0.001	0.027±0.001*	0.057±0.001	0.055±0.001*
impute.knn	0.028±0.001*	0.030±0.002	0.060±0.002*	0.074±0.011
imputePCA	0.027±0.001*	0.027±0.001	0.057±0.001	0.054±0.001*
SVDmiss	0.028±0.001	0.027±0.001*	0.058±0.001	0.055±0.001*
methyLImp	0.030±0.001	**0.022±0.001***	0.066±0.001	**0.044±0.001***
missForest	0.026±0.001*	0.026±0.001	0.055±0.001	0.053±0.001*

Table 156: Dataset GSE61151 (D24). Imputation performance on **MNAR:low** type missing values.

	MAE		RMSE	
Method	M-value	B-value	M-value	B-value
mean	0.017±0.001*	0.017±0.001	0.034±0.001*	0.035±0.001
softImpute	0.017±0.001*	0.017±0.001	0.034±0.001	0.034±0.001*
impute.knn	0.016±0.001*	0.017±0.001	0.034±0.001*	0.041±0.004
imputePCA	0.016±0.001*	0.017±0.001	0.033±0.001*	0.034±0.001
SVDmiss	0.017±0.001*	0.017±0.001	0.035±0.001	0.034±0.001*
methyLImp	0.019±0.001*	0.019±0.001	0.050±0.001*	0.050±0.001
missForest	**0.015±0.001***	0.016±0.001	**0.032±0.001***	0.033±0.001

Table 157: Dataset GSE61151 (D24). Imputation performance on **MNAR:mid** type missing values.

	MAE		RMSE	
Method	M-value	B-value	M-value	B-value
mean	0.044±0.001	0.042±0.001*	0.069±0.001	0.065±0.001*
softImpute	0.044±0.001	0.040±0.001*	0.069±0.001	0.063±0.001*
impute.knn	0.041±0.001*	0.041±0.001	0.066±0.001*	0.068±0.003
imputePCA	0.041±0.001	0.040±0.001*	0.067±0.001	0.063±0.001*
SVDmiss	0.044±0.001	0.039±0.001*	0.069±0.001	0.062±0.001*
methyLImp	0.049±0.001	**0.037±0.001***	0.078±0.001	**0.061±0.001***
missForest	0.040±0.001	0.038±0.001*	0.065±0.001	**0.060±0.001***

Table 158: Dataset GSE61151 (D24). Imputation performance on **MNAR:high** type missing values.

	MAE		RMSE	
Method	M-value	B-value	M-value	B-value
mean	0.018±0.001*	0.018±0.001	0.035±0.001	0.035±0.001*
softImpute	0.018±0.001	0.017±0.001*	0.035±0.001	0.034±0.001*
impute.knn	0.017±0.001*	0.019±0.001	0.035±0.001*	0.043±0.006
imputePCA	0.017±0.001*	0.017±0.001	0.035±0.001	0.034±0.001*
SVDmiss	0.018±0.001	0.017±0.001*	0.036±0.001	0.033±0.001*
methyLImp	0.026±0.001	0.025±0.001*	0.058±0.002	0.056±0.002*
missForest	**0.017±0.001***	0.017±0.001	0.034±0.001	**0.033±0.001***

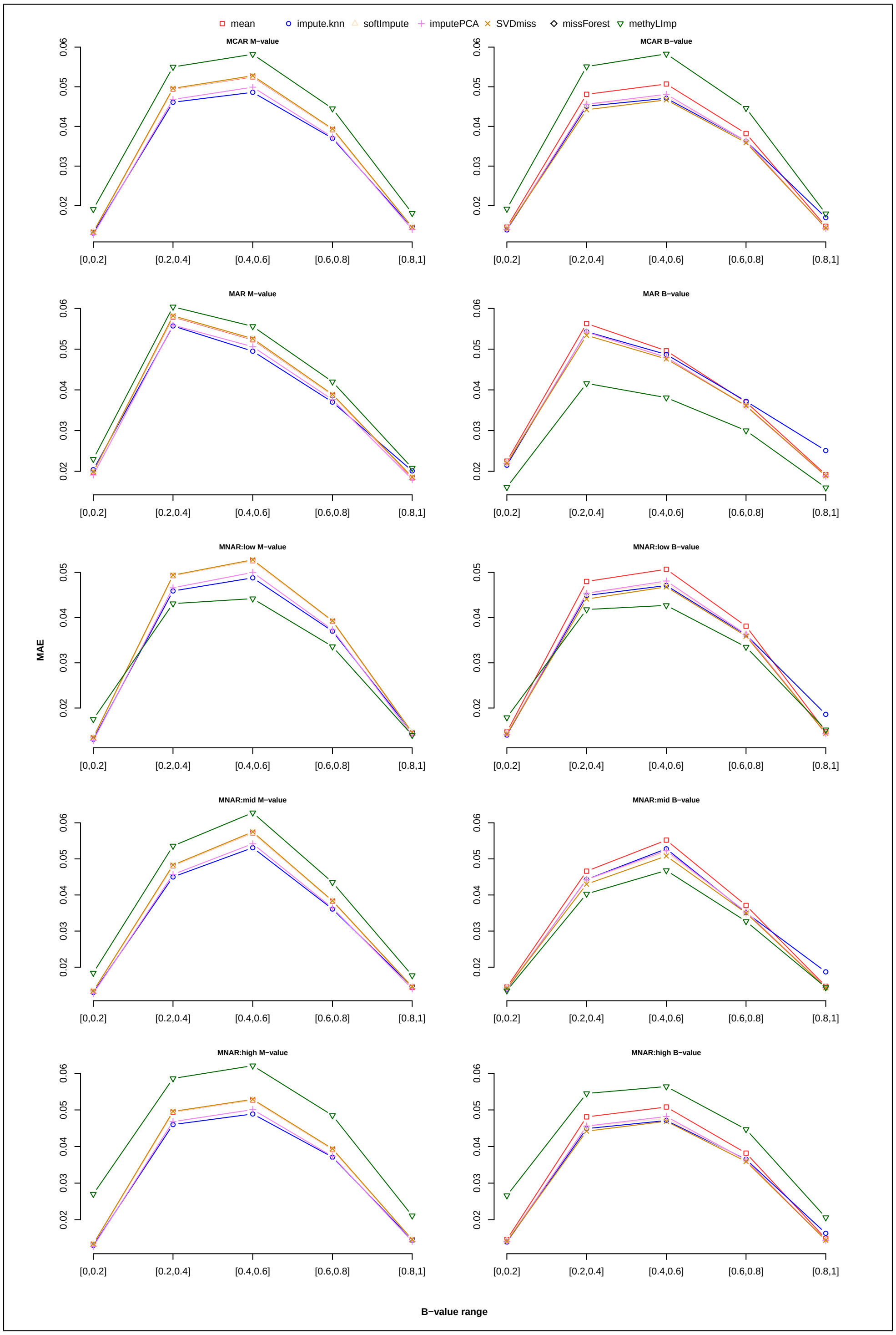

Figure 51: Dataset GSE61151 (D24). MAE imputation performances with respect to B-value range.

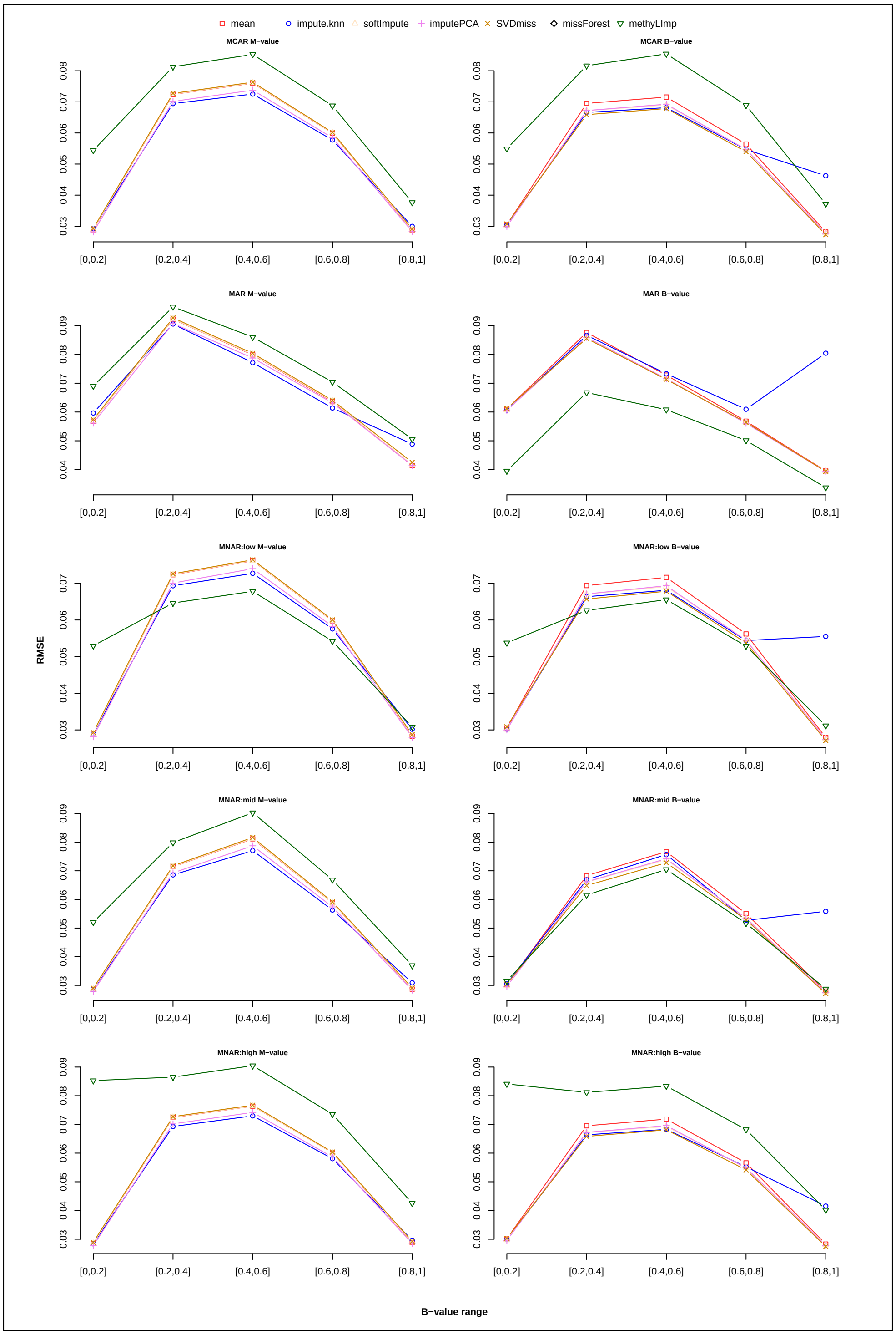

Figure 52: Dataset GSE61151 (D24). RMSE imputation performances with respect to B-value range.

2.25 GSE61257 (D25) - Adipose - Non-alcoholic fatty liver disease (NAFLD) - 8 samples

Method	Avg time (sec)	Avg RAM (Mb)
mean	< 1	7
softImpute	< 1	40
imputePCA	5	145
impute.knn	< 1	42
SVDmiss	14	3849
methyLImp	4	127
missForest	1375	260

Table 159: Dataset GSE61257 (D25). Average time and memory usage.

Table 160: Dataset GSE61257 (D25). Imputation performance on **MCAR** type missing values.

	MAE		RMSE	
Method	M-value	B-value	M-value	B-value
mean	0.026±0.001	0.026±0.001*	0.044±0.001	0.043±0.001*
softImpute	0.027±0.005	0.030±0.014*	0.047±0.013	0.057±0.038*
impute.knn	0.049±0.011*	0.071±0.019	0.104±0.021*	0.136±0.027
imputePCA	0.024±0.001	0.023±0.001*	0.041±0.002	0.040±0.002*
SVDmiss	0.032±0.001	0.030±0.001*	0.056±0.002	0.055±0.004*
missForest	0.025±0.001	0.025±0.001*	0.042±0.002	0.042±0.002*
methyLImp	0.023±0.001	**0.023±0.001***	0.040±0.002	**0.039±0.002***

Table 161: Dataset GSE61257 (D25). Imputation performance on **MAR** type missing values.

	MAE		RMSE	
Method	M-value	B-value	M-value	B-value
mean	0.037±0.001	0.036±0.001*	0.069±0.002	0.066±0.002*
softImpute	0.039±0.001	0.044±0.017	0.074±0.003	0.087±0.045
impute.knn	0.047±0.006*	0.072±0.014	0.091±0.014*	0.133±0.021
imputePCA	0.036±0.001	**0.035±0.001***	0.068±0.002	0.066±0.002*
SVDmiss	0.045±0.001	0.044±0.001*	0.085±0.003*	0.093±0.005
missForest	0.036±0.001	0.035±0.001*	0.068±0.002	**0.066±0.002***
methyLImp	0.035±0.001*	0.036±0.001	0.068±0.002*	0.070±0.002

Table 162: Dataset GSE61257 (D25). Imputation performance on **MNAR:low** type missing values.

	MAE		RMSE	
Method	M-value	B-value	M-value	B-value
mean	0.019±0.001*	0.019±0.001	0.039±0.002	0.039±0.002
softImpute	0.020±0.004	0.019±0.003*	0.041±0.010	0.040±0.009*
impute.knn	0.061±0.021	0.041±0.012*	0.127±0.033	0.099±0.024*
imputePCA	0.018±0.001*	0.018±0.001	0.036±0.002	0.036±0.002*
SVDmiss	0.024±0.001	0.023±0.001*	0.047±0.002	0.048±0.005
missForest	0.018±0.001*	0.018±0.001	0.037±0.002	0.037±0.002
methyLImp	**0.017±0.001***	0.017±0.001	**0.035±0.002***	0.035±0.002

Table 163: Dataset GSE61257 (D25). Imputation performance on **MNAR:mid** type missing values.

	MAE		RMSE	
Method	M-value	B-value	M-value	B-value
mean	0.042±0.001	0.040±0.001*	0.061±0.001	0.058±0.001*
softImpute	0.040±0.001	0.043±0.017*	0.060±0.002	0.072±0.042*
impute.knn	0.036±0.003*	0.053±0.008	0.058±0.008*	0.097±0.017
imputePCA	0.036±0.001	0.035±0.001*	0.055±0.001	0.052±0.001*
SVDmiss	0.044±0.001	0.041±0.001*	0.070±0.001	0.069±0.003*
missForest	0.039±0.001	0.038±0.001*	0.058±0.001	0.055±0.001*
methyLImp	0.035±0.001	**0.034±0.001***	0.052±0.001	**0.052±0.001***

Table 164: Dataset GSE61257 (D25). Imputation performance on **MNAR:high** type missing values.

	MAE		RMSE	
Method	M-value	B-value	M-value	B-value
mean	0.022±0.001*	0.022±0.001	0.037±0.002*	0.037±0.001
softImpute	0.024±0.001*	0.035±0.024	0.042±0.003*	0.071±0.067
impute.knn	0.083±0.024*	0.137±0.036	0.165±0.034*	0.218±0.035
imputePCA	0.021±0.001*	0.021±0.001	0.035±0.002*	0.035±0.002
SVDmiss	0.030±0.001	0.029±0.001*	0.053±0.003	0.053±0.005
missForest	0.021±0.001*	0.021±0.001	0.036±0.002*	0.036±0.002
methyLImp	0.021±0.001	**0.020±0.001***	**0.035±0.002**	**0.035±0.002**

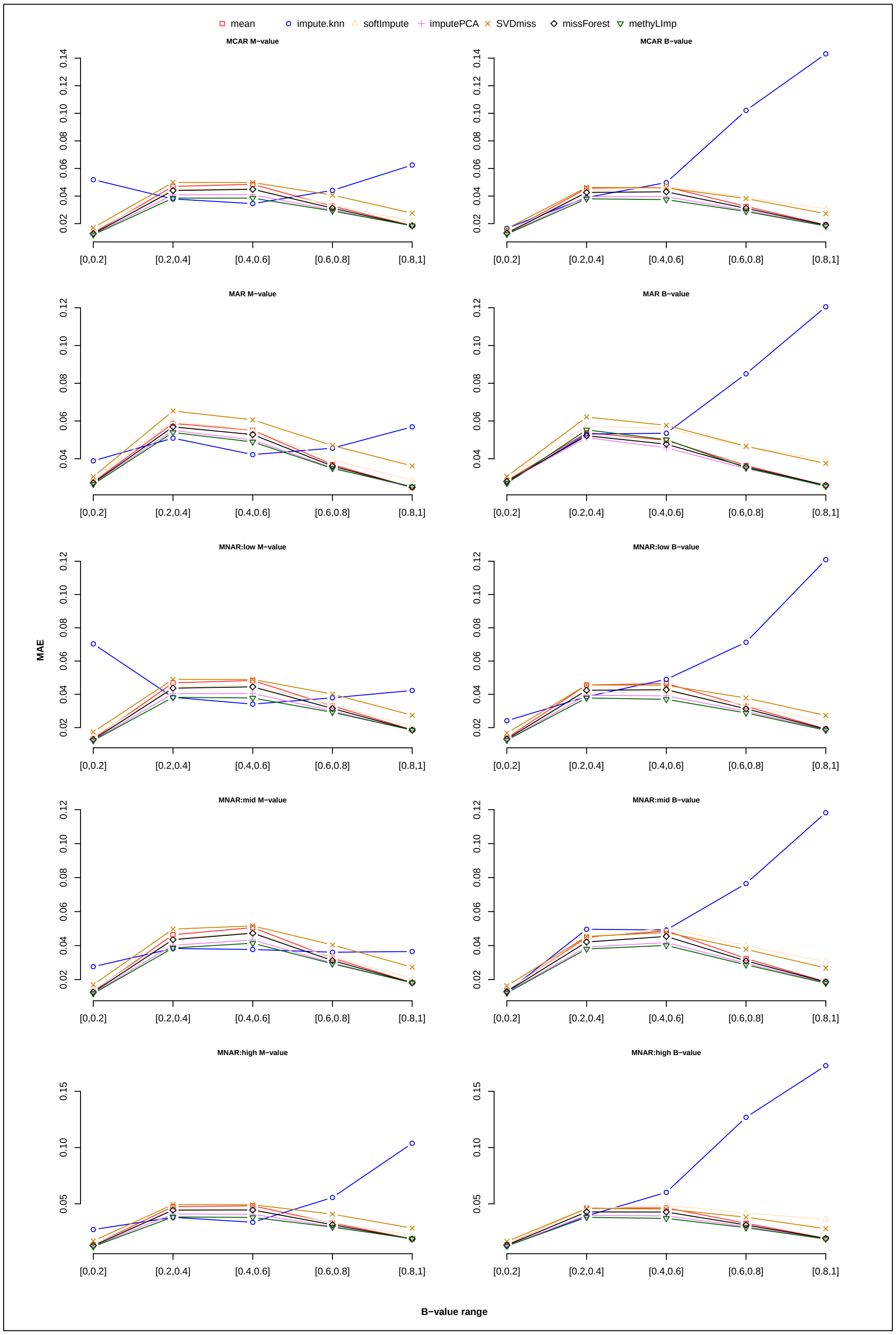

Figure 53: Dataset GSE61257 (D25). MAE imputation performances with respect to B-value range.

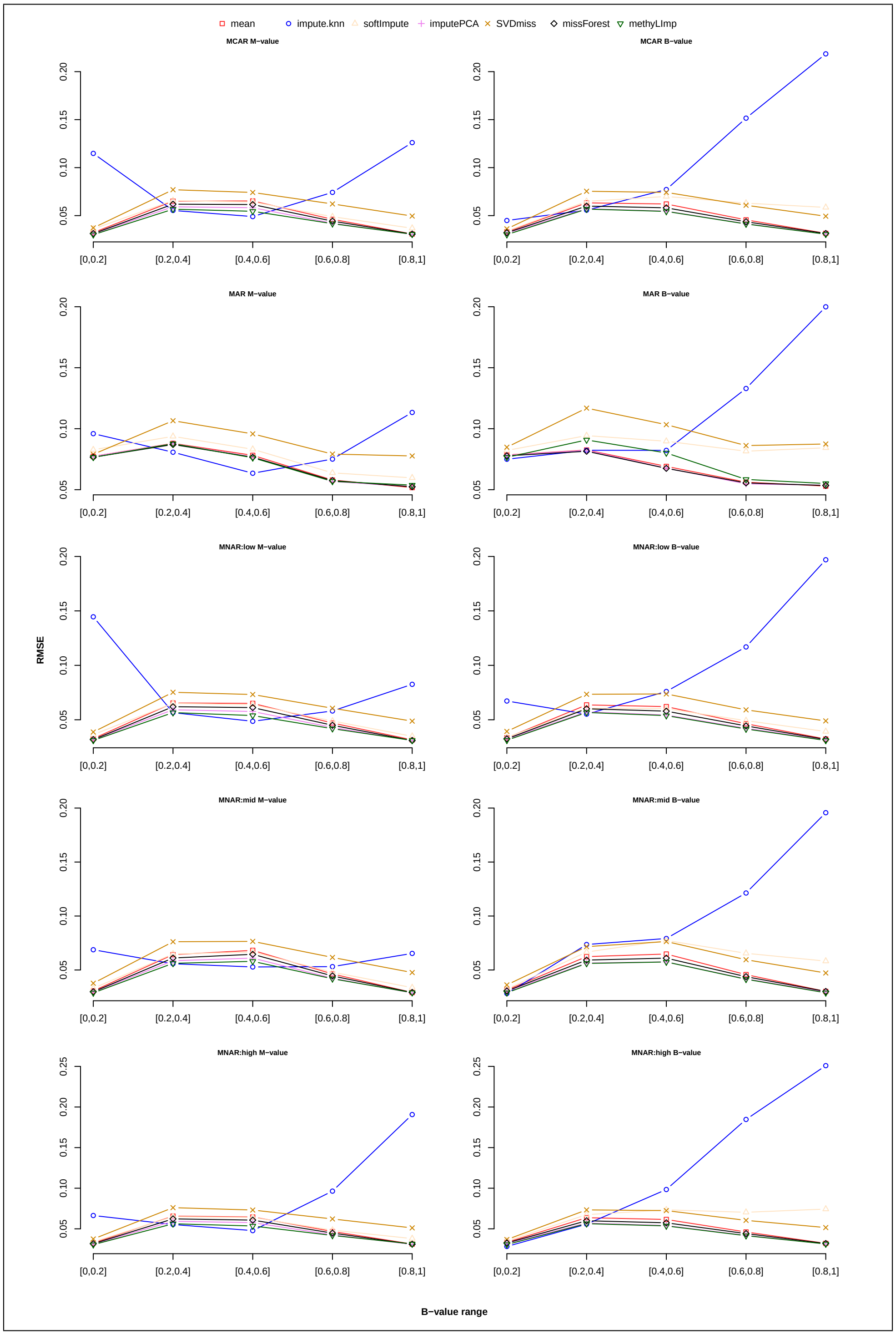

Figure 54: Dataset GSE61257 (D25). RMSE imputation performances with respect to B-value range.

2.26 GSE61257 (D26) - Adipose - Non-alcoholic steatohepatitis (NASH) - 9 samples

Method	Avg time (sec)	Avg RAM (Mb)
mean	< 1	8
softImpute	< 1	40
imputePCA	5	155
impute.knn	< 1	45
SVDmiss	16	3890
methyLImp	5	126
missForest	1952	285

Table 165: Dataset GSE61257 (D26). Average time and memory usage.

Table 166: Dataset GSE61257 (D26). Imputation performance on **MCAR** type missing values.

	MAE		RMSE	
Method	M-value	B-value	M-value	B-value
mean	0.026±0.001	0.026±0.001*	0.046±0.001	0.045±0.001*
softImpute	0.025±0.001	0.023±0.001*	0.043±0.002	0.041±0.002*
impute.knn	0.042±0.011*	0.063±0.017	0.093±0.023*	0.126±0.026
imputePCA	0.023±0.001	0.023±0.001*	0.041±0.002	0.040±0.001*
SVDmiss	0.024±0.001	0.024±0.001*	0.043±0.002*	0.043±0.002
missForest	0.024±0.001	0.024±0.001*	0.043±0.001	0.042±0.001*
methyLImp	0.021±0.001	**0.021±0.001***	0.037±0.002	**0.037±0.002***

Table 167: Dataset GSE61257 (D26). Imputation performance on **MAR** type missing values.

	MAE		RMSE	
Method	M-value	B-value	M-value	B-value
mean	0.040±0.001	0.038±0.001*	0.074±0.002	0.071±0.002*
softImpute	0.039±0.001	0.039±0.006*	0.075±0.002	0.076±0.017*
impute.knn	0.046±0.006*	0.065±0.012	0.092±0.014*	0.124±0.019
imputePCA	0.038±0.001	0.037±0.001*	0.074±0.002	0.070±0.002*
SVDmiss	0.036±0.001*	0.037±0.001	0.073±0.003*	0.076±0.004
missForest	0.040±0.001	0.038±0.001*	0.074±0.002	0.070±0.002*
methyLImp	**0.035±0.001***	0.036±0.001	**0.068±0.002***	0.070±0.002

Table 168: Dataset GSE61257 (D26). Imputation performance on **MNAR:low** type missing values.

	MAE		RMSE	
Method	M-value	B-value	M-value	B-value
mean	0.019±0.001*	0.019±0.001	0.040±0.002*	0.040±0.002
softImpute	0.018±0.001	0.018±0.001*	0.036±0.002*	0.037±0.002
impute.knn	0.053±0.018	0.038±0.012*	0.117±0.031	0.095±0.024*
imputePCA	0.017±0.001*	0.017±0.001	0.037±0.002*	0.037±0.002
SVDmiss	0.018±0.001*	0.019±0.001	0.036±0.002*	0.037±0.003
missForest	0.018±0.001*	0.018±0.001	0.037±0.002*	0.038±0.002
methyLImp	**0.016±0.001**	**0.016±0.001**	**0.032±0.002***	0.032±0.002

Table 169: Dataset GSE61257 (D26). Imputation performance on **MNAR:mid** type missing values.

	MAE		RMSE	
Method	M-value	B-value	M-value	B-value
mean	0.043±0.001	0.041±0.001*	0.066±0.001	0.061±0.001*
softImpute	0.040±0.001	0.036±0.001*	0.062±0.002	0.056±0.002*
impute.knn	0.034±0.002*	0.045±0.006	0.056±0.007*	0.083±0.014
imputePCA	0.037±0.001	0.035±0.001*	0.059±0.001	0.054±0.001*
SVDmiss	0.035±0.001	0.034±0.001*	0.056±0.002*	0.057±0.003
missForest	0.040±0.001	0.038±0.001*	0.063±0.001	0.057±0.001*
methyLImp	0.033±0.001	**0.033±0.001***	0.052±0.001	**0.051±0.001***

Table 170: Dataset GSE61257 (D26). Imputation performance on **MNAR:high** type missing values.

	MAE		RMSE	
Method	M-value	B-value	M-value	B-value
mean	0.021±0.001*	0.022±0.001	0.038±0.002*	0.038±0.002
softImpute	0.021±0.001	0.026±0.019	0.037±0.002*	0.053±0.055
impute.knn	0.074±0.024*	0.118±0.034	0.155±0.035*	0.200±0.035
imputePCA	0.019±0.001*	0.020±0.001	0.035±0.002	0.035±0.002*
SVDmiss	0.022±0.001*	0.022±0.001	0.040±0.003*	0.041±0.003
missForest	0.020±0.001*	0.021±0.001	0.036±0.002	0.036±0.002
methyLImp	**0.019±0.001***	0.019±0.001	**0.033±0.002***	0.034±0.002

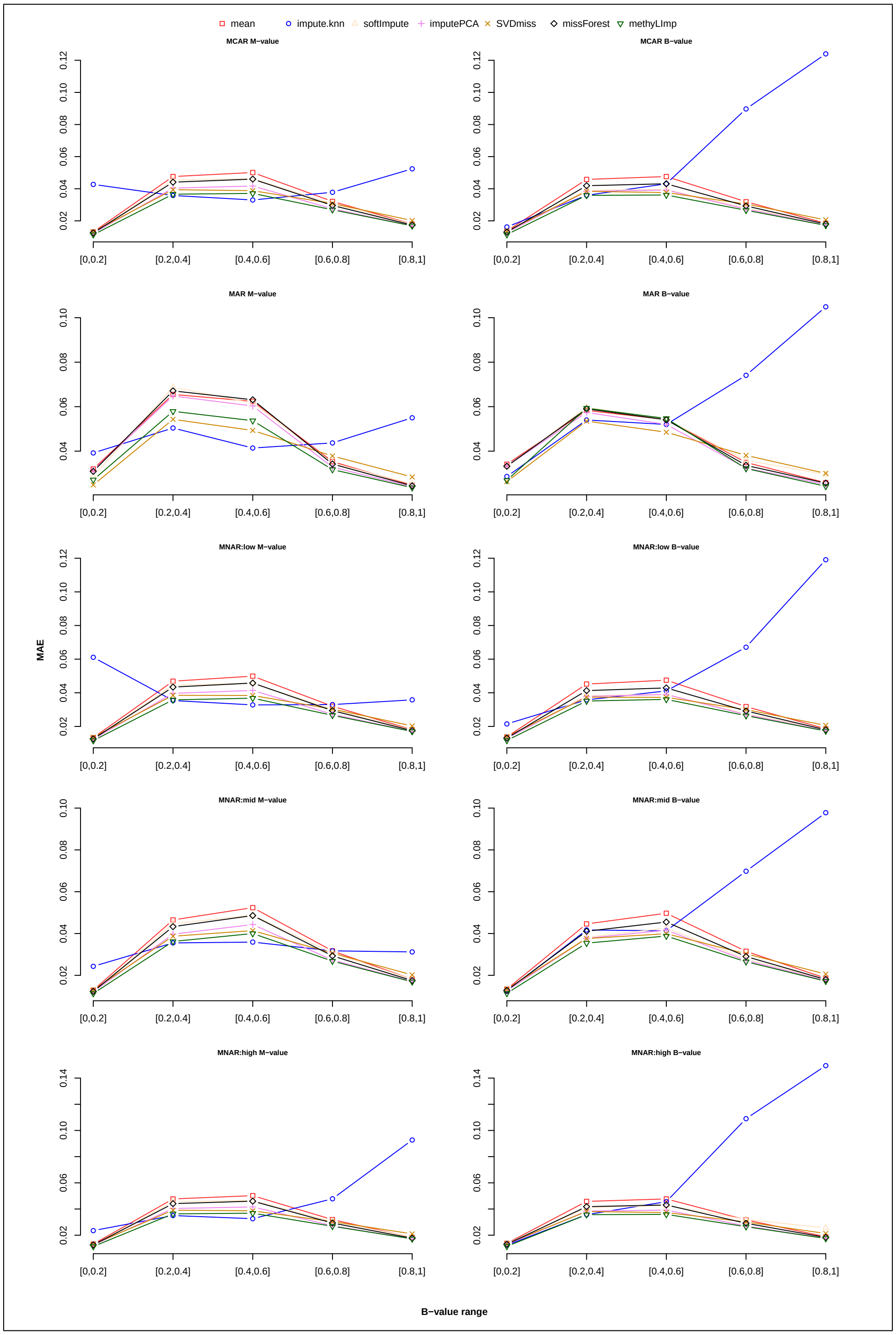

Figure 55: Dataset GSE61257 (D26). MAE imputation performances with respect to B-value range.

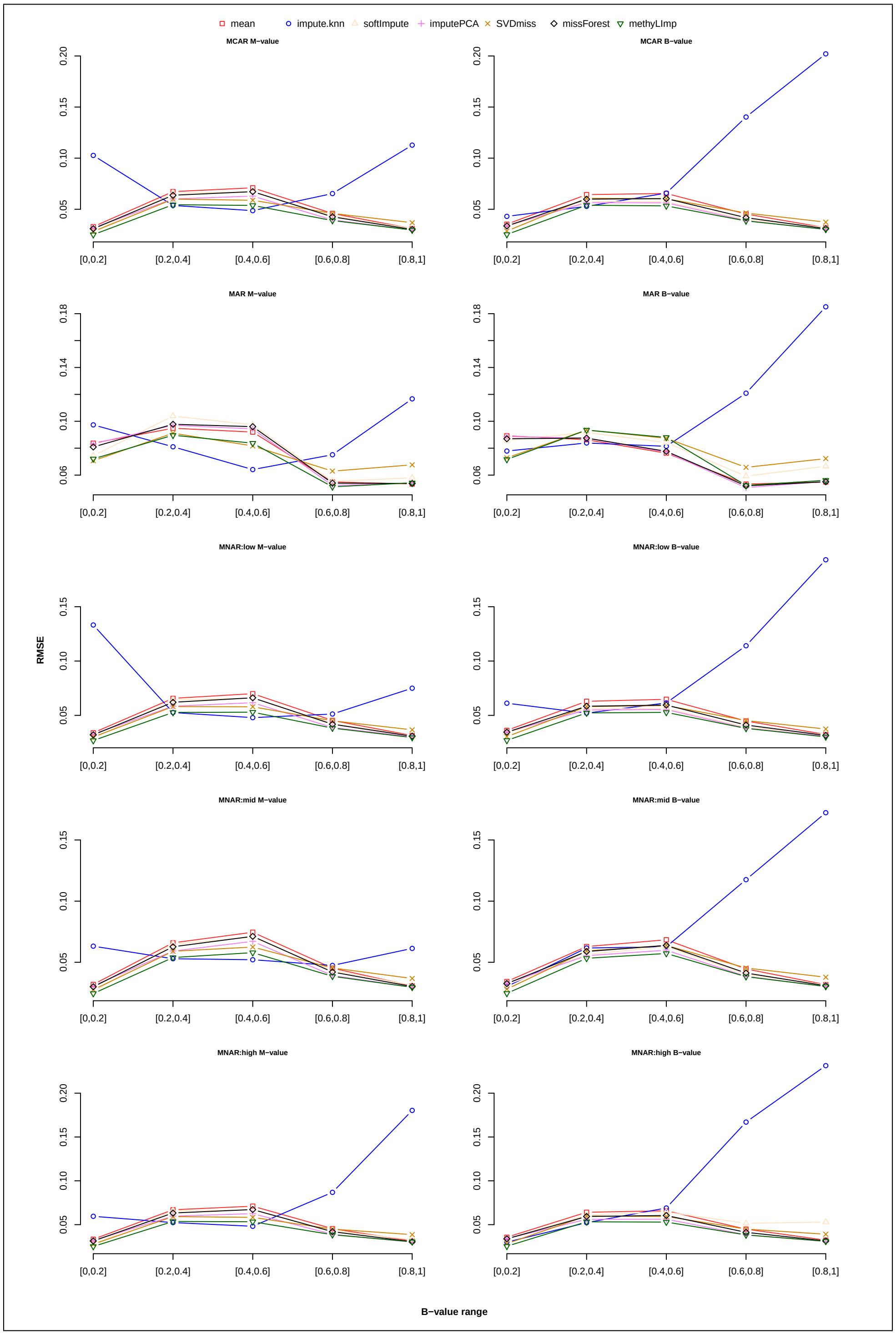

Figure 56: Dataset GSE61257 (D26). RMSE imputation performances with respect to B-value range.

2.27 GSE61257 (D27) - Adipose - Normal - 15 samples

Method	Avg time (sec)	Avg RAM (Mb)
mean	< 1	13
softImpute	< 1	48
imputePCA	6	169
impute.knn	< 1	51
SVDmiss	41	4056
methyLImp	29	105
missForest	6463	122

Table 171: Dataset GSE61257 (D27). Average time and memory usage.

Table 172: Dataset GSE61257 (D27). Imputation performance on **MCAR** type missing values.

	MAE		RMSE	
Method	M-value	B-value	M-value	B-value
mean	0.027±0.001	0.026±0.001*	0.046±0.001	0.046±0.001*
softImpute	0.025±0.001	0.024±0.001*	0.043±0.001	0.042±0.001*
impute.knn	0.033±0.007*	0.048±0.010	0.077±0.017*	0.104±0.018
imputePCA	0.022±0.001	0.022±0.001*	0.040±0.001	0.039±0.001*
SVDmiss	0.021±0.001	0.021±0.001	0.036±0.001	0.036±0.001*
missForest	0.023±0.001	0.023±0.001	0.039±0.001	0.039±0.001
methyLImp	0.020±0.001	**0.020±0.001***	0.035±0.001	**0.035±0.001***

Table 173: Dataset GSE61257 (D27). Imputation performance on **MAR** type missing values.

	MAE		RMSE	
Method	M-value	B-value	M-value	B-value
mean	0.041±0.001	0.039±0.001*	0.073±0.001	0.071±0.001*
softImpute	0.037±0.001*	0.039±0.001	0.068±0.002*	0.070±0.002
impute.knn	0.036±0.003*	0.045±0.007	0.072±0.009*	0.091±0.014
imputePCA	0.038±0.001	0.036±0.001*	0.071±0.002	0.069±0.001*
SVDmiss	**0.030±0.001***	0.030±0.001	0.060±0.002	**0.060±0.002***
missForest	0.037±0.001	0.036±0.001*	0.068±0.001	0.067±0.001*
methyLImp	0.035±0.001*	0.035±0.001	0.066±0.002*	0.068±0.002

Table 174: Dataset GSE61257 (D27). Imputation performance on **MNAR:low** type missing values.

	MAE		RMSE	
Method	M-value	B-value	M-value	B-value
mean	0.019±0.001*	0.020±0.001	0.039±0.001*	0.040±0.001
softImpute	0.018±0.001*	0.018±0.001	0.034±0.001*	0.036±0.002
impute.knn	0.033±0.010	0.024±0.005*	0.083±0.024	0.062±0.015*
imputePCA	0.017±0.001*	0.017±0.001	0.034±0.002*	0.035±0.001
SVDmiss	0.015±0.001*	0.016±0.001	0.030±0.002*	0.031±0.002
missForest	0.017±0.001*	0.017±0.001	0.033±0.002*	0.035±0.002
methyLImp	**0.015±0.001***	0.015±0.001	**0.030±0.002***	0.030±0.002

Table 175: Dataset GSE61257 (D27). Imputation performance on **MNAR:mid** type missing values.

	MAE		RMSE	
Method	M-value	B-value	M-value	B-value
mean	0.045±0.001	0.043±0.001*	0.068±0.001	0.063±0.001*
softImpute	0.042±0.001	0.039±0.001*	0.063±0.001	0.058±0.001*
impute.knn	0.031±0.001*	0.035±0.003	0.051±0.005*	0.061±0.009
imputePCA	0.035±0.001	0.034±0.001*	0.058±0.001	0.052±0.001*
SVDmiss	0.031±0.001	**0.029±0.001***	0.048±0.001	**0.046±0.001***
missForest	0.038±0.001	0.036±0.001*	0.058±0.001	0.053±0.001*
methyLImp	0.032±0.001	0.031±0.001*	0.049±0.001	0.048±0.001*

Table 176: Dataset GSE61257 (D27). Imputation performance on **MNAR:high** type missing values.

	MAE		RMSE	
Method	M-value	B-value	M-value	B-value
mean	0.021±0.001*	0.022±0.001	0.038±0.001*	0.038±0.001
softImpute	0.021±0.001*	0.021±0.001	0.036±0.001*	0.036±0.001
impute.knn	0.043±0.012*	0.078±0.019	0.105±0.028*	0.156±0.025
imputePCA	0.019±0.001*	0.019±0.001	0.034±0.001*	0.034±0.001
SVDmiss	0.019±0.001*	0.019±0.001	0.032±0.002*	0.033±0.001
missForest	0.019±0.001*	0.020±0.001	0.035±0.002*	0.035±0.002
methyLImp	0.018±0.001	**0.018±0.001***	**0.031±0.001***	0.031±0.001

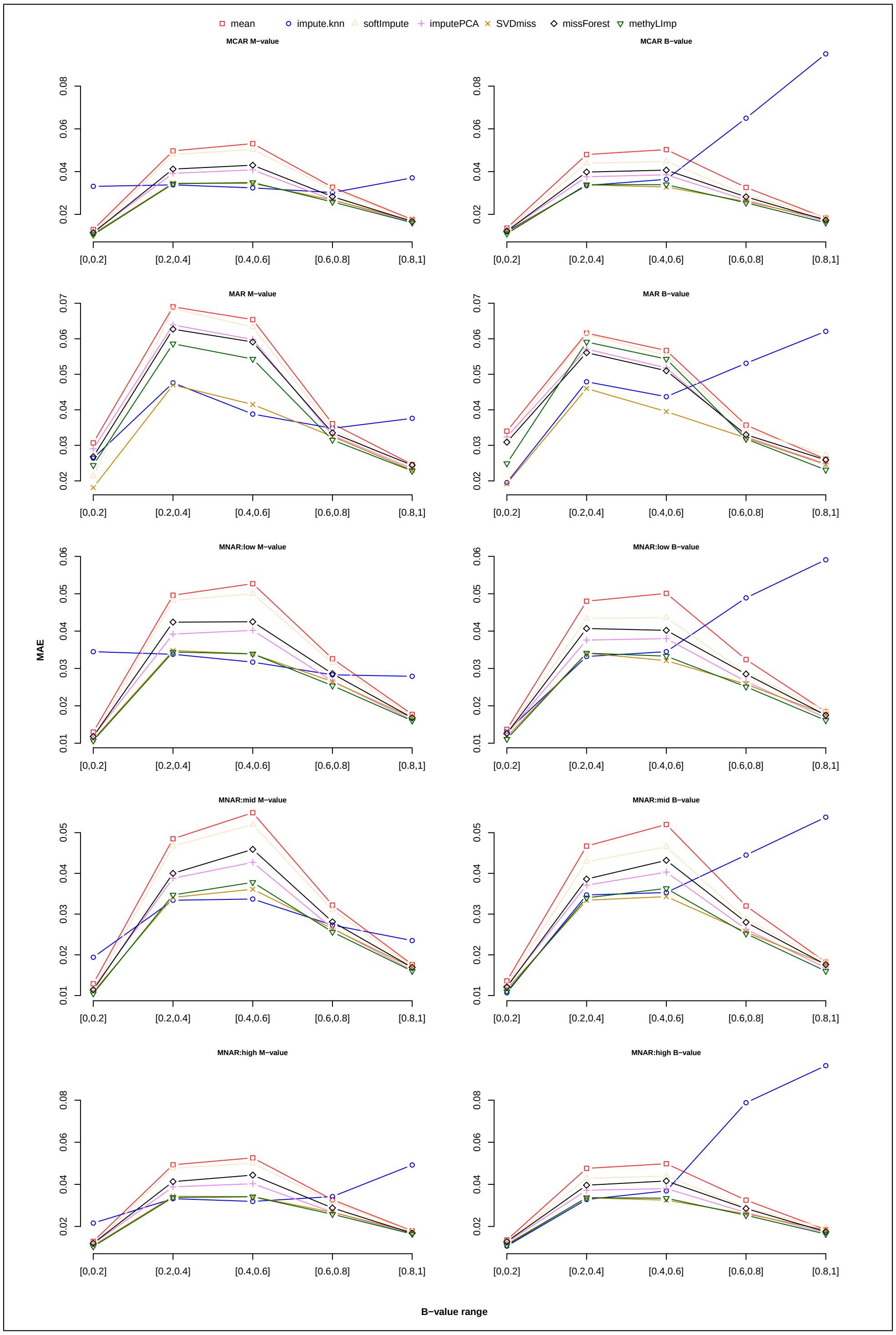

Figure 57: Dataset GSE61257 (D27). MAE imputation performances with respect to B-value range.

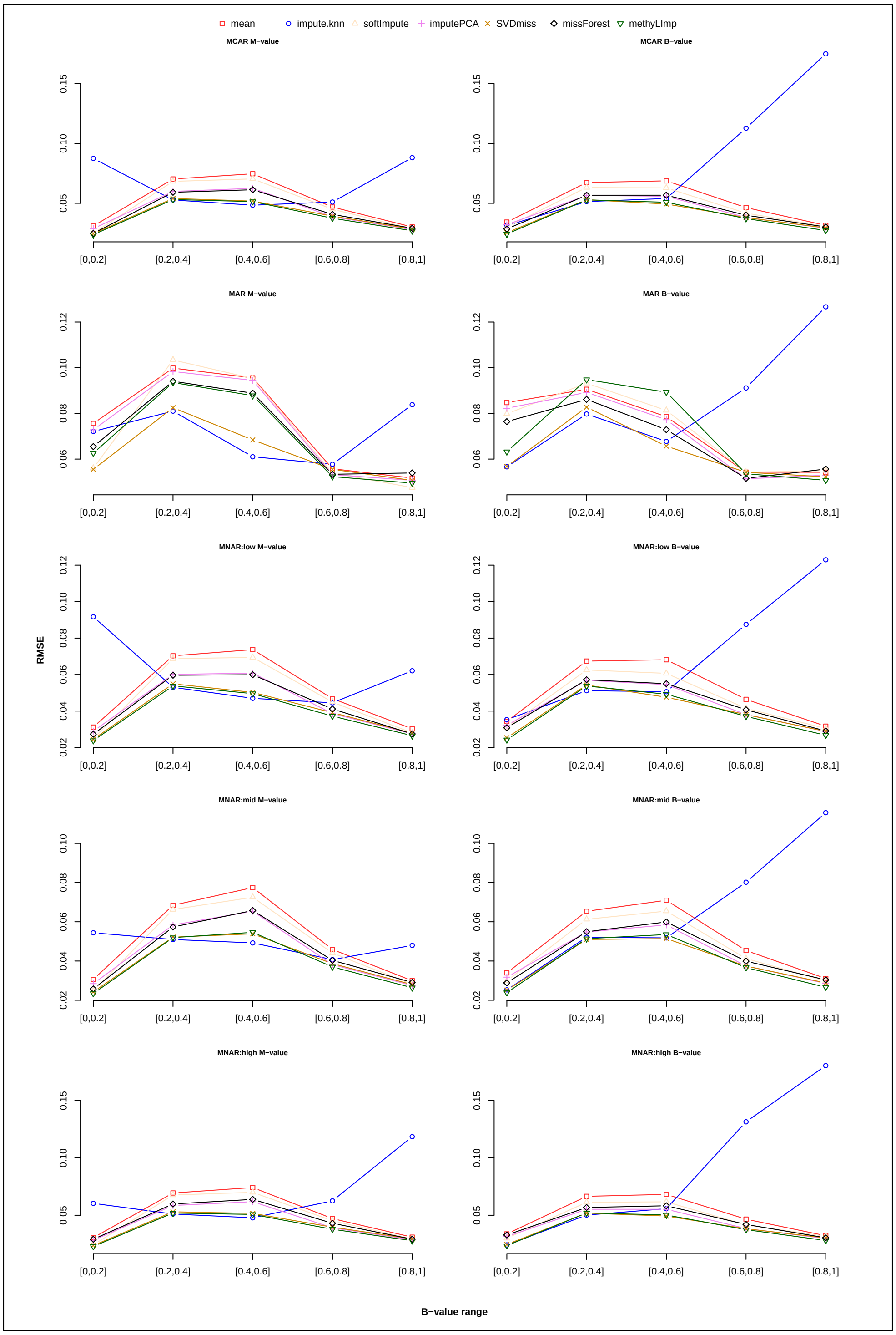

Figure 58: Dataset GSE61257 (D27). RMSE imputation performances with respect to B-value range.

2.28 GSE61258 (D28) - Liver - Non-alcoholic fatty liver disease (NAFLD) - 14 samples

Method	Avg time (sec)	Avg RAM (Mb)
mean	< 1	12
softImpute	< 1	45
imputePCA	12	166
impute.knn	< 1	51
SVDmiss	34	4095
methyLImp	21	109
missForest	8315	113

Table 177: Dataset GSE61258 (D28). Average time and memory usage.

Table 178: Dataset GSE61258 (D28). Imputation performance on **MCAR** type missing values.

	MAE		RMSE	
Method	M-value	B-value	M-value	B-value
mean	0.044±0.001*	0.045±0.001	0.074±0.001	0.074±0.001*
softImpute	0.034±0.001*	0.035±0.001	0.058±0.001*	0.060±0.001
impute.knn	0.036±0.004*	0.053±0.009	0.069±0.012*	0.104±0.017
imputePCA	0.029±0.001*	0.030±0.001	0.051±0.001	0.051±0.001*
SVDmiss	0.029±0.001*	0.034±0.001	0.052±0.001*	0.065±0.003
missForest	0.032±0.001*	0.033±0.001	0.057±0.001	0.056±0.001*
methyLImp	**0.027±0.001***	0.027±0.001	**0.047±0.001***	0.047±0.001

Table 179: Dataset GSE61258 (D28). Imputation performance on **MAR** type missing values.

	MAE		RMSE	
Method	M-value	B-value	M-value	B-value
mean	0.056±0.001*	0.057±0.001	0.092±0.001	0.090±0.001*
softImpute	0.046±0.001*	0.047±0.001	0.078±0.002*	0.080±0.002
impute.knn	0.044±0.002*	0.054±0.006	0.076±0.004*	0.096±0.012
imputePCA	0.043±0.001	0.043±0.001*	0.076±0.002	0.074±0.001*
SVDmiss	0.041±0.003*	0.051±0.002	0.078±0.007*	0.103±0.010
missForest	0.045±0.001*	0.046±0.001	0.078±0.001	0.076±0.001*
methyLImp	**0.039±0.001***	0.039±0.001	**0.070±0.002***	0.070±0.002

Table 180: Dataset GSE61258 (D28). Imputation performance on **MNAR:low** type missing values.

	MAE		RMSE	
Method	M-value	B-value	M-value	B-value
mean	0.031±0.001*	0.033±0.001	0.058±0.002*	0.061±0.001
softImpute	0.024±0.001*	0.027±0.001	0.047±0.001*	0.051±0.001
impute.knn	0.037±0.010	0.028±0.004*	0.083±0.023	0.062±0.012*
imputePCA	0.021±0.001*	0.022±0.001	0.042±0.001*	0.044±0.001
SVDmiss	0.021±0.001*	0.027±0.001	0.043±0.002*	0.062±0.005
missForest	0.023±0.001*	0.024±0.001	0.045±0.002*	0.046±0.001
methyLImp	**0.020±0.001***	0.020±0.001	**0.038±0.001***	0.038±0.001

Table 181: Dataset GSE61258 (D28). Imputation performance on **MNAR:mid** type missing values.

	MAE		RMSE	
Method	M-value	B-value	M-value	B-value
mean	0.075±0.001	0.072±0.001*	0.110±0.001	0.105±0.001*
softImpute	0.055±0.001	0.054±0.001*	0.086±0.001	0.085±0.001*
impute.knn	0.045±0.001*	0.050±0.003	**0.069±0.002***	0.083±0.006
imputePCA	0.048±0.001	0.046±0.001*	0.077±0.001	0.072±0.001*
SVDmiss	0.046±0.001*	0.050±0.001	0.073±0.001*	0.083±0.001
missForest	0.055±0.001	0.052±0.001*	0.086±0.001	0.081±0.001*
methyLImp	0.044±0.001	**0.044±0.001***	0.070±0.001	**0.069±0.001***

Table 182: Dataset GSE61258 (D28). Imputation performance on **MNAR:high** type missing values.

	MAE		RMSE	
Method	M-value	B-value	M-value	B-value
mean	0.035±0.001*	0.037±0.001	0.060±0.001*	0.061±0.001
softImpute	0.028±0.001*	0.029±0.001	0.048±0.001*	0.050±0.001
impute.knn	0.040±0.011*	0.087±0.022	0.083±0.028*	0.160±0.029
imputePCA	0.024±0.001*	0.025±0.001	0.042±0.001*	0.042±0.001
SVDmiss	0.025±0.001*	0.029±0.001	0.043±0.002*	0.055±0.003
missForest	0.026±0.001*	0.028±0.001	0.045±0.001*	0.046±0.001
methyLImp	**0.023±0.001***	0.023±0.001	**0.039±0.001***	0.039±0.001

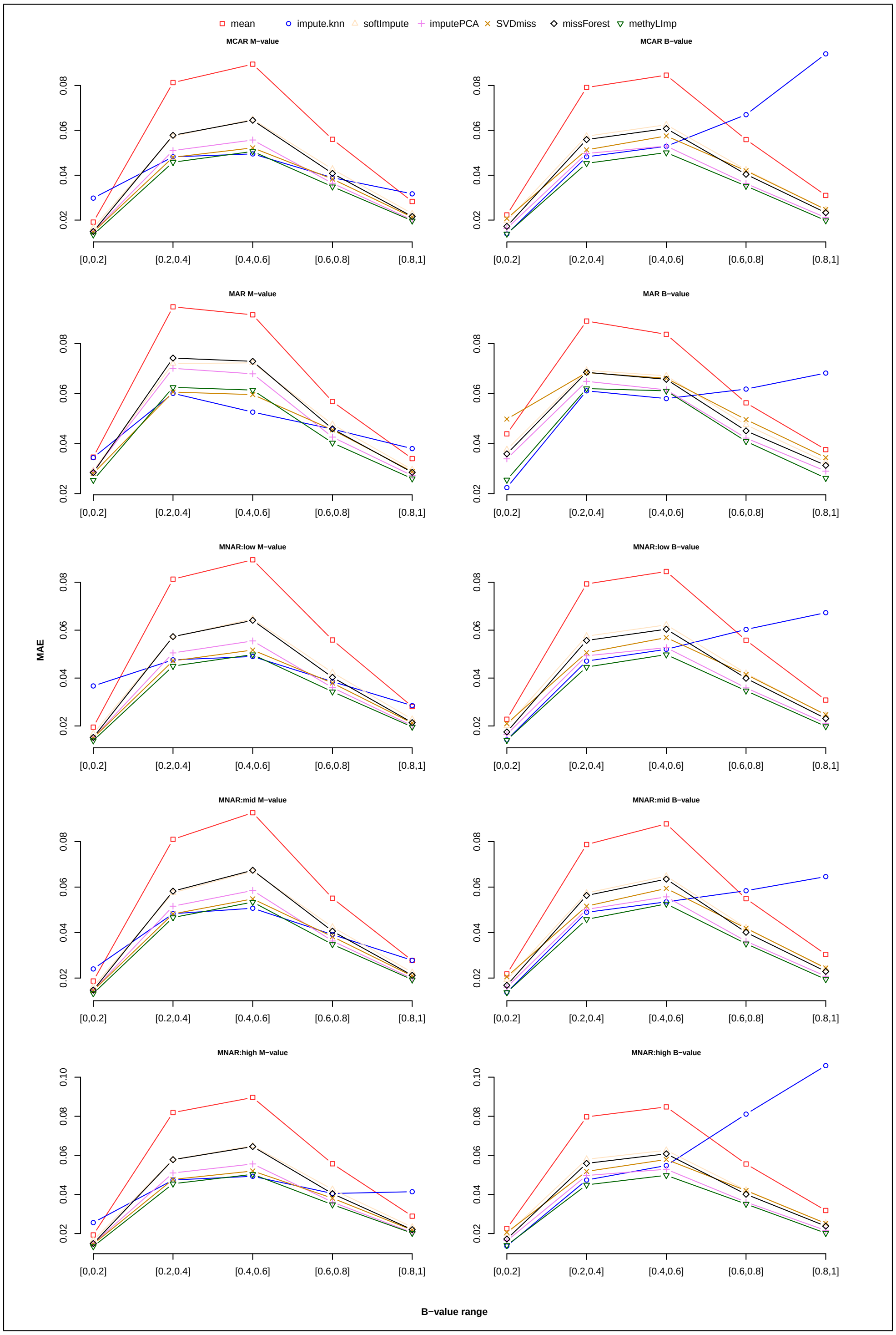

Figure 59: Dataset GSE61258 (D28). MAE imputation performances with respect to B-value range.

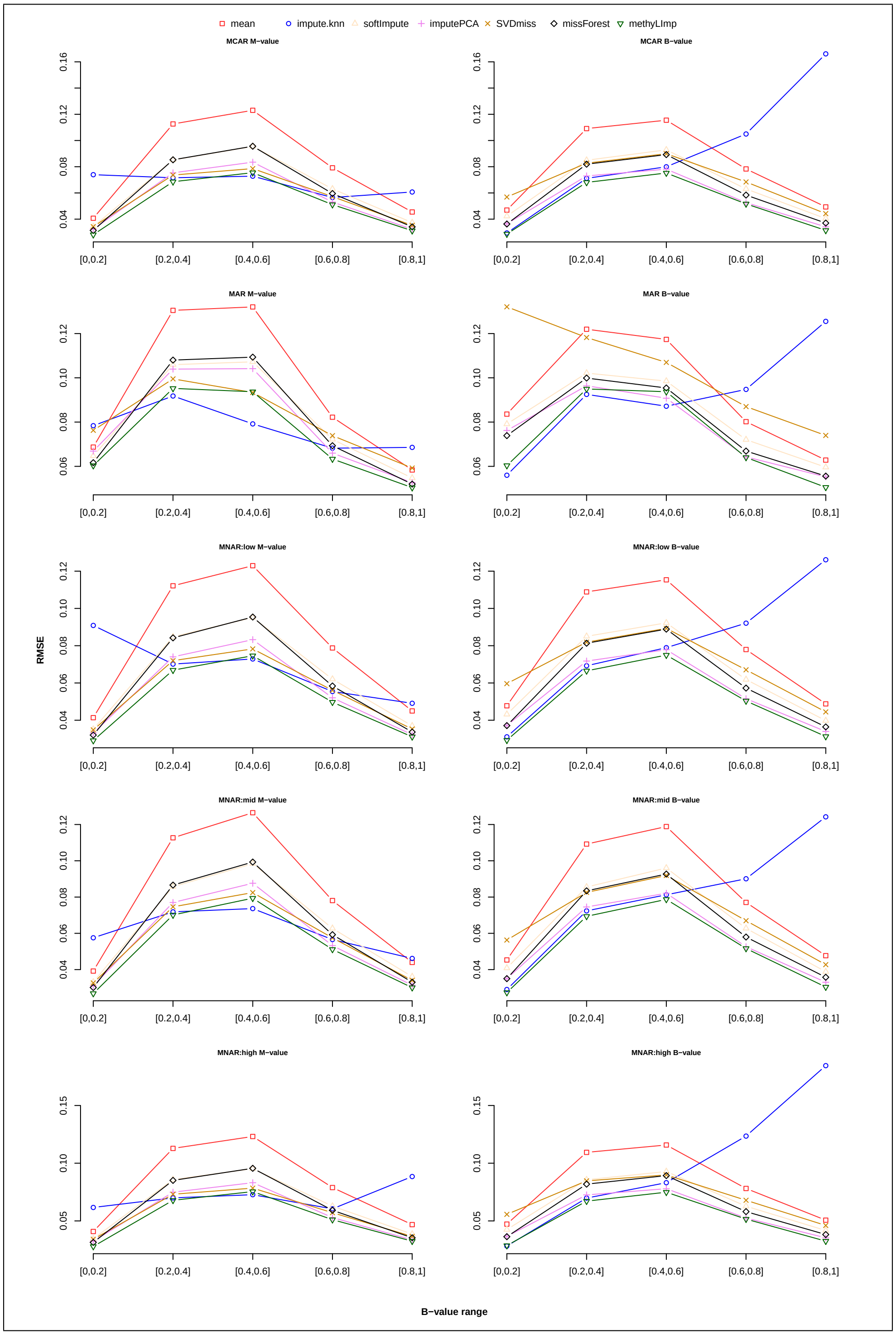

Figure 60: Dataset GSE61258 (D28). RMSE imputation performances with respect to B-value range.

2.29 GSE61258 (D29) - Liver - Non-alcoholic steatohepatitis (NASH) - 7 samples

Method	Avg time (sec)	Avg RAM (Mb)
mean	< 1	6
softImpute	< 1	41
imputePCA	9	140
impute.knn	< 1	41
SVDmiss	12	3808
methyLImp	3	126
missForest	836	253

Table 183: Dataset GSE61258 (D29). Average time and memory usage.

Table 184: Dataset GSE61258 (D29). Imputation performance on **MCAR** type missing values.

	MAE		RMSE	
Method	M-value	B-value	M-value	B-value
mean	0.056±0.001*	0.057±0.001	0.093±0.002*	0.093±0.002
softImpute	0.056±0.002*	0.061±0.006	0.106±0.005*	0.116±0.015
impute.knn	0.073±0.010*	0.097±0.017	0.124±0.015*	0.164±0.021
imputePCA	0.047±0.001*	0.048±0.001	0.085±0.002	0.085±0.002*
SVDmiss	0.057±0.002	0.048±0.001*	0.105±0.003	0.086±0.003*
missForest	0.049±0.001*	0.050±0.001	0.087±0.002	0.086±0.002*
methyLImp	**0.045±0.001***	0.045±0.001	**0.081±0.002**	**0.081±0.002**

Table 185: Dataset GSE61258 (D29). Imputation performance on **MAR** type missing values.

	MAE		RMSE	
Method	M-value	B-value	M-value	B-value
mean	0.061±0.001*	0.062±0.001	0.100±0.002	0.099±0.002*
softImpute	0.063±0.002*	0.070±0.009	0.114±0.004*	0.133±0.023
impute.knn	0.082±0.006*	0.102±0.014	0.133±0.008*	0.166±0.017
imputePCA	0.054±0.001*	0.054±0.001	0.094±0.002	0.093±0.002*
SVDmiss	0.065±0.001	0.055±0.001*	0.117±0.003	0.103±0.004*
missForest	0.055±0.001*	0.056±0.001	0.095±0.002	0.094±0.002*
methyLImp	**0.051±0.001***	0.052±0.001	**0.092±0.002***	0.092±0.002

Table 186: Dataset GSE61258 (D29). Imputation performance on **MNAR:low** type missing values.

	MAE		RMSE	
Method	M-value	B-value	M-value	B-value
mean	0.038±0.001*	0.040±0.001	0.074±0.002*	0.075±0.002
softImpute	0.041±0.002*	0.045±0.005	0.093±0.005*	0.099±0.014
impute.knn	0.084±0.020	0.052±0.008*	0.149±0.027	0.111±0.015*
imputePCA	0.032±0.001*	0.033±0.001	0.067±0.002*	0.068±0.002
SVDmiss	0.041±0.001	0.034±0.001*	0.082±0.003	0.069±0.003*
missForest	0.034±0.001*	0.035±0.001	0.069±0.002*	0.070±0.002
methyLImp	**0.032±0.001***	0.032±0.001	**0.065±0.002***	0.065±0.002

Table 187: Dataset GSE61258 (D29). Imputation performance on **MNAR:mid** type missing values.

	MAE		RMSE	
Method	M-value	B-value	M-value	B-value
mean	0.089±0.001	0.087±0.001*	0.129±0.002	0.125±0.002*
softImpute	0.085±0.001*	0.088±0.004	0.130±0.002*	0.142±0.009
impute.knn	**0.069±0.002***	0.091±0.008	**0.104±0.004***	0.141±0.011
imputePCA	0.079±0.001	0.077±0.001*	0.120±0.002	0.117±0.002*
SVDmiss	0.091±0.002	0.072±0.001*	0.146±0.002	0.114±0.003*
missForest	0.081±0.001	0.078±0.001*	0.121±0.002	0.117±0.002*
methyLImp	0.072±0.001	0.072±0.001*	0.112±0.002	0.112±0.002*

Table 188: Dataset GSE61258 (D29). Imputation performance on **MNAR:high** type missing values.

	MAE		RMSE	
Method	M-value	B-value	M-value	B-value
mean	0.046±0.001*	0.050±0.001	0.075±0.002*	0.078±0.002
softImpute	0.046±0.002*	0.056±0.012	0.091±0.006*	0.110±0.030
impute.knn	0.094±0.022*	0.182±0.033	0.159±0.031*	0.258±0.029
imputePCA	0.036±0.001*	0.038±0.001	0.065±0.002*	0.067±0.002
SVDmiss	0.043±0.001	0.042±0.001*	0.080±0.002	0.077±0.004*
missForest	0.039±0.001*	0.042±0.001	0.068±0.002*	0.070±0.002
methyLImp	**0.035±0.001***	0.035±0.001	0.063±0.002	**0.063±0.002***

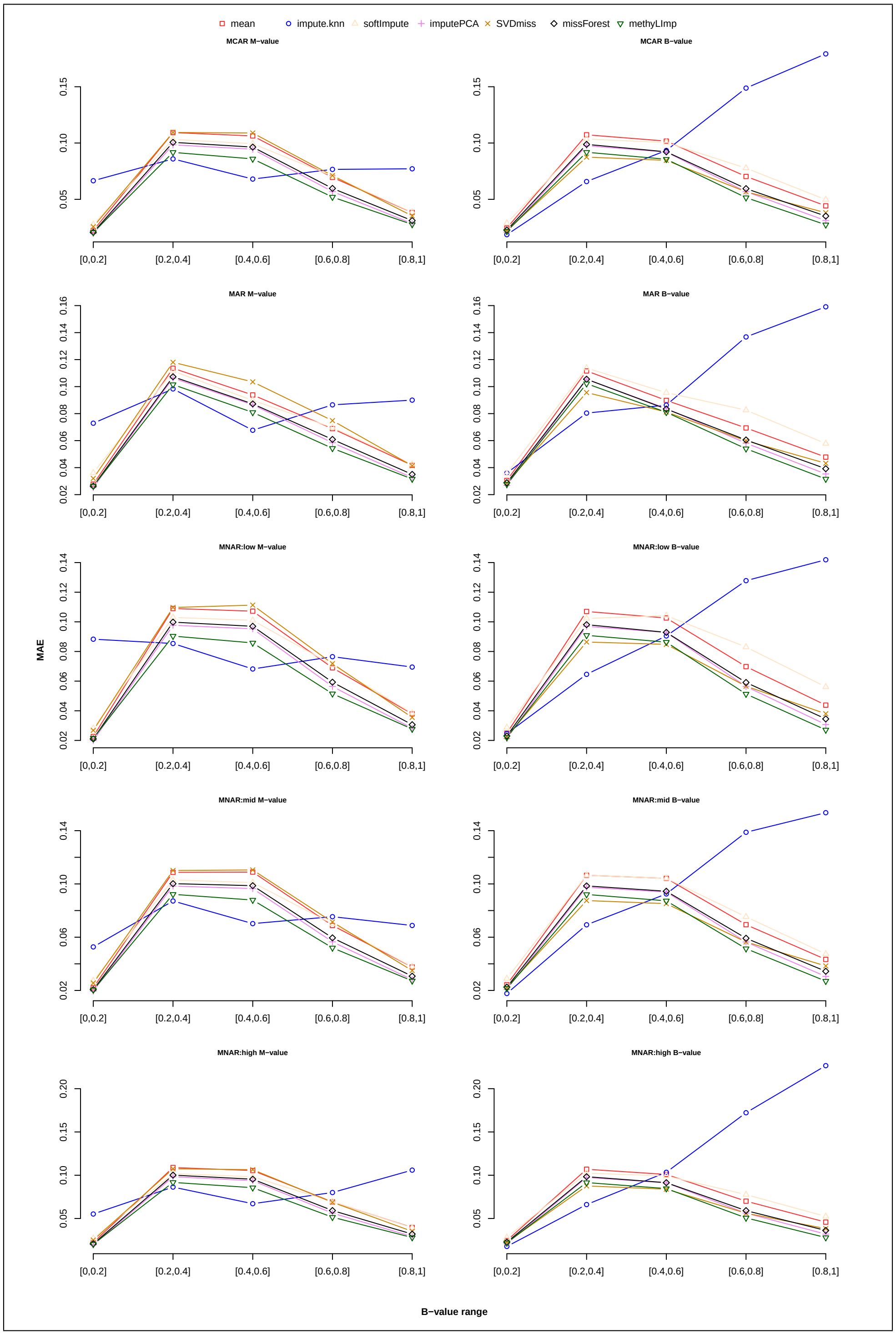

Figure 61: Dataset GSE61258 (D29). MAE imputation performances with respect to B-value range.

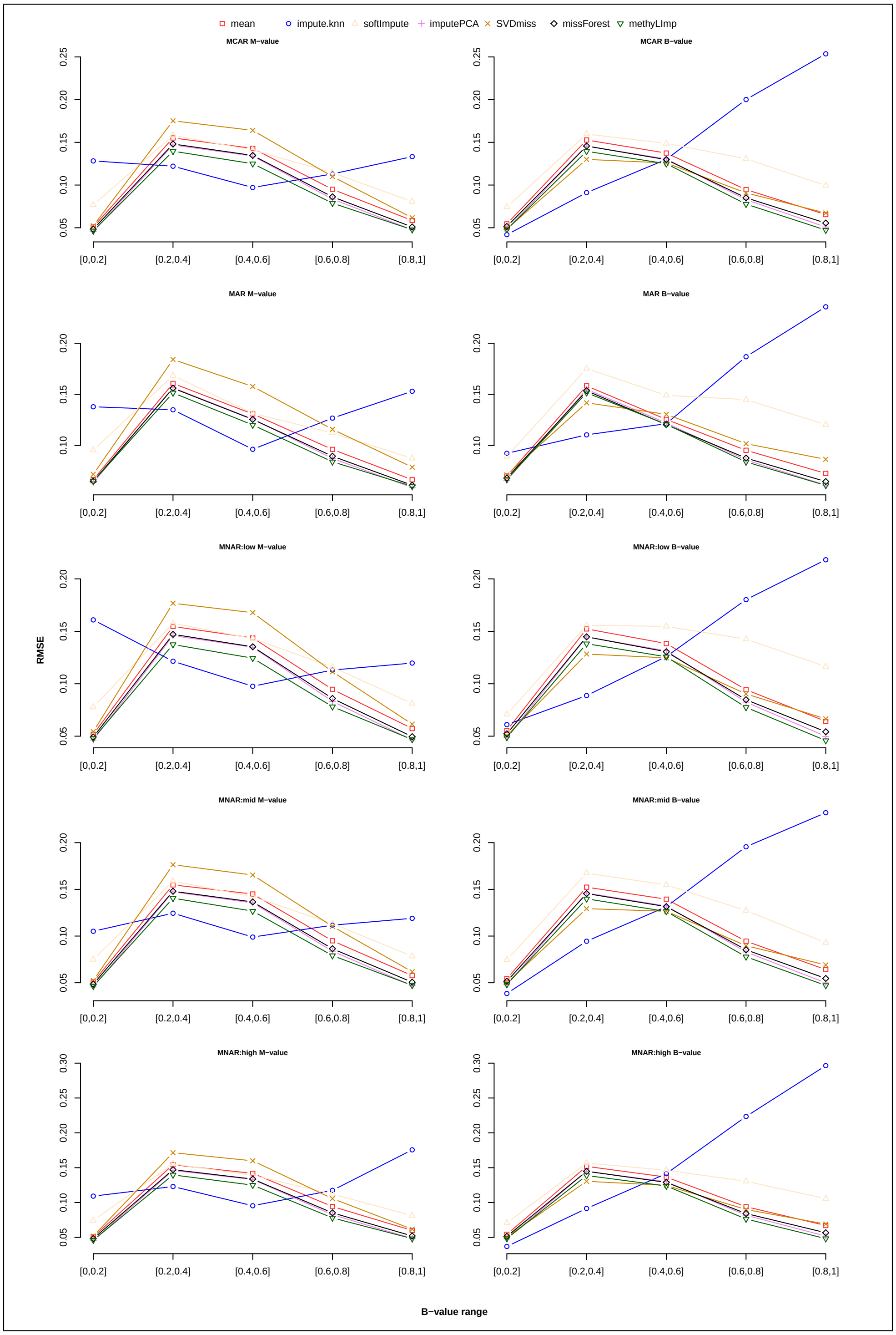

Figure 62: Dataset GSE61258 (D29). RMSE imputation performances with respect to B-value range.

2.30 GSE61258 (D30) - Liver - Normal - 32 samples

Method	Avg time (sec)	Avg RAM (Mb)
mean	< 1	27
softImpute	< 1	75
imputePCA	43	211
impute.knn	1	79
SVDmiss	128	4828
methyLImp	376	125
missForest	38764	204

Table 189: Dataset GSE61258 (D30). Average time and memory usage.

Table 190: Dataset GSE61258 (D30). Imputation performance on **MCAR** type missing values.

	MAE		RMSE	
Method	M-value	B-value	M-value	B-value
mean	0.033±0.001*	0.033±0.001	0.061±0.001	0.060±0.001*
softImpute	0.030±0.001*	0.031±0.001	0.059±0.001	0.058±0.001*
impute.knn	0.028±0.002*	0.036±0.004	0.056±0.005*	0.077±0.011
imputePCA	0.030±0.001*	0.030±0.001	0.057±0.001*	0.059±0.002
SVDmiss	0.028±0.001	0.027±0.001*	0.058±0.001	0.050±0.001*
missForest	0.026±0.001*	0.026±0.001	0.050±0.001	0.050±0.001*
methyLImp	**0.024±0.001***	0.025±0.001	**0.046±0.001***	0.047±0.001

Table 191: Dataset GSE61258 (D30). Imputation performance on **MAR** type missing values.

	MAE		RMSE	
Method	M-value	B-value	M-value	B-value
mean	0.046±0.001*	0.047±0.001	0.082±0.001	0.080±0.001*
softImpute	0.045±0.001*	0.046±0.001	0.083±0.002	0.081±0.002*
impute.knn	0.035±0.001*	0.041±0.003	0.066±0.003*	0.079±0.007
imputePCA	0.044±0.001*	0.046±0.001	0.085±0.001*	0.090±0.002
SVDmiss	0.035±0.001	**0.034±0.001***	0.070±0.001	0.065±0.001*
missForest	**0.034±0.001***	0.034±0.001	0.064±0.001	**0.063±0.001***
methyLImp	0.037±0.001*	0.038±0.001	0.070±0.001*	0.075±0.001

Table 192: Dataset GSE61258 (D30). Imputation performance on **MNAR:low** type missing values.

	MAE		RMSE	
Method	M-value	B-value	M-value	B-value
mean	0.024±0.001*	0.026±0.001	0.051±0.001*	0.052±0.001
softImpute	0.022±0.001*	0.024±0.001	0.050±0.002*	0.051±0.002
impute.knn	0.021±0.002*	0.023±0.002	0.048±0.008*	0.055±0.007
imputePCA	0.023±0.001*	0.024±0.001	0.051±0.002*	0.054±0.002
SVDmiss	0.021±0.001	0.021±0.001*	0.048±0.002	0.044±0.001*
missForest	0.019±0.001*	0.020±0.001	0.042±0.001*	0.042±0.001
methyLImp	**0.018±0.001***	0.019±0.001	**0.039±0.001***	0.040±0.001

Table 193: Dataset GSE61258 (D30). Imputation performance on **MNAR:mid** type missing values.

	MAE		RMSE	
Method	M-value	B-value	M-value	B-value
mean	0.054±0.001	0.052±0.001*	0.089±0.001	0.084±0.001*
softImpute	0.050±0.001	0.047±0.001*	0.084±0.001	0.079±0.001*
impute.knn	**0.038±0.001***	0.041±0.002	**0.062±0.002***	0.070±0.005
imputePCA	0.048±0.001	0.048±0.001*	0.080±0.001*	0.080±0.001
SVDmiss	0.047±0.001	0.040±0.001*	0.086±0.001	0.066±0.001*
missForest	0.043±0.001	0.041±0.001*	0.072±0.001	0.069±0.001*
methyLImp	**0.038±0.001***	0.039±0.001	0.063±0.001*	0.064±0.001

Table 194: Dataset GSE61258 (D30). Imputation performance on **MNAR:high** type missing values.

	MAE		RMSE	
Method	M-value	B-value	M-value	B-value
mean	0.027±0.001*	0.028±0.001	0.050±0.001*	0.051±0.001
softImpute	0.025±0.001*	0.027±0.001	0.050±0.001*	0.052±0.002
impute.knn	0.025±0.002*	0.043±0.008	0.053±0.007*	0.097±0.017
imputePCA	0.024±0.001*	0.026±0.001	0.047±0.001*	0.050±0.002
SVDmiss	0.023±0.001*	0.023±0.001	0.046±0.001	0.044±0.001*
missForest	0.022±0.001*	0.023±0.001	0.042±0.001*	0.043±0.001
methyLImp	**0.021±0.001***	0.021±0.001	**0.040±0.001***	0.040±0.001

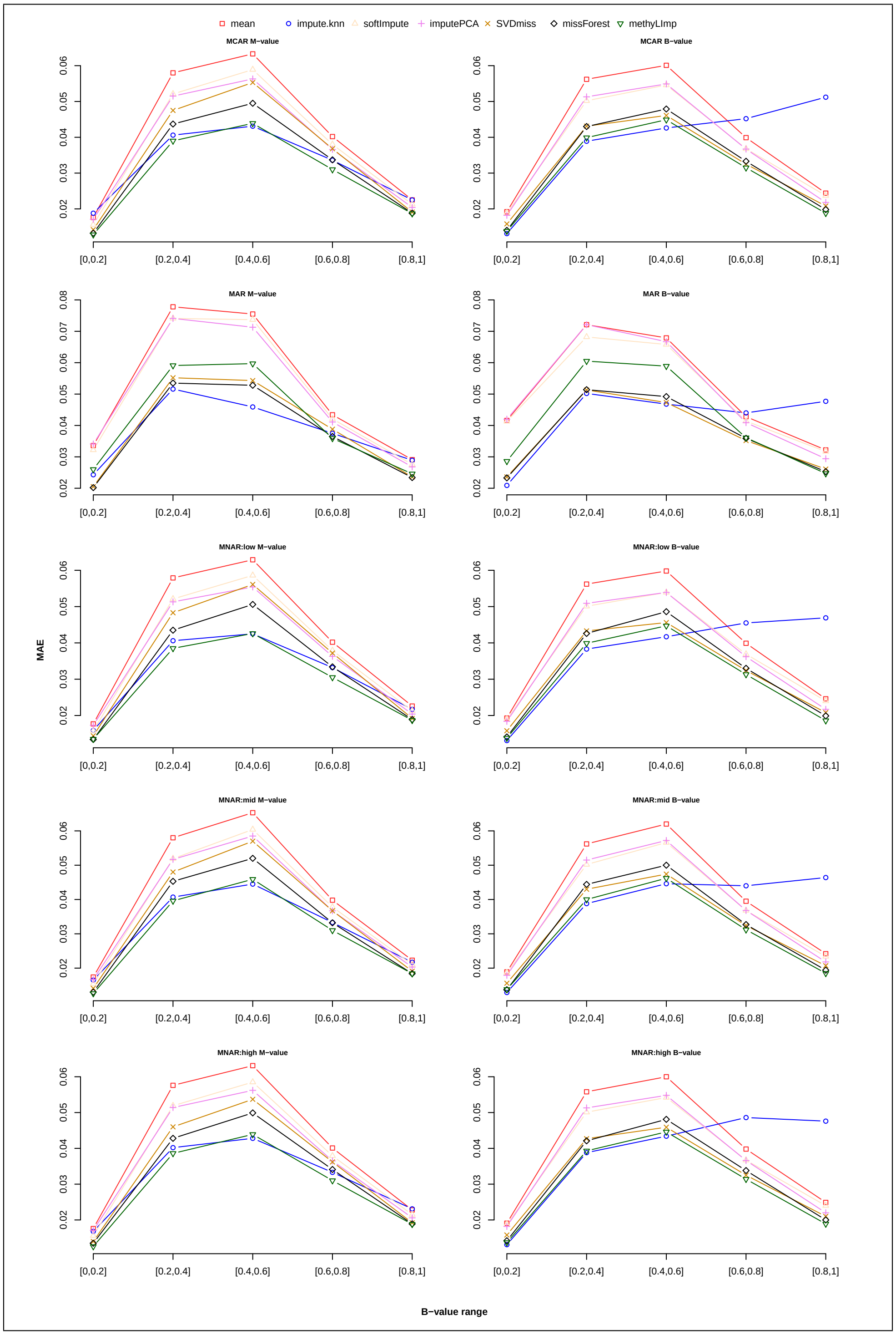

Figure 63: Dataset GSE61258 (D30). MAE imputation performances with respect to B-value range.

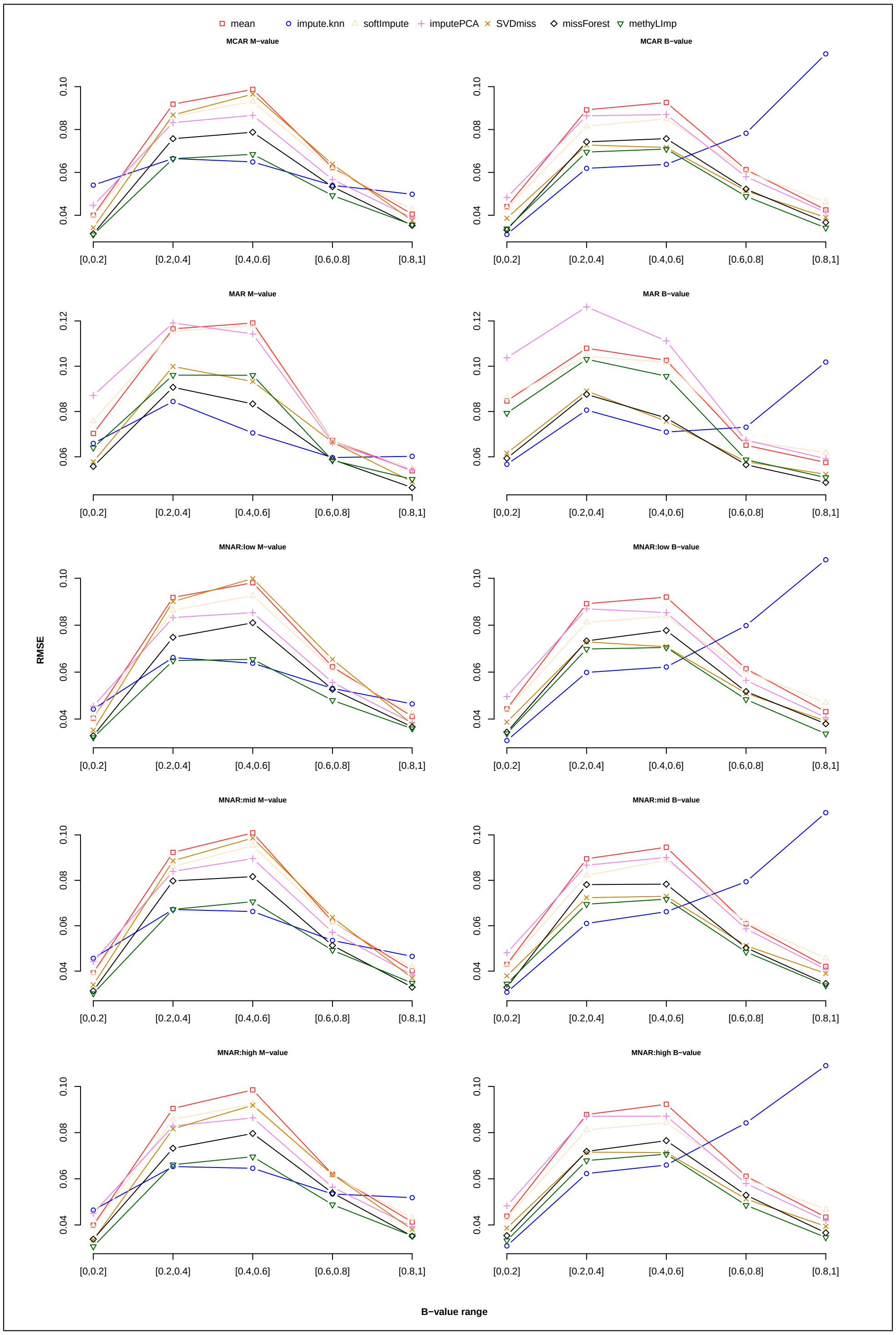

Figure 64: Dataset GSE61258 (D30). RMSE imputation performances with respect to B-value range.

2.31 GSE61258 (D31) - Liver - Primary biliary cholangitis (PBC) - 12 samples

Method	Avg time (sec)	Avg RAM (Mb)
mean	< 1	10
softImpute	< 1	43
imputePCA	7	154
impute.knn	< 1	51
SVDmiss	28	4013
methyLImp	13	111
missForest	4402	100

Table 195: Dataset GSE61258 (D31). Average time and memory usage.

Table 196: Dataset GSE61258 (D31). Imputation performance on **MCAR** type missing values.

	MAE		RMSE	
Method	M-value	B-value	M-value	B-value
mean	0.028±0.001	0.028±0.001*	0.048±0.001	0.048±0.001*
softImpute	0.025±0.001	0.025±0.001*	0.044±0.002	0.044±0.002
impute.knn	0.038±0.009*	0.055±0.013	0.083±0.021*	0.113±0.022
imputePCA	0.025±0.001	0.024±0.001*	0.043±0.001	0.042±0.001*
SVDmiss	0.025±0.001	0.025±0.001*	0.043±0.002	0.044±0.002
missForest	0.026±0.001	0.026±0.001*	0.045±0.001	0.044±0.001*
methyLImp	**0.023±0.001***	0.023±0.001	0.039±0.001	**0.039±0.001***

Table 197: Dataset GSE61258 (D31). Imputation performance on **MAR** type missing values.

	MAE		RMSE	
Method	M-value	B-value	M-value	B-value
mean	0.041±0.001	0.040±0.001*	0.073±0.001	0.070±0.001*
softImpute	0.040±0.001	0.041±0.002	0.075±0.002*	0.078±0.007
impute.knn	0.040±0.004*	0.054±0.008	0.078±0.011*	0.104±0.014
imputePCA	0.039±0.001	0.038±0.001*	0.073±0.002	0.070±0.001*
SVDmiss	**0.035±0.001***	0.035±0.001	0.067±0.002*	0.069±0.003
missForest	0.039±0.001	0.038±0.001*	0.071±0.001	0.068±0.001*
methyLImp	0.036±0.001	0.036±0.001	**0.066±0.002***	0.067±0.002

Table 198: Dataset GSE61258 (D31). Imputation performance on **MNAR:low** type missing values.

	MAE		RMSE	
Method	M-value	B-value	M-value	B-value
mean	0.021±0.001*	0.021±0.001	0.041±0.002*	0.042±0.002
softImpute	0.019±0.001*	0.019±0.001	0.038±0.002*	0.040±0.002
impute.knn	0.038±0.013	0.029±0.006*	0.091±0.029	0.072±0.017*
imputePCA	0.018±0.001*	0.019±0.001	0.037±0.002*	0.038±0.002
SVDmiss	0.019±0.001*	0.019±0.001	0.036±0.002	0.036±0.002
missForest	0.019±0.001*	0.019±0.001	0.037±0.002*	0.039±0.002
methyLImp	**0.017±0.001***	0.017±0.001	**0.032±0.002***	0.033±0.002

Table 199: Dataset GSE61258 (D31). Imputation performance on **MNAR:mid** type missing values.

	MAE		RMSE	
Method	M-value	B-value	M-value	B-value
mean	0.044±0.001	0.042±0.001*	0.067±0.001	0.063±0.001*
softImpute	0.039±0.001	0.037±0.001*	0.062±0.001	0.062±0.004
impute.knn	0.035±0.002*	0.043±0.004	0.058±0.005*	0.076±0.010
imputePCA	0.038±0.001	0.037±0.001*	0.061±0.001	0.057±0.001*
SVDmiss	0.038±0.001	0.037±0.001*	0.059±0.001	0.058±0.002*
missForest	0.041±0.001	0.039±0.001*	0.063±0.001	0.059±0.001*
methyLImp	0.035±0.001	**0.035±0.001***	0.054±0.001	**0.054±0.001***

Table 200: Dataset GSE61258 (D31). Imputation performance on **MNAR:high** type missing values.

	MAE		RMSE	
Method	M-value	B-value	M-value	B-value
mean	0.023±0.001*	0.023±0.001	0.039±0.001*	0.039±0.001
softImpute	0.021±0.001*	0.024±0.011	0.037±0.001*	0.046±0.035
impute.knn	0.055±0.017*	0.098±0.025	0.123±0.030*	0.176±0.028
imputePCA	0.020±0.001*	0.021±0.001	0.036±0.001*	0.036±0.001
SVDmiss	0.022±0.001*	0.023±0.001	0.038±0.002*	0.039±0.002
missForest	0.021±0.001*	0.022±0.001	0.036±0.001*	0.037±0.001
methyLImp	0.020±0.001	**0.020±0.001***	**0.033±0.001**	**0.033±0.001**

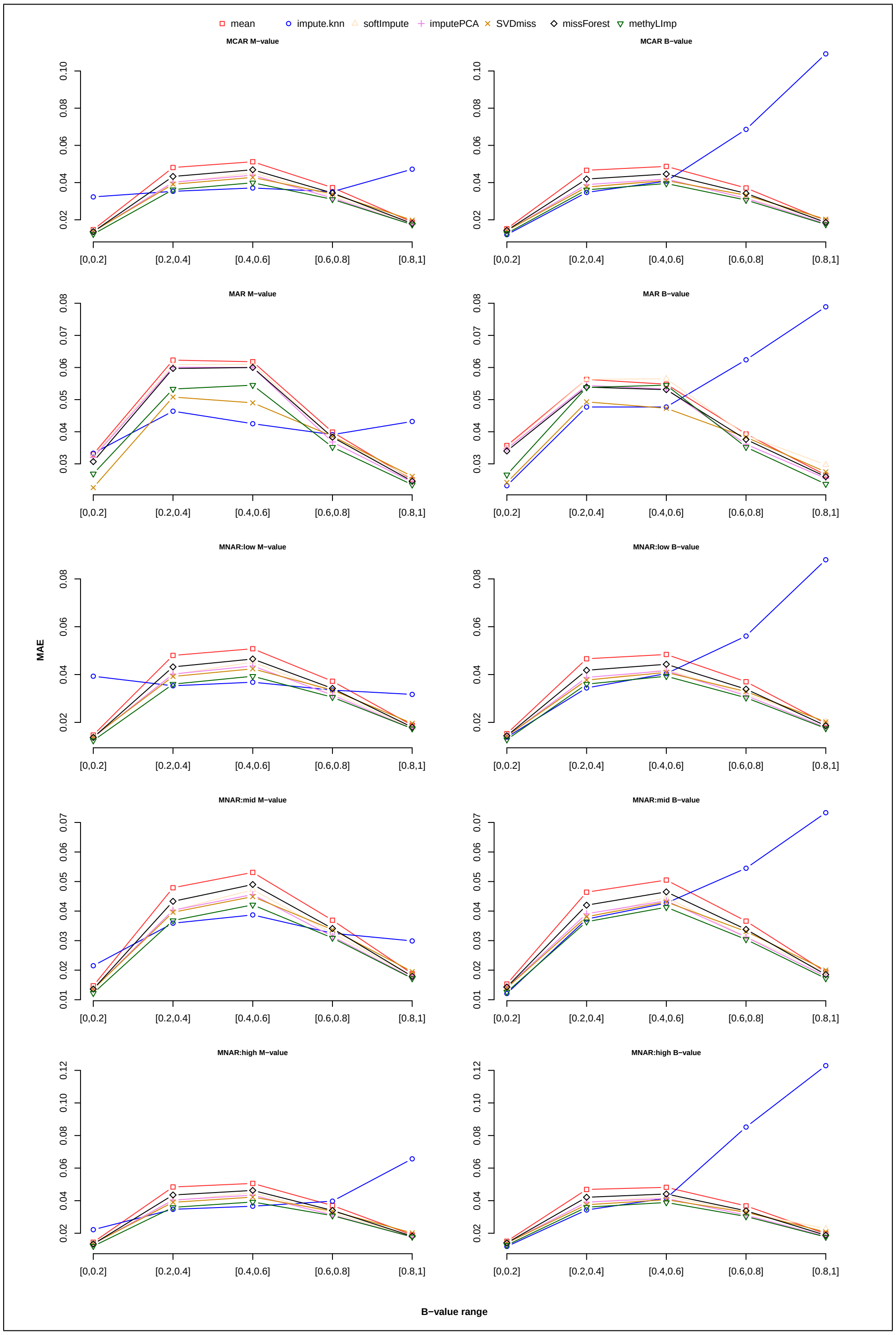

Figure 65: Dataset GSE61258 (D31). MAE imputation performances with respect to B-value range.

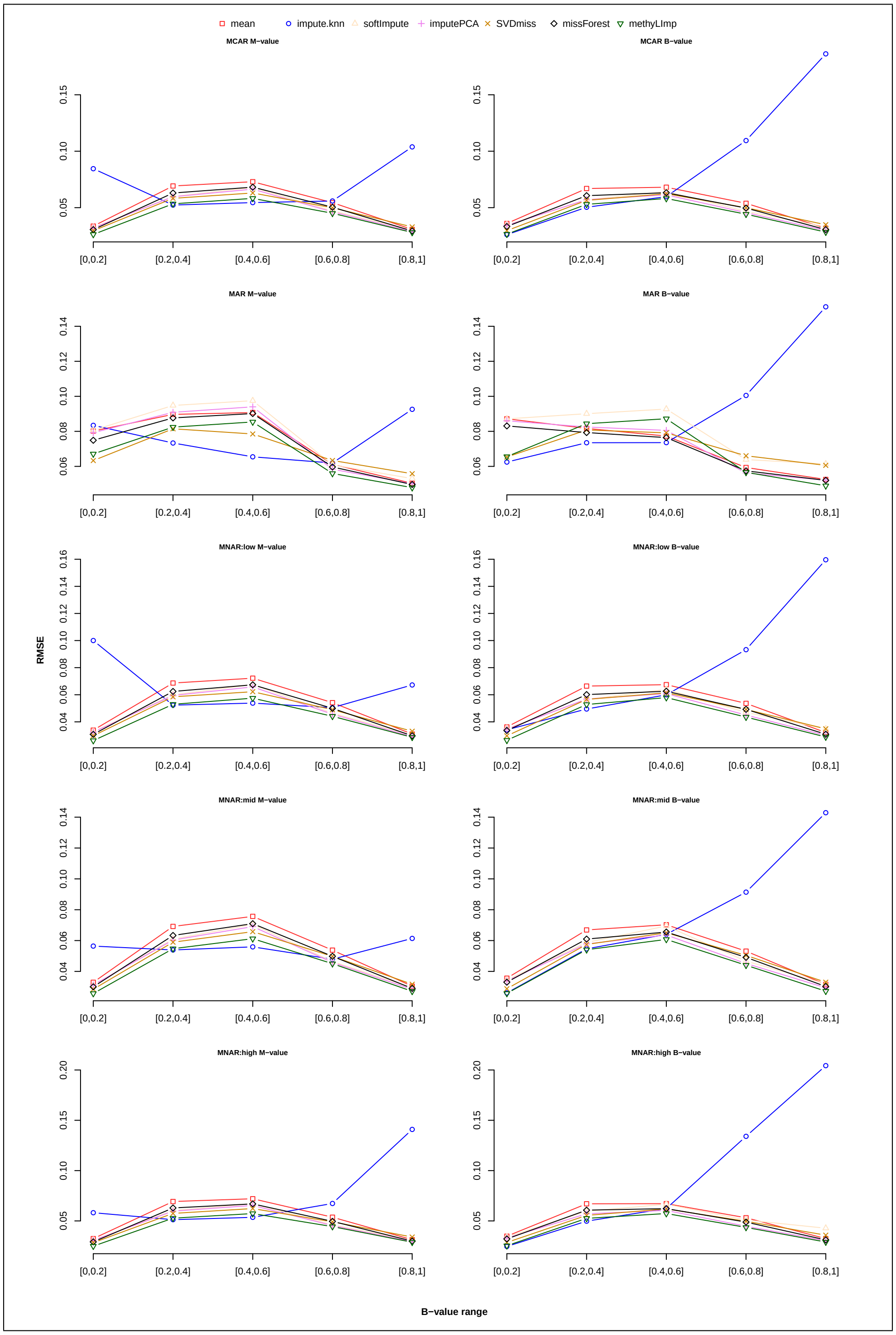

Figure 66: Dataset GSE61258 (D31). RMSE imputation performances with respect to B-value range.

2.32 GSE61258 (D32) - Liver - Primary sclerosing cholangitis (PSC) - 14 samples

Method	Avg time (sec)	Avg RAM (Mb)
mean	< 1	12
softImpute	< 1	46
imputePCA	6	158
impute.knn	< 1	51
SVDmiss	35	4065
methyLImp	21	108
missForest	5280	117

Table 201: Dataset GSE61258 (D32). Average time and memory usage.

Table 202: Dataset GSE61258 (D32). Imputation performance on **MCAR** type missing values.

	MAE		RMSE	
Method	M-value	B-value	M-value	B-value
mean	0.026±0.001*	0.026±0.001	0.044±0.001	0.043±0.001*
softImpute	0.025±0.001*	0.025±0.001	0.041±0.001	0.041±0.001*
impute.knn	0.036±0.007*	0.054±0.013	0.080±0.019*	0.113±0.022
imputePCA	0.024±0.001*	0.024±0.001	0.042±0.001	0.042±0.001*
SVDmiss	0.024±0.001	0.024±0.001*	0.041±0.001	0.041±0.002*
missForest	0.024±0.001*	0.024±0.001	0.041±0.001	0.040±0.001*
methyLImp	**0.022±0.001***	0.022±0.001	0.038±0.001	**0.038±0.001***

Table 203: Dataset GSE61258 (D32). Imputation performance on **MAR** type missing values.

	MAE		RMSE	
Method	M-value	B-value	M-value	B-value
mean	0.039±0.001*	0.040±0.001	0.070±0.002	0.069±0.001*
softImpute	**0.032±0.001***	0.036±0.004	**0.059±0.002***	0.063±0.011
impute.knn	0.038±0.004*	0.049±0.007	0.075±0.011*	0.096±0.014
imputePCA	0.038±0.001*	0.039±0.001	0.071±0.002	0.070±0.001*
SVDmiss	0.033±0.001*	0.034±0.001	0.064±0.002	0.064±0.002
missForest	0.036±0.001*	0.037±0.001	0.067±0.001	0.065±0.001*
methyLImp	0.034±0.001*	0.034±0.001	0.064±0.002*	0.064±0.002

Table 204: Dataset GSE61258 (D32). Imputation performance on **MNAR:low** type missing values.

	MAE		RMSE	
Method	M-value	B-value	M-value	B-value
mean	0.019±0.001*	0.019±0.001	0.036±0.001*	0.037±0.001
softImpute	0.018±0.001*	0.018±0.001	0.033±0.001*	0.034±0.002
impute.knn	0.034±0.011	0.026±0.005*	0.086±0.025	0.068±0.015*
imputePCA	0.018±0.001*	0.019±0.001	0.035±0.002*	0.036±0.001
SVDmiss	0.018±0.001*	0.018±0.001	0.034±0.002*	0.035±0.002
missForest	0.018±0.001*	0.018±0.001	0.033±0.002*	0.034±0.002
methyLImp	**0.016±0.001***	0.017±0.001	**0.031±0.002***	0.031±0.002

Table 205: Dataset GSE61258 (D32). Imputation performance on **MNAR:mid** type missing values.

	MAE		RMSE	
Method	M-value	B-value	M-value	B-value
mean	0.042±0.001	0.042±0.001*	0.066±0.001	0.063±0.001*
softImpute	0.041±0.001	0.040±0.001*	0.060±0.001	0.058±0.001*
impute.knn	**0.035±0.001***	0.041±0.004	0.057±0.005*	0.072±0.010
imputePCA	0.040±0.001	0.039±0.001*	0.064±0.001	0.060±0.001*
SVDmiss	0.038±0.001	0.037±0.001*	0.059±0.001	0.057±0.001*
missForest	0.040±0.001	0.039±0.001*	0.062±0.001	0.058±0.001*
methyLImp	0.036±0.001	0.036±0.001*	0.055±0.001	**0.054±0.001***

Table 206: Dataset GSE61258 (D32). Imputation performance on **MNAR:high** type missing values.

	MAE		RMSE	
Method	M-value	B-value	M-value	B-value
mean	0.021±0.001*	0.022±0.001	0.035±0.001*	0.036±0.001
softImpute	0.021±0.001*	0.022±0.003	0.035±0.001*	0.036±0.010
impute.knn	0.046±0.014*	0.082±0.019	0.107±0.031*	0.159±0.025
imputePCA	0.020±0.001*	0.021±0.001	0.034±0.001*	0.035±0.001
SVDmiss	0.021±0.001*	0.022±0.001	0.036±0.002*	0.036±0.002
missForest	0.020±0.001*	0.021±0.001	0.035±0.001	0.035±0.001
methyLImp	**0.019±0.001***	0.020±0.001	**0.032±0.001***	0.032±0.001

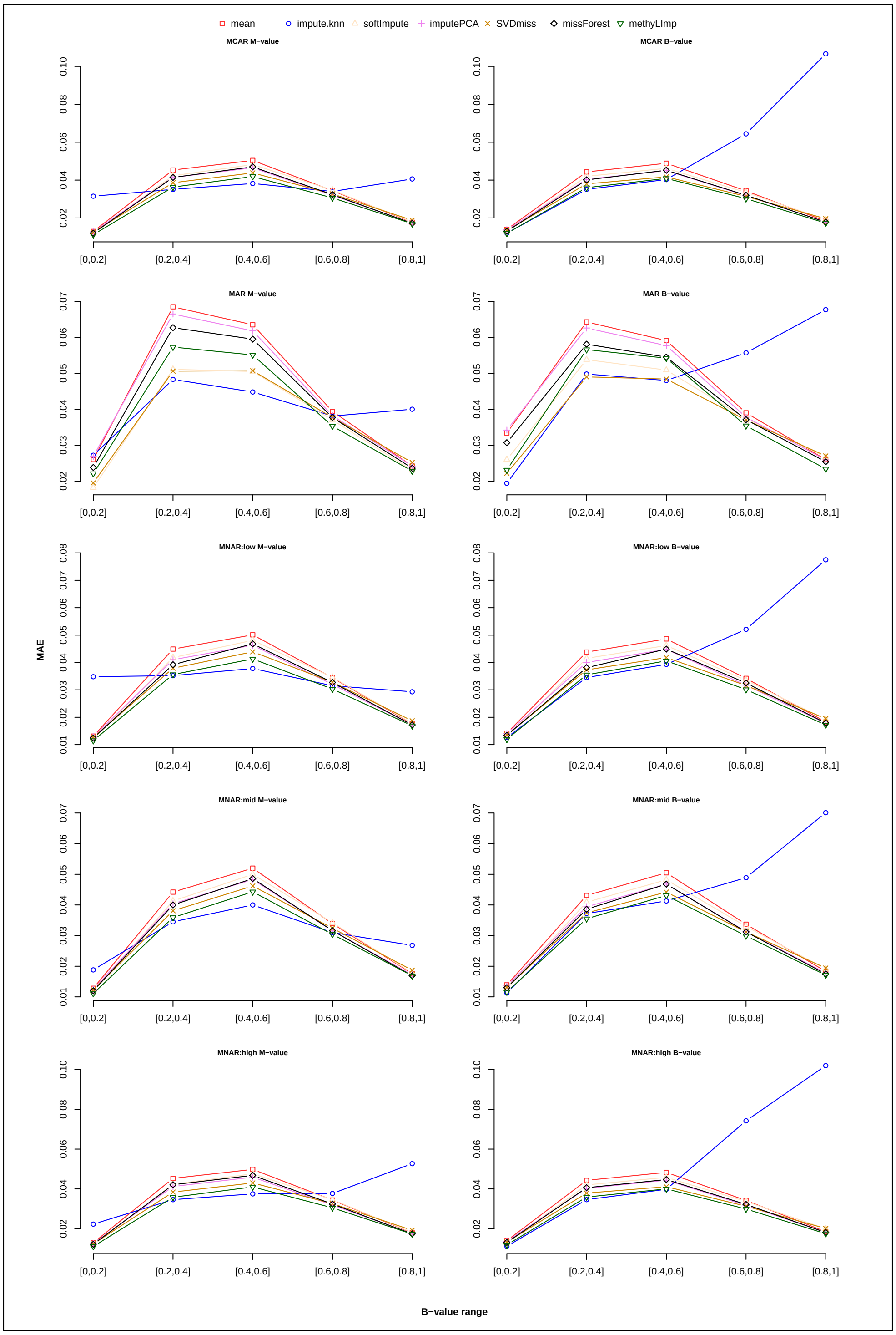

Figure 67: Dataset GSE61258 (D32). MAE imputation performances with respect to B-value range.

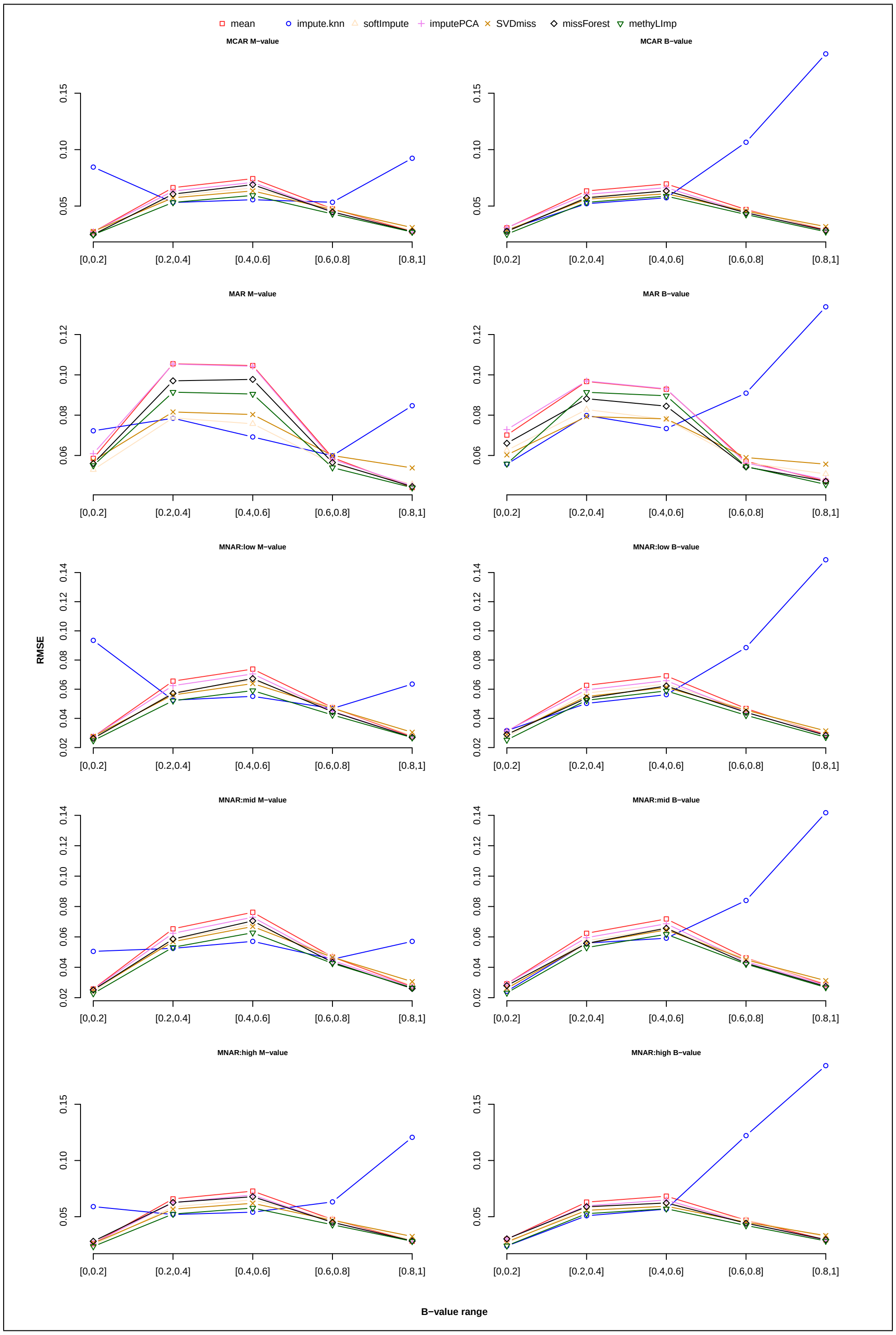

Figure 68: Dataset GSE61258 (D32). RMSE imputation performances with respect to B-value range.

2.33 GSE61259 (D33) - Muscle - Non-alcoholic fatty liver disease (NAFLD) - 9 samples

Method	Avg time (sec)	Avg RAM (Mb)
mean	< 1	8
softImpute	< 1	40
imputePCA	5	154
impute.knn	< 1	43
SVDmiss	16	3890
methyLImp	5	126
missForest	2060	285

Table 207: Dataset GSE61259 (D33). Average time and memory usage.

Table 208: Dataset GSE61259 (D33). Imputation performance on **MCAR** type missing values.

	MAE		RMSE	
Method	M-value	B-value	M-value	B-value
mean	0.031±0.001	0.030±0.001*	0.051±0.001	0.051±0.001*
softImpute	0.032±0.001	0.033±0.007	0.055±0.003*	0.061±0.018
impute.knn	0.044±0.010*	0.068±0.015	0.087±0.021*	0.130±0.022
imputePCA	0.029±0.001	0.029±0.001*	0.049±0.001	0.049±0.001*
SVDmiss	0.033±0.001	0.032±0.001*	0.056±0.002*	0.057±0.003
missForest	0.029±0.001	0.029±0.001*	0.050±0.001	0.049±0.001*
methyLImp	0.027±0.001	**0.027±0.001***	0.046±0.001	**0.045±0.002***

Table 209: Dataset GSE61259 (D33). Imputation performance on **MAR** type missing values.

	MAE		RMSE	
Method	M-value	B-value	M-value	B-value
mean	0.044±0.001	0.043±0.001*	0.076±0.002	0.074±0.002*
softImpute	0.046±0.004*	0.049±0.010	0.084±0.011*	0.094±0.029
impute.knn	0.049±0.005*	0.070±0.012	0.091±0.010*	0.125±0.019
imputePCA	0.042±0.001	0.041±0.001*	0.074±0.002	0.073±0.002*
SVDmiss	0.048±0.001*	0.048±0.001	0.085±0.002*	0.092±0.004
missForest	0.043±0.001	0.042±0.001*	0.074±0.002	**0.072±0.002***
methyLImp	**0.040±0.001***	0.041±0.001	0.073±0.002*	0.075±0.002

Table 210: Dataset GSE61259 (D33). Imputation performance on **MNAR:low** type missing values.

	MAE		RMSE	
Method	M-value	B-value	M-value	B-value
mean	0.022±0.001*	0.022±0.001	0.043±0.002*	0.043±0.002
softImpute	0.023±0.001*	0.024±0.003	0.044±0.003*	0.050±0.010
impute.knn	0.054±0.014	0.038±0.008*	0.114±0.022	0.091±0.018*
imputePCA	0.021±0.001*	0.021±0.001	0.042±0.002*	0.042±0.002
SVDmiss	0.024±0.001*	0.024±0.001	0.046±0.002*	0.048±0.003
missForest	0.021±0.001*	0.021±0.001	0.042±0.002*	0.042±0.002
methyLImp	**0.019±0.001***	0.020±0.001	**0.037±0.002***	0.038±0.002

Table 211: Dataset GSE61259 (D33). Imputation performance on **MNAR:mid** type missing values.

	MAE		RMSE	
Method	M-value	B-value	M-value	B-value
mean	0.048±0.001	0.046±0.001*	0.070±0.001	0.066±0.001*
softImpute	0.047±0.001	0.047±0.006*	0.071±0.002*	0.076±0.014
impute.knn	**0.039±0.002***	0.052±0.006	0.061±0.005*	0.090±0.013
imputePCA	0.044±0.001	0.043±0.001*	0.065±0.001	0.063±0.001*
SVDmiss	0.047±0.001	0.044±0.001*	0.072±0.002	0.071±0.003*
missForest	0.045±0.001	0.044±0.001*	0.067±0.001	0.063±0.001*
methyLImp	0.040±0.001	**0.039±0.001***	0.060±0.001	**0.059±0.001***

Table 212: Dataset GSE61259 (D33). Imputation performance on **MNAR:high** type missing values.

	MAE		RMSE	
Method	M-value	B-value	M-value	B-value
mean	0.026±0.001*	0.026±0.001	0.044±0.001*	0.044±0.001
softImpute	0.029±0.002*	0.035±0.012	0.052±0.005*	0.069±0.036
impute.knn	0.076±0.021*	0.127±0.031	0.154±0.033*	0.210±0.031
imputePCA	0.025±0.001*	0.025±0.001	0.042±0.001*	0.043±0.001
SVDmiss	0.030±0.001*	0.031±0.001	0.052±0.002*	0.053±0.003
missForest	0.025±0.001*	0.025±0.001	0.043±0.001*	0.043±0.001
methyLImp	**0.024±0.001***	0.024±0.001	**0.040±0.001***	0.041±0.002

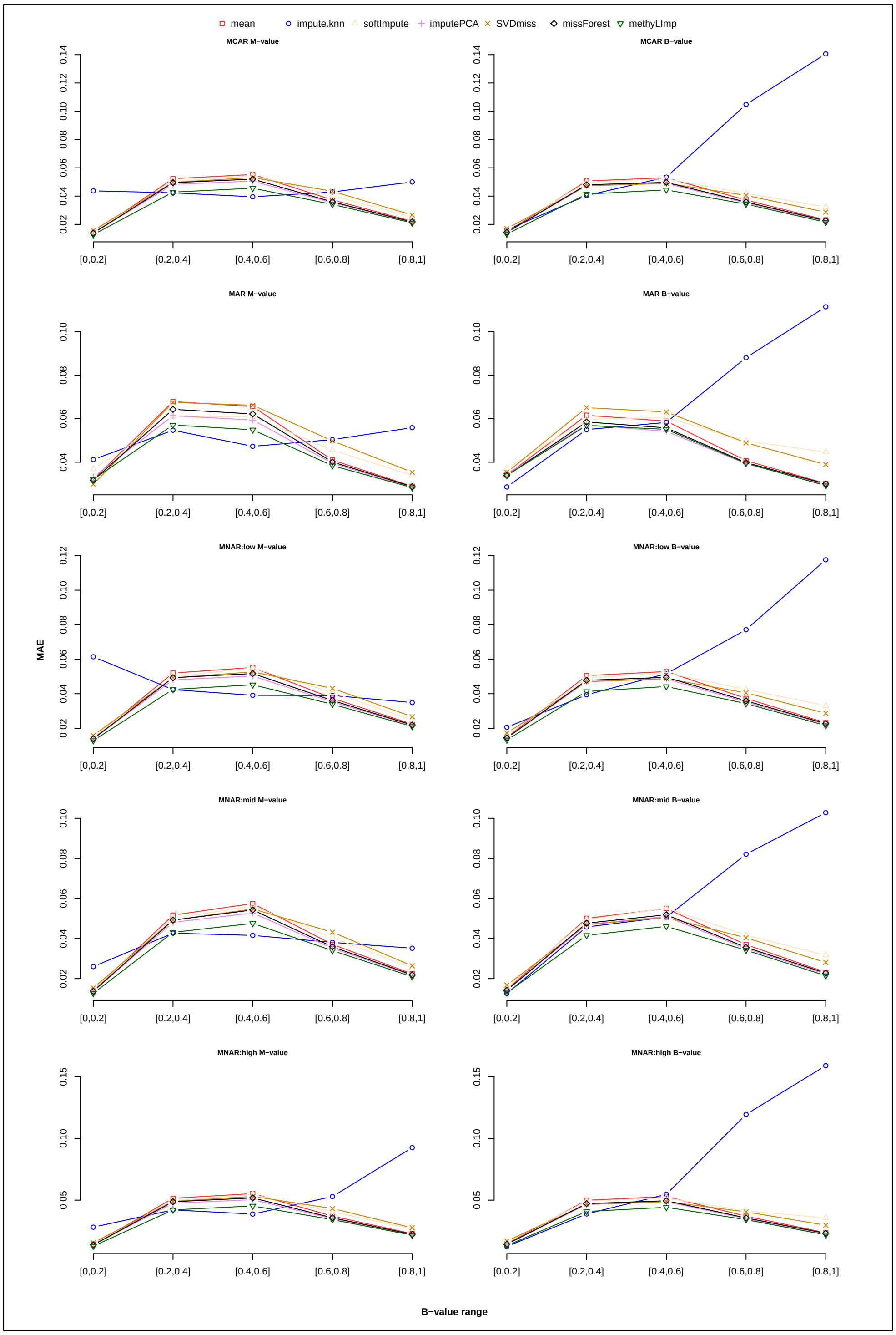

Figure 69: Dataset GSE61259 (D33). MAE imputation performances with respect to B-value range.

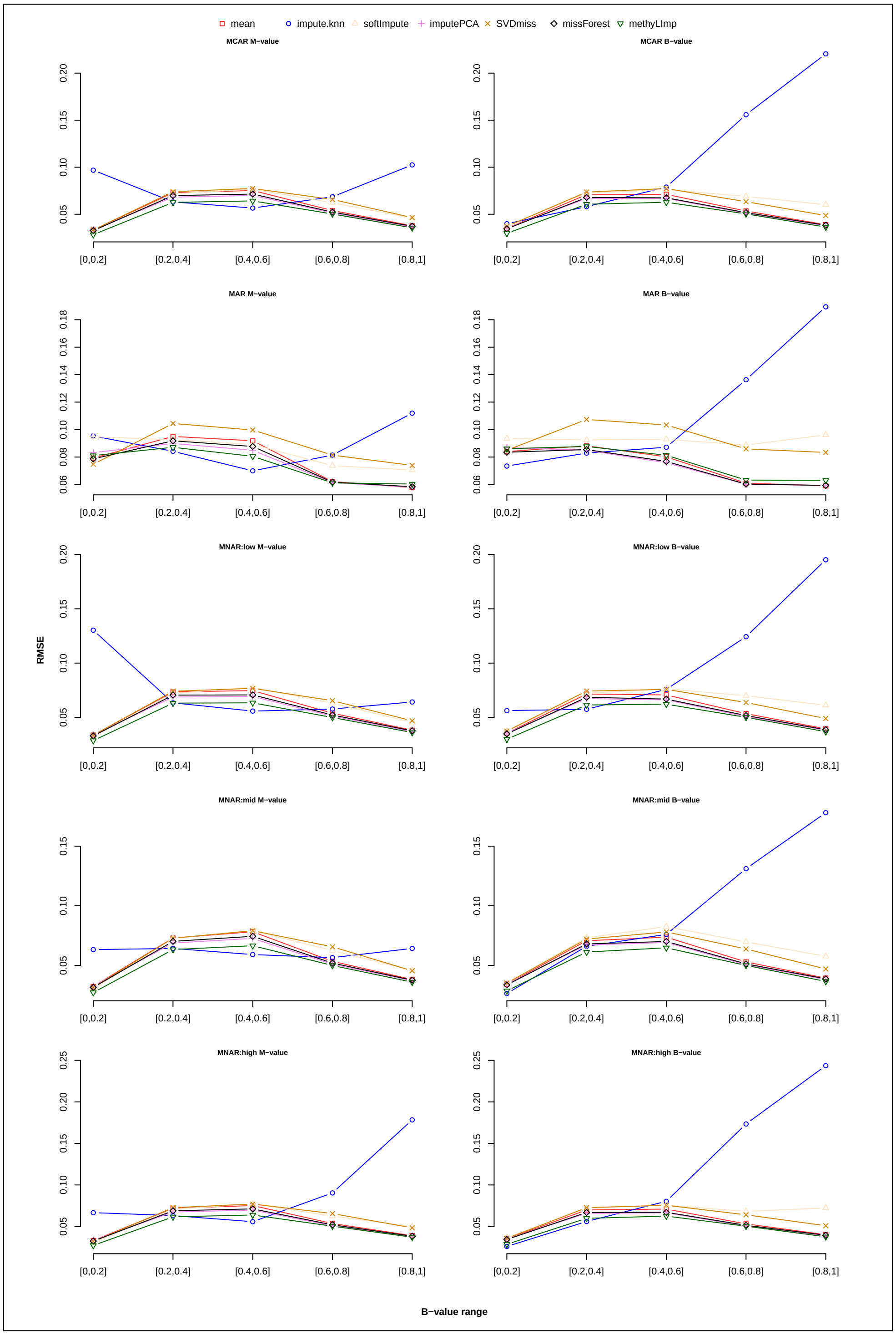

Figure 70: Dataset GSE61259 (D33). RMSE imputation performances with respect to B-value range.

2.34 GSE61259 (D34) - Muscle - Non-alcoholic steatohepatitis (NASH) - 7 samples

Method	Avg time (sec)	Avg RAM (Mb)
mean	< 1	6
softImpute	< 1	41
imputePCA	5	140
impute.knn	< 1	41
SVDmiss	12	3808
methyLImp	3	127
missForest	501	272

Table 213: Dataset GSE61259 (D34). Average time and memory usage.

Table 214: Dataset GSE61259 (D34). Imputation performance on **MCAR** type missing values.

	MAE		RMSE	
Method	M-value	B-value	M-value	B-value
mean	0.025±0.001	0.025±0.001*	0.042±0.002	0.041±0.002*
softImpute	0.031±0.009	0.040±0.025	0.054±0.023	0.083±0.073
impute.knn	0.047±0.011*	0.070±0.018	0.095±0.021*	0.134±0.025
imputePCA	0.024±0.001	0.024±0.001*	0.040±0.002	0.040±0.002*
SVDmiss	0.033±0.001*	0.033±0.001	0.056±0.002*	0.059±0.004
missForest	0.024±0.001	0.024±0.001*	0.041±0.002	0.041±0.002*
methyLImp	**0.023±0.001***	0.023±0.001	0.040±0.002	**0.040±0.002***

Table 215: Dataset GSE61259 (D34). Imputation performance on **MAR** type missing values.

	MAE		RMSE	
Method	M-value	B-value	M-value	B-value
mean	0.037±0.001	0.036±0.001*	0.068±0.002	**0.065±0.002***
softImpute	0.042±0.004*	0.052±0.022	0.077±0.009*	0.104±0.059
impute.knn	0.055±0.008*	0.081±0.014	0.105±0.014*	0.144±0.018
imputePCA	0.037±0.001	0.036±0.001*	0.068±0.002	**0.065±0.002***
SVDmiss	0.048±0.001*	0.049±0.001	0.087±0.003*	0.096±0.005
missForest	0.038±0.001	0.036±0.001*	0.069±0.002	0.066±0.002*
methyLImp	**0.036±0.001***	0.036±0.001	0.067±0.002*	0.067±0.002

Table 216: Dataset GSE61259 (D34). Imputation performance on **MNAR:low** type missing values.

	MAE		RMSE	
Method	M-value	B-value	M-value	B-value
mean	0.018±0.001*	0.018±0.001	0.036±0.002	0.036±0.002
softImpute	0.027±0.015	0.025±0.011*	0.055±0.036	0.055±0.038*
impute.knn	0.067±0.022	0.044±0.013*	0.131±0.029	0.103±0.023*
imputePCA	0.018±0.001*	0.018±0.001	0.035±0.002	0.035±0.002*
SVDmiss	0.025±0.001*	0.025±0.001	0.047±0.003*	0.049±0.004
missForest	0.018±0.001*	0.018±0.001	0.036±0.002	0.036±0.002*
methyLImp	**0.017±0.001***	0.018±0.001	**0.035±0.002***	0.035±0.002

Table 217: Dataset GSE61259 (D34). Imputation performance on **MNAR:mid** type missing values.

	MAE		RMSE	
Method	M-value	B-value	M-value	B-value
mean	0.037±0.001	0.036±0.001*	0.055±0.001	0.052±0.001*
softImpute	0.041±0.002*	0.055±0.025	0.062±0.004*	0.100±0.063
impute.knn	0.037±0.003*	0.056±0.010	0.059±0.007*	0.099±0.020
imputePCA	0.035±0.001	0.034±0.001*	0.053±0.001	**0.050±0.001***
SVDmiss	0.045±0.001	0.044±0.001*	0.069±0.002*	0.072±0.003
missForest	0.037±0.001	0.035±0.001*	0.055±0.001	0.052±0.001*
methyLImp	0.034±0.001	**0.034±0.001***	0.051±0.001	**0.050±0.001***

Table 218: Dataset GSE61259 (D34). Imputation performance on **MNAR:high** type missing values.

	MAE		RMSE	
Method	M-value	B-value	M-value	B-value
mean	0.021±0.001*	0.021±0.001	0.036±0.002	0.036±0.002
softImpute	0.029±0.013*	0.059±0.035	0.054±0.032*	0.136±0.096
impute.knn	0.099±0.027*	0.154±0.033	0.186±0.035*	0.237±0.031
imputePCA	0.021±0.001*	0.021±0.001	0.035±0.002*	0.035±0.002
SVDmiss	0.031±0.001*	0.032±0.001	0.054±0.003*	0.057±0.004
missForest	0.021±0.001*	0.021±0.001	0.036±0.002	0.036±0.002
methyLImp	0.021±0.001	**0.020±0.001***	0.035±0.002	**0.035±0.002***

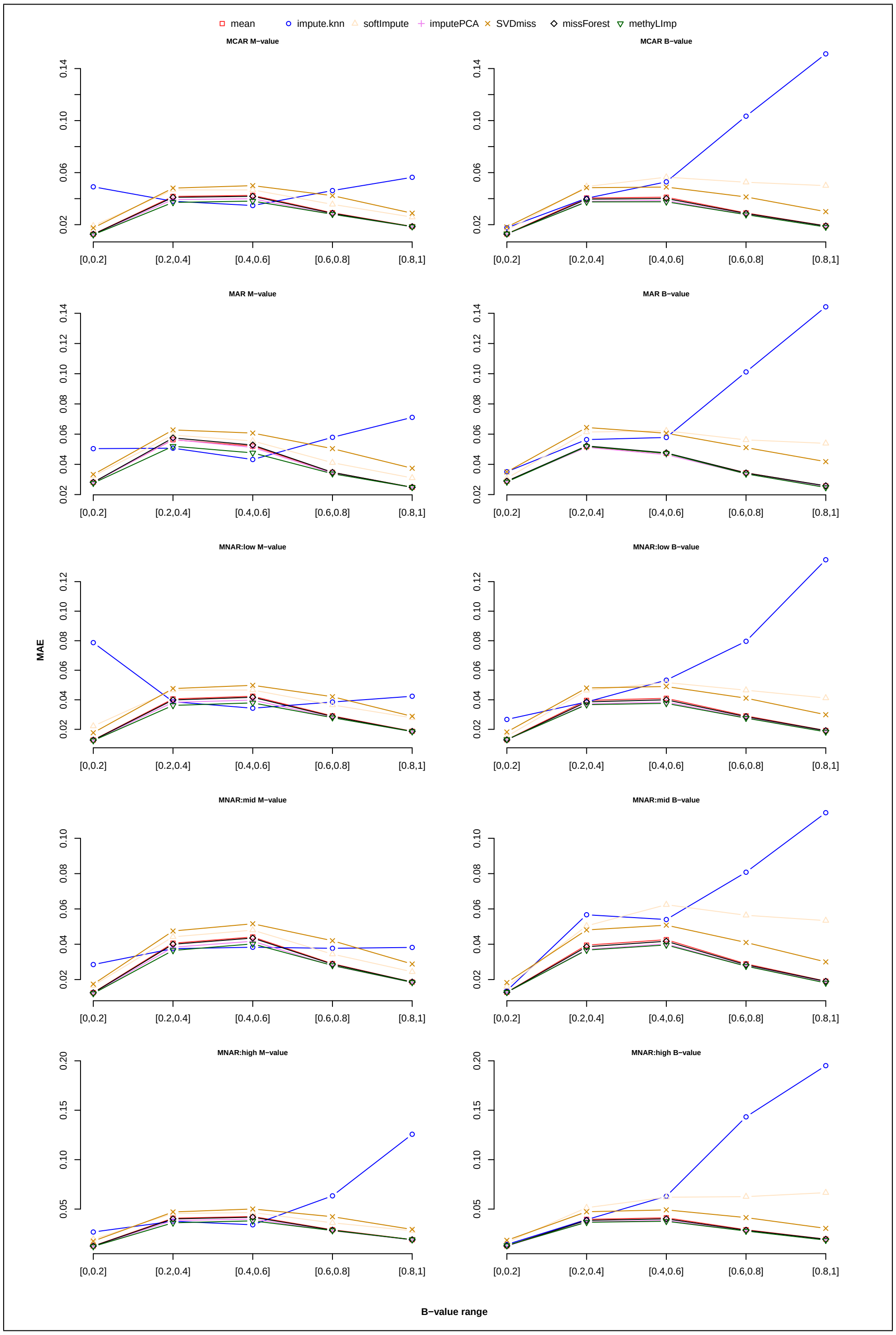

Figure 71: Dataset GSE61259 (D34). MAE imputation performances with respect to B-value range.

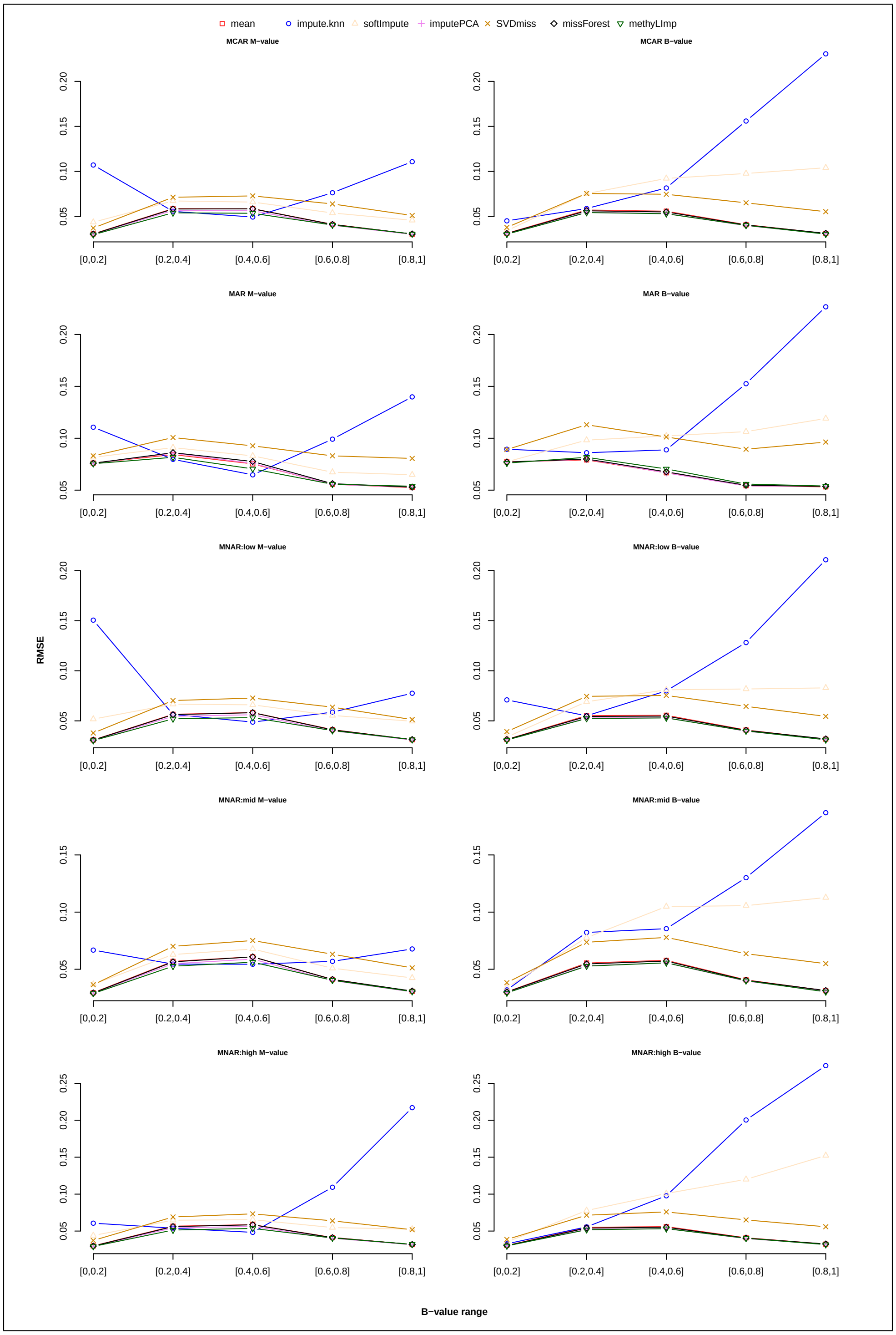

Figure 72: Dataset GSE61259 (D34). RMSE imputation performances with respect to B-value range.

2.35 GSE61259 (D35) - Muscle - Normal - 10 samples

Method	Avg time (sec)	Avg RAM (Mb)
mean	< 1	9
softImpute	< 1	41
imputePCA	10	155
impute.knn	< 1	48
SVDmiss	19	3931
methyLImp	7	120
missForest	2943	287

Table 219: Dataset GSE61259 (D35). Average time and memory usage.

Table 220: Dataset GSE61259 (D35). Imputation performance on **MCAR** type missing values.

	MAE		RMSE	
Method	M-value	B-value	M-value	B-value
mean	0.038±0.001	0.038±0.001*	0.068±0.002	0.067±0.001*
softImpute	0.031±0.001*	0.032±0.002	0.055±0.002*	0.058±0.004
impute.knn	0.045±0.009*	0.064±0.013	0.091±0.017*	0.129±0.020
imputePCA	0.030±0.001	0.030±0.001*	0.054±0.001	0.054±0.001*
SVDmiss	0.035±0.001	0.034±0.001*	0.064±0.002	0.062±0.002*
missForest	0.033±0.001	0.032±0.001*	0.059±0.002	0.058±0.001*
methyLImp	**0.028±0.001***	0.028±0.001	0.049±0.001	**0.049±0.001***

Table 221: Dataset GSE61259 (D35). Imputation performance on **MAR** type missing values.

	MAE		RMSE	
Method	M-value	B-value	M-value	B-value
mean	0.050±0.001	0.048±0.001*	0.084±0.002	0.082±0.002*
softImpute	0.047±0.001*	0.049±0.002	0.084±0.002*	0.090±0.007
impute.knn	0.049±0.004*	0.072±0.009	0.091±0.008*	0.131±0.014
imputePCA	0.045±0.001	0.043±0.001*	0.080±0.002	**0.077±0.002***
SVDmiss	0.048±0.001	0.047±0.001*	0.088±0.002*	0.089±0.003
missForest	0.047±0.001	0.045±0.001*	0.081±0.002	0.079±0.002*
methyLImp	**0.043±0.001***	0.045±0.001	0.080±0.002*	0.083±0.003

Table 222: Dataset GSE61259 (D35). Imputation performance on **MNAR:low** type missing values.

	MAE		RMSE	
Method	M-value	B-value	M-value	B-value
mean	0.027±0.001*	0.027±0.001	0.057±0.002*	0.057±0.002
softImpute	0.024±0.001	0.024±0.001	0.050±0.002*	0.051±0.003
impute.knn	0.053±0.015	0.037±0.008*	0.113±0.024	0.089±0.016*
imputePCA	0.022±0.001*	0.022±0.001	0.047±0.002*	0.048±0.002
SVDmiss	0.027±0.001	0.026±0.001*	0.055±0.002	0.052±0.003*
missForest	0.024±0.001*	0.024±0.001	0.051±0.002*	0.051±0.002
methyLImp	**0.021±0.001***	0.021±0.001	**0.042±0.002***	0.042±0.002

Table 223: Dataset GSE61259 (D35). Imputation performance on **MNAR:mid** type missing values.

	MAE		RMSE	
Method	M-value	B-value	M-value	B-value
mean	0.059±0.001	0.055±0.001*	0.084±0.001	0.078±0.001*
softImpute	0.043±0.001	0.042±0.001*	0.067±0.002	0.067±0.003
impute.knn	0.039±0.002*	0.051±0.005	0.066±0.005*	0.091±0.009
imputePCA	0.043±0.001	0.041±0.001*	0.067±0.001	0.063±0.001*
SVDmiss	0.048±0.001	0.043±0.001*	0.075±0.002	0.072±0.003*
missForest	0.047±0.001	0.044±0.001*	0.072±0.001	0.066±0.001*
methyLImp	0.039±0.001	**0.038±0.001***	0.060±0.001	**0.059±0.001***

Table 224: Dataset GSE61259 (D35). Imputation performance on **MNAR:high** type missing values.

	MAE		RMSE	
Method	M-value	B-value	M-value	B-value
mean	0.035±0.001*	0.036±0.001	0.068±0.002*	0.069±0.002
softImpute	0.030±0.001*	0.034±0.003	0.054±0.003*	0.065±0.009
impute.knn	0.075±0.019*	0.133±0.027	0.149±0.030*	0.219±0.028
imputePCA	0.028±0.001*	0.029±0.001	0.052±0.002*	0.053±0.002
SVDmiss	0.035±0.001*	0.035±0.001	0.064±0.002	0.063±0.003*
missForest	0.031±0.001*	0.032±0.001	0.059±0.002*	0.060±0.002
methyLImp	**0.027±0.001**	**0.027±0.001**	0.048±0.002	**0.048±0.002***

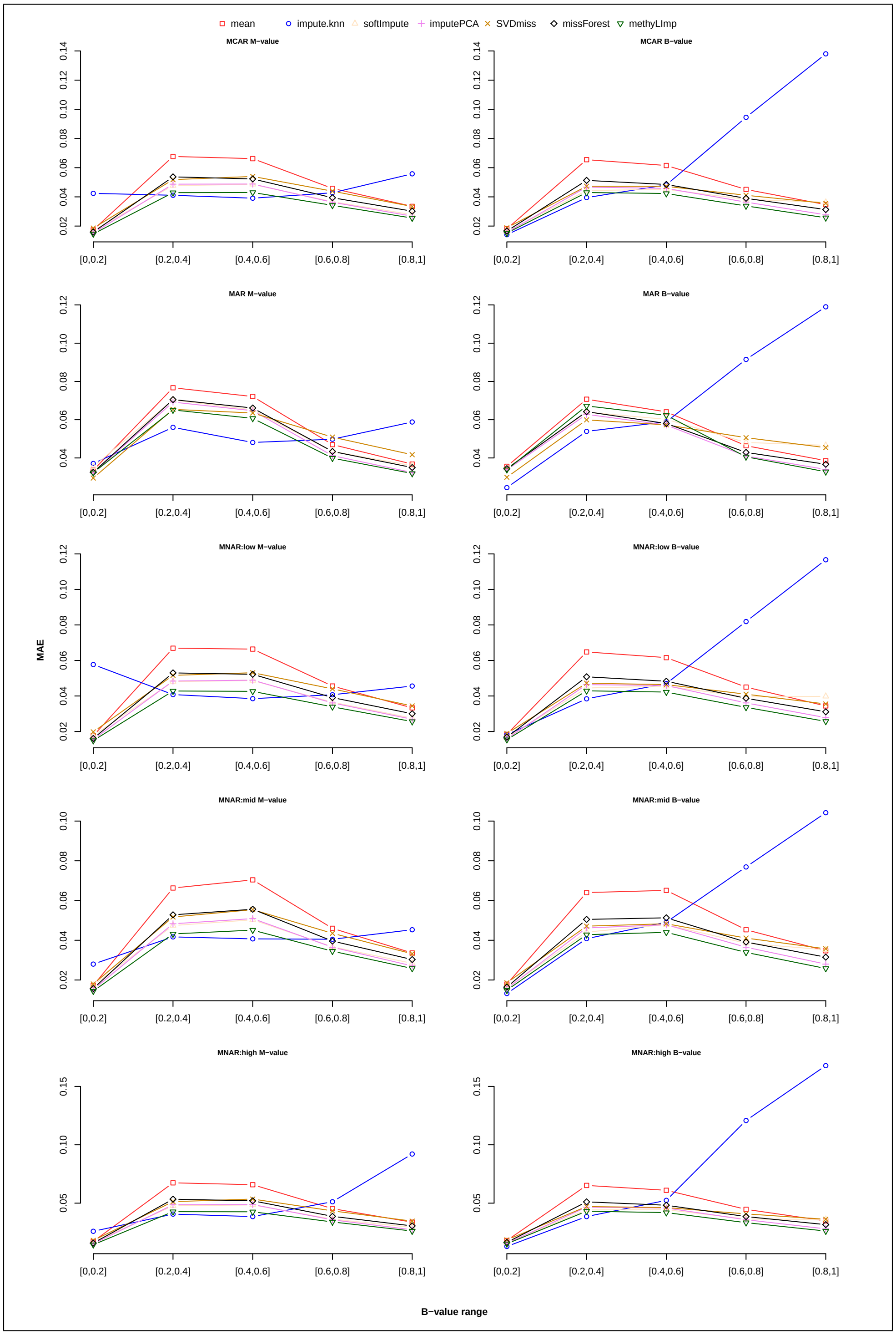

Figure 73: Dataset GSE61259 (D35). MAE imputation performances with respect to B-value range.

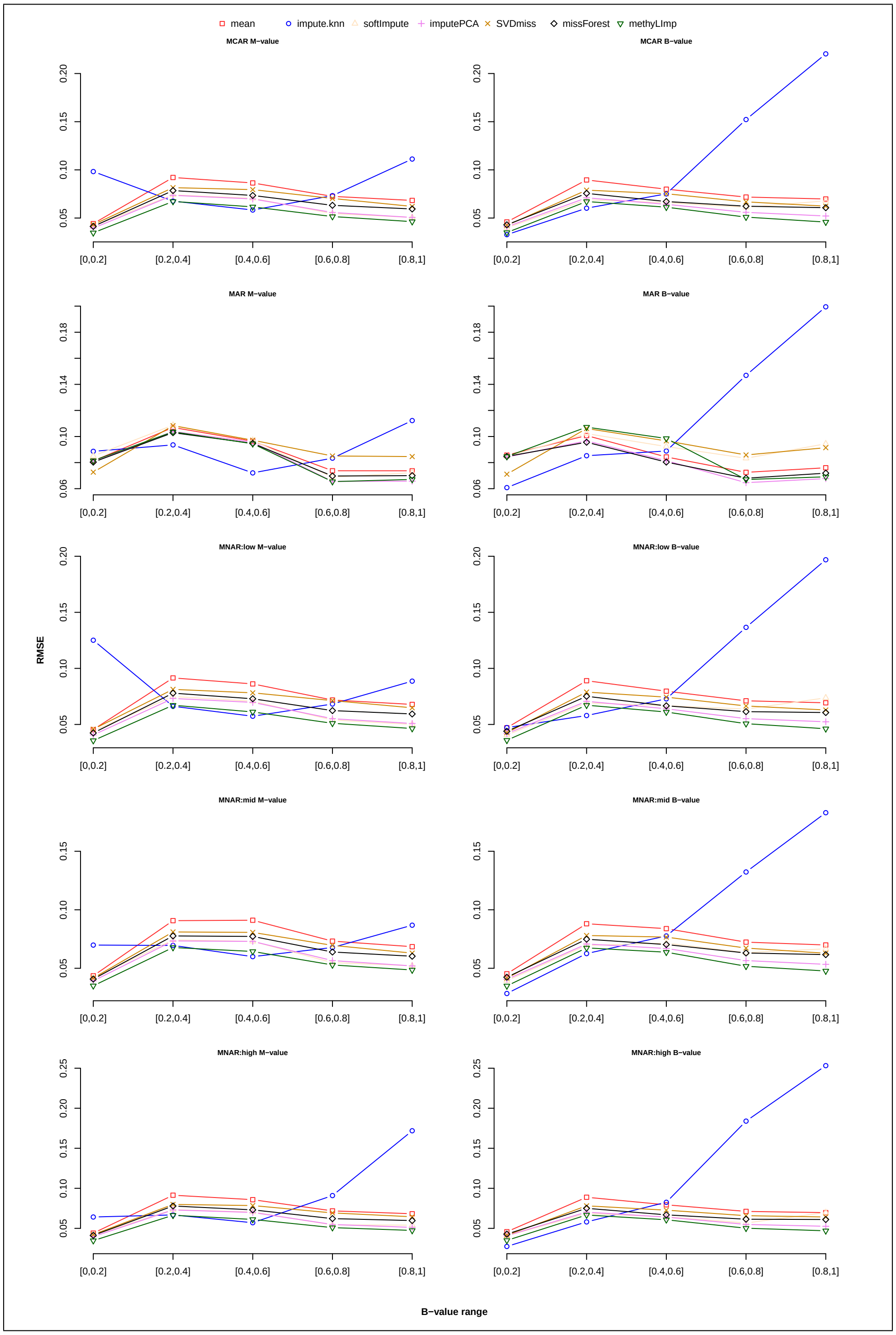

Figure 74: Dataset GSE61259 (D35). RMSE imputation performances with respect to B-value range.

2.36 GSE61380 (D36) - Brain - Normal - 15 samples

Method	Avg time (sec)	Avg RAM (Mb)
mean	< 1	13
softImpute	< 1	48
imputePCA	5	160
impute.knn	< 1	51
SVDmiss	34	3572
methyLImp	26	98
missForest	4485	114

Table 225: Dataset GSE61380 (D36). Average time and memory usage.

Table 226: Dataset GSE61380 (D36). Imputation performance on **MCAR** type missing values.

	MAE		RMSE	
Method	M-value	B-value	M-value	B-value
mean	0.025±0.001*	0.025±0.001	0.038±0.001	0.038±0.001
softImpute	0.025±0.001	0.026±0.004*	0.037±0.001	0.039±0.014*
impute.knn	0.035±0.007*	0.047±0.009	0.072±0.019*	0.096±0.017
imputePCA	0.024±0.001*	0.024±0.001	0.036±0.001	0.036±0.001*
SVDmiss	0.027±0.001*	0.027±0.001	0.041±0.001	0.040±0.001*
missForest	0.024±0.001*	0.024±0.001	0.037±0.001	0.036±0.001*
methyLImp	**0.024±0.001***	0.024±0.001	**0.035±0.001**	**0.035±0.001**

Table 227: Dataset GSE61380 (D36). Imputation performance on **MAR** type missing values.

	MAE		RMSE	
Method	M-value	B-value	M-value	B-value
mean	0.030±0.001*	0.031±0.001	0.051±0.001*	0.051±0.001
softImpute	0.029±0.001*	0.031±0.001	**0.045±0.003***	0.050±0.001
impute.knn	0.033±0.003*	0.043±0.007	0.058±0.009*	0.081±0.015
imputePCA	0.029±0.001*	0.030±0.001	0.049±0.001	0.049±0.001*
SVDmiss	0.031±0.001*	0.032±0.001	0.048±0.001*	0.050±0.001
missForest	0.029±0.001*	0.031±0.001	0.049±0.001	0.049±0.001
methyLImp	**0.029±0.001***	0.029±0.001	0.048±0.001*	0.048±0.001

Table 228: Dataset GSE61380 (D36). Imputation performance on **MNAR:low** type missing values.

	MAE		RMSE	
Method	M-value	B-value	M-value	B-value
mean	0.018±0.001*	0.019±0.001	0.030±0.001*	0.030±0.001
softImpute	0.019±0.001*	0.019±0.001	0.029±0.001*	0.030±0.001
impute.knn	0.038±0.013	0.027±0.005*	0.090±0.027	0.062±0.014*
imputePCA	0.018±0.001*	0.018±0.001	0.028±0.001*	0.029±0.001
SVDmiss	0.020±0.001*	0.021±0.001	0.032±0.001*	0.033±0.001
missForest	0.018±0.001*	0.018±0.001	0.029±0.001*	0.029±0.001
methyLImp	**0.017±0.001***	0.018±0.001	**0.027±0.001***	0.027±0.001

Table 229: Dataset GSE61380 (D36). Imputation performance on **MNAR:mid** type missing values.

	MAE		RMSE	
Method	M-value	B-value	M-value	B-value
mean	0.038±0.001	0.038±0.001*	0.058±0.001	0.057±0.001*
softImpute	0.038±0.001	0.036±0.001*	0.054±0.001	0.053±0.002*
impute.knn	0.037±0.001*	0.039±0.002	0.056±0.005*	0.064±0.006
imputePCA	0.036±0.001	0.035±0.001*	0.054±0.001	0.053±0.001*
SVDmiss	0.039±0.001	0.039±0.001*	0.058±0.001	0.057±0.001*
missForest	0.037±0.001	0.036±0.001*	0.055±0.001	0.054±0.001*
methyLImp	0.035±0.001	**0.035±0.001***	0.051±0.001	**0.050±0.001***

Table 230: Dataset GSE61380 (D36). Imputation performance on **MNAR:high** type missing values.

	MAE		RMSE	
Method	M-value	B-value	M-value	B-value
mean	0.023±0.001*	0.023±0.001	0.033±0.001*	0.033±0.001
softImpute	0.023±0.001*	0.025±0.007	0.033±0.001	0.039±0.025
impute.knn	0.041±0.011*	0.080±0.021	0.089±0.026*	0.152±0.028
imputePCA	**0.022±0.001***	0.022±0.001	0.031±0.001*	0.032±0.001
SVDmiss	0.025±0.001*	0.025±0.001	0.037±0.001*	0.037±0.001
missForest	0.022±0.001*	0.023±0.001	0.032±0.001*	0.032±0.001
methyLImp	**0.022±0.001***	0.022±0.001	**0.031±0.001***	0.031±0.001

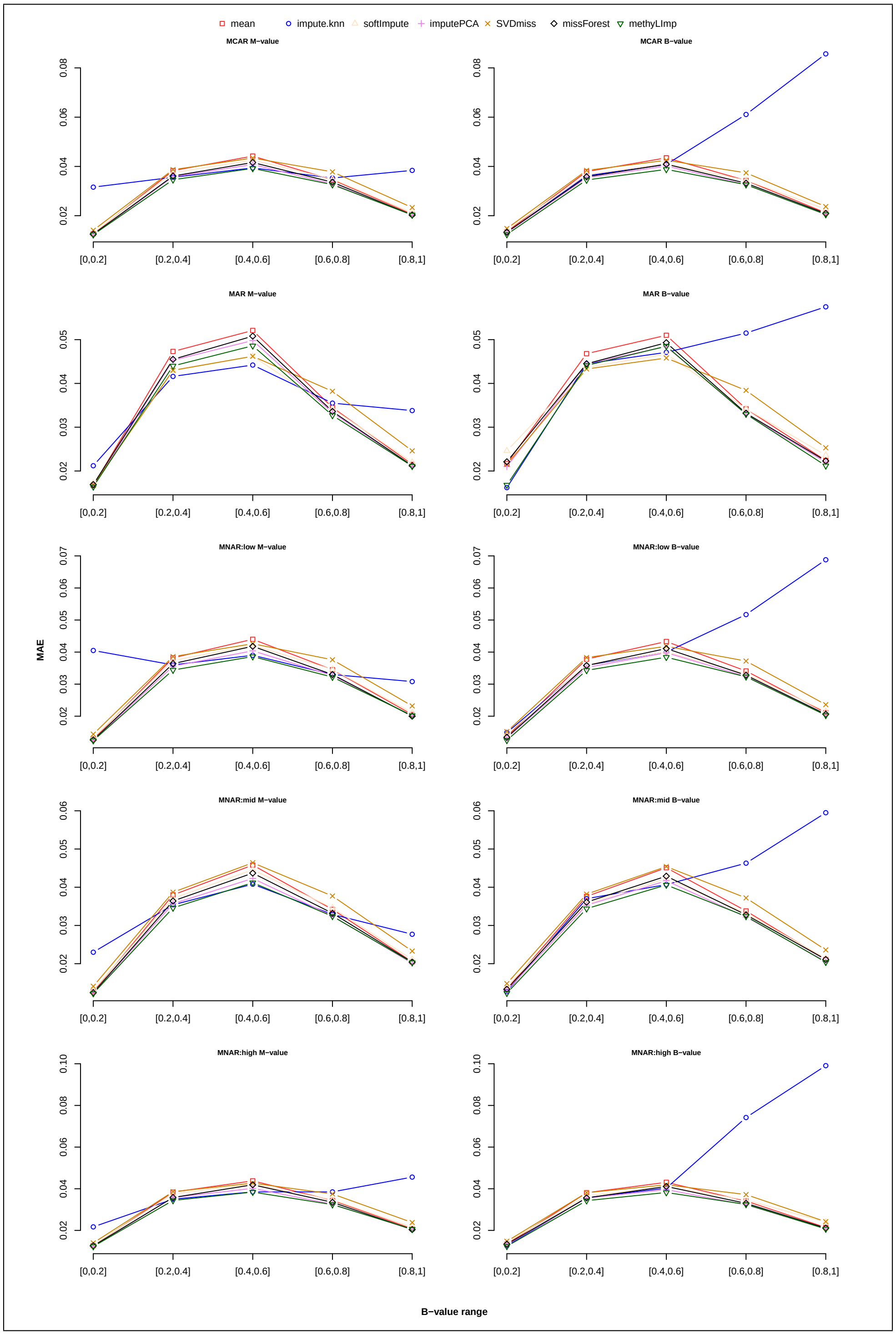

Figure 75: Dataset GSE61380 (D36). MAE imputation performances with respect to B-value range.

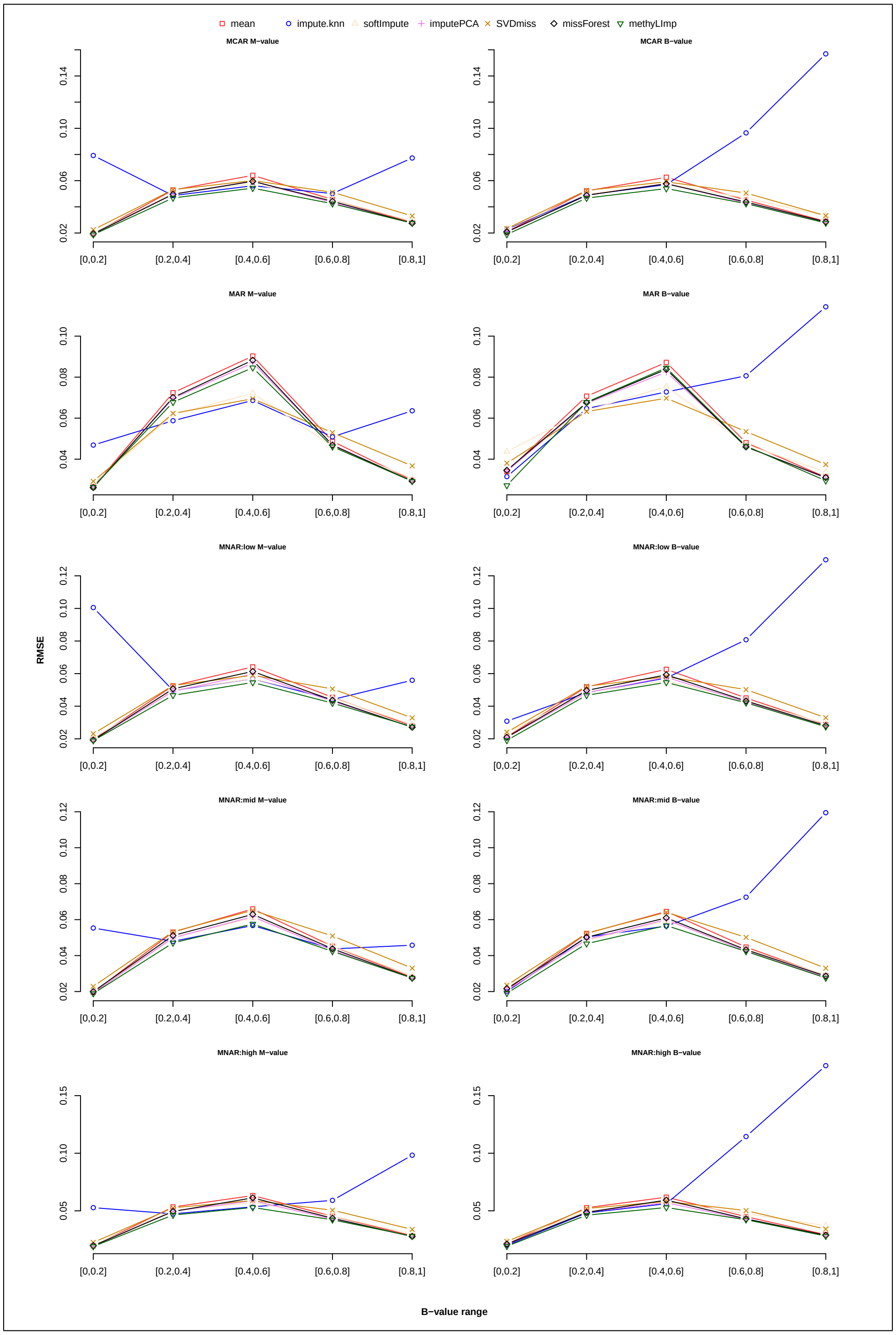

Figure 76: Dataset GSE61380 (D36). RMSE imputation performances with respect to B-value range.

2.37 GSE62003 (D37) - Blood - Normal - 35 samples

Method	Avg time (sec)	Avg RAM (Mb)
mean	< 1	29
softImpute	< 1	79
imputePCA	22	204
impute.knn	1	90
SVDmiss	144	4529
methyLImp	545	121
missForest	43825	216

Table 231: Dataset GSE62003 (D37). Average time and memory usage.

Table 232: Dataset GSE62003 (D37). Imputation performance on **MCAR** type missing values.

	MAE		RMSE	
Method	M-value	B-value	M-value	B-value
mean	0.025±0.001*	0.025±0.001	0.043±0.001	0.043±0.001*
softImpute	0.023±0.001*	0.024±0.001	0.039±0.001*	0.039±0.001
impute.knn	0.025±0.002*	0.032±0.005	0.051±0.008*	0.069±0.012
imputePCA	0.022±0.001*	0.023±0.001	0.041±0.001	0.041±0.001*
SVDmiss	0.021±0.001*	0.022±0.001	0.037±0.001	0.037±0.001
missForest	0.021±0.001*	0.021±0.001	0.035±0.001	0.035±0.001
methyLImp	**0.018±0.001***	0.018±0.001	**0.030±0.001***	0.030±0.001

Table 233: Dataset GSE62003 (D37). Imputation performance on **MAR** type missing values.

	MAE		RMSE	
Method	M-value	B-value	M-value	B-value
mean	0.038±0.001*	0.039±0.001	0.068±0.001	0.067±0.001*
softImpute	0.029±0.001*	0.031±0.002	0.052±0.001*	0.054±0.003
impute.knn	0.031±0.001*	0.034±0.002	0.057±0.004*	0.065±0.006
imputePCA	0.036±0.001*	0.037±0.001	0.066±0.001	0.066±0.001*
SVDmiss	0.028±0.001*	0.029±0.001	0.052±0.001*	0.052±0.001
missForest	0.027±0.001*	0.028±0.001	0.049±0.001	0.049±0.001*
methyLImp	0.028±0.001	**0.027±0.001***	0.049±0.001	**0.048±0.001***

Table 234: Dataset GSE62003 (D37). Imputation performance on **MNAR:low** type missing values.

	MAE		RMSE	
Method	M-value	B-value	M-value	B-value
mean	0.019±0.001*	0.020±0.001	0.036±0.001*	0.037±0.001
softImpute	0.017±0.001*	0.018±0.001	0.031±0.001*	0.032±0.001
impute.knn	0.018±0.002*	0.020±0.002	0.041±0.009*	0.047±0.007
imputePCA	0.017±0.001*	0.018±0.001	0.034±0.001*	0.036±0.001
SVDmiss	0.016±0.001*	0.017±0.001	0.030±0.001*	0.031±0.001
missForest	0.015±0.001*	0.016±0.001	0.028±0.001*	0.029±0.001
methyLImp	**0.014±0.001***	0.014±0.001	**0.024±0.001***	0.024±0.001

Table 235: Dataset GSE62003 (D37). Imputation performance on **MNAR:mid** type missing values.

	MAE		RMSE	
Method	M-value	B-value	M-value	B-value
mean	0.049±0.001	0.048±0.001*	0.078±0.001	0.073±0.001*
softImpute	0.043±0.001	0.043±0.001*	0.064±0.001	0.063±0.001*
impute.knn	0.038±0.001*	0.040±0.002	0.061±0.003*	0.065±0.004
imputePCA	0.045±0.001	0.043±0.001*	0.073±0.001	0.069±0.001*
SVDmiss	0.039±0.001	0.039±0.001*	0.062±0.001	0.060±0.001*
missForest	0.038±0.001	0.037±0.001*	0.059±0.001	0.057±0.001*
methyLImp	0.033±0.001	**0.033±0.001***	0.053±0.001	**0.051±0.001***

Table 236: Dataset GSE62003 (D37). Imputation performance on **MNAR:high** type missing values.

	MAE		RMSE	
Method	M-value	B-value	M-value	B-value
mean	0.019±0.001*	0.020±0.001	0.034±0.001*	0.034±0.001
softImpute	0.018±0.001*	0.019±0.001	0.031±0.001*	0.032±0.001
impute.knn	0.021±0.002*	0.036±0.008	0.046±0.009*	0.083±0.016
imputePCA	0.018±0.001*	0.018±0.001	0.032±0.001*	0.033±0.001
SVDmiss	0.024±0.006	0.018±0.001*	0.063±0.030	0.031±0.001*
missForest	0.017±0.001*	0.017±0.001	0.028±0.001*	0.029±0.001
methyLImp	**0.016±0.001***	0.016±0.001	**0.025±0.001***	0.025±0.001

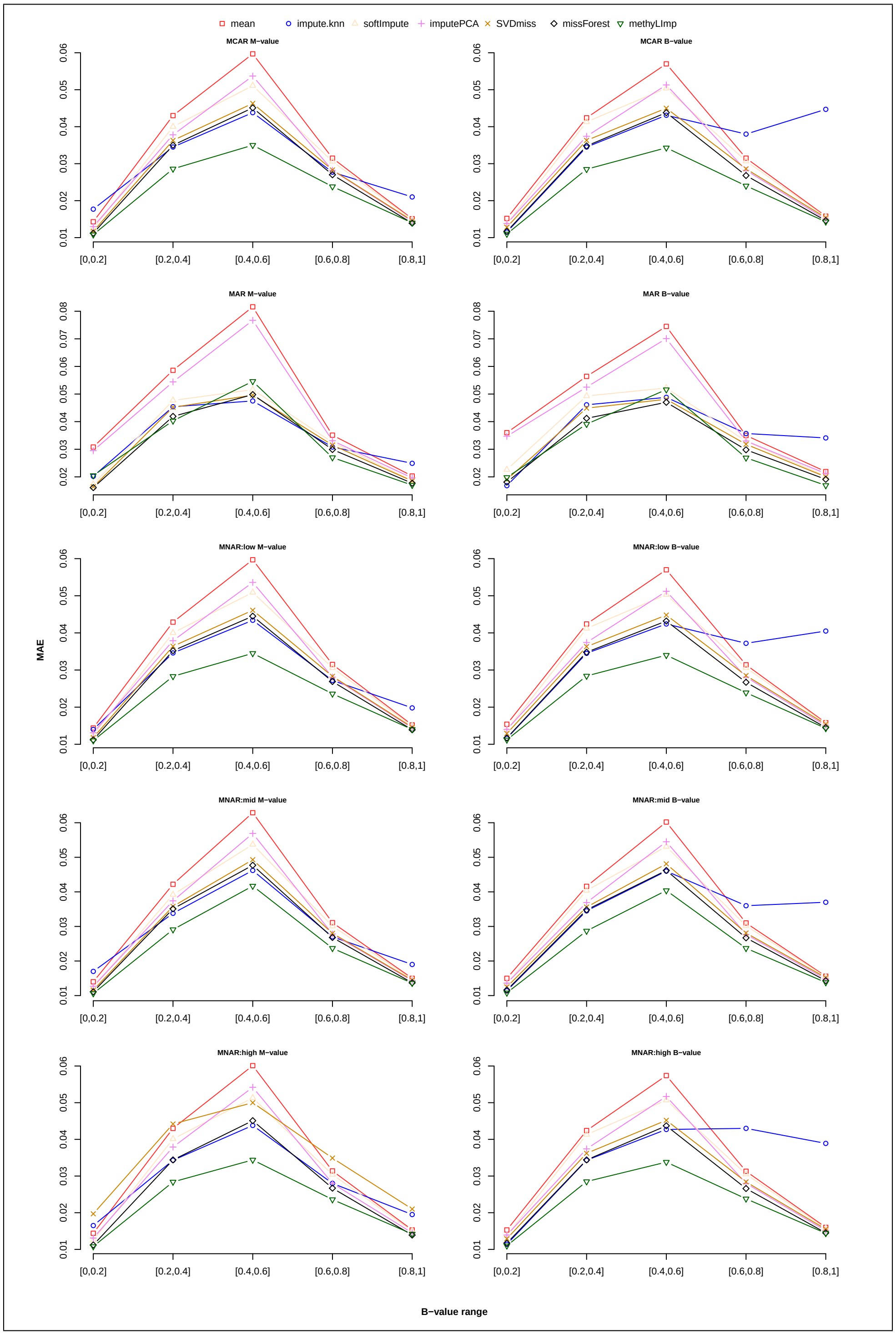

Figure 77: Dataset GSE62003 (D37). MAE imputation performances with respect to B-value range.

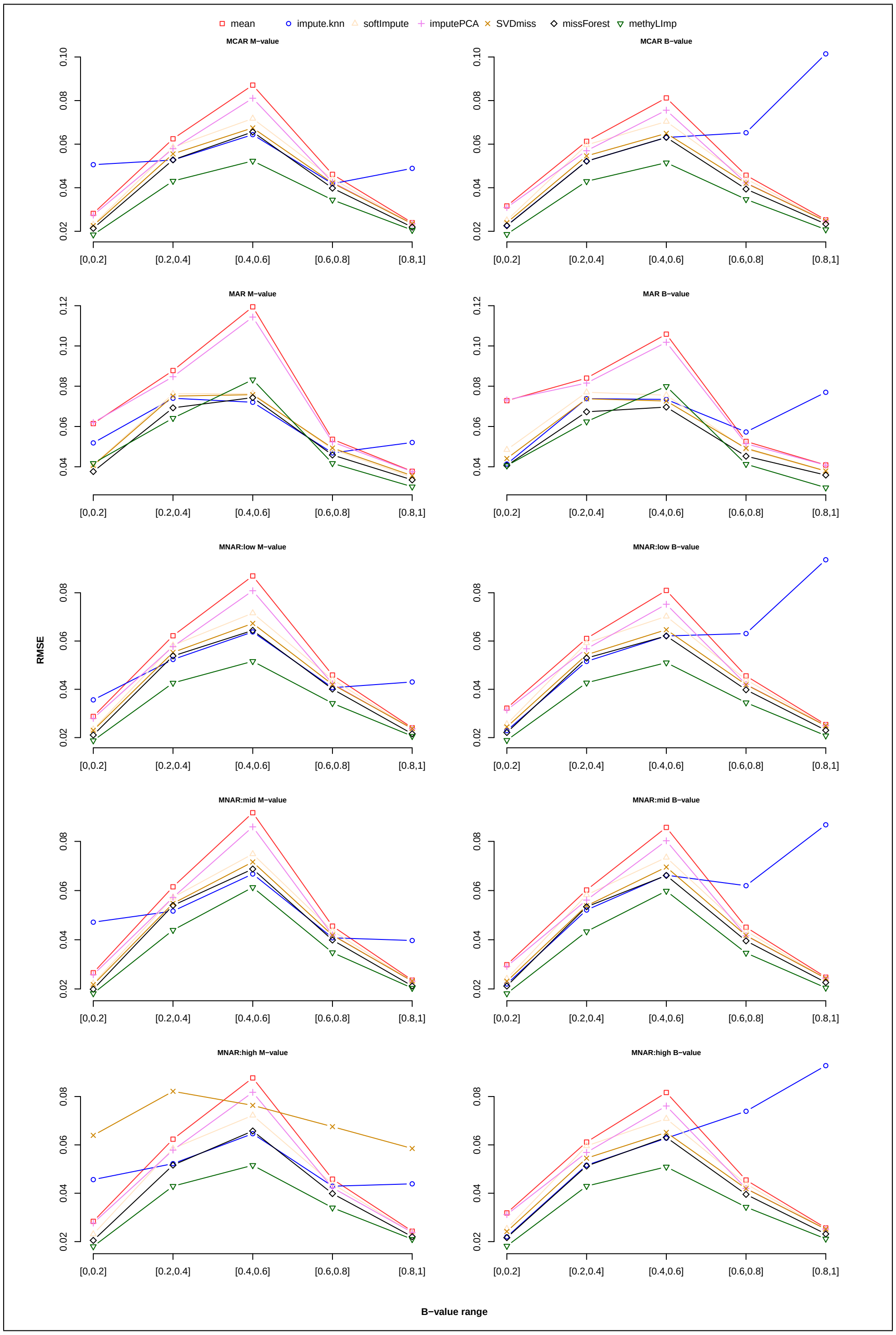

Figure 78: Dataset GSE62003 (D37). RMSE imputation performances with respect to B-value range.

2.38 GSE64495 (D38) - Blood - Normal - 106 samples

Method	Avg time (sec)	Avg RAM (Mb)
mean	< 1	86
softImpute	2	180
imputePCA	48	387
impute.knn	8	193
SVDmiss	550	4897
methyLImp	6870	186
missForest	309685	547

Table 237: Dataset GSE64495 (D38). Average time and memory usage.

Table 238: Dataset GSE64495 (D38). Imputation performance on **MCAR** type missing values.

	MAE		RMSE	
Method	M-value	B-value	M-value	B-value
mean	0.028±0.001	0.027±0.001*	0.051±0.001	0.047±0.001*
softImpute	0.033±0.001	0.024±0.001*	0.070±0.002	0.041±0.001*
impute.knn	0.023±0.001*	0.023±0.001	0.043±0.001*	0.048±0.005
imputePCA	0.025±0.001	0.024±0.001*	0.049±0.001	0.044±0.001*
SVDmiss	0.033±0.001	**0.022±0.001***	0.071±0.002	**0.038±0.001***
methyLImp	0.059±0.002	0.036±0.001*	0.143±0.005	0.095±0.002*
missForest	**0.022±0.001**	**0.021±0.001**	**0.042±0.001**	**0.038±0.001**

Table 239: Dataset GSE64495 (D38). Imputation performance on **MAR** type missing values.

	MAE		RMSE	
Method	M-value	B-value	M-value	B-value
mean	0.047±0.001	0.044±0.001*	0.092±0.001	0.082±0.001*
softImpute	0.051±0.001	0.033±0.001*	0.103±0.001	0.063±0.001*
impute.knn	0.035±0.001	0.035±0.002	0.075±0.002*	0.080±0.008
imputePCA	0.045±0.001	0.042±0.001*	0.092±0.001	0.080±0.001*
SVDmiss	0.052±0.001	0.031±0.001*	0.105±0.001	0.062±0.001*
methyLImp	0.053±0.001	0.041±0.001*	0.109±0.001	0.088±0.001*
missForest	0.033±0.001	**0.031±0.001***	0.073±0.001	**0.061±0.001***

Table 240: Dataset GSE64495 (D38). Imputation performance on **MNAR:low** type missing values.

	MAE		RMSE	
Method	M-value	B-value	M-value	B-value
mean	0.023±0.001	0.022±0.001*	0.045±0.001*	0.045±0.001
softImpute	0.028±0.001	0.020±0.001*	0.068±0.003	0.037±0.001*
impute.knn	0.018±0.001*	0.019±0.001	0.038±0.001*	0.044±0.004
imputePCA	0.021±0.001	0.020±0.001*	0.044±0.001	0.043±0.001*
SVDmiss	0.027±0.001	0.018±0.001*	0.062±0.001	0.035±0.001*
methyLImp	0.041±0.001	0.024±0.001*	0.108±0.004	0.064±0.002*
missForest	0.018±0.001	**0.017±0.001***	0.036±0.001	**0.034±0.001***

Table 241: Dataset GSE64495 (D38). Imputation performance on **MNAR:mid** type missing values.

	MAE		RMSE	
Method	M-value	B-value	M-value	B-value
mean	0.058±0.001	0.053±0.001*	0.094±0.001	0.078±0.001*
softImpute	0.060±0.001	0.044±0.001*	0.099±0.001	0.066±0.001*
impute.knn	0.041±0.001	0.040±0.001*	0.068±0.001	0.066±0.003*
imputePCA	0.052±0.001	0.047±0.001*	0.089±0.001	0.074±0.001*
SVDmiss	0.062±0.001	**0.039±0.001***	0.101±0.001	**0.060±0.001***
methyLImp	0.062±0.001	0.042±0.001*	0.101±0.001	0.072±0.001*
missForest	**0.041±0.001**	**0.038±0.001**	**0.068±0.001**	**0.059±0.001**

Table 242: Dataset GSE64495 (D38). Imputation performance on **MNAR:high** type missing values.

	MAE		RMSE	
Method	M-value	B-value	M-value	B-value
mean	0.025±0.001	0.024±0.001*	0.045±0.001	0.041±0.001*
softImpute	0.031±0.001	0.022±0.001*	0.068±0.002	0.037±0.001*
impute.knn	0.021±0.001*	0.022±0.001	0.040±0.001*	0.047±0.006
imputePCA	0.023±0.001	0.021±0.001*	0.044±0.001	0.039±0.001*
SVDmiss	0.031±0.001	**0.020±0.001***	0.072±0.002	**0.035±0.001***
methyLImp	0.052±0.001	0.031±0.001*	0.132±0.003	0.080±0.002*
missForest	**0.021±0.001**	**0.020±0.001**	**0.039±0.001**	**0.035±0.001**

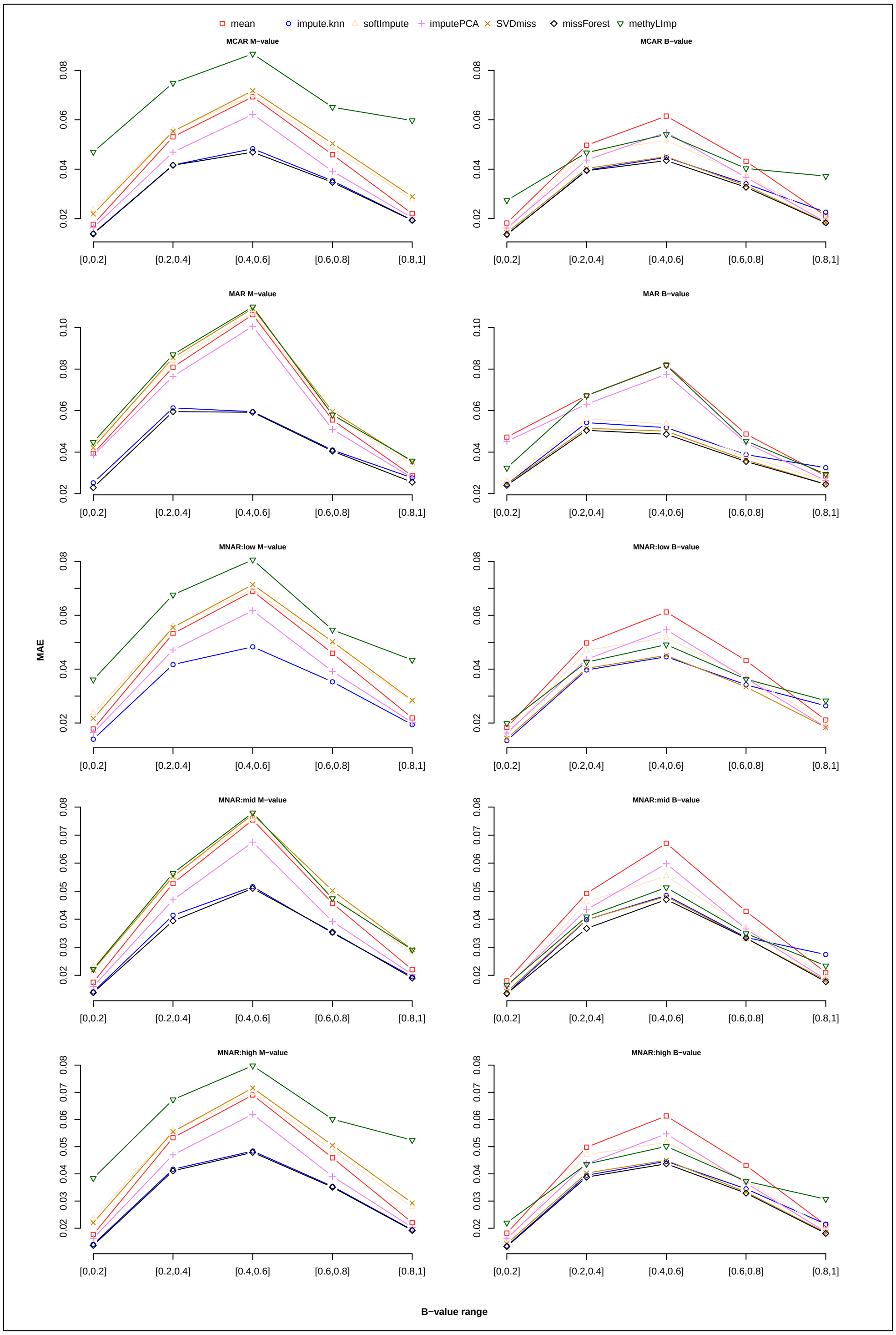

Figure 79: Dataset GSE64495 (D38). MAE imputation performances with respect to B-value range.

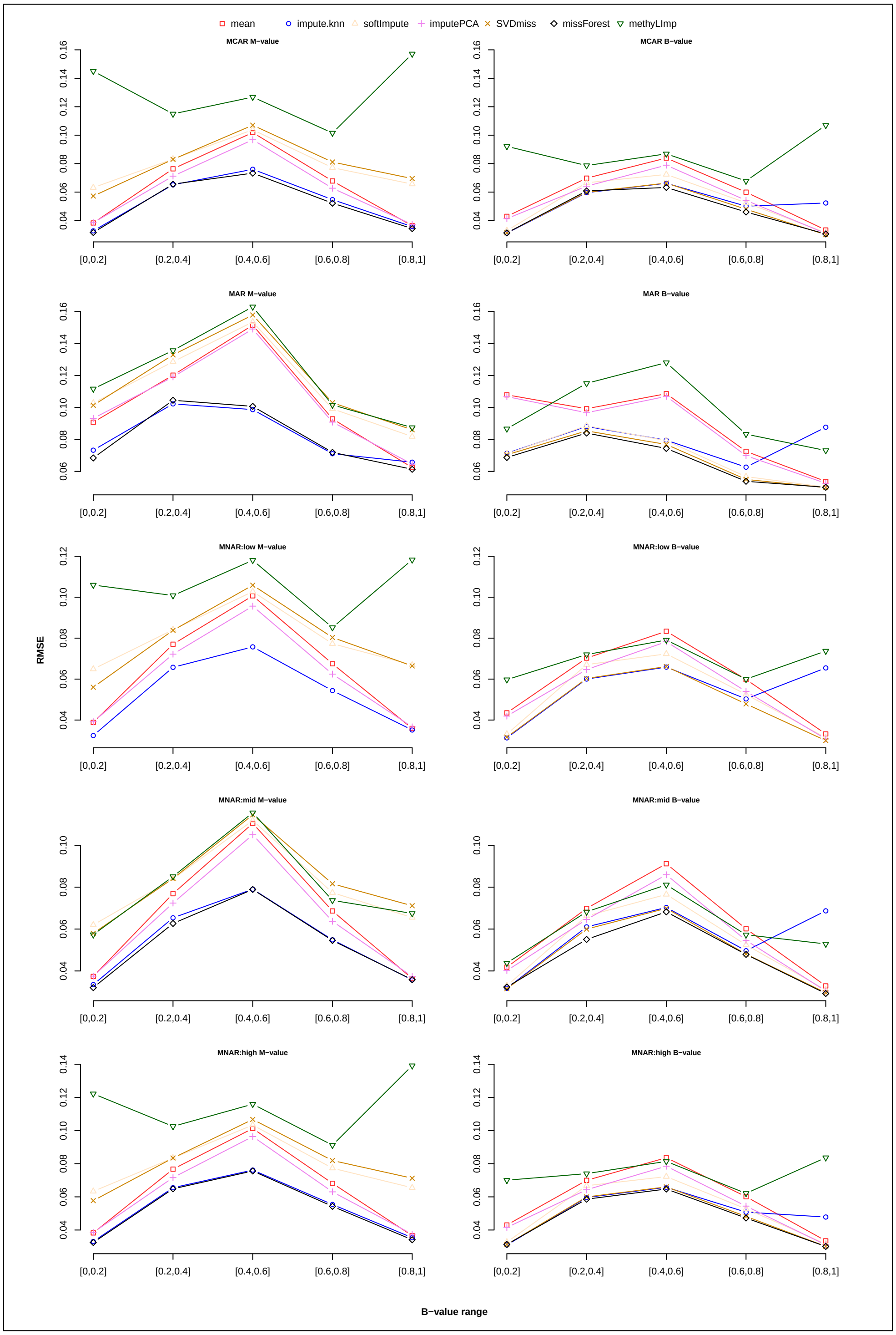

Figure 80: Dataset GSE64495 (D38). RMSE imputation performances with respect to B-value range.

2.39 GSE67477 (D39) - Liver - Cancer - 6 samples

Method	Avg time (sec)	Avg RAM (Mb)
mean	< 1	5
softImpute	< 1	41
imputePCA	9	141
impute.knn	< 1	41
SVDmiss	11	3599
methyLImp	3	133
missForest	539	250

Table 243: Dataset GSE67477 (D39). Average time and memory usage.

Table 244: Dataset GSE67477 (D39). Imputation performance on **MCAR** type missing values.

	MAE		RMSE	
Method	M-value	B-value	M-value	B-value
mean	0.120±0.002*	0.122±0.002	0.192±0.003	0.188±0.003*
softImpute	0.086±0.002*	0.093±0.005	0.149±0.004*	0.155±0.007
impute.knn	0.112±0.008*	0.154±0.017	0.190±0.010*	0.245±0.019
imputePCA	0.080±0.002*	0.083±0.002	0.141±0.003	0.141±0.003*
SVDmiss	0.121±0.003	0.058±0.002*	0.207±0.004	0.125±0.003*
missForest	0.093±0.002*	0.096±0.002	0.154±0.003	0.153±0.003*
methyLImp	0.055±0.001	**0.055±0.002***	**0.118±0.003***	0.120±0.003

Table 245: Dataset GSE67477 (D39). Imputation performance on **MAR** type missing values.

	MAE		RMSE	
Method	M-value	B-value	M-value	B-value
mean	0.125±0.002*	0.127±0.002	0.191±0.003	0.189±0.003*
softImpute	0.097±0.002*	0.106±0.006	0.162±0.004*	0.174±0.010
impute.knn	0.126±0.006*	0.164±0.012	0.202±0.008*	0.250±0.014
imputePCA	0.089±0.002*	0.091±0.002	0.152±0.004	0.151±0.004*
SVDmiss	0.134±0.003	0.070±0.002*	0.225±0.005	0.137±0.004*
missForest	0.100±0.002*	0.103±0.002	0.161±0.004	0.160±0.003*
methyLImp	0.066±0.002	**0.066±0.002***	**0.131±0.004***	0.134±0.004

Table 246: Dataset GSE67477 (D39). Imputation performance on **MNAR:low** type missing values.

	MAE		RMSE	
Method	M-value	B-value	M-value	B-value
mean	0.104±0.002*	0.109±0.002	0.184±0.004*	0.184±0.003
softImpute	0.076±0.002*	0.083±0.002	0.144±0.004*	0.151±0.005
impute.knn	0.122±0.013	0.090±0.010*	0.212±0.014	0.176±0.013*
imputePCA	0.068±0.002*	0.073±0.002	0.133±0.004*	0.136±0.004
SVDmiss	0.150±0.004	0.051±0.002*	0.257±0.005	0.123±0.004*
missForest	0.079±0.002*	0.085±0.002	0.146±0.004*	0.149±0.004
methyLImp	0.050±0.002	**0.048±0.002***	**0.114±0.004***	0.115±0.005

Table 247: Dataset GSE67477 (D39). Imputation performance on **MNAR:mid** type missing values.

	MAE		RMSE	
Method	M-value	B-value	M-value	B-value
mean	0.161±0.002	0.150±0.002*	0.210±0.002	0.196±0.002*
softImpute	0.132±0.002	0.128±0.005*	0.182±0.003*	0.184±0.007
impute.knn	0.103±0.004*	0.152±0.007	0.157±0.005*	0.217±0.010
imputePCA	0.122±0.002	0.113±0.002*	0.170±0.003	0.160±0.003*
SVDmiss	0.086±0.002	0.081±0.002*	0.159±0.004	0.142±0.003*
missForest	0.132±0.002	0.121±0.002*	0.178±0.002	0.166±0.002*
methyLImp	**0.079±0.003***	0.082±0.003	**0.137±0.003***	0.143±0.003

Table 248: Dataset GSE67477 (D39). Imputation performance on **MNAR:high** type missing values.

	MAE		RMSE	
Method	M-value	B-value	M-value	B-value
mean	0.111±0.002*	0.117±0.002	0.185±0.004*	0.185±0.003
softImpute	0.076±0.002*	0.089±0.006	0.142±0.004*	0.154±0.009
impute.knn	0.120±0.016*	0.230±0.038	0.203±0.018*	0.321±0.031
imputePCA	0.072±0.002*	0.077±0.002	0.134±0.004*	0.137±0.004
SVDmiss	0.123±0.003	0.057±0.002*	0.201±0.003	0.121±0.004*
missForest	0.085±0.002*	0.092±0.002	0.147±0.004*	0.151±0.003
methyLImp	0.053±0.002	**0.051±0.002***	**0.116±0.004***	0.116±0.004

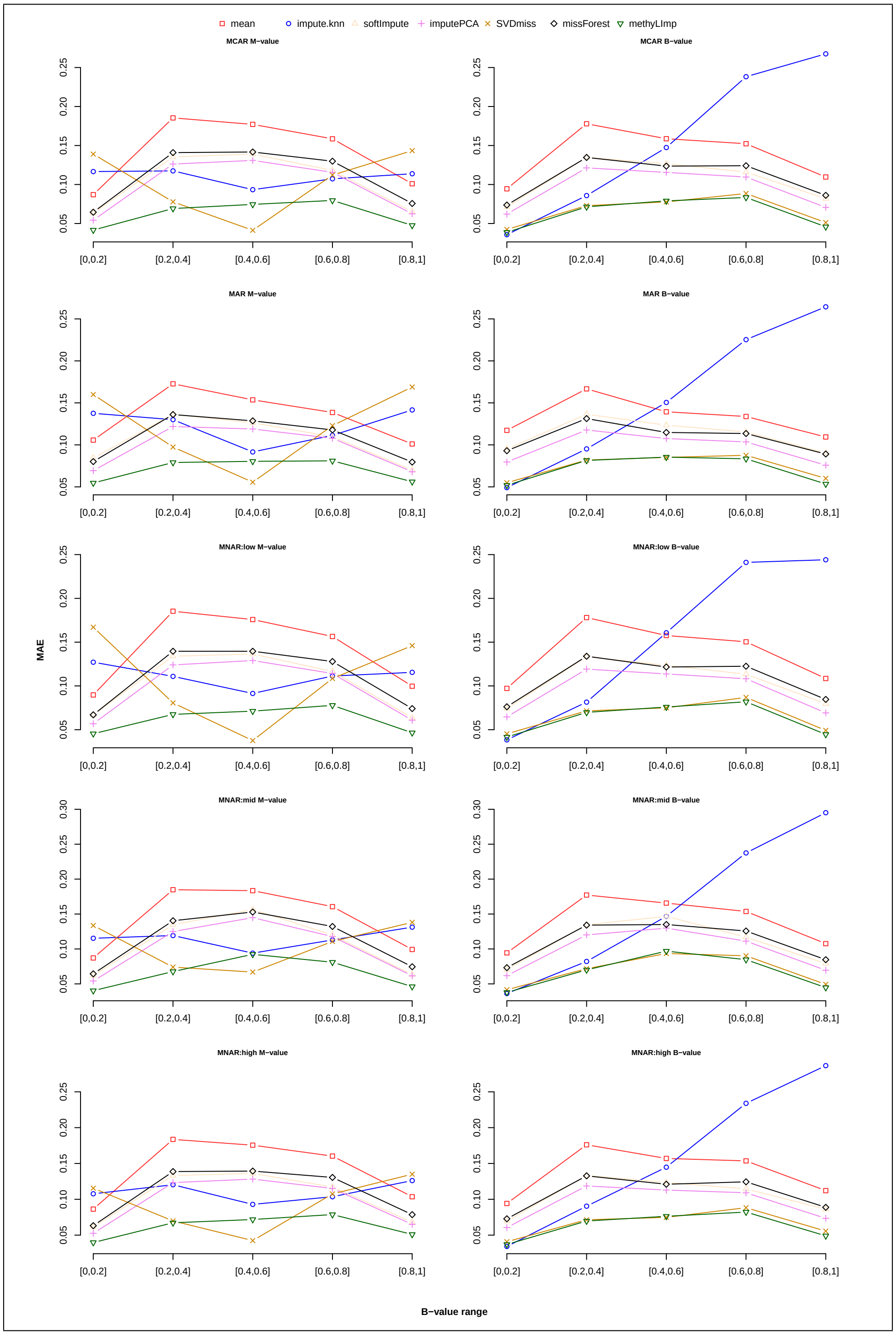

Figure 81: Dataset GSE67477 (D39). MAE imputation performances with respect to B-value range.

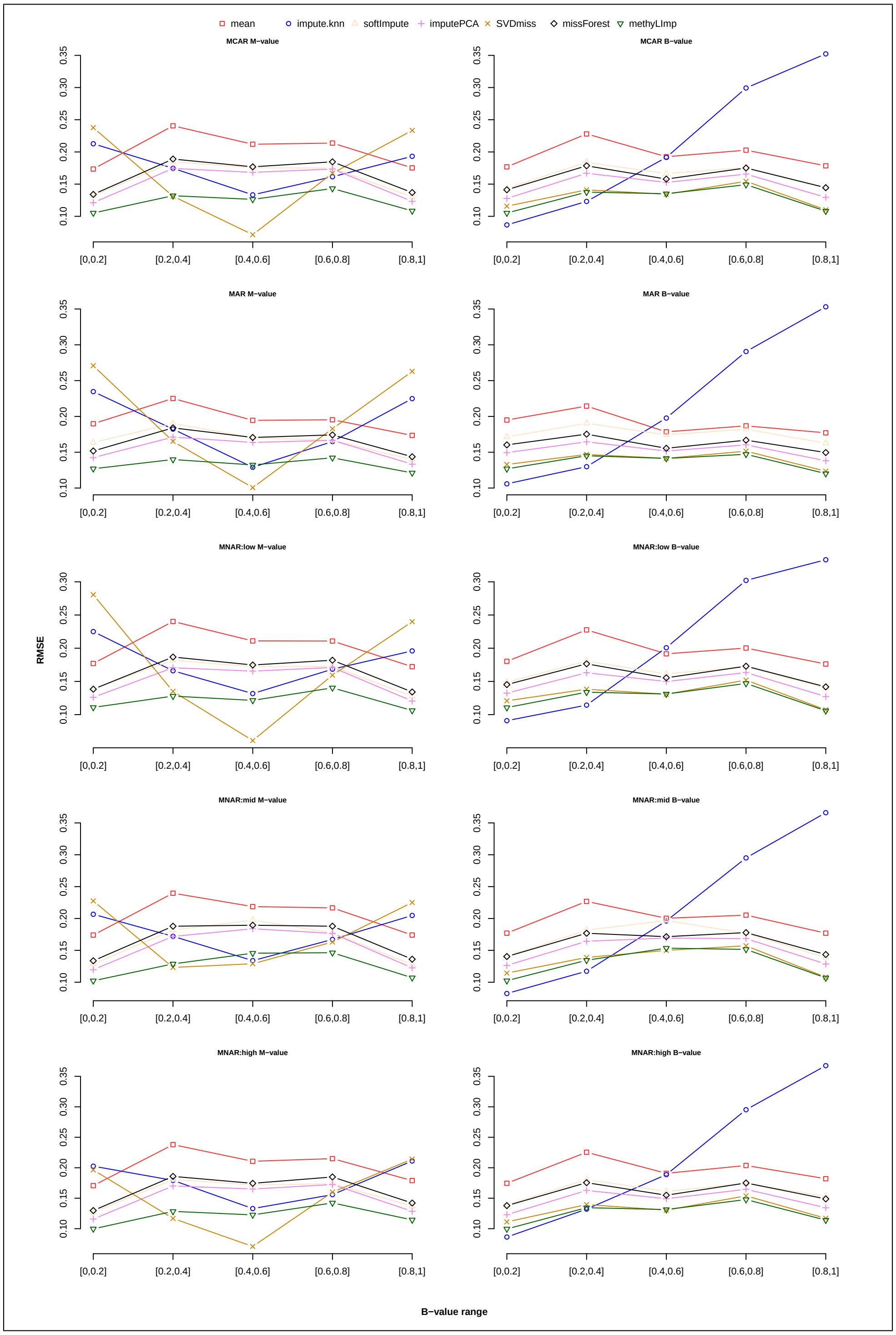

Figure 82: Dataset GSE67477 (D39). RMSE imputation performances with respect to B-value range.

2.40 GSE67484 (D40) - Liver, Intestine-Small - Normal - 4 samples

Method	Avg time (sec)	Avg RAM (Mb)
mean	< 1	4
softImpute	< 1	42
imputePCA	16	142
impute.knn	< 1	42
SVDmiss	15	3500
methyLImp	3	111
missForest	497	307

Table 249: Dataset GSE67484 (D40). Average time and memory usage.

Table 250: Dataset GSE67484 (D40). Imputation performance on **MCAR** type missing values.

	MAE		RMSE	
Method	M-value	B-value	M-value	B-value
mean	0.070±0.002	0.069±0.002*	0.116±0.003	0.111±0.003*
softImpute	0.079±0.002	0.051±0.003*	0.127±0.005	0.097±0.012*
impute.knn	0.090±0.011*	0.165±0.021	0.139±0.014*	0.239±0.023
imputePCA	0.049±0.002	0.046±0.001*	0.097±0.006	**0.075±0.003***
SVDmiss	0.095±0.002	0.069±0.002*	0.131±0.003	0.111±0.003*
missForest	0.061±0.002	0.060±0.001*	0.102±0.003	0.096±0.003*
methyLImp	0.088±0.002	**0.045±0.002***	0.119±0.003	0.095±0.008*

Table 251: Dataset GSE67484 (D40). Imputation performance on **MAR** type missing values.

	MAE		RMSE	
Method	M-value	B-value	M-value	B-value
mean	0.077±0.002	0.076±0.002*	0.123±0.003	0.119±0.003*
softImpute	0.091±0.003	0.084±0.012*	0.145±0.006*	0.165±0.035
impute.knn	0.108±0.006*	0.181±0.018	0.163±0.009*	0.253±0.018
imputePCA	0.058±0.001	**0.055±0.001***	0.104±0.004	**0.088±0.003***
SVDmiss	0.099±0.002	0.076±0.002*	0.137±0.003	0.119±0.003*
missForest	0.068±0.002	0.068±0.001*	0.111±0.003	0.106±0.003*
methyLImp	0.093±0.001	**0.055±0.002***	0.125±0.003	0.103±0.007*

Table 252: Dataset GSE67484 (D40). Imputation performance on **MNAR:low** type missing values.

	MAE		RMSE	
Method	M-value	B-value	M-value	B-value
mean	0.051±0.001*	0.051±0.001	0.097±0.003	0.096±0.003*
softImpute	0.069±0.002	0.037±0.002*	0.132±0.007	0.070±0.008*
impute.knn	0.100±0.020	0.086±0.011*	0.149±0.021*	0.161±0.018
imputePCA	0.041±0.002	**0.035±0.001***	0.104±0.008	**0.065±0.003***
SVDmiss	0.077±0.002	0.051±0.001*	0.113±0.003	0.095±0.003*
missForest	0.044±0.001*	0.045±0.001	0.086±0.003	0.084±0.003*
methyLImp	0.075±0.002	0.041±0.002*	0.106±0.003*	0.109±0.011

Table 253: Dataset GSE67484 (D40). Imputation performance on **MNAR:mid** type missing values.

	MAE		RMSE	
Method	M-value	B-value	M-value	B-value
mean	0.104±0.002	0.099±0.002*	0.145±0.002	0.136±0.002*
softImpute	0.102±0.003	0.082±0.009*	0.145±0.005	0.137±0.022*
impute.knn	0.075±0.003*	0.133±0.011	0.106±0.004*	0.192±0.014
imputePCA	0.073±0.002	0.068±0.002*	0.114±0.004	**0.100±0.002***
SVDmiss	0.114±0.002	0.099±0.002*	0.153±0.002	0.135±0.002*
missForest	0.093±0.002	0.087±0.002*	0.132±0.003	0.121±0.002*
methyLImp	0.093±0.002	**0.064±0.002***	0.123±0.002	0.106±0.005*

Table 254: Dataset GSE67484 (D40). Imputation performance on **MNAR:high** type missing values.

	MAE		RMSE	
Method	M-value	B-value	M-value	B-value
mean	0.063±0.002*	0.064±0.002	0.109±0.004	0.106±0.003*
softImpute	0.074±0.002	0.064±0.010*	0.114±0.005*	0.142±0.037
impute.knn	0.155±0.031*	0.286±0.035	0.234±0.038*	0.343±0.027
imputePCA	0.043±0.001	0.043±0.001	0.079±0.004	**0.068±0.003***
SVDmiss	0.096±0.002	0.065±0.002*	0.130±0.004	0.106±0.003*
missForest	0.055±0.001*	0.056±0.001	0.095±0.005	0.090±0.003*
methyLImp	0.099±0.002	**0.038±0.001***	0.128±0.003	0.075±0.004*

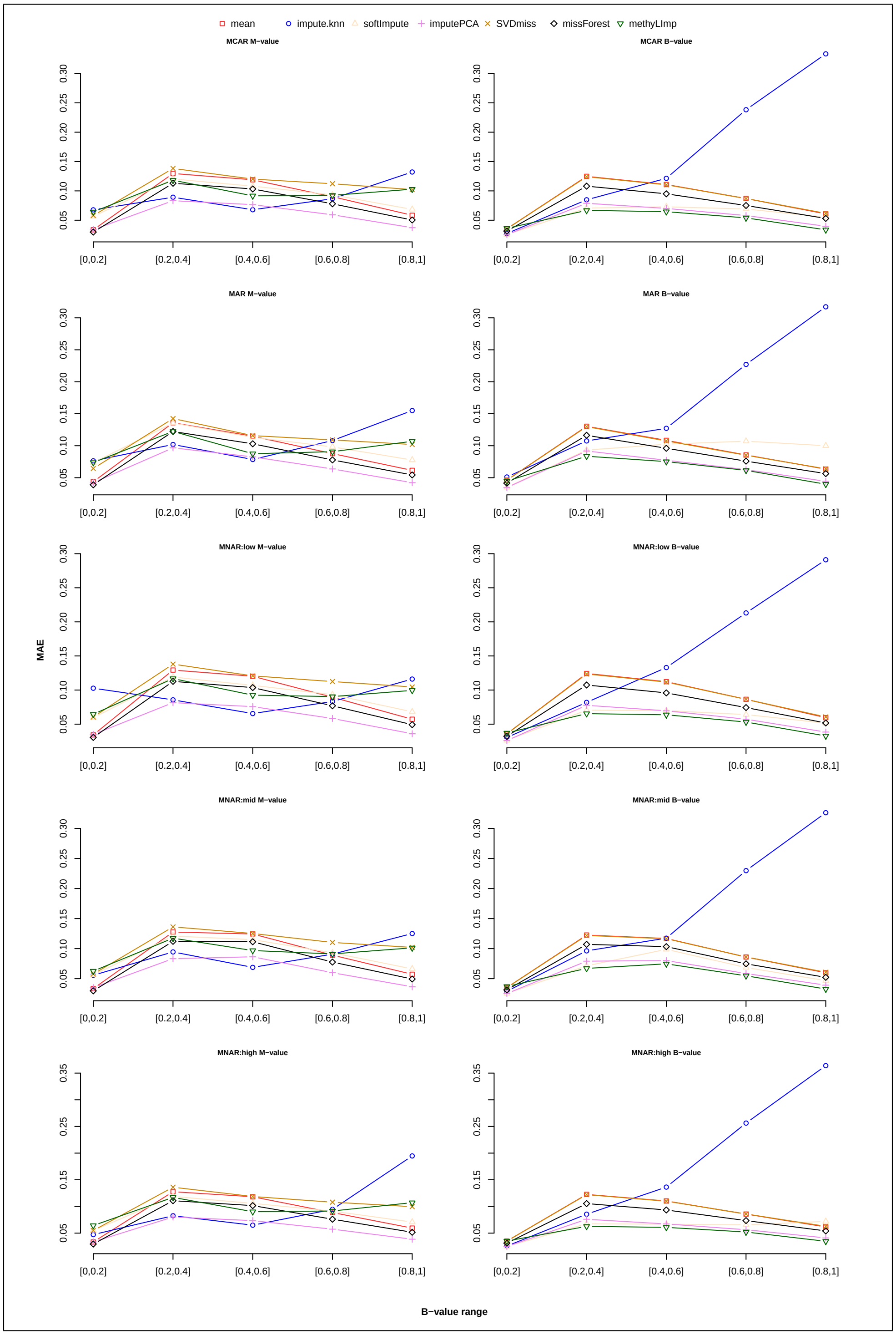

Figure 83: Dataset GSE67484 (D40). MAE imputation performances with respect to B-value range.

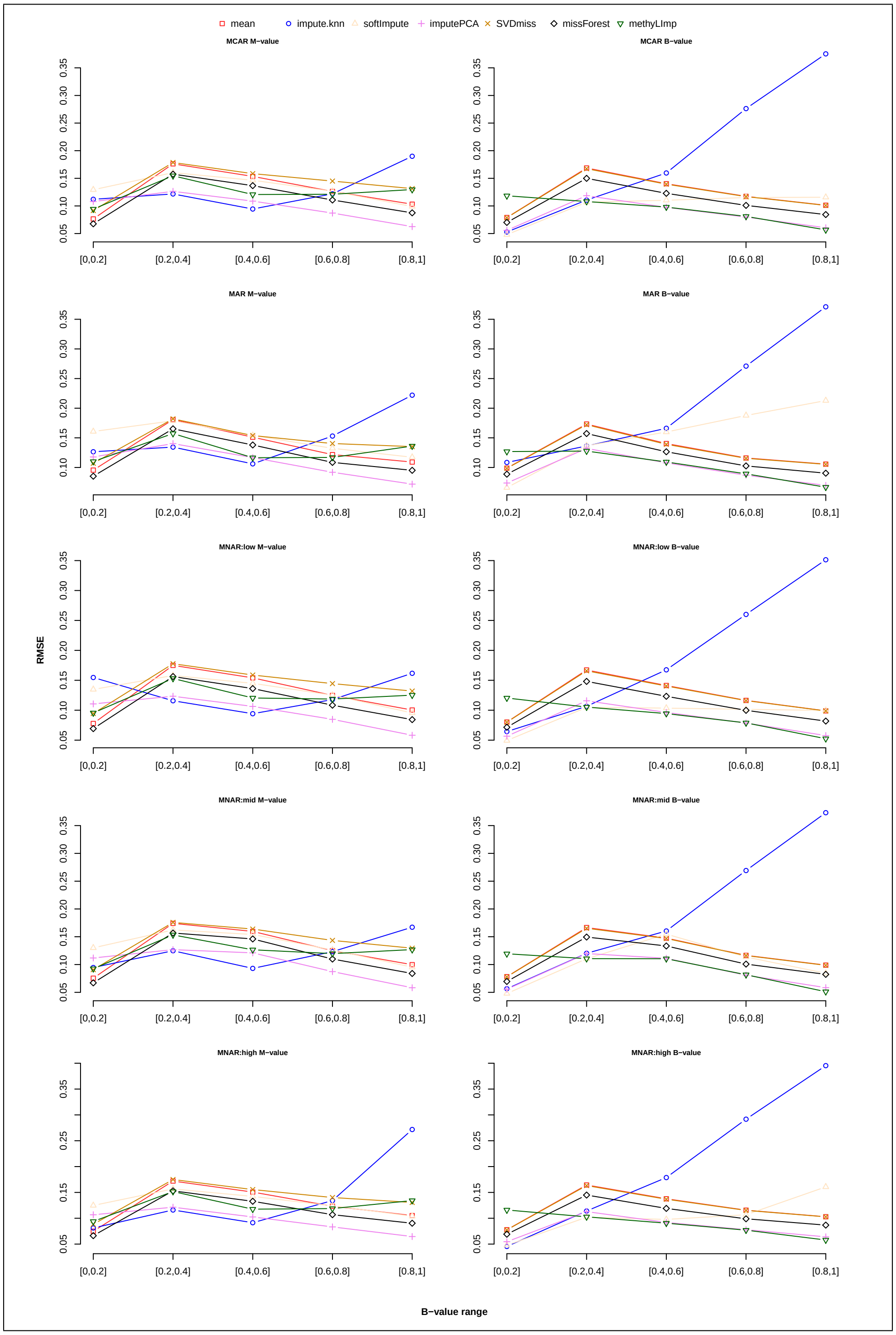

Figure 84: Dataset GSE67484 (D40). RMSE imputation performances with respect to B-value range.

2.41 GSE69502 (D41) - Brain, Spinal Cord - Normal - 20 samples

Method	Avg time (sec)	Avg RAM (Mb)
mean	< 1	18
softImpute	< 1	54
imputePCA	8	168
impute.knn	1	63
SVDmiss	50	3688
methyLImp	57	89
missForest	18422	134

Table 255: Dataset GSE69502 (D41). Average time and memory usage.

Table 256: Dataset GSE69502 (D41). Imputation performance on **MCAR** type missing values.

	MAE		RMSE	
Method	M-value	B-value	M-value	B-value
mean	0.031±0.001*	0.031±0.001	0.054±0.001	0.054±0.001*
softImpute	0.023±0.001*	0.023±0.001	0.038±0.001*	0.038±0.001
impute.knn	0.032±0.006*	0.044±0.009	0.071±0.017*	0.100±0.019
imputePCA	0.024±0.001*	0.024±0.001	0.039±0.001*	0.039±0.001
SVDmiss	0.022±0.001*	0.023±0.001	0.038±0.001*	0.039±0.001
missForest	0.023±0.001*	0.023±0.001	0.038±0.001	0.038±0.001
methyLImp	**0.020±0.001***	0.020±0.001	0.032±0.001	**0.032±0.001***

Table 257: Dataset GSE69502 (D41). Imputation performance on **MAR** type missing values.

	MAE		RMSE	
Method	M-value	B-value	M-value	B-value
mean	0.031±0.001*	0.031±0.001	0.053±0.001	0.053±0.001*
softImpute	0.025±0.001*	0.025±0.001	0.040±0.001*	0.040±0.001
impute.knn	0.028±0.003*	0.035±0.005	0.052±0.011*	0.072±0.015
imputePCA	0.025±0.001*	0.025±0.001	0.041±0.001	0.041±0.001*
SVDmiss	0.025±0.001*	0.026±0.001	0.041±0.001*	0.042±0.001
missForest	0.025±0.001*	0.025±0.001	0.040±0.001	0.040±0.001
methyLImp	**0.022±0.001***	0.022±0.001	**0.036±0.001**	**0.036±0.001**

Table 258: Dataset GSE69502 (D41). Imputation performance on **MNAR:low** type missing values.

	MAE		RMSE	
Method	M-value	B-value	M-value	B-value
mean	0.023±0.001*	0.024±0.001	0.043±0.001*	0.044±0.001
softImpute	0.017±0.001*	0.018±0.001	0.030±0.001*	0.030±0.001
impute.knn	0.028±0.008	0.026±0.005*	0.072±0.022	0.064±0.016*
imputePCA	0.018±0.001*	0.018±0.001	0.031±0.001*	0.031±0.001
SVDmiss	0.017±0.001*	0.018±0.001	0.030±0.001*	0.031±0.001
missForest	0.017±0.001*	0.018±0.001	0.030±0.001*	0.031±0.001
methyLImp	**0.016±0.001***	0.016±0.001	**0.026±0.001***	0.026±0.001

Table 259: Dataset GSE69502 (D41). Imputation performance on **MNAR:mid** type missing values.

	MAE		RMSE	
Method	M-value	B-value	M-value	B-value
mean	0.061±0.001	0.059±0.001*	0.092±0.001	0.087±0.001*
softImpute	0.043±0.001	0.042±0.001*	0.062±0.001	0.062±0.001*
impute.knn	0.042±0.001*	0.044±0.003	0.064±0.004*	0.072±0.009
imputePCA	0.045±0.001	0.044±0.001*	0.066±0.001	0.064±0.001*
SVDmiss	0.042±0.001	0.041±0.001*	0.062±0.001	0.062±0.001*
missForest	0.044±0.001	0.043±0.001*	0.065±0.001	0.063±0.001*
methyLImp	0.037±0.001	**0.036±0.001***	0.055±0.001	**0.053±0.001***

Table 260: Dataset GSE69502 (D41). Imputation performance on **MNAR:high** type missing values.

	MAE		RMSE	
Method	M-value	B-value	M-value	B-value
mean	0.027±0.001*	0.028±0.001	0.046±0.001*	0.047±0.001
softImpute	0.021±0.001*	0.021±0.001	0.033±0.001*	0.033±0.001
impute.knn	0.031±0.007*	0.061±0.014	0.074±0.021*	0.132±0.025
imputePCA	0.021±0.001*	0.022±0.001	0.034±0.001*	0.034±0.001
SVDmiss	0.020±0.001*	0.022±0.001	0.033±0.001*	0.035±0.001
missForest	0.020±0.001*	0.021±0.001	0.033±0.001*	0.033±0.001
methyLImp	**0.018±0.001***	0.019±0.001	**0.028±0.001***	0.028±0.001

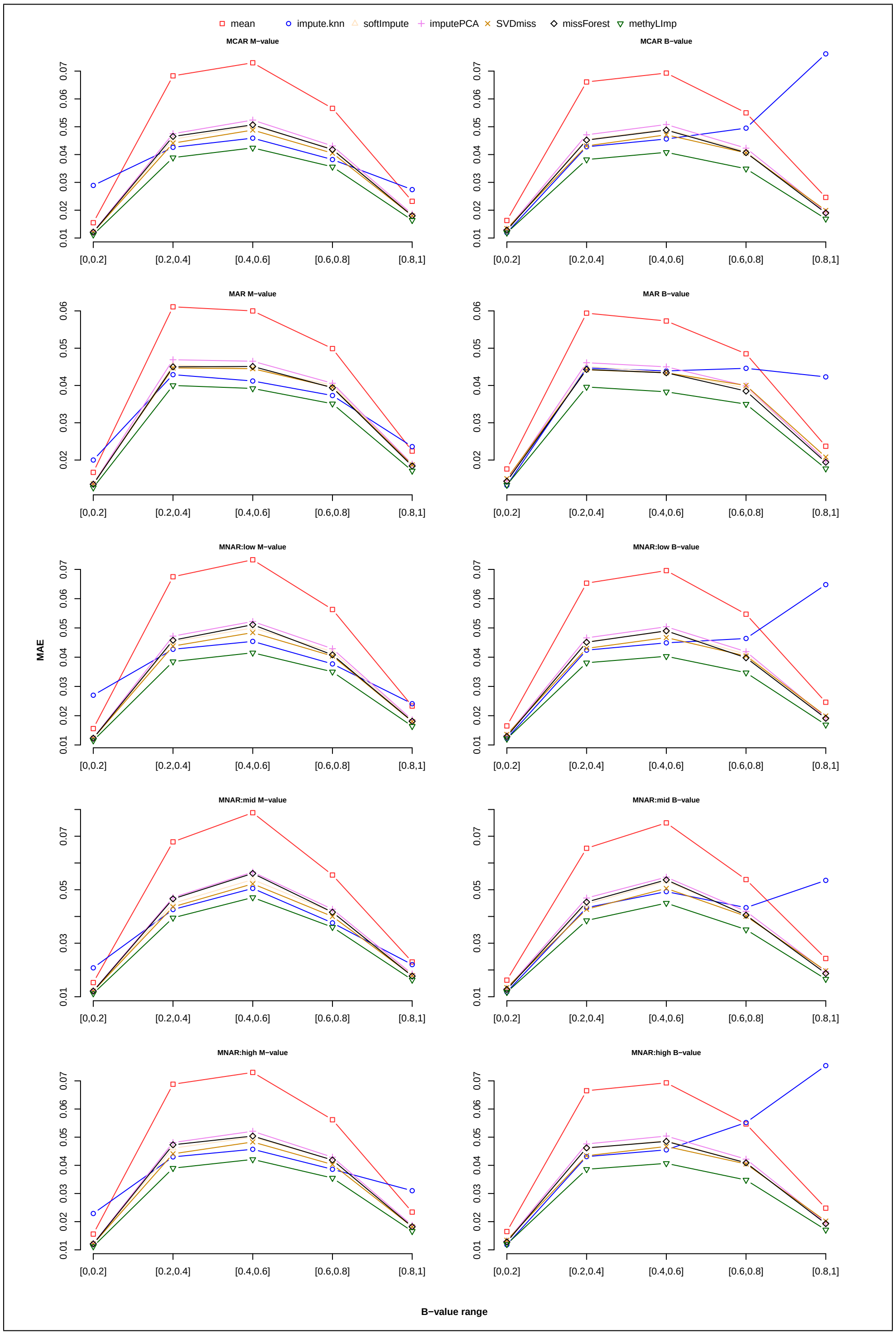

Figure 85: Dataset GSE69502 (D41). MAE imputation performances with respect to B-value range.

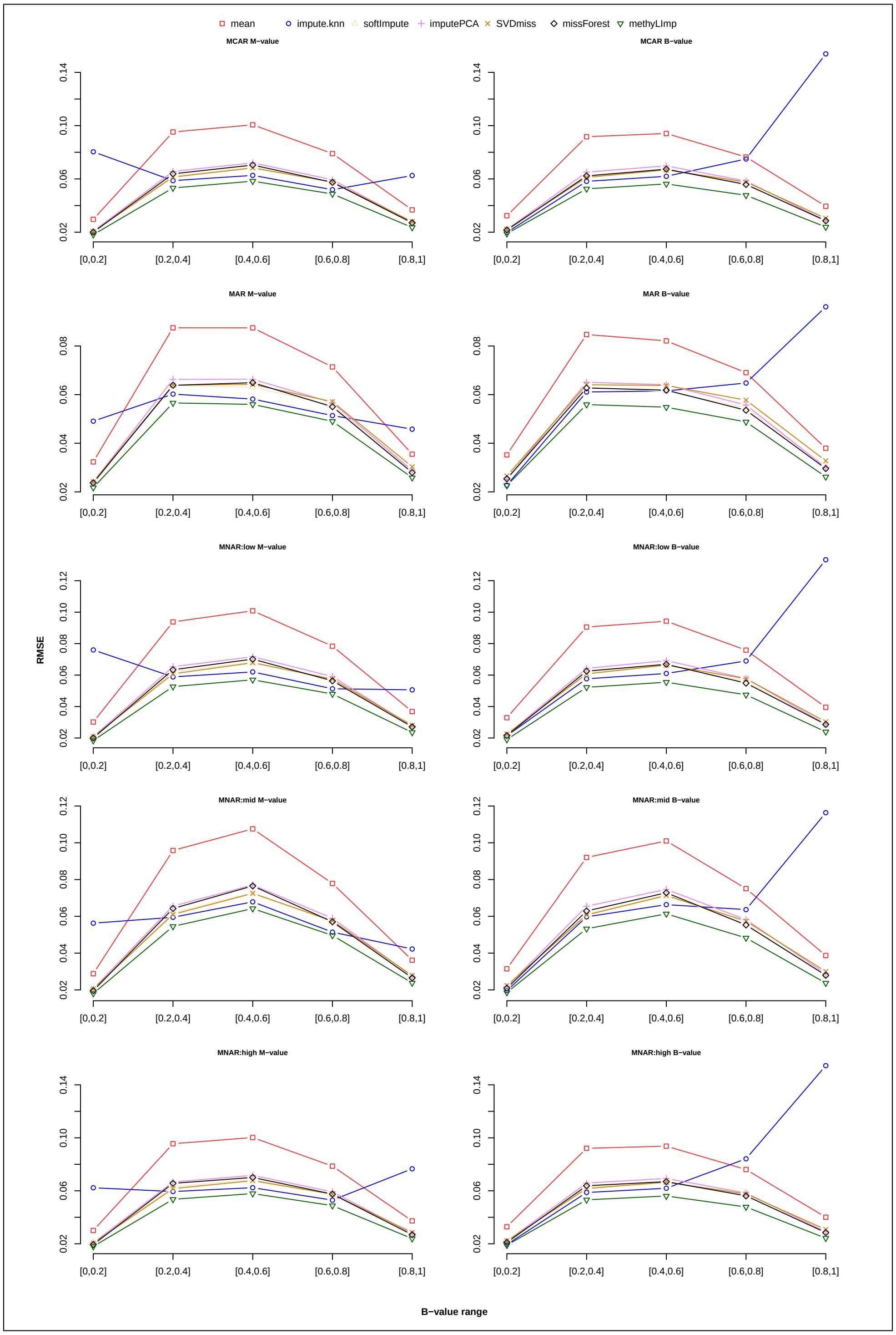

Figure 86: Dataset GSE69502 (D41). RMSE imputation performances with respect to B-value range.

2.42 GSE71955 (D42) - Blood - Normal - 62 samples

Method	Avg time (sec)	Avg RAM (Mb)
mean	< 1	56
softImpute	1	109
imputePCA	49	259
impute.knn	2	124
SVDmiss	163	3361
methyLImp	1084	145
missForest	138547	319

Table 261: Dataset GSE71955 (D42). Average time and memory usage.

Table 262: Dataset GSE71955 (D42). Imputation performance on **MCAR** type missing values.

	MAE		RMSE	
Method	M-value	B-value	M-value	B-value
mean	0.026±0.001*	0.026±0.001	0.041±0.001	0.041±0.001*
softImpute	0.023±0.001*	0.024±0.001	0.036±0.001*	0.037±0.001
impute.knn	0.022±0.001*	0.025±0.002	0.037±0.003*	0.048±0.007
imputePCA	0.022±0.001*	0.022±0.001	0.035±0.001	0.035±0.001*
SVDmiss	0.021±0.001*	0.022±0.001	0.033±0.001	0.032±0.001*
missForest	0.021±0.001*	0.021±0.001	0.031±0.001	0.031±0.001
methyLImp	**0.019±0.001***	0.019±0.001	0.028±0.001	**0.028±0.001***

Table 263: Dataset GSE71955 (D42). Imputation performance on **MAR** type missing values.

	MAE		RMSE	
Method	M-value	B-value	M-value	B-value
mean	0.033±0.001	0.032±0.001*	0.053±0.001	0.053±0.001*
softImpute	0.031±0.001*	0.031±0.001	0.051±0.001*	0.051±0.001
impute.knn	0.028±0.001*	0.030±0.002	0.048±0.002*	0.060±0.010
imputePCA	0.030±0.001	0.029±0.001*	0.050±0.001	0.049±0.001*
SVDmiss	0.028±0.001	0.027±0.001*	0.044±0.001	0.041±0.001*
missForest	0.025±0.001*	0.025±0.001	**0.038±0.001***	0.039±0.001
methyLImp	0.025±0.001	**0.024±0.001***	0.041±0.001	0.038±0.001*

Table 264: Dataset GSE71955 (D42). Imputation performance on **MNAR:low** type missing values.

	MAE		RMSE	
Method	M-value	B-value	M-value	B-value
mean	0.021±0.001*	0.022±0.001	0.035±0.001*	0.036±0.001
softImpute	0.019±0.001*	0.020±0.001	0.031±0.001*	0.032±0.001
impute.knn	0.018±0.001*	0.020±0.001	0.032±0.002*	0.042±0.005
imputePCA	0.018±0.001*	0.019±0.001	0.030±0.001*	0.031±0.001
SVDmiss	0.018±0.001*	0.018±0.001	0.028±0.001*	0.028±0.001
missForest	0.017±0.001*	0.017±0.001	0.026±0.001*	0.027±0.001
methyLImp	**0.016±0.001***	0.016±0.001	**0.024±0.001***	0.024±0.001

Table 265: Dataset GSE71955 (D42). Imputation performance on **MNAR:mid** type missing values.

	MAE		RMSE	
Method	M-value	B-value	M-value	B-value
mean	0.046±0.001	0.044±0.001*	0.065±0.001	0.063±0.001*
softImpute	0.039±0.001	0.039±0.001*	0.056±0.001	0.055±0.001*
impute.knn	0.034±0.001*	0.036±0.001	0.050±0.001*	0.056±0.004
imputePCA	0.037±0.001	0.036±0.001*	0.053±0.001	0.052±0.001*
SVDmiss	0.035±0.001	0.034±0.001*	0.051±0.001	0.048±0.001*
missForest	0.033±0.001	0.032±0.001*	0.048±0.001	0.047±0.001*
methyLImp	0.030±0.001	**0.029±0.001***	0.044±0.001	**0.042±0.001***

Table 266: Dataset GSE71955 (D42). Imputation performance on **MNAR:high** type missing values.

	MAE		RMSE	
Method	M-value	B-value	M-value	B-value
mean	0.022±0.001*	0.022±0.001	0.034±0.001*	0.034±0.001
softImpute	0.020±0.001*	0.020±0.001	0.030±0.001*	0.031±0.001
impute.knn	0.020±0.001*	0.024±0.003	0.034±0.003*	0.052±0.011
imputePCA	0.019±0.001*	0.019±0.001	0.029±0.001*	0.029±0.001
SVDmiss	0.018±0.001*	0.019±0.001	0.028±0.001*	0.028±0.001
missForest	0.018±0.001*	0.018±0.001	0.026±0.001*	0.027±0.001
methyLImp	**0.017±0.001***	0.017±0.001	**0.024±0.001***	0.025±0.001

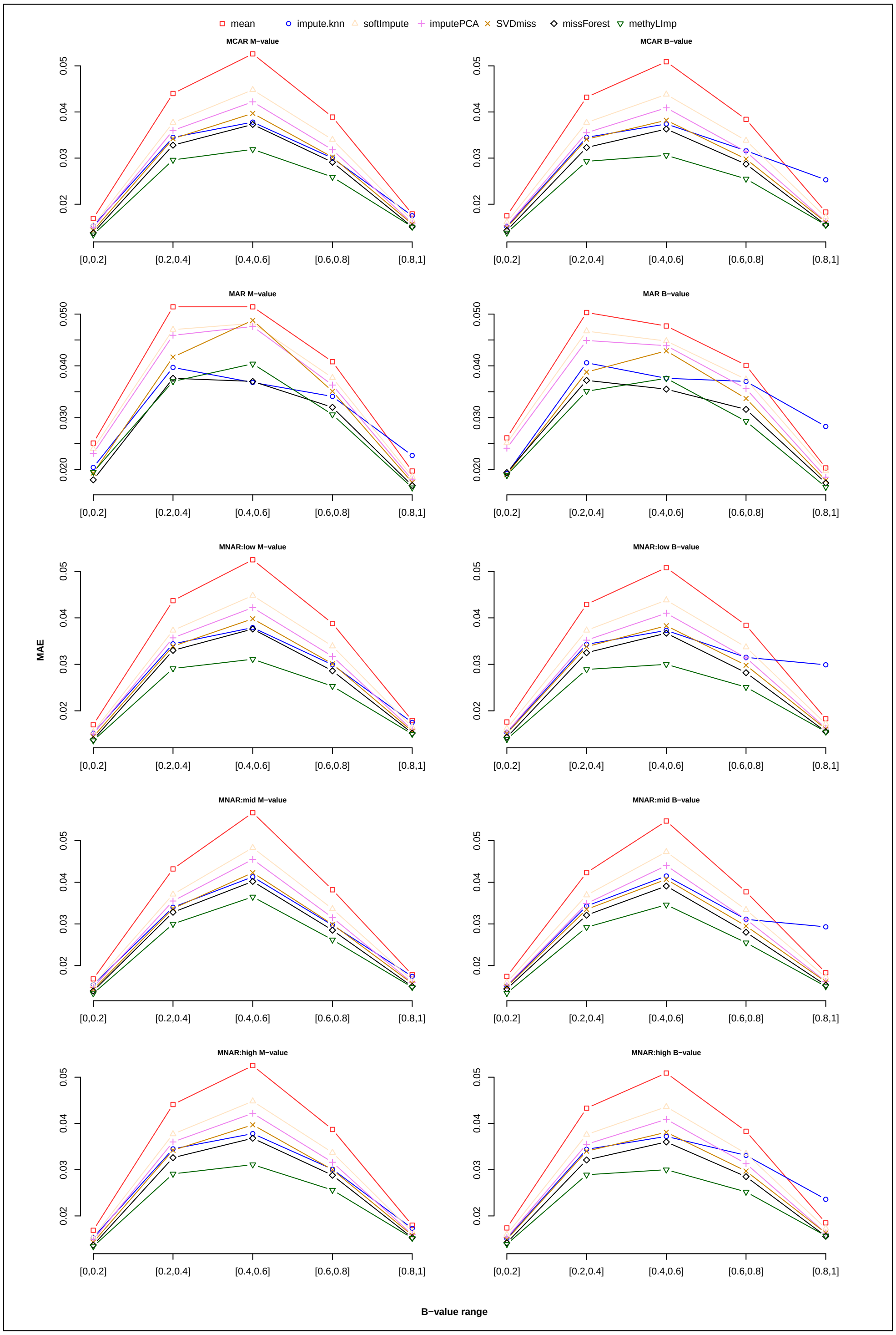

Figure 87: Dataset GSE71955 (D42). MAE imputation performances with respect to B-value range.

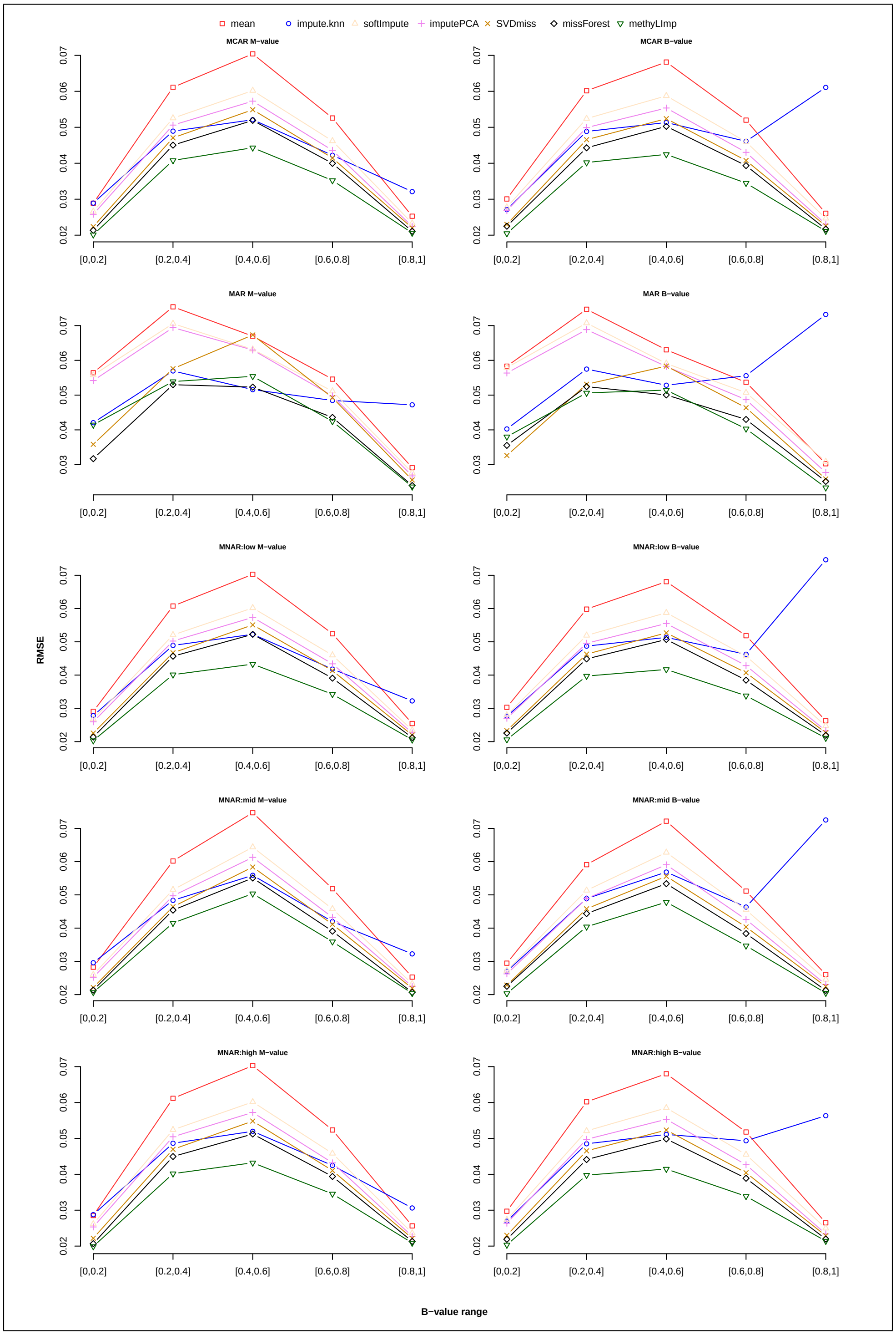

Figure 88: Dataset GSE71955 (D42). RMSE imputation performances with respect to B-value range.

2.43 GSE73103 (D43) - Blood - Normal - 268 samples

Method	Avg time (sec)	Avg RAM (Mb)
mean	< 1	227
softImpute	3	381
imputePCA	132	681
impute.knn	19	404
SVDmiss	1131	3781
methyLImp	15664	353
missForest	786797	1178

Table 267: Dataset GSE73103 (D43). Average time and memory usage.

Table 268: Dataset GSE73103 (D43). Imputation performance on **MCAR** type missing values.

	MAE		RMSE	
Method	M-value	B-value	M-value	B-value
mean	0.026±0.001*	0.027±0.001	0.050±0.001	0.050±0.001*
softImpute	0.022±0.001*	0.023±0.001	0.042±0.001	0.042±0.001*
impute.knn	0.021±0.001*	0.022±0.001	0.040±0.001*	0.045±0.005
imputePCA	0.021±0.001*	0.022±0.001	0.041±0.001	0.041±0.001*
SVDmiss	0.020±0.001*	0.021±0.001	0.040±0.001	0.039±0.001*
methyLImp	0.024±0.001	0.024±0.001	0.045±0.001*	0.046±0.001
missForest	**0.020±0.001***	0.020±0.001	0.039±0.001	**0.039±0.001***

Table 269: Dataset GSE73103 (D43). Imputation performance on **MAR** type missing values.

	MAE		RMSE	
Method	M-value	B-value	M-value	B-value
mean	0.031±0.001*	0.032±0.001	0.059±0.001	0.058±0.001*
softImpute	0.025±0.001*	0.026±0.001	0.049±0.001	0.049±0.001*
impute.knn	0.024±0.001*	0.028±0.002	0.048±0.002*	0.070±0.013
imputePCA	0.024±0.001*	0.025±0.001	0.048±0.001	0.048±0.001*
SVDmiss	0.023±0.001*	0.024±0.001	0.047±0.001	0.046±0.001*
methyLImp	**0.022±0.001***	0.023±0.001	**0.046±0.001***	0.047±0.001
missForest	0.023±0.001*	0.023±0.001	0.046±0.001	**0.046±0.001***

Table 270: Dataset GSE73103 (D43). Imputation performance on **MNAR:low** type missing values.

	MAE		RMSE	
Method	M-value	B-value	M-value	B-value
mean	0.019±0.001*	0.020±0.001	0.040±0.001*	0.040±0.001
softImpute	0.016±0.001*	0.017±0.001	0.034±0.001*	0.034±0.001
impute.knn	0.016±0.001*	0.017±0.001	0.033±0.001*	0.038±0.004
imputePCA	0.016±0.001*	0.016±0.001	0.033±0.001*	0.033±0.001
SVDmiss	0.015±0.001*	0.016±0.001	0.032±0.001*	0.033±0.001
methyLImp	0.019±0.001*	0.020±0.001	0.039±0.001*	0.046±0.003
missForest	**0.015±0.001***	0.016±0.001	**0.032±0.001***	0.032±0.001

Table 271: Dataset GSE73103 (D43). Imputation performance on **MNAR:mid** type missing values.

	MAE		RMSE	
Method	M-value	B-value	M-value	B-value
mean	0.066±0.001	0.064±0.001*	0.105±0.001	0.100±0.001*
softImpute	0.053±0.001	0.052±0.001*	0.083±0.001	0.080±0.001*
impute.knn	0.050±0.001	0.050±0.001	0.078±0.001*	0.080±0.003
imputePCA	0.052±0.001	0.050±0.001*	0.081±0.001	0.078±0.001*
SVDmiss	0.048±0.001	0.047±0.001*	0.077±0.001	0.074±0.001*
methyLImp	0.078±0.004*	0.094±0.005	0.118±0.005*	0.144±0.006
missForest	0.048±0.001	**0.046±0.001***	0.078±0.001	**0.073±0.001***

Table 272: Dataset GSE73103 (D43). Imputation performance on **MNAR:high** type missing values.

	MAE		RMSE	
Method	M-value	B-value	M-value	B-value
mean	0.024±0.001*	0.025±0.001	0.044±0.001*	0.045±0.001
softImpute	0.019±0.001*	0.021±0.001	0.036±0.001*	0.037±0.001
impute.knn	0.019±0.001*	0.020±0.001	0.035±0.001*	0.041±0.006
imputePCA	0.019±0.001*	0.020±0.001	0.036±0.001*	0.036±0.001
SVDmiss	0.018±0.001*	0.019±0.001	0.035±0.001*	0.035±0.001
methyLImp	0.021±0.001*	0.022±0.001	0.040±0.001*	0.043±0.003
missForest	**0.018±0.001***	0.018±0.001	**0.034±0.001***	0.034±0.001

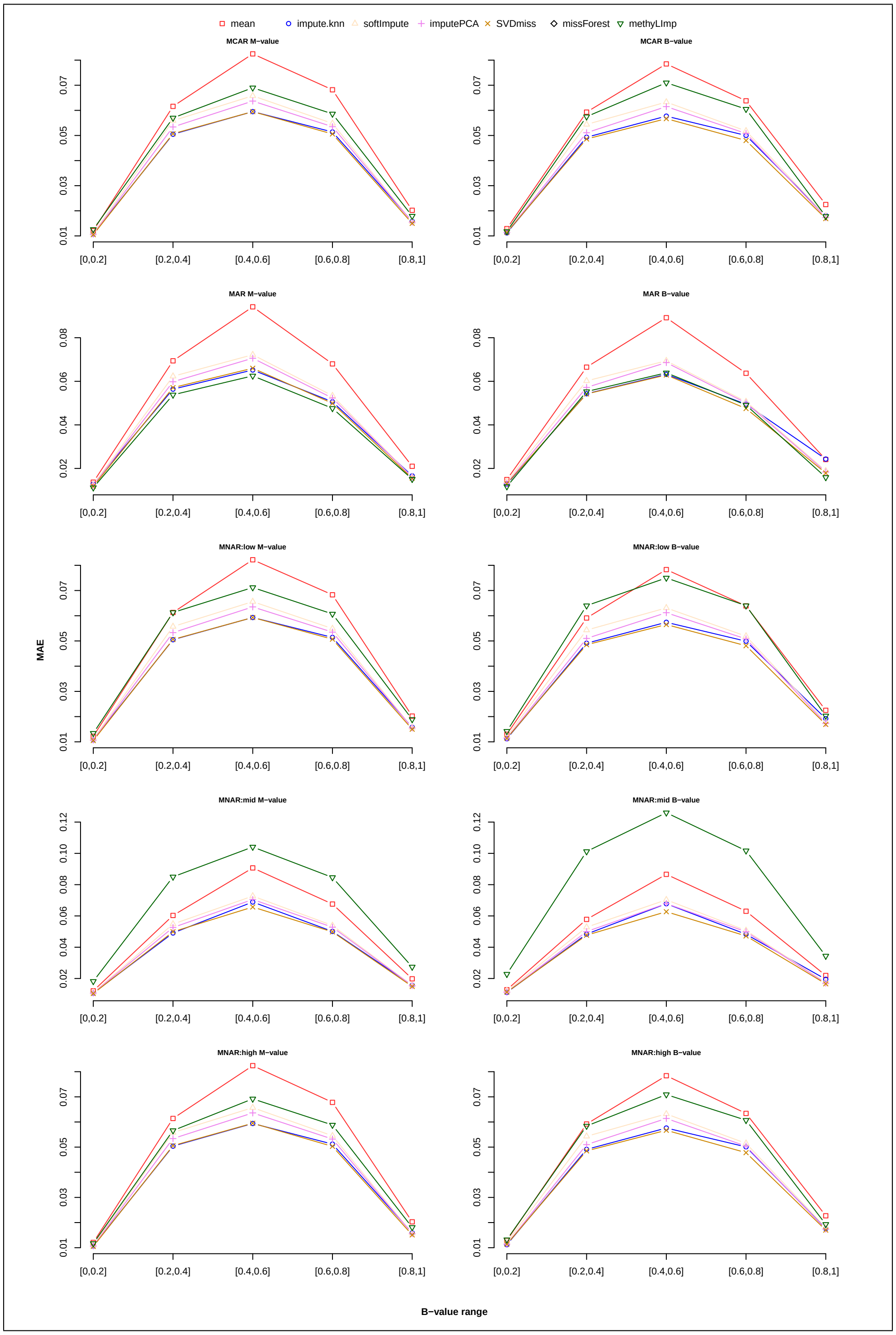

Figure 89: Dataset GSE73103 (D43). MAE imputation performances with respect to B-value range.

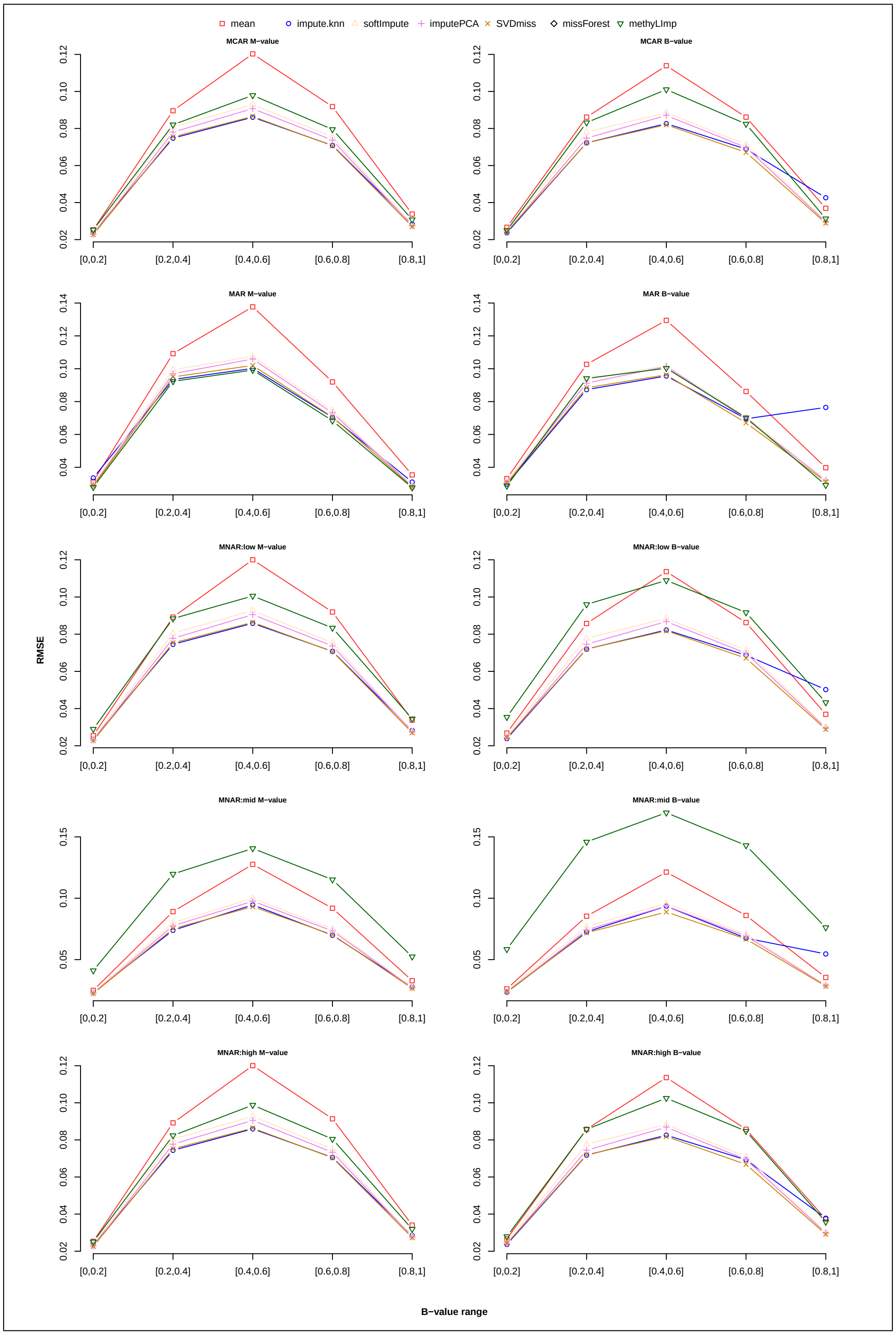

Figure 90: Dataset GSE73103 (D43). RMSE imputation performances with respect to B-value range.

2.44 GSE73747 (D44) - Brain - Normal - 9 samples

Method	Avg time (sec)	Avg RAM (Mb)
mean	< 1	8
softImpute	< 1	40
imputePCA	7	154
impute.knn	< 1	44
SVDmiss	14	3624
methyLImp	5	118
missForest	1758	281

Table 273: Dataset GSE73747 (D44). Average time and memory usage.

Table 274: Dataset GSE73747 (D44). Imputation performance on **MCAR** type missing values.

	MAE		RMSE	
Method	M-value	B-value	M-value	B-value
mean	0.029±0.001	0.029±0.001*	0.053±0.002	0.053±0.002*
softImpute	0.024±0.001	0.026±0.009*	0.045±0.002	0.048±0.028*
impute.knn	0.048±0.015*	0.078±0.023	0.111±0.028*	0.151±0.030
imputePCA	0.023±0.001*	0.023±0.001	0.043±0.002	0.043±0.002*
SVDmiss	0.104±0.003	0.027±0.001*	0.266±0.005	0.054±0.004*
missForest	0.026±0.001*	0.026±0.001	0.047±0.002	0.047±0.002*
methyLImp	**0.021±0.001***	0.022±0.001	**0.041±0.002***	0.041±0.002

Table 275: Dataset GSE73747 (D44). Imputation performance on **MAR** type missing values.

	MAE		RMSE	
Method	M-value	B-value	M-value	B-value
mean	0.036±0.001*	0.036±0.001	0.067±0.002	0.066±0.002*
softImpute	0.034±0.001*	0.036±0.008	0.067±0.003*	0.073±0.022
impute.knn	0.043±0.007*	0.067±0.017	0.093±0.016*	0.132±0.026
imputePCA	0.030±0.001*	0.031±0.001	0.063±0.003	**0.061±0.003***
SVDmiss	0.100±0.004	0.038±0.001*	0.238±0.008	0.085±0.005*
missForest	0.033±0.001*	0.033±0.001	0.064±0.002	0.063±0.002*
methyLImp	**0.029±0.001***	0.030±0.001	0.064±0.003*	0.064±0.003

Table 276: Dataset GSE73747 (D44). Imputation performance on **MNAR:low** type missing values.

	MAE		RMSE	
Method	M-value	B-value	M-value	B-value
mean	0.021±0.001*	0.021±0.001	0.044±0.002*	0.044±0.002
softImpute	0.018±0.001*	0.018±0.001	0.038±0.003	0.037±0.003*
impute.knn	0.064±0.026	0.037±0.011*	0.148±0.043	0.095±0.027*
imputePCA	0.017±0.001*	0.018±0.001	0.036±0.002*	0.037±0.002
SVDmiss	0.105±0.003	0.021±0.001*	0.278±0.005	0.046±0.004*
missForest	0.018±0.001*	0.019±0.001	0.039±0.002*	0.039±0.002
methyLImp	**0.016±0.001***	0.017±0.001	**0.035±0.003***	0.035±0.003

Table 277: Dataset GSE73747 (D44). Imputation performance on **MNAR:mid** type missing values.

	MAE		RMSE	
Method	M-value	B-value	M-value	B-value
mean	0.054±0.001	0.052±0.001*	0.081±0.001	0.078±0.001*
softImpute	0.042±0.001	0.043±0.008*	0.066±0.001*	0.071±0.022
impute.knn	0.040±0.003*	0.050±0.007	0.068±0.009*	0.091±0.015
imputePCA	0.043±0.001	0.042±0.001*	0.066±0.001	0.064±0.001*
SVDmiss	0.085±0.002	0.045±0.001*	0.182±0.005	0.078±0.002*
missForest	0.048±0.001	0.046±0.001*	0.073±0.001	0.069±0.001*
methyLImp	**0.039±0.001**	**0.039±0.001**	0.063±0.001	**0.062±0.001***

Table 278: Dataset GSE73747 (D44). Imputation performance on **MNAR:high** type missing values.

	MAE		RMSE	
Method	M-value	B-value	M-value	B-value
mean	0.026±0.001*	0.026±0.001	0.046±0.002*	0.047±0.002
softImpute	0.022±0.001*	0.027±0.017	0.040±0.003*	0.052±0.050
impute.knn	0.067±0.023*	0.124±0.035	0.141±0.038*	0.205±0.035
imputePCA	0.020±0.001*	0.021±0.001	0.037±0.002*	0.037±0.002
SVDmiss	0.047±0.013	0.025±0.001*	0.101±0.033	0.049±0.004*
missForest	0.022±0.001*	0.023±0.001	0.041±0.002*	0.041±0.002
methyLImp	**0.019±0.001***	0.019±0.001	**0.036±0.002***	0.036±0.002

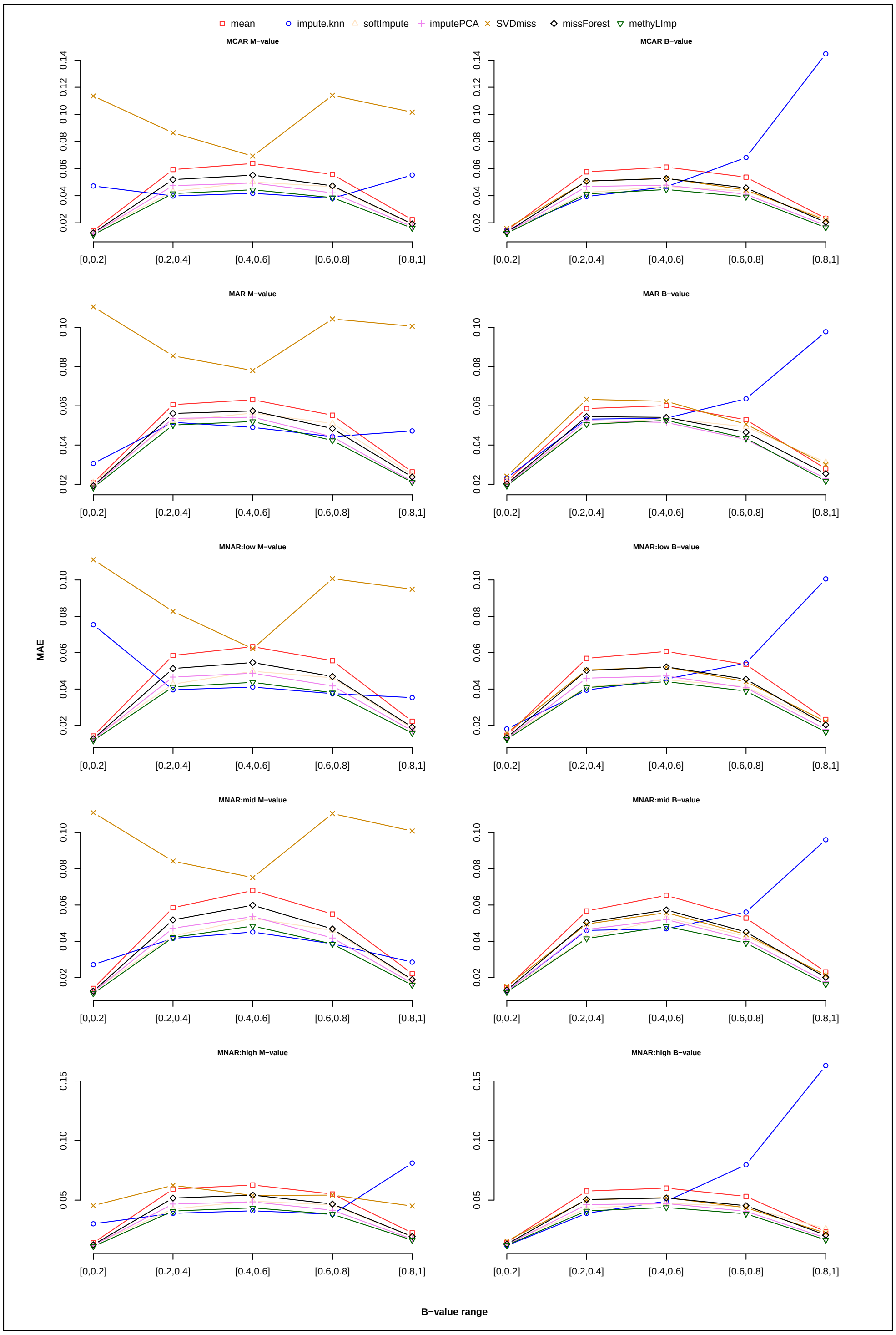

Figure 91: Dataset GSE73747 (D44). MAE imputation performances with respect to B-value range.

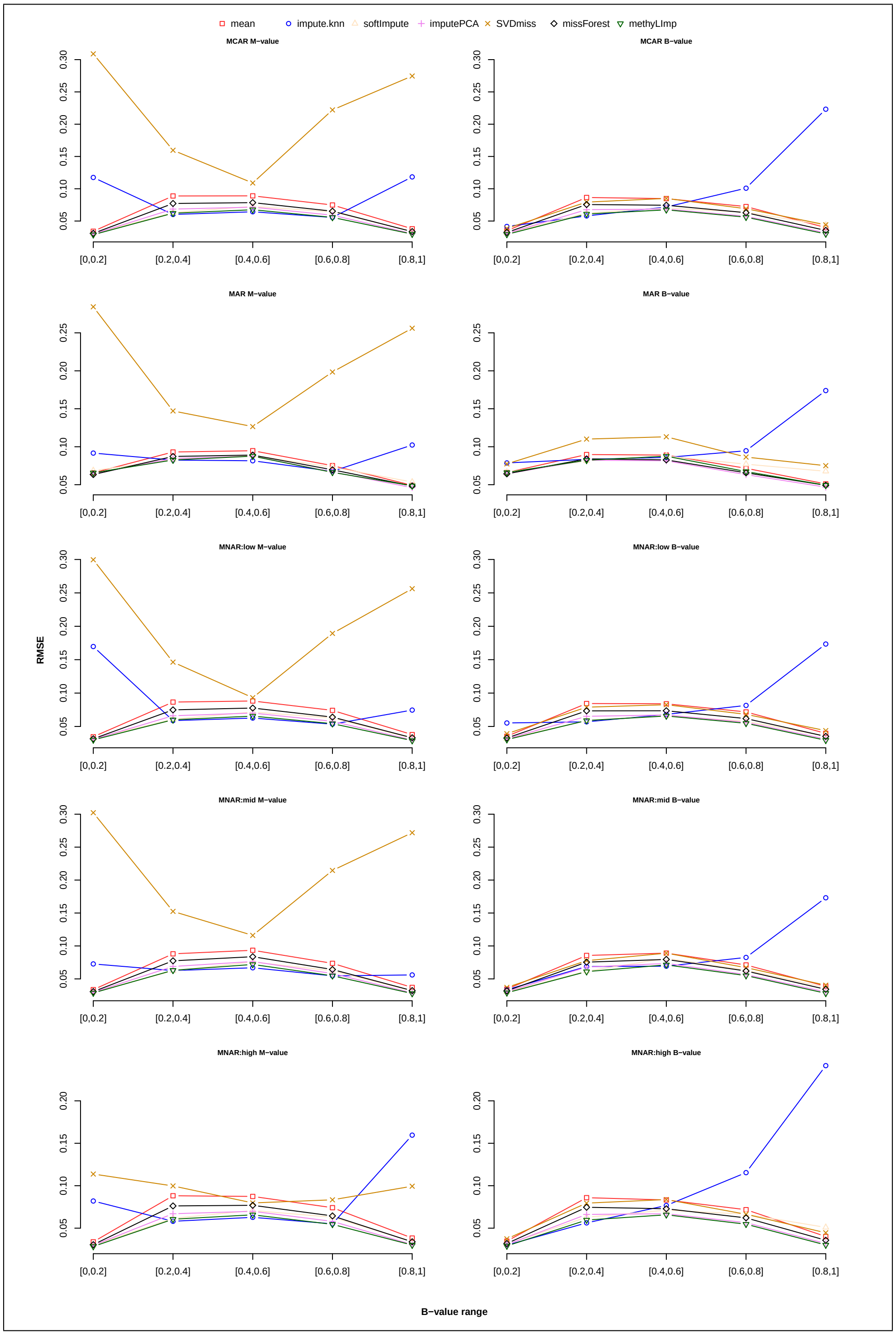

Figure 92: Dataset GSE73747 (D44). RMSE imputation performances with respect to B-value range.

2.45 GSE79122 (D45) - Brain - Normal - 7 samples

Method	Avg time (sec)	Avg RAM (Mb)
mean	< 1	6
softImpute	< 1	41
imputePCA	18	141
impute.knn	< 1	41
SVDmiss	14	3808
methyLImp	4	125
missForest	1051	236

Table 279: Dataset GSE79122 (D45). Average time and memory usage.

Table 280: Dataset GSE79122 (D45). Imputation performance on **MCAR** type missing values.

	MAE		RMSE	
Method	M-value	B-value	M-value	B-value
mean	0.045±0.001	0.044±0.001*	0.069±0.002	0.066±0.001*
softImpute	0.065±0.003	0.032±0.012*	0.113±0.007	0.059±0.034*
impute.knn	0.050±0.012*	0.099±0.023	0.103±0.024*	0.171±0.027
imputePCA	0.027±0.001	**0.026±0.001***	0.053±0.002	**0.047±0.002***
SVDmiss	0.056±0.004	0.033±0.001*	0.099±0.008	0.060±0.004*
missForest	0.032±0.001	0.032±0.001*	0.056±0.002	0.053±0.001*
methyLImp	0.046±0.001	0.027±0.001*	0.067±0.002	0.056±0.003*

Table 281: Dataset GSE79122 (D45). Imputation performance on **MAR** type missing values.

	MAE		RMSE	
Method	M-value	B-value	M-value	B-value
mean	0.063±0.001	0.061±0.001*	0.105±0.003	0.096±0.002*
softImpute	0.085±0.003	0.050±0.012*	0.148±0.007	0.105±0.029*
impute.knn	0.061±0.007*	0.100±0.016	0.116±0.013*	0.168±0.021
imputePCA	0.045±0.001	**0.042±0.001***	0.093±0.004	**0.081±0.003***
SVDmiss	0.080±0.007	0.049±0.001*	0.141±0.012	0.103±0.006*
missForest	0.050±0.001	0.048±0.001*	0.095±0.003	0.084±0.003*
methyLImp	0.063±0.001	0.044±0.001*	0.102±0.003	0.096±0.004*

Table 282: Dataset GSE79122 (D45). Imputation performance on **MNAR:low** type missing values.

	MAE		RMSE	
Method	M-value	B-value	M-value	B-value
mean	0.034±0.001*	0.036±0.001	0.058±0.002*	0.059±0.002
softImpute	0.051±0.002	0.023±0.001*	0.101±0.006	0.049±0.004*
impute.knn	0.051±0.018	0.045±0.010*	0.110±0.032	0.105±0.020
imputePCA	0.022±0.001	**0.021±0.001***	0.055±0.004	**0.045±0.003***
SVDmiss	0.055±0.011	0.027±0.001*	0.124±0.020	0.054±0.006*
missForest	0.025±0.001*	0.025±0.001	0.049±0.003	0.049±0.002
methyLImp	0.036±0.001	0.024±0.001*	0.057±0.002*	0.067±0.006

Table 283: Dataset GSE79122 (D45). Imputation performance on **MNAR:mid** type missing values.

	MAE		RMSE	
Method	M-value	B-value	M-value	B-value
mean	0.076±0.001	0.072±0.001*	0.104±0.002	0.095±0.001*
softImpute	0.105±0.003	0.055±0.002*	0.152±0.006	0.085±0.008*
impute.knn	**0.044±0.002***	0.062±0.010	**0.070±0.005***	0.109±0.018
imputePCA	0.047±0.001	0.044±0.001*	0.077±0.002	**0.068±0.002***
SVDmiss	0.087±0.004	0.049±0.001*	0.128±0.007	0.084±0.004*
missForest	0.057±0.001	0.053±0.001*	0.086±0.002	0.077±0.002*
methyLImp	0.068±0.001	0.045±0.001*	0.094±0.002	0.074±0.003*

Table 284: Dataset GSE79122 (D45). Imputation performance on **MNAR:high** type missing values.

	MAE		RMSE	
Method	M-value	B-value	M-value	B-value
mean	0.040±0.001*	0.040±0.001	0.063±0.003	0.060±0.002*
softImpute	0.054±0.004	0.029±0.003*	0.094±0.010	0.059±0.016*
impute.knn	0.088±0.027*	0.161±0.035	0.167±0.039*	0.234±0.031
imputePCA	0.024±0.001	**0.023±0.001***	0.048±0.004	**0.042±0.002***
SVDmiss	0.051±0.004	0.031±0.001*	0.085±0.007	0.056±0.004*
missForest	0.029±0.001*	0.029±0.001	0.051±0.003	0.048±0.002*
methyLImp	0.045±0.001	0.024±0.001*	0.066±0.003	0.050±0.004*

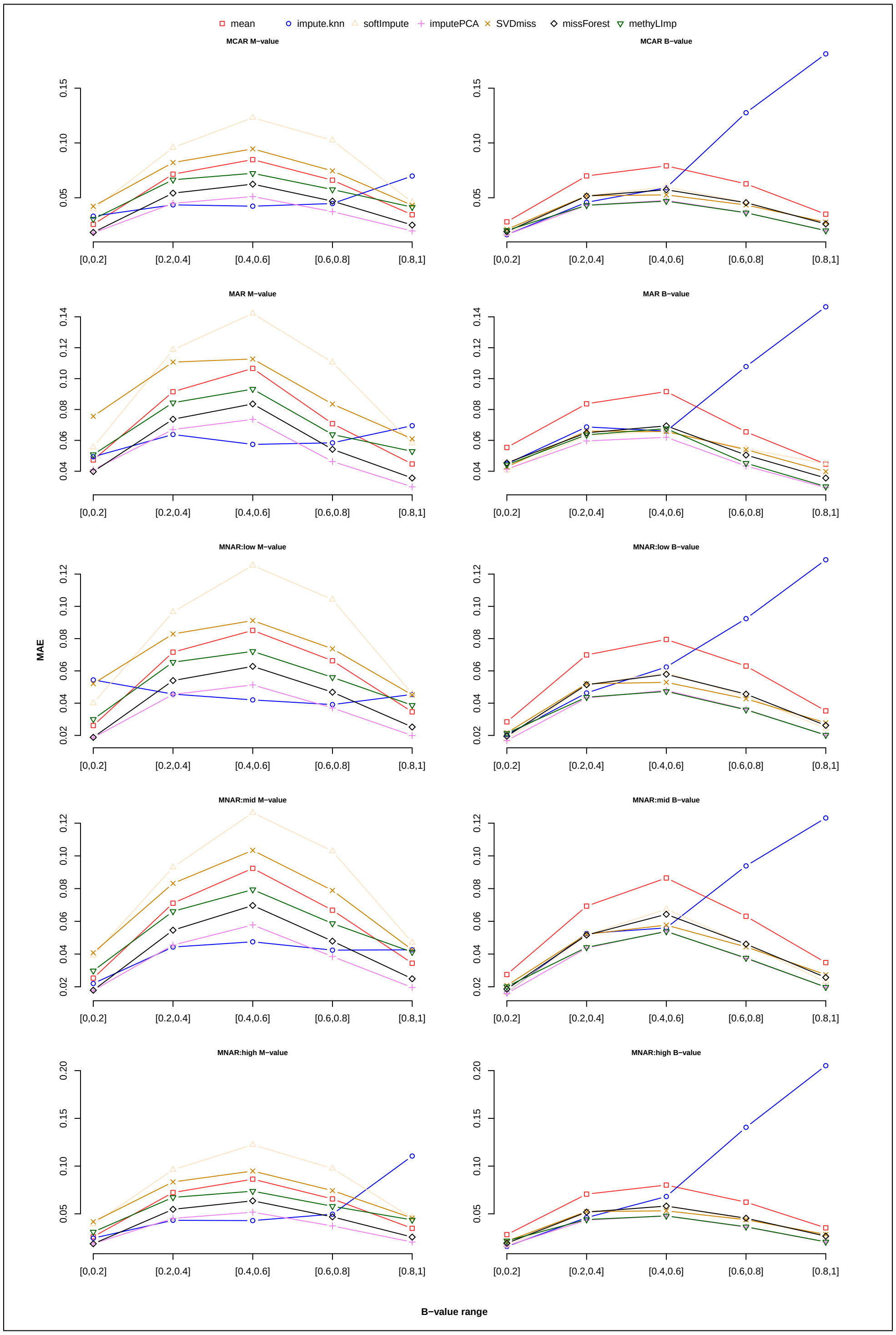

Figure 93: Dataset GSE79122 (D45). MAE imputation performances with respect to B-value range.

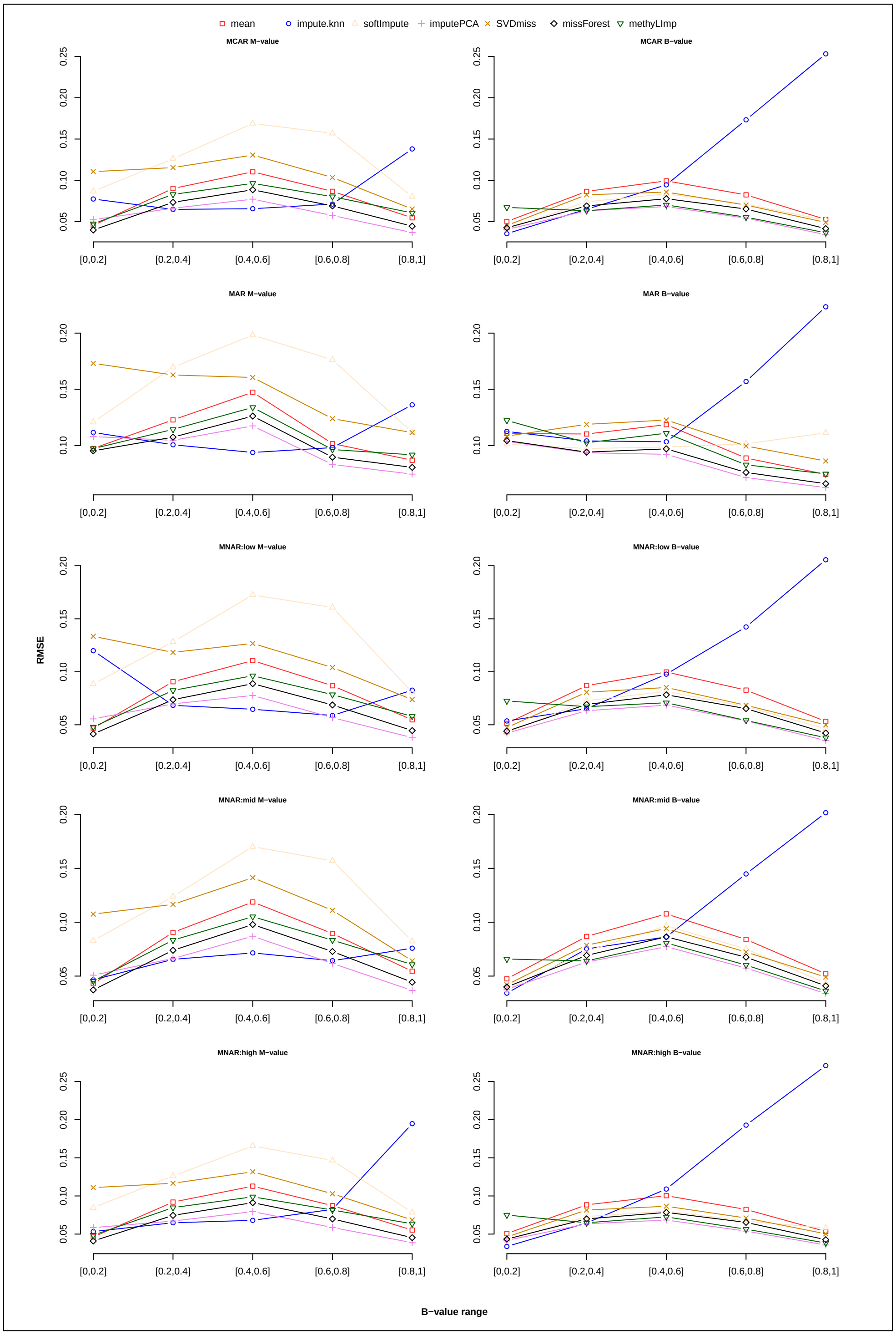

Figure 94: Dataset GSE79122 (D45). RMSE imputation performances with respect to B-value range.

2.46 GSE80970 (D46) - Prefrontal Cortex - Normal - 68 samples

Method	Avg time (sec)	Avg RAM (Mb)
mean	< 1	56
softImpute	1	118
imputePCA	27	288
impute.knn	3	142
SVDmiss	292	5142
methyLImp	3141	149
missForest	193586	381

Table 285: Dataset GSE80970 (D46). Average time and memory usage.

Table 286: Dataset GSE80970 (D46). Imputation performance on **MCAR** type missing values.

	MAE		RMSE	
Method	M-value	B-value	M-value	B-value
mean	0.026±0.001*	0.026±0.001	0.043±0.001	0.042±0.001*
softImpute	0.023±0.001	0.022±0.001*	0.038±0.001	0.036±0.001*
impute.knn	0.019±0.001*	0.022±0.001	0.035±0.002*	0.046±0.005
imputePCA	0.022±0.001*	0.022±0.001	0.039±0.001	0.039±0.001
SVDmiss	0.021±0.001*	0.022±0.001	0.037±0.001*	0.041±0.001
missForest	**0.019±0.001***	0.019±0.001	**0.032±0.001**	**0.032±0.001**
methyLImp	**0.018±0.001***	0.019±0.001	0.033±0.001*	0.034±0.001

Table 287: Dataset GSE80970 (D46). Imputation performance on **MAR** type missing values.

	MAE		RMSE	
Method	M-value	B-value	M-value	B-value
mean	0.040±0.001*	0.040±0.001	0.069±0.001	0.068±0.001*
softImpute	0.033±0.002	0.029±0.001*	0.059±0.005	0.051±0.001*
impute.knn	0.027±0.001*	0.032±0.002	0.052±0.002*	0.069±0.009
imputePCA	0.037±0.001*	0.037±0.001	0.068±0.001	0.067±0.001*
SVDmiss	0.027±0.001*	0.028±0.001	0.052±0.001*	0.055±0.001
missForest	**0.025±0.001***	0.026±0.001	0.049±0.001	**0.049±0.001***
methyLImp	0.033±0.001	0.033±0.001*	0.065±0.001	0.064±0.001*

Table 288: Dataset GSE80970 (D46). Imputation performance on **MNAR:low** type missing values.

	MAE		RMSE	
Method	M-value	B-value	M-value	B-value
mean	0.019±0.001*	0.020±0.001	0.035±0.001*	0.037±0.001
softImpute	0.017±0.001	0.017±0.001*	0.031±0.001	0.030±0.001*
impute.knn	0.014±0.001*	0.016±0.001	0.030±0.003*	0.037±0.004
imputePCA	0.016±0.001*	0.017±0.001	0.033±0.001*	0.035±0.001
SVDmiss	0.015±0.001*	0.016±0.001	0.032±0.001*	0.034±0.001
missForest	**0.014±0.001***	0.014±0.001	**0.027±0.001***	0.028±0.001
methyLImp	**0.014±0.001***	0.014±0.001	**0.027±0.001***	0.027±0.001

Table 289: Dataset GSE80970 (D46). Imputation performance on **MNAR:mid** type missing values.

	MAE		RMSE	
Method	M-value	B-value	M-value	B-value
mean	0.042±0.001	0.040±0.001*	0.067±0.001	0.062±0.001*
softImpute	0.037±0.001	0.033±0.001*	0.057±0.002	0.052±0.001*
impute.knn	0.028±0.001*	0.030±0.001	0.048±0.001*	0.053±0.003
imputePCA	0.036±0.001	0.035±0.001*	0.063±0.001	0.058±0.001*
SVDmiss	0.031±0.001*	0.032±0.001	0.051±0.001*	0.055±0.001
missForest	0.028±0.001	0.028±0.001*	0.048±0.001	**0.047±0.001***
methyLImp	**0.027±0.001***	0.028±0.001	**0.047±0.001***	0.047±0.001

Table 290: Dataset GSE80970 (D46). Imputation performance on **MNAR:high** type missing values.

	MAE		RMSE	
Method	M-value	B-value	M-value	B-value
mean	0.022±0.001*	0.023±0.001	0.035±0.001*	0.036±0.001
softImpute	0.020±0.001	0.020±0.001*	0.032±0.001	0.031±0.001*
impute.knn	0.017±0.001*	0.024±0.003	0.030±0.003*	0.057±0.010
imputePCA	0.018±0.001*	0.019±0.001	0.032±0.001*	0.033±0.001
SVDmiss	0.018±0.001*	0.020±0.001	0.032±0.001*	0.037±0.001
missForest	0.016±0.001*	0.017±0.001	**0.028±0.001***	0.028±0.001
methyLImp	**0.016±0.001***	0.017±0.001	**0.028±0.001***	0.028±0.001

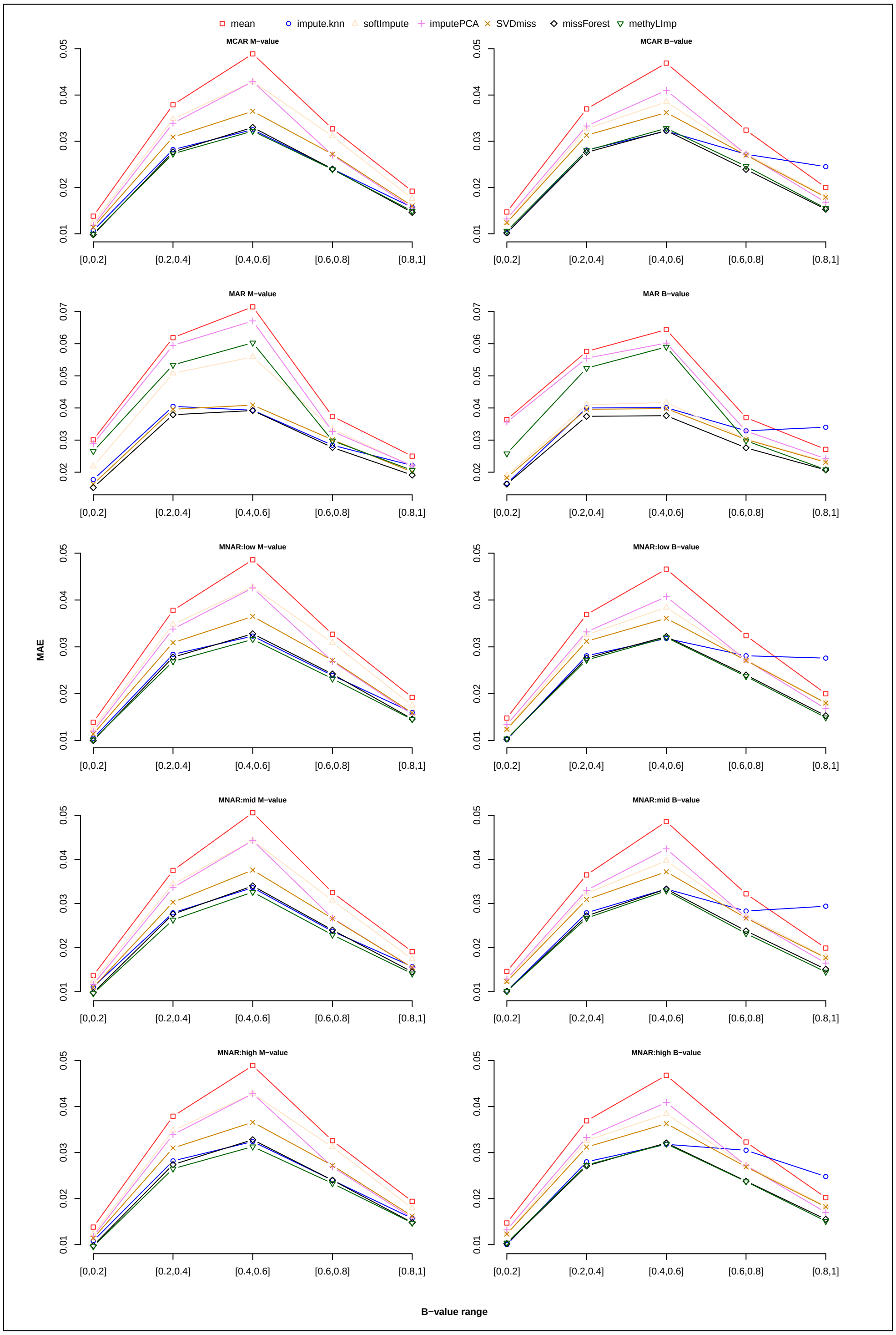

Figure 95: Dataset GSE80970 (D46). MAE imputation performances with respect to B-value range.

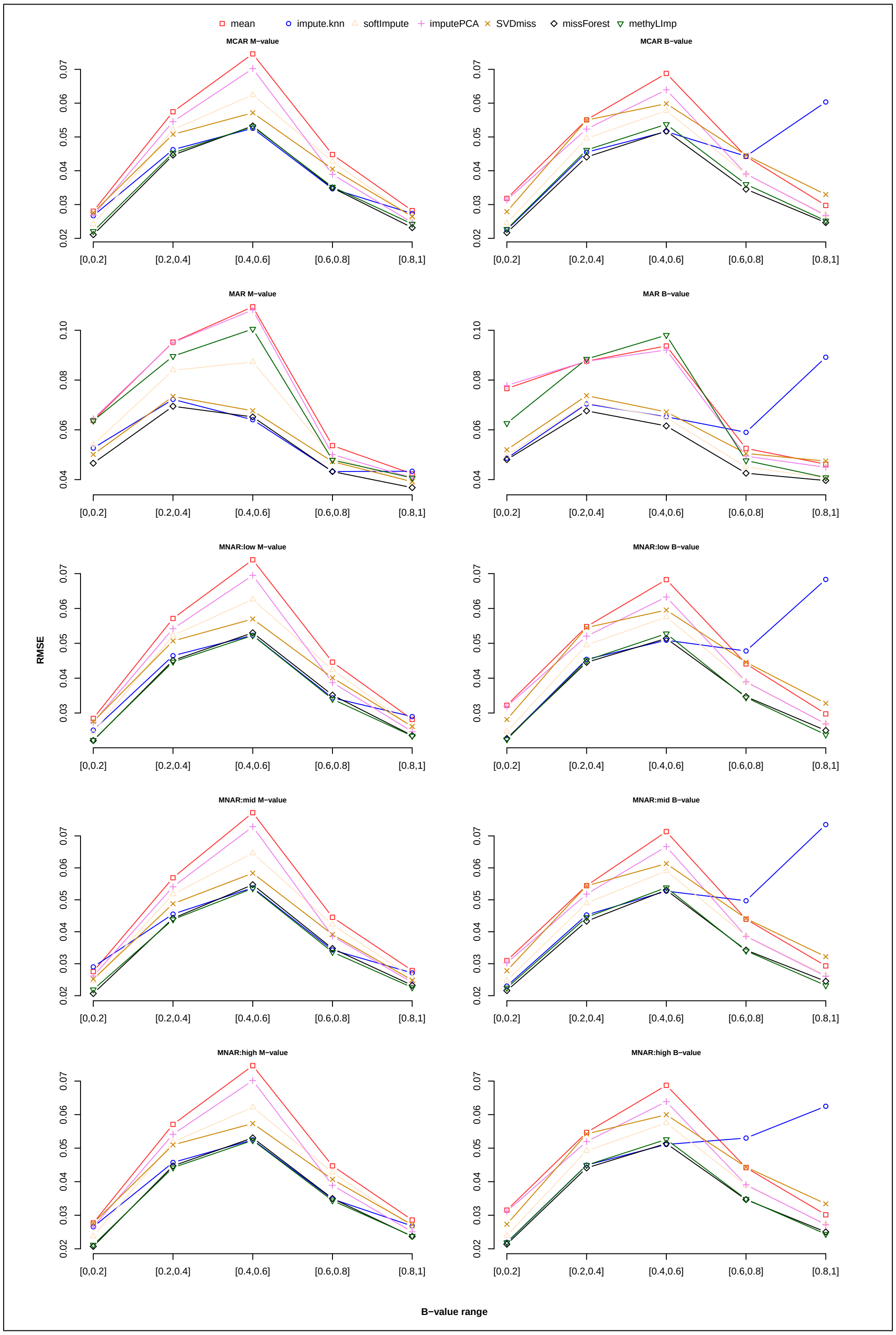

Figure 96: Dataset GSE80970 (D46). RMSE imputation performances with respect to B-value range.

2.47 GSE82218 (D47) - Blood - Normal - 25 samples

Method	Avg time (sec)	Avg RAM (Mb)
mean	< 1	21
softImpute	< 1	62
imputePCA	9	188
impute.knn	1	72
SVDmiss	95	4259
methyLImp	156	101
missForest	20546	157

Table 291: Dataset GSE82218 (D47). Average time and memory usage.

Table 292: Dataset GSE82218 (D47). Imputation performance on **MCAR** type missing values.

	MAE		RMSE	
Method	M-value	B-value	M-value	B-value
mean	0.022±0.001	0.022±0.001*	0.038±0.001	0.038±0.001*
softImpute	0.020±0.001	0.019±0.001*	0.035±0.001	0.033±0.001*
impute.knn	0.027±0.005*	0.038±0.009	0.071±0.014*	0.091±0.017
imputePCA	0.018±0.001	0.018±0.001*	0.036±0.001	0.035±0.001*
SVDmiss	0.036±0.002	0.017±0.001*	0.109±0.006	0.032±0.001*
missForest	0.017±0.001*	0.017±0.001	0.032±0.001	0.032±0.001
methyLImp	**0.016±0.001***	0.017±0.001	**0.031±0.001***	0.032±0.001

Table 293: Dataset GSE82218 (D47). Imputation performance on **MAR** type missing values.

	MAE		RMSE	
Method	M-value	B-value	M-value	B-value
mean	0.035±0.001	0.034±0.001*	0.064±0.001	0.063±0.001*
softImpute	0.031±0.001	0.027±0.001*	0.060±0.003	**0.052±0.001***
impute.knn	0.030±0.003*	0.033±0.003	0.065±0.008*	0.072±0.008
imputePCA	0.032±0.001	0.031±0.001*	0.066±0.001	0.064±0.001*
SVDmiss	**0.026±0.004**	**0.025±0.001**	0.056±0.011	**0.052±0.001***
missForest	0.027±0.001*	0.028±0.001	0.054±0.001	0.054±0.001
methyLImp	0.029±0.001*	0.029±0.001	0.060±0.002	0.059±0.002*

Table 294: Dataset GSE82218 (D47). Imputation performance on **MNAR:low** type missing values.

	MAE		RMSE	
Method	M-value	B-value	M-value	B-value
mean	0.018±0.001*	0.018±0.001	0.035±0.001*	0.036±0.001
softImpute	0.016±0.001	0.015±0.001*	0.030±0.001	0.029±0.001*
impute.knn	0.021±0.006	0.020±0.003	0.060±0.019	0.057±0.011
imputePCA	0.015±0.001*	0.015±0.001	0.034±0.001*	0.034±0.001
SVDmiss	0.038±0.001	0.014±0.001*	0.132±0.005	0.028±0.001*
missForest	0.014±0.001*	0.014±0.001	0.028±0.001*	0.029±0.001
methyLImp	**0.013±0.001***	0.013±0.001	**0.027±0.001***	0.027±0.001

Table 295: Dataset GSE82218 (D47). Imputation performance on **MNAR:mid** type missing values.

	MAE		RMSE	
Method	M-value	B-value	M-value	B-value
mean	0.041±0.001	0.039±0.001*	0.063±0.001	0.058±0.001*
softImpute	0.037±0.001	0.032±0.001*	0.059±0.002	0.050±0.001*
impute.knn	0.030±0.002*	0.032±0.002	0.056±0.005*	0.061±0.007
imputePCA	0.036±0.001	0.034±0.001*	0.061±0.001	0.055±0.001*
SVDmiss	0.030±0.001	**0.029±0.001***	0.050±0.001	**0.048±0.001***
missForest	0.031±0.001	0.030±0.001*	0.051±0.001	**0.048±0.001***
methyLImp	0.030±0.001*	0.030±0.001	0.051±0.001*	0.051±0.001

Table 296: Dataset GSE82218 (D47). Imputation performance on **MNAR:high** type missing values.

	MAE		RMSE	
Method	M-value	B-value	M-value	B-value
mean	0.019±0.001*	0.020±0.001	0.032±0.001*	0.033±0.001
softImpute	0.017±0.001	0.017±0.002*	0.029±0.001	0.029±0.008*
impute.knn	0.029±0.007*	0.048±0.012	0.076±0.019*	0.111±0.020
imputePCA	0.016±0.001*	0.016±0.001	0.030±0.001*	0.030±0.001
SVDmiss	0.036±0.001	0.015±0.001*	0.110±0.005	0.028±0.001*
missForest	0.015±0.001*	0.015±0.001	0.027±0.001*	0.028±0.001
methyLImp	**0.014±0.001***	0.015±0.001	**0.027±0.001***	0.027±0.001

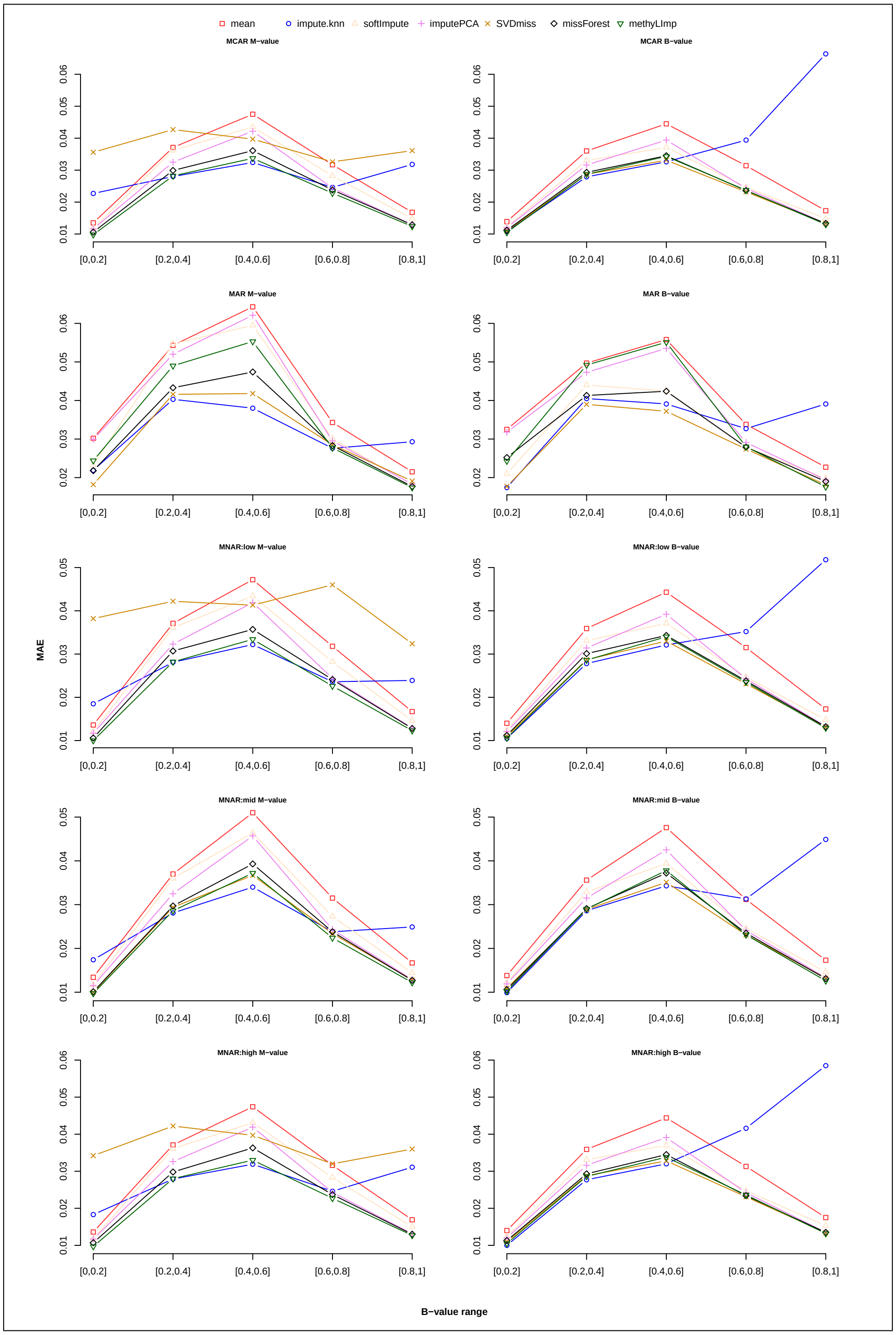

Figure 97: Dataset GSE82218 (D47). MAE imputation performances with respect to B-value range.

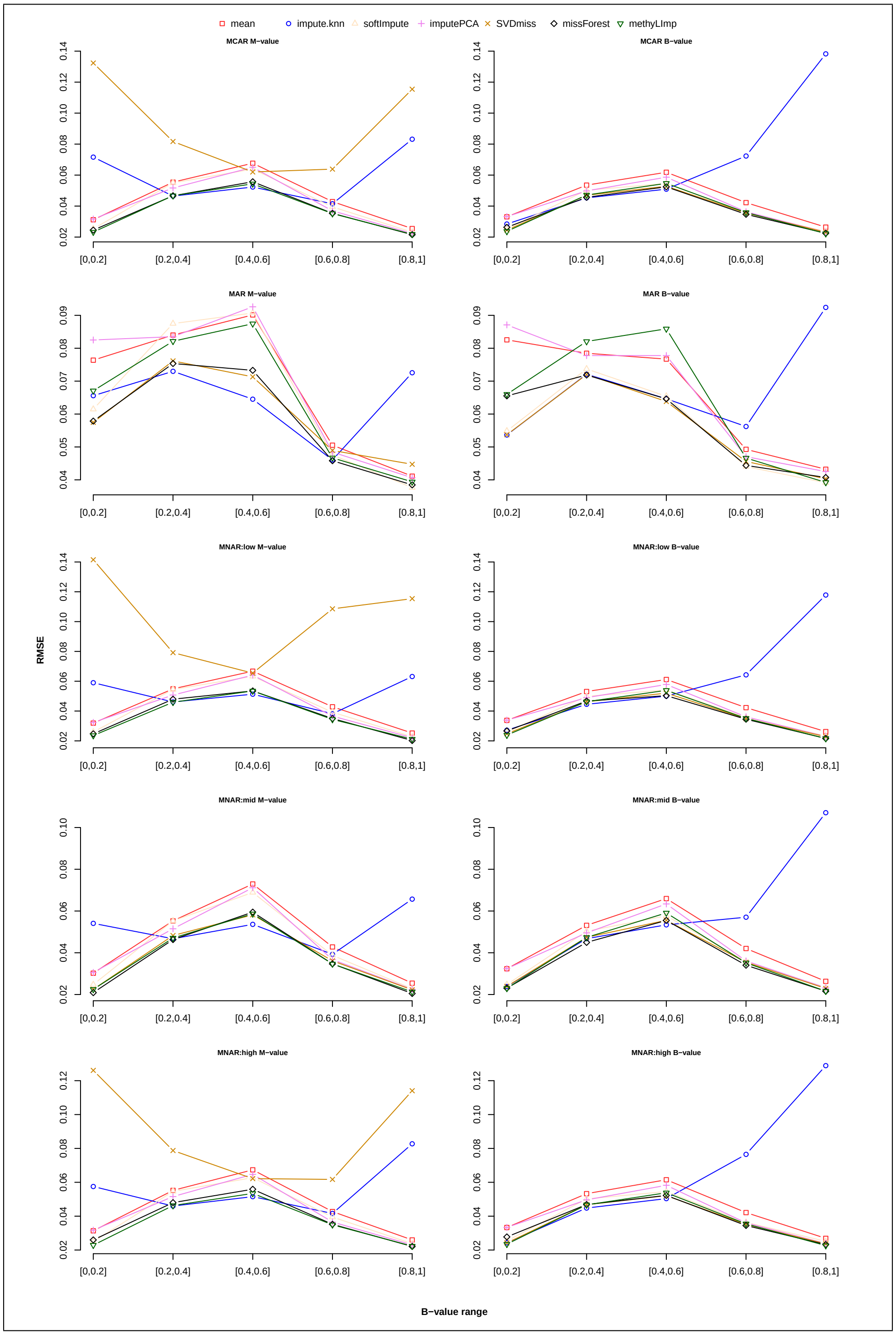

Figure 98: Dataset GSE82218 (D47). RMSE imputation performances with respect to B-value range.

2.48 GSE84003 (D48) - Blood - Normal - 6 samples

Method	Avg time (sec)	Avg RAM (Mb)
mean	< 1	5
softImpute	< 1	41
imputePCA	4	142
impute.knn	< 1	41
SVDmiss	12	3767
methyLImp	3	121
missForest	421	246

Table 297: Dataset GSE84003 (D48). Average time and memory usage.

Table 298: Dataset GSE84003 (D48). Imputation performance on **MCAR** type missing values.

	MAE		RMSE	
Method	M-value	B-value	M-value	B-value
mean	0.021±0.001	0.021±0.001*	0.039±0.002	0.038±0.002*
softImpute	0.029±0.002*	0.057±0.030	0.063±0.007*	0.132±0.081
impute.knn	0.069±0.019*	0.118±0.027	0.144±0.030*	0.202±0.030
imputePCA	0.021±0.001	**0.020±0.001***	0.039±0.002	**0.038±0.002***
SVDmiss	0.046±0.003	0.034±0.001*	0.093±0.008	0.069±0.006*
missForest	0.021±0.001	0.021±0.001*	0.039±0.002	0.038±0.002*
methyLImp	0.026±0.001	**0.020±0.001***	0.042±0.002	0.039±0.002*

Table 299: Dataset GSE84003 (D48). Imputation performance on **MAR** type missing values.

	MAE		RMSE	
Method	M-value	B-value	M-value	B-value
mean	0.032±0.001	0.032±0.001*	0.070±0.004	**0.066±0.003***
softImpute	0.043±0.002*	0.076±0.029	0.094±0.007*	0.165±0.069
impute.knn	0.069±0.015*	0.109±0.026	0.139±0.024*	0.187±0.029
imputePCA	0.032±0.001	**0.032±0.001***	0.070±0.004	0.066±0.003*
SVDmiss	0.057±0.003	0.051±0.002*	0.112±0.007	0.113±0.006
missForest	0.032±0.001	0.032±0.001*	0.070±0.004	0.066±0.003*
methyLImp	0.037±0.001	0.032±0.001*	0.071±0.004	0.070±0.004*

Table 300: Dataset GSE84003 (D48). Imputation performance on **MNAR:low** type missing values.

	MAE		RMSE	
Method	M-value	B-value	M-value	B-value
mean	0.017±0.001*	0.017±0.001	0.036±0.004	0.036±0.003
softImpute	0.026±0.003*	0.037±0.017	0.072±0.011*	0.101±0.062
impute.knn	0.075±0.028	0.052±0.015*	0.153±0.039	0.127±0.029*
imputePCA	0.016±0.001*	0.016±0.001	0.036±0.004	**0.036±0.003**
SVDmiss	0.041±0.003	0.027±0.001*	0.107±0.007	0.063±0.007*
missForest	0.017±0.001*	0.017±0.001	0.036±0.004	0.036±0.003
methyLImp	0.021±0.001	**0.016±0.001***	0.039±0.003	**0.036±0.004***

Table 301: Dataset GSE84003 (D48). Imputation performance on **MNAR:mid** type missing values.

	MAE		RMSE	
Method	M-value	B-value	M-value	B-value
mean	0.039±0.001	0.038±0.001*	0.065±0.002	0.061±0.002*
softImpute	0.051±0.002*	0.081±0.025	0.085±0.005*	0.154±0.057
impute.knn	0.048±0.005*	0.065±0.013	0.083±0.013*	0.124±0.025
imputePCA	0.039±0.001	**0.038±0.001***	0.065±0.002	**0.061±0.002***
SVDmiss	0.066±0.002	0.058±0.001*	0.106±0.005	0.104±0.005*
missForest	0.039±0.001	0.038±0.001*	0.065±0.002	0.061±0.002*
methyLImp	0.038±0.001*	0.039±0.001	0.062±0.002*	0.065±0.002

Table 302: Dataset GSE84003 (D48). Imputation performance on **MNAR:high** type missing values.

	MAE		RMSE	
Method	M-value	B-value	M-value	B-value
mean	0.019±0.001*	0.019±0.001	0.037±0.003	0.035±0.002*
softImpute	0.026±0.001*	0.079±0.044	0.054±0.006*	0.182±0.113
impute.knn	0.123±0.037*	0.182±0.038	0.217±0.043*	0.262±0.032
imputePCA	0.019±0.001*	0.019±0.001	0.036±0.003	**0.035±0.002***
SVDmiss	0.039±0.003	0.033±0.001*	0.077±0.007	0.065±0.006*
missForest	0.019±0.001*	0.019±0.001	0.037±0.003	0.035±0.002*
methyLImp	0.026±0.001	**0.019±0.001***	0.041±0.003	0.036±0.003*

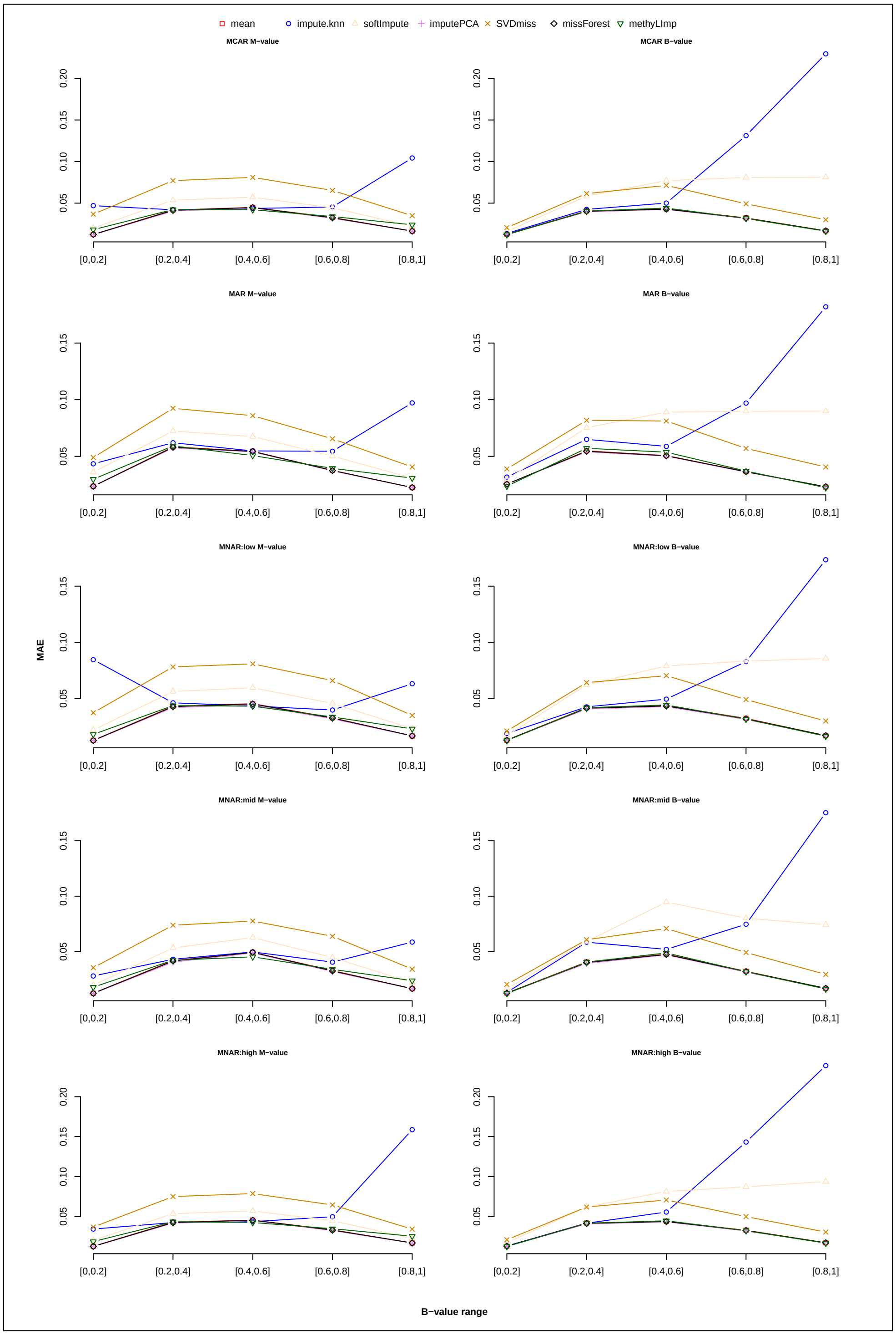

Figure 99: Dataset GSE84003 (D48). MAE imputation performances with respect to B-value range.

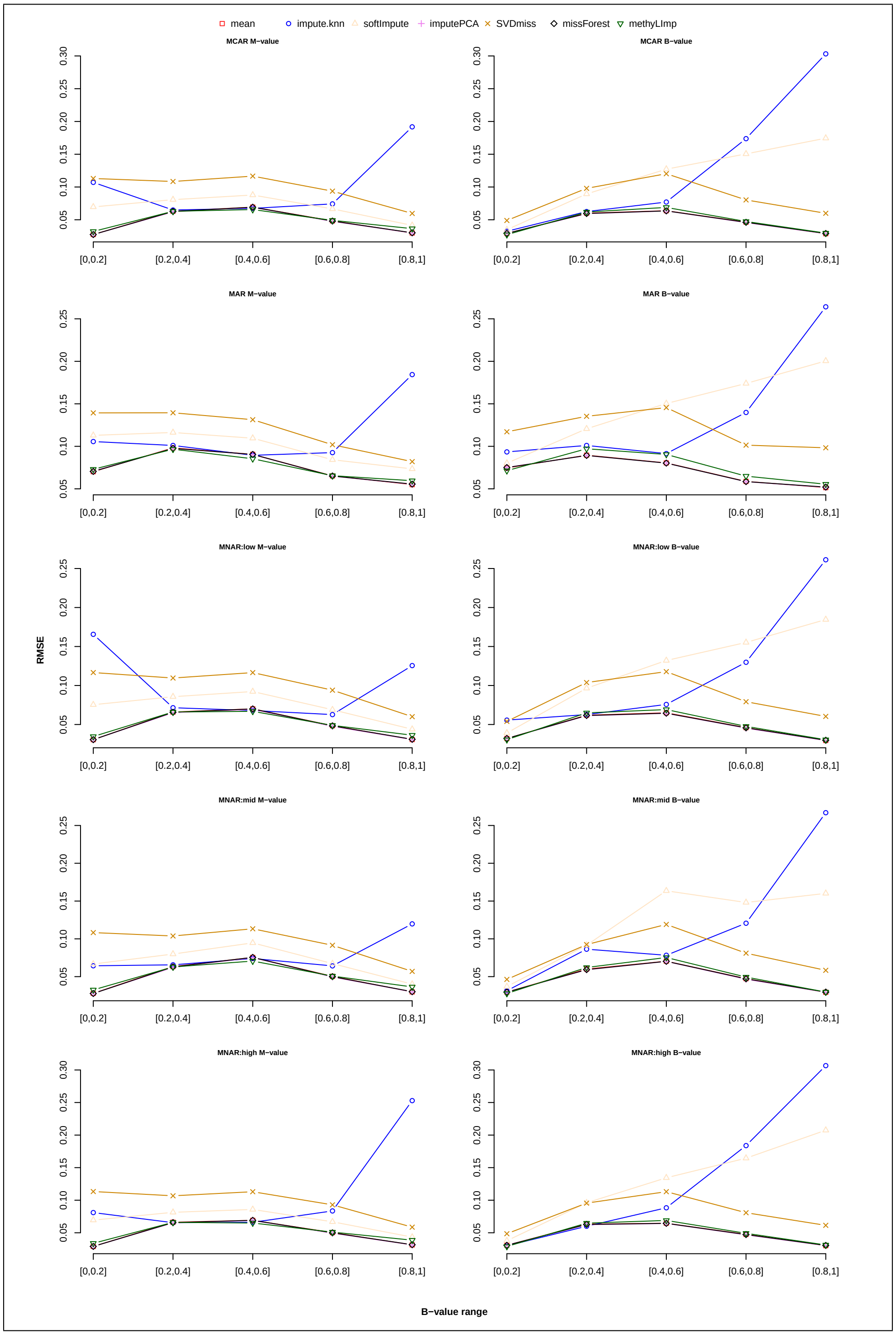

Figure 100: Dataset GSE84003 (D48). RMSE imputation performances with respect to B-value range.

2.49 GSE88821 (D49) - Colon (Ascending, Descending, Sigmoid), Rectum - Cancer - 63 samples

Method	Avg time (sec)	Avg RAM (Mb)
mean	< 1	52
softImpute	1	130
imputePCA	20	275
impute.knn	2	141
SVDmiss	247	4780
methyLImp	2206	143
missForest	189823	345

Table 303: Dataset GSE88821 (D49). Average time and memory usage.

Table 304: Dataset GSE88821 (D49). Imputation performance on **MCAR** type missing values.

	MAE		RMSE	
Method	M-value	B-value	M-value	B-value
mean	0.064±0.001*	0.064±0.001	0.104±0.001	0.102±0.001*
softImpute	0.057±0.001	0.051±0.001*	0.090±0.001	0.082±0.001*
impute.knn	0.039±0.001*	0.040±0.001	0.065±0.001	0.065±0.003
imputePCA	0.046±0.001*	0.046±0.001	0.078±0.001	0.076±0.001*
SVDmiss	0.053±0.001	0.042±0.001*	0.090±0.002	0.069±0.001*
missForest	0.034±0.001*	0.034±0.001	0.057±0.001	**0.055±0.001***
methyLImp	0.056±0.001	**0.031±0.001***	0.103±0.003	0.057±0.001*

Table 305: Dataset GSE88821 (D49). Imputation performance on **MAR** type missing values.

	MAE		RMSE	
Method	M-value	B-value	M-value	B-value
mean	0.062±0.001*	0.063±0.001	0.102±0.001	0.100±0.001*
softImpute	0.056±0.001	0.050±0.001*	0.089±0.001	0.081±0.001*
impute.knn	0.039±0.001*	0.042±0.001	0.065±0.001*	0.071±0.006
imputePCA	0.044±0.001*	0.045±0.001	0.076±0.001	0.075±0.001*
SVDmiss	0.052±0.001	0.042±0.001*	0.089±0.002	0.068±0.001*
missForest	0.034±0.001*	0.034±0.001	0.056±0.001	0.055±0.001*
methyLImp	0.044±0.001	**0.029±0.001***	0.074±0.001	**0.051±0.001***

Table 306: Dataset GSE88821 (D49). Imputation performance on **MNAR:low** type missing values.

	MAE		RMSE	
Method	M-value	B-value	M-value	B-value
mean	0.049±0.001*	0.052±0.001	0.089±0.001*	0.092±0.001
softImpute	0.044±0.001	0.038±0.001*	0.075±0.001	0.070±0.001*
impute.knn	0.028±0.001*	0.029±0.001	0.052±0.001*	0.054±0.002
imputePCA	0.033±0.001*	0.035±0.001	0.063±0.001*	0.066±0.001
SVDmiss	0.041±0.001	0.032±0.001*	0.079±0.003	0.058±0.001*
missForest	0.025±0.001*	0.026±0.001	**0.044±0.001***	0.046±0.001
methyLImp	0.032±0.002	**0.024±0.001***	0.086±0.009	0.050±0.001*

Table 307: Dataset GSE88821 (D49). Imputation performance on **MNAR:mid** type missing values.

	MAE		RMSE	
Method	M-value	B-value	M-value	B-value
mean	0.098±0.001	0.091±0.001*	0.135±0.001	0.124±0.001*
softImpute	0.084±0.001	0.075±0.001*	0.119±0.001	0.106±0.001*
impute.knn	0.061±0.001	0.057±0.001*	0.090±0.001	0.082±0.001*
imputePCA	0.073±0.001	0.069±0.001*	0.107±0.001	0.099±0.001*
SVDmiss	0.082±0.001	0.063±0.001*	0.118±0.001	0.090±0.001*
missForest	0.054±0.001	0.050±0.001*	0.080±0.001	0.072±0.001*
methyLImp	0.083±0.001	**0.044±0.001***	0.119±0.002	**0.068±0.001***

Table 308: Dataset GSE88821 (D49). Imputation performance on **MNAR:high** type missing values.

	MAE		RMSE	
Method	M-value	B-value	M-value	B-value
mean	0.051±0.001*	0.054±0.001	0.088±0.001*	0.088±0.001
softImpute	0.049±0.001	0.043±0.001*	0.077±0.001	0.070±0.001*
impute.knn	0.033±0.001*	0.037±0.002	0.055±0.001*	0.064±0.007
imputePCA	0.037±0.001*	0.039±0.001	0.064±0.001*	0.065±0.001
SVDmiss	0.043±0.001	0.037±0.001*	0.073±0.001	0.060±0.001*
missForest	0.029±0.001*	0.030±0.001	0.047±0.001*	0.048±0.001
methyLImp	0.041±0.001	**0.026±0.001***	0.069±0.001	**0.044±0.001***

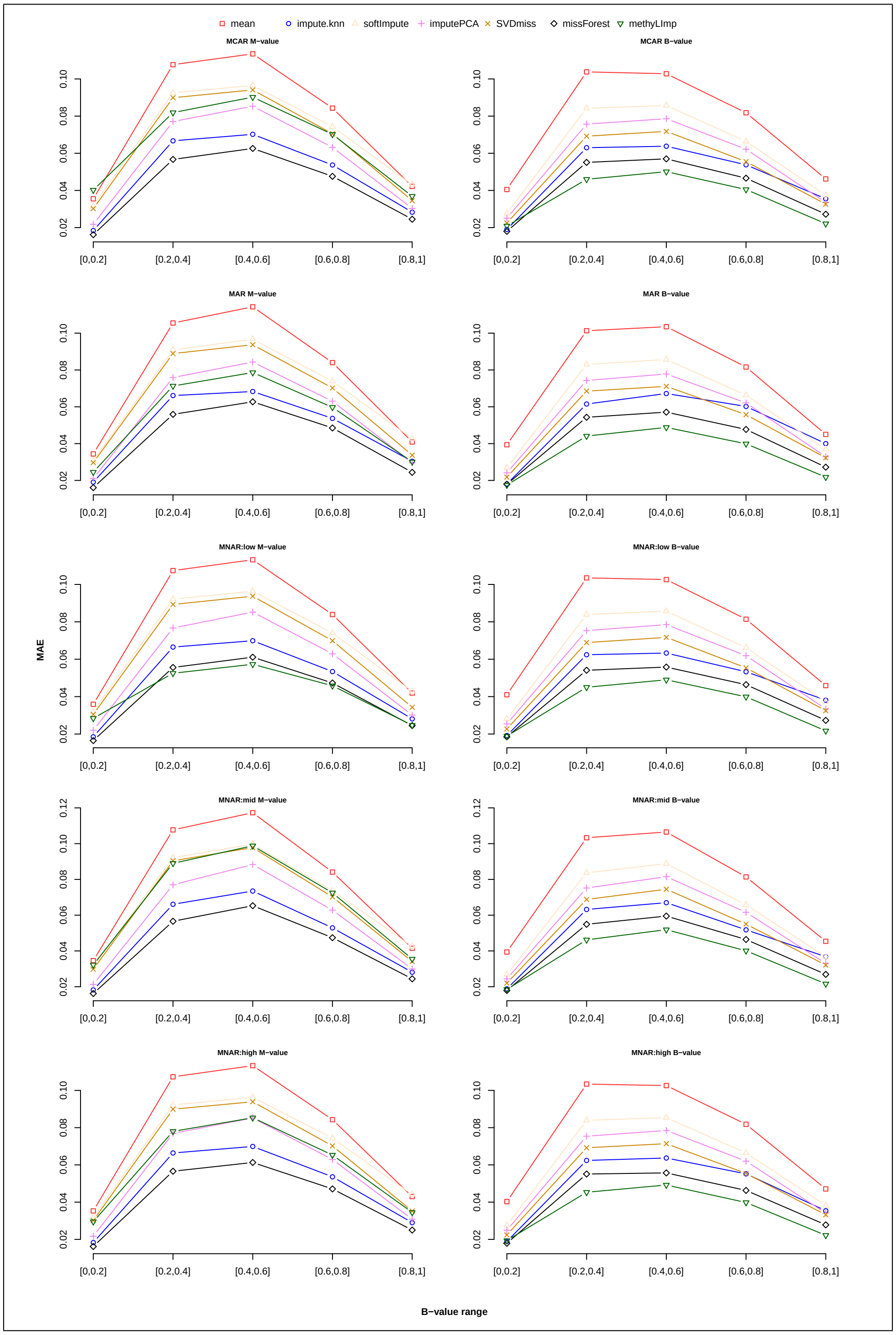

Figure 101: Dataset GSE88821 (D49). MAE imputation performances with respect to B-value range.

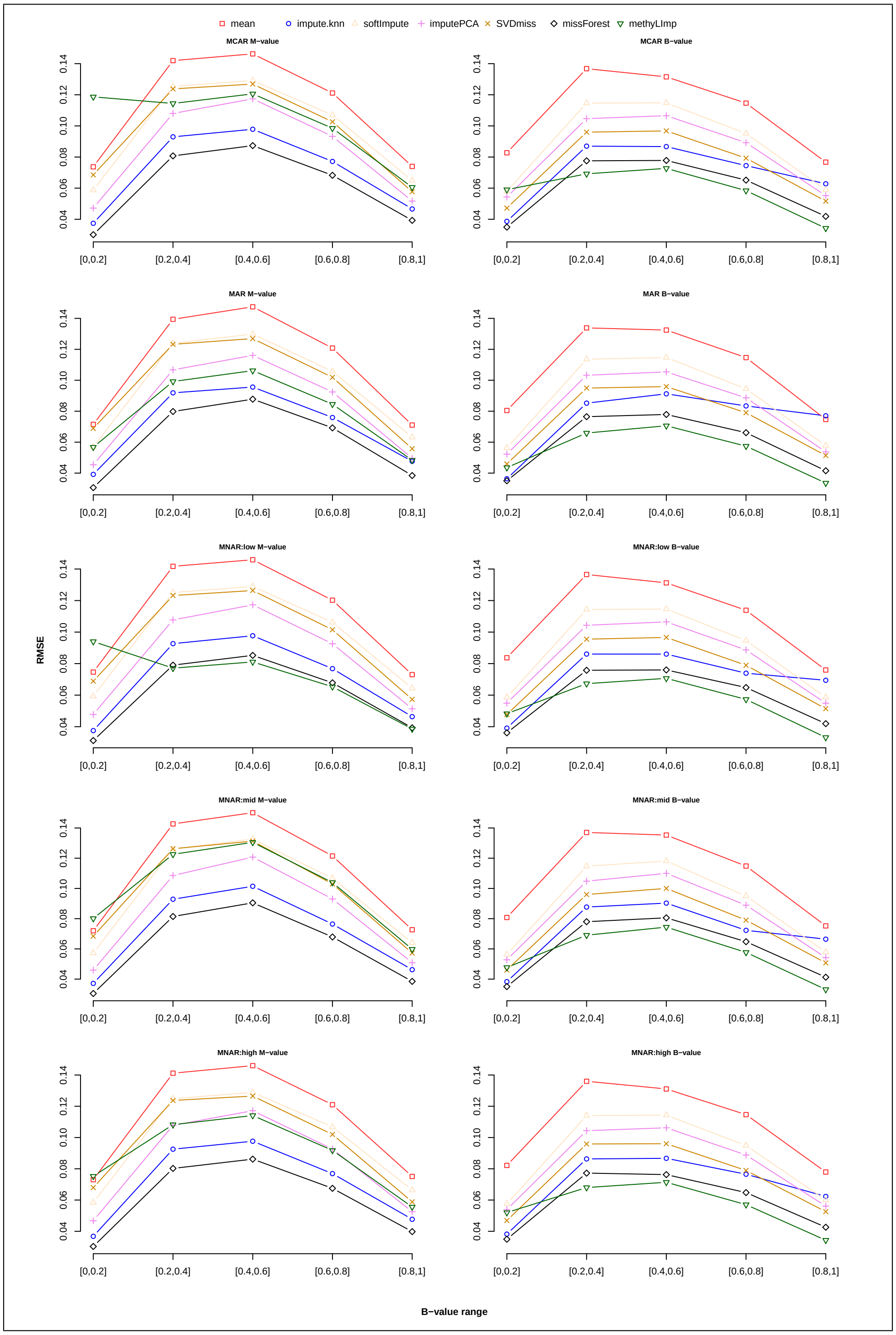

Figure 102: Dataset GSE88821 (D49). RMSE imputation performances with respect to B-value range.

2.50 GSE88821 (D50) - Colon (Ascending, Descending, Sigmoid), Rectum - Normal - 8 samples

Method	Avg time (sec)	Avg RAM (Mb)
mean	< 1	7
softImpute	< 1	41
imputePCA	6	142
impute.knn	< 1	42
SVDmiss	13	3656
methyLImp	4	122
missForest	1219	279

Table 309: Dataset GSE88821 (D50). Average time and memory usage.

Table 310: Dataset GSE88821 (D50). Imputation performance on **MCAR** type missing values.

	MAE		RMSE	
Method	M-value	B-value	M-value	B-value
mean	0.026±0.001	0.025±0.001*	0.042±0.002	0.041±0.002*
softImpute	0.043±0.009	0.037±0.017*	0.077±0.023	0.075±0.050*
impute.knn	0.041±0.010*	0.069±0.018	0.088±0.025*	0.137±0.026
imputePCA	0.024±0.001	0.024±0.001*	0.041±0.002	**0.039±0.002***
SVDmiss	0.047±0.002	0.032±0.001*	0.080±0.004	0.059±0.004*
missForest	0.025±0.001	0.025±0.001*	0.041±0.002	0.040±0.002*
methyLImp	0.032±0.001	**0.024±0.001***	0.048±0.002	0.041±0.002*

Table 311: Dataset GSE88821 (D50). Imputation performance on **MAR** type missing values.

	MAE		RMSE	
Method	M-value	B-value	M-value	B-value
mean	0.025±0.001	0.025±0.001*	0.041±0.002	0.041±0.002*
softImpute	0.047±0.013	0.035±0.017*	0.088±0.035	0.074±0.053*
impute.knn	0.041±0.009*	0.059±0.013	0.088±0.021*	0.123±0.023
imputePCA	0.024±0.001	0.024±0.001*	0.040±0.002	**0.039±0.002***
SVDmiss	0.045±0.002	0.032±0.001*	0.078±0.005	0.060±0.005*
missForest	0.025±0.001	0.024±0.001*	0.041±0.002	0.040±0.002*
methyLImp	0.033±0.001	**0.024±0.001***	0.048±0.002	0.040±0.002*

Table 312: Dataset GSE88821 (D50). Imputation performance on **MNAR:low** type missing values.

	MAE		RMSE	
Method	M-value	B-value	M-value	B-value
mean	0.020±0.001	0.020±0.001*	0.037±0.002	0.037±0.002
softImpute	0.044±0.014	0.024±0.007*	0.097±0.041	0.051±0.026*
impute.knn	0.048±0.017	0.037±0.009*	0.105±0.030	0.094±0.022*
imputePCA	0.019±0.001	**0.019±0.001***	0.036±0.002	**0.035±0.002***
SVDmiss	0.039±0.002	0.026±0.001*	0.084±0.007	0.051±0.005*
missForest	0.020±0.001	0.020±0.001*	0.036±0.002	0.036±0.002
methyLImp	0.025±0.001	0.019±0.001*	0.041±0.002	0.037±0.003*

Table 313: Dataset GSE88821 (D50). Imputation performance on **MNAR:mid** type missing values.

	MAE		RMSE	
Method	M-value	B-value	M-value	B-value
mean	0.036±0.001	0.036±0.001*	0.054±0.002	0.052±0.001*
softImpute	0.045±0.004	0.045±0.019*	0.070±0.011	0.080±0.049
impute.knn	0.036±0.003*	0.048±0.007	0.060±0.009*	0.090±0.017
imputePCA	0.034±0.001	0.033±0.001*	0.052±0.002	**0.050±0.001***
SVDmiss	0.062±0.002	0.042±0.001*	0.093±0.004	0.075±0.004*
missForest	0.035±0.001	0.034±0.001*	0.053±0.002	0.051±0.001*
methyLImp	0.038±0.001	**0.033±0.001***	0.056±0.002	0.052±0.002*

Table 314: Dataset GSE88821 (D50). Imputation performance on **MNAR:high** type missing values.

	MAE		RMSE	
Method	M-value	B-value	M-value	B-value
mean	0.021±0.001*	0.022±0.001	0.037±0.002	0.035±0.001*
softImpute	0.042±0.011	0.044±0.031	0.073±0.028	0.100±0.086
impute.knn	0.080±0.028*	0.134±0.037	0.165±0.042*	0.217±0.037
imputePCA	0.021±0.001*	0.021±0.001	0.036±0.002	**0.034±0.001***
SVDmiss	0.040±0.001	0.029±0.001*	0.065±0.003	0.055±0.005*
missForest	0.021±0.001*	0.021±0.001	0.036±0.002	0.035±0.001*
methyLImp	0.033±0.001	**0.020±0.001***	0.047±0.002	0.036±0.002*

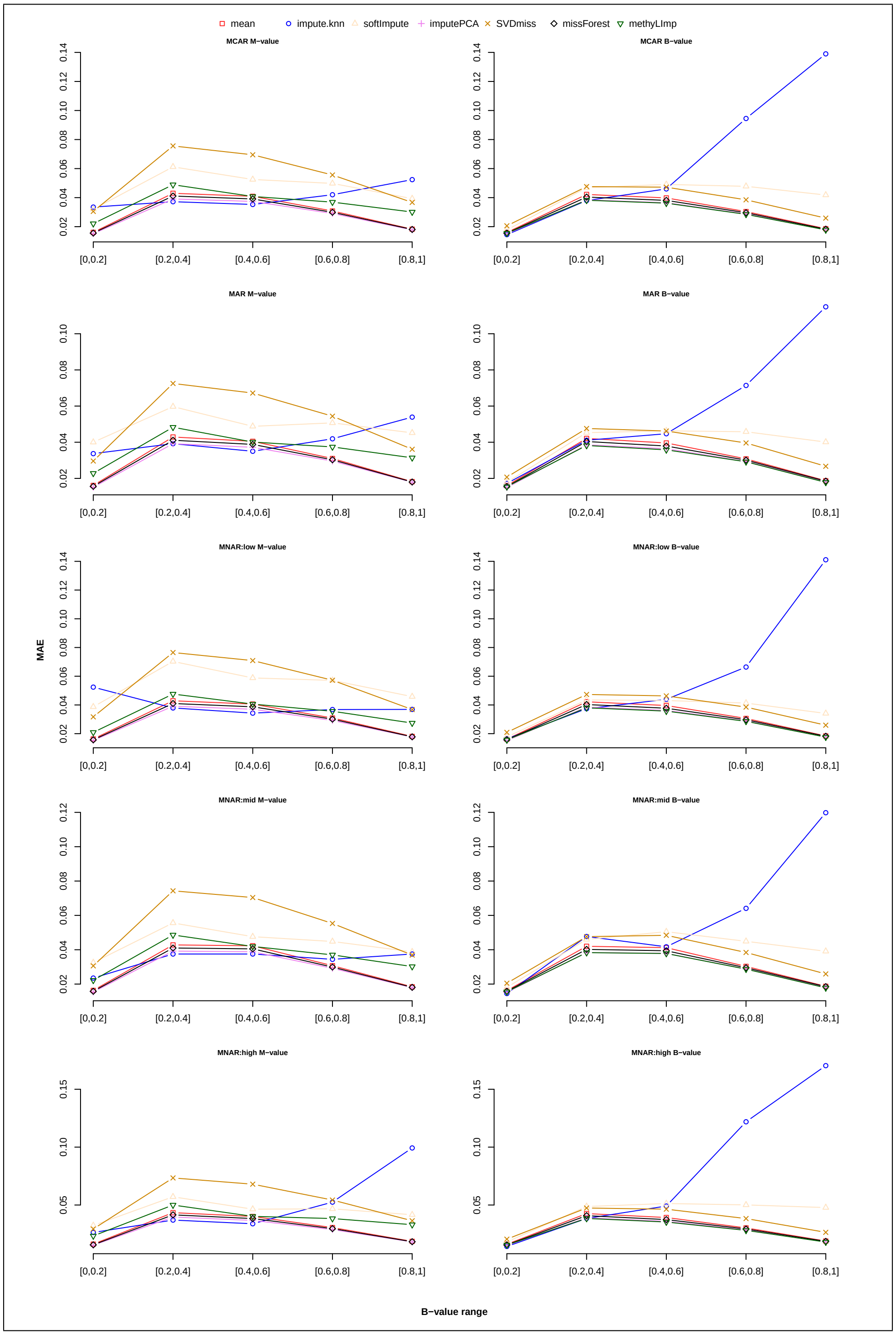

Figure 103: Dataset GSE88821 (D50). MAE imputation performances with respect to B-value range.

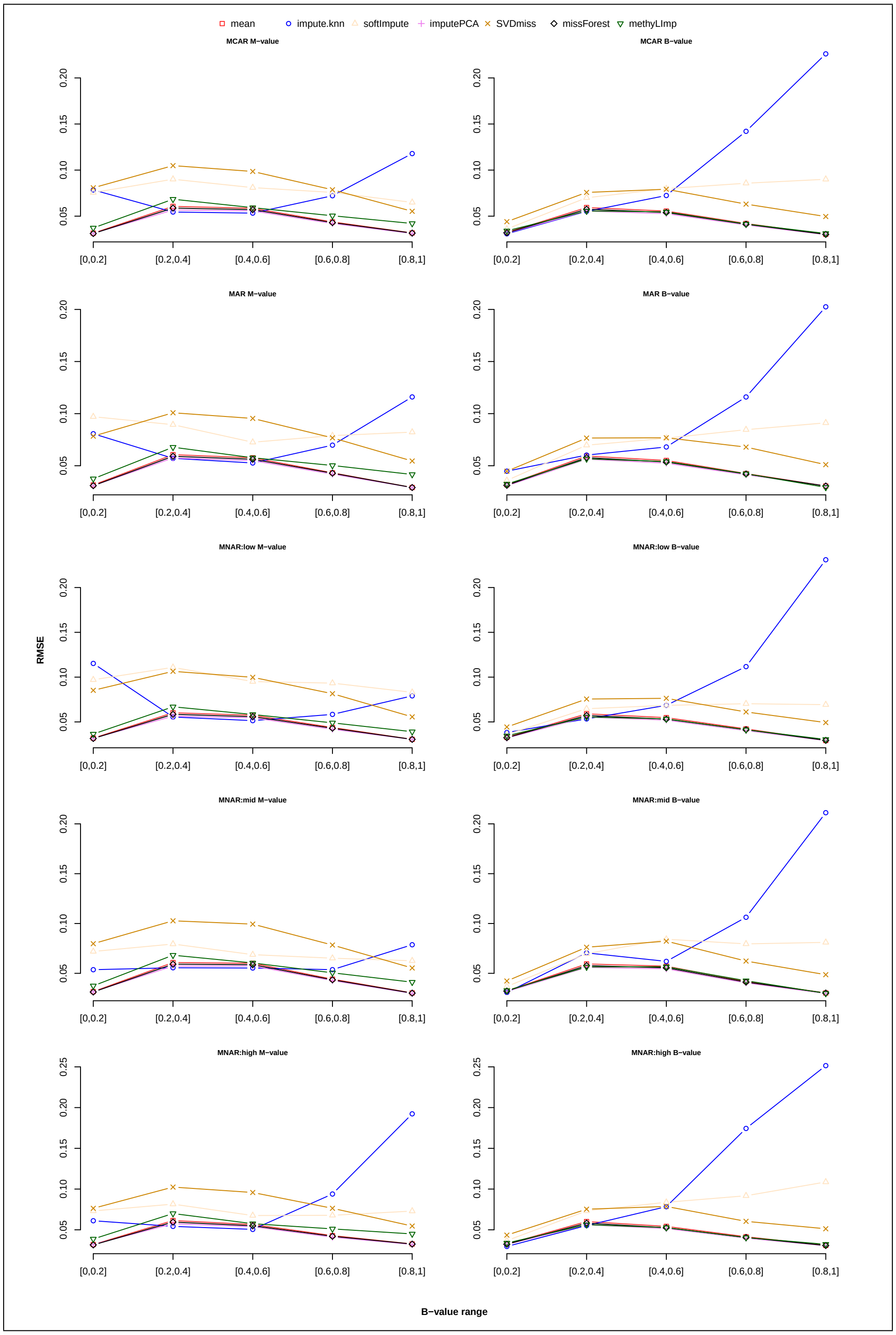

Figure 104: Dataset GSE88821 (D50). RMSE imputation performances with respect to B-value range.

2.51 GSE88821 (D51) - Liver - Cancer - 4 samples

Method	Avg time (sec)	Avg RAM (Mb)
mean	< 1	4
softImpute	< 1	42
imputePCA	6	142
impute.knn	< 1	42
SVDmiss	9	3317
methyLImp	1	109
missForest	167	249

Table 315: Dataset GSE88821 (D51). Average time and memory usage.

Table 316: Dataset GSE88821 (D51). Imputation performance on **MCAR** type missing values.

	MAE		RMSE	
Method	M-value	B-value	M-value	B-value
mean	0.059±0.002*	0.060±0.002	0.110±0.004*	0.110±0.004
softImpute	0.109±0.033	0.092±0.035*	0.182±0.052	0.201±0.080
impute.knn	0.127±0.018*	0.162±0.028	0.206±0.018*	0.260±0.024
imputePCA	0.048±0.002*	0.049±0.002	0.100±0.005*	0.100±0.005
SVDmiss	0.067±0.002	0.059±0.002*	0.113±0.004	0.108±0.004*
missForest	0.053±0.002*	0.054±0.002	0.104±0.005*	0.104±0.005
methyLImp	0.069±0.002	**0.045±0.002***	0.109±0.004	**0.095±0.005***

Table 317: Dataset GSE88821 (D51). Imputation performance on **MAR** type missing values.

	MAE		RMSE	
Method	M-value	B-value	M-value	B-value
mean	0.059±0.002*	0.059±0.002	0.109±0.004	0.109±0.004
softImpute	0.115±0.030	0.101±0.026*	0.193±0.047*	0.229±0.056
impute.knn	0.129±0.012*	0.152±0.022	0.211±0.014*	0.248±0.018
imputePCA	0.048±0.002*	0.048±0.002	0.097±0.004*	0.097±0.004
SVDmiss	0.067±0.002	0.058±0.002*	0.112±0.004	0.106±0.004*
missForest	0.053±0.002*	0.053±0.002	0.102±0.004	0.102±0.004
methyLImp	0.070±0.002	**0.044±0.002***	0.108±0.004	**0.093±0.004***

Table 318: Dataset GSE88821 (D51). Imputation performance on **MNAR:low** type missing values.

	MAE		RMSE	
Method	M-value	B-value	M-value	B-value
mean	0.047±0.002*	0.049±0.002	0.098±0.004*	0.101±0.004
softImpute	0.111±0.034	0.063±0.016*	0.201±0.053	0.153±0.045*
impute.knn	0.151±0.025	0.087±0.018*	0.229±0.019	0.180±0.020*
imputePCA	0.039±0.002*	0.040±0.002	0.090±0.005*	0.091±0.004
SVDmiss	0.057±0.002	0.048±0.002*	0.102±0.004	0.098±0.004*
missForest	0.042±0.002*	0.044±0.002	0.093±0.004*	0.095±0.004
methyLImp	0.061±0.002	**0.037±0.002***	0.101±0.004	**0.088±0.004***

Table 319: Dataset GSE88821 (D51). Imputation performance on **MNAR:mid** type missing values.

	MAE		RMSE	
Method	M-value	B-value	M-value	B-value
mean	0.101±0.002	0.096±0.002*	0.147±0.003	0.140±0.003*
softImpute	0.136±0.015	0.124±0.036*	0.189±0.023*	0.227±0.065
impute.knn	0.079±0.005*	0.125±0.014	0.132±0.007*	0.196±0.016
imputePCA	0.079±0.002	0.076±0.002*	0.127±0.003	0.125±0.003*
SVDmiss	0.103±0.002	0.093±0.002*	0.147±0.003	0.136±0.003*
missForest	0.089±0.002	0.085±0.002*	0.136±0.003	0.131±0.003*
methyLImp	0.093±0.002	**0.066±0.002***	0.129±0.003	**0.117±0.003***

Table 320: Dataset GSE88821 (D51). Imputation performance on **MNAR:high** type missing values.

	MAE		RMSE	
Method	M-value	B-value	M-value	B-value
mean	0.053±0.002*	0.056±0.002	0.102±0.004*	0.105±0.004
softImpute	0.116±0.038	0.115±0.043	0.190±0.055*	0.244±0.083
impute.knn	0.199±0.037*	0.306±0.040	0.289±0.038*	0.384±0.025
imputePCA	0.044±0.002*	0.045±0.002	0.092±0.005*	0.093±0.005
SVDmiss	0.062±0.002	0.055±0.002*	0.105±0.004	0.103±0.004*
missForest	0.048±0.002*	0.050±0.002	0.096±0.004*	0.097±0.004
methyLImp	0.070±0.002	**0.040±0.002***	0.108±0.004	**0.088±0.005***

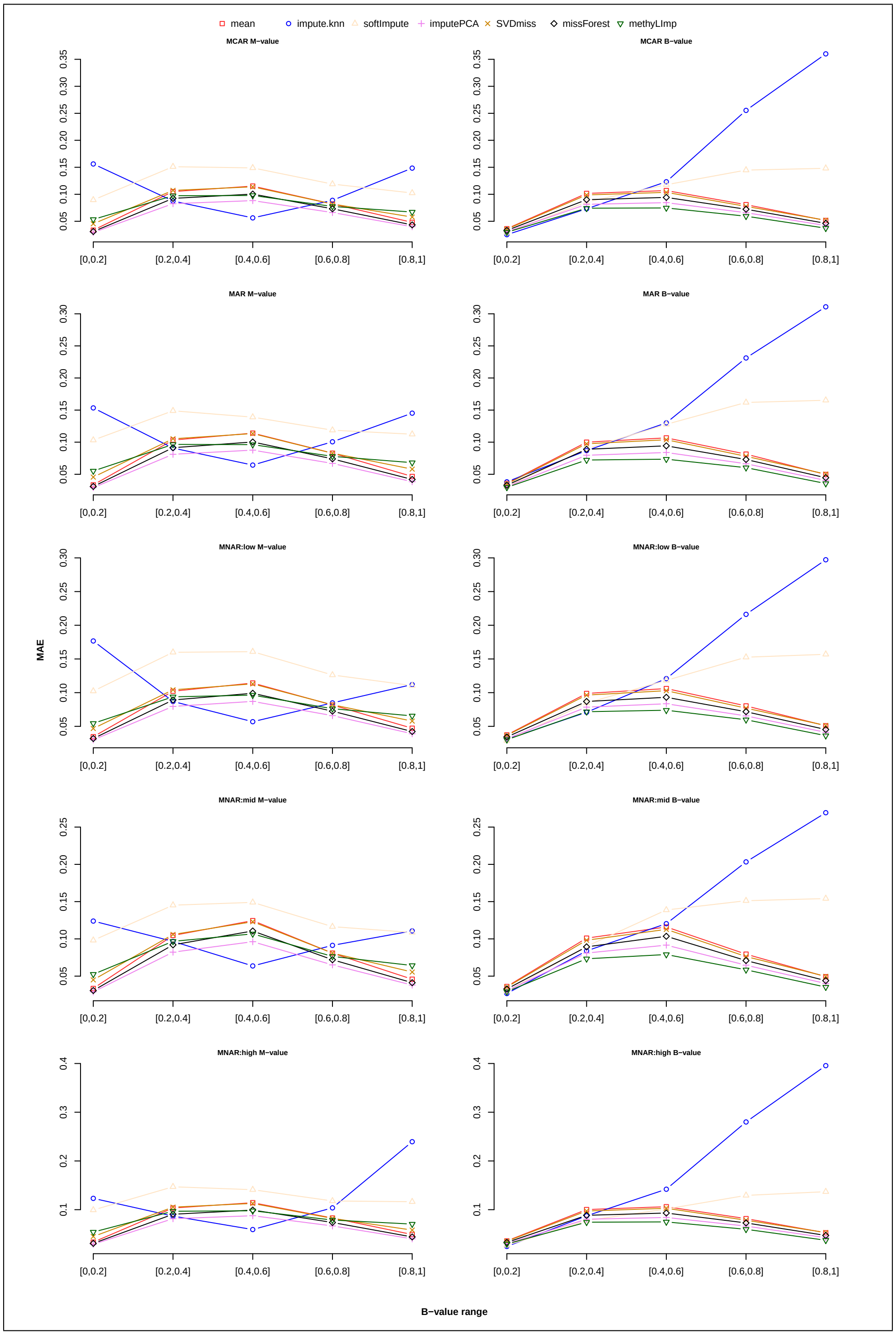

Figure 105: Dataset GSE88821 (D51). MAE imputation performances with respect to B-value range.

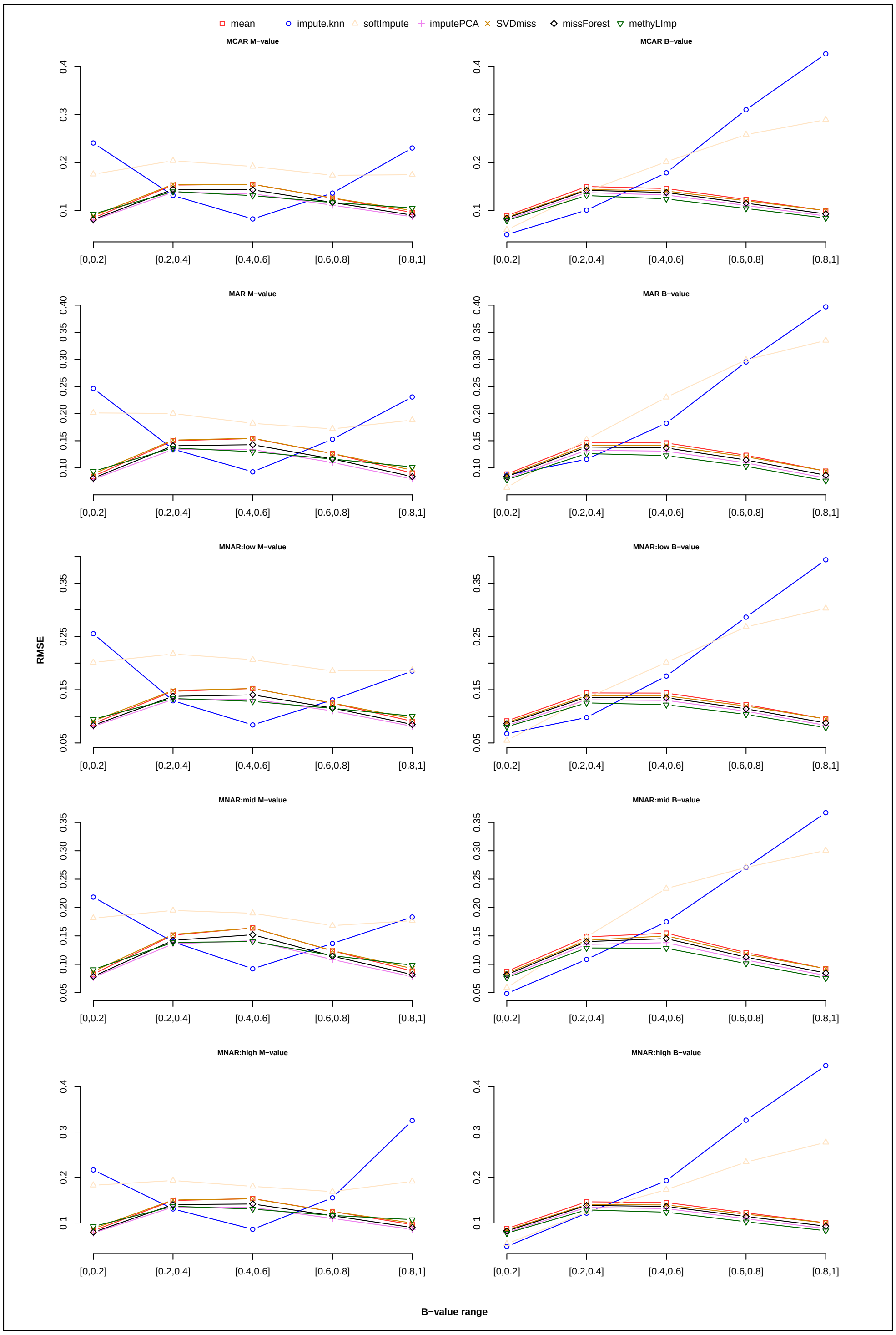

Figure 106: Dataset GSE88821 (D51). RMSE imputation performances with respect to B-value range.

2.52 GSE89093 (D52) - Blood - Normal - 46 samples

Method	Avg time (sec)	Avg RAM (Mb)
mean	< 1	40
softImpute	< 1	104
imputePCA	14	232
impute.knn	2	119
SVDmiss	144	3834
methyLImp	828	123
missForest	72316	270

Table 321: Dataset GSE89093 (D52). Average time and memory usage.

Table 322: Dataset GSE89093 (D52). Imputation performance on **MCAR** type missing values.

	MAE		RMSE	
Method	M-value	B-value	M-value	B-value
mean	0.025±0.001*	0.025±0.001	0.045±0.001	0.045±0.001*
softImpute	0.023±0.001	0.023±0.001	0.043±0.001	0.041±0.001*
impute.knn	0.021±0.001*	0.029±0.004	0.047±0.007*	0.071±0.012
imputePCA	0.022±0.001*	0.022±0.001	0.042±0.001	0.041±0.001*
SVDmiss	0.020±0.001*	0.020±0.001	0.039±0.001	**0.038±0.001***
missForest	0.020±0.001*	0.020±0.001	0.039±0.001	0.038±0.001*
methyLImp	**0.019±0.001***	0.020±0.001	0.039±0.001*	0.040±0.001

Table 323: Dataset GSE89093 (D52). Imputation performance on **MAR** type missing values.

	MAE		RMSE	
Method	M-value	B-value	M-value	B-value
mean	0.033±0.001*	0.033±0.001	0.065±0.001	0.064±0.001*
softImpute	0.031±0.001*	0.031±0.001	0.065±0.002	0.062±0.001*
impute.knn	0.029±0.001*	0.034±0.003	0.065±0.003*	0.079±0.010
imputePCA	0.030±0.001*	0.030±0.001	0.064±0.002	0.062±0.001*
SVDmiss	0.028±0.001*	0.029±0.001	0.063±0.002	0.061±0.001*
missForest	0.028±0.001*	0.028±0.001	0.063±0.001	**0.061±0.001***
methyLImp	**0.027±0.001***	0.027±0.001	0.063±0.002*	0.064±0.002

Table 324: Dataset GSE89093 (D52). Imputation performance on **MNAR:low** type missing values.

	MAE		RMSE	
Method	M-value	B-value	M-value	B-value
mean	0.019±0.001*	0.020±0.001	0.038±0.001*	0.039±0.001
softImpute	0.017±0.001*	0.018±0.001	0.037±0.001	0.036±0.001*
impute.knn	0.016±0.002*	0.021±0.003	0.042±0.010*	0.058±0.009
imputePCA	0.017±0.001*	0.017±0.001	0.036±0.001	0.036±0.001
SVDmiss	0.015±0.001*	0.016±0.001	0.034±0.001	0.034±0.001
missForest	0.015±0.001*	0.016±0.001	**0.034±0.001**	**0.034±0.001**
methyLImp	**0.015±0.001***	0.016±0.001	**0.034±0.001***	0.034±0.001

Table 325: Dataset GSE89093 (D52). Imputation performance on **MNAR:mid** type missing values.

	MAE		RMSE	
Method	M-value	B-value	M-value	B-value
mean	0.057±0.001	0.055±0.001*	0.086±0.001	0.082±0.001*
softImpute	0.055±0.001	0.049±0.001*	0.083±0.001	0.075±0.001*
impute.knn	0.044±0.001*	0.047±0.002	0.073±0.002*	0.081±0.004
imputePCA	0.051±0.001	0.049±0.001*	0.080±0.001	0.075±0.001*
SVDmiss	0.046±0.001	**0.043±0.001***	0.075±0.001	**0.070±0.001***
missForest	0.046±0.001	0.045±0.001*	0.075±0.001	0.070±0.001*
methyLImp	0.044±0.001	**0.043±0.001***	0.073±0.001	0.073±0.001*

Table 326: Dataset GSE89093 (D52). Imputation performance on **MNAR:high** type missing values.

	MAE		RMSE	
Method	M-value	B-value	M-value	B-value
mean	0.022±0.001*	0.022±0.001	0.039±0.001*	0.039±0.001
softImpute	0.020±0.001*	0.021±0.001	0.037±0.001	0.036±0.001*
impute.knn	0.019±0.002*	0.028±0.006	0.044±0.008*	0.072±0.017
imputePCA	0.019±0.001*	0.019±0.001	0.036±0.001	0.036±0.001*
SVDmiss	0.017±0.001*	0.018±0.001	0.034±0.001*	0.034±0.001
missForest	0.017±0.001*	0.018±0.001	**0.034±0.001***	0.034±0.001
methyLImp	**0.017±0.001***	0.017±0.001	**0.034±0.001***	0.034±0.001

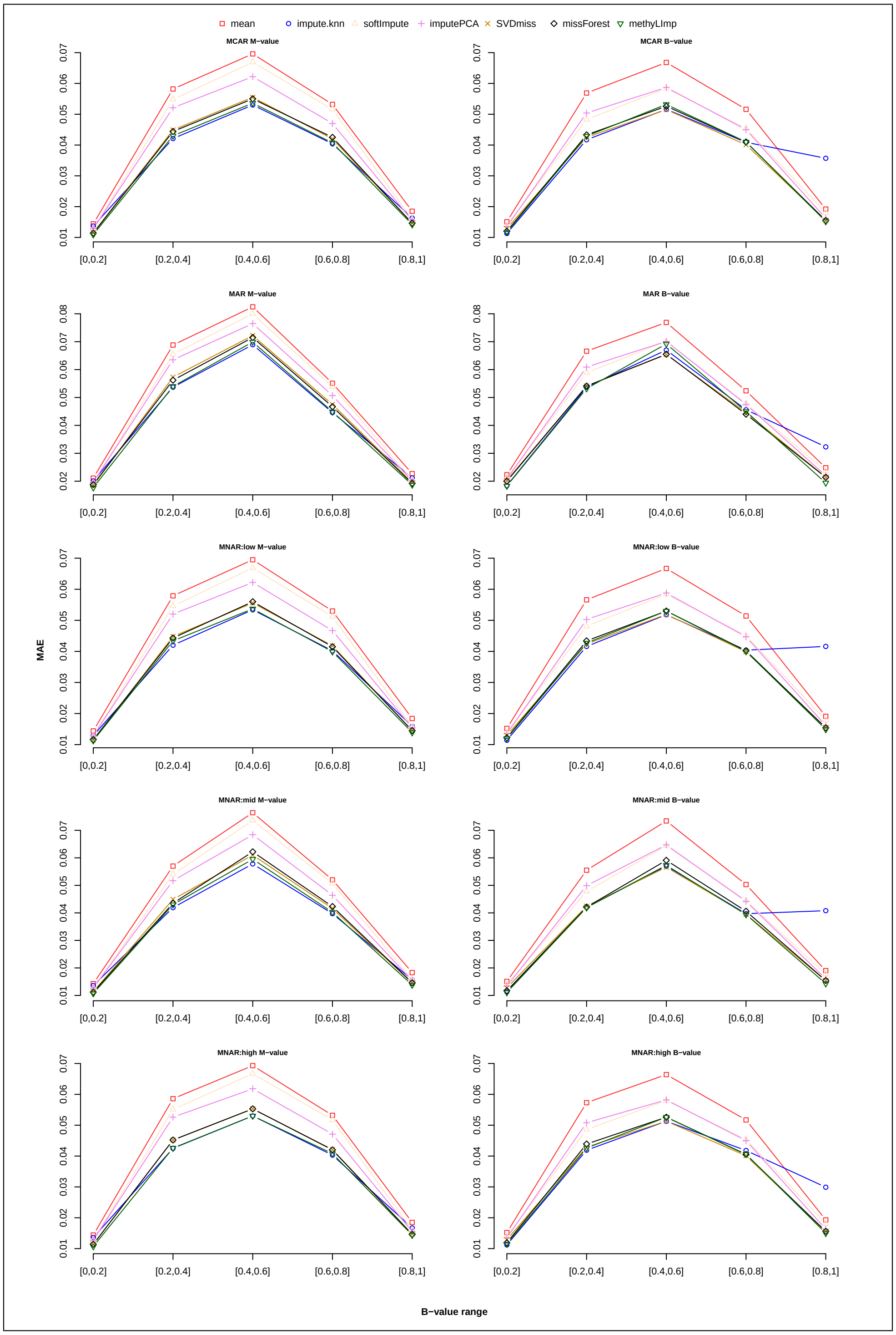

Figure 107: Dataset GSE89093 (D52). MAE imputation performances with respect to B-value range.

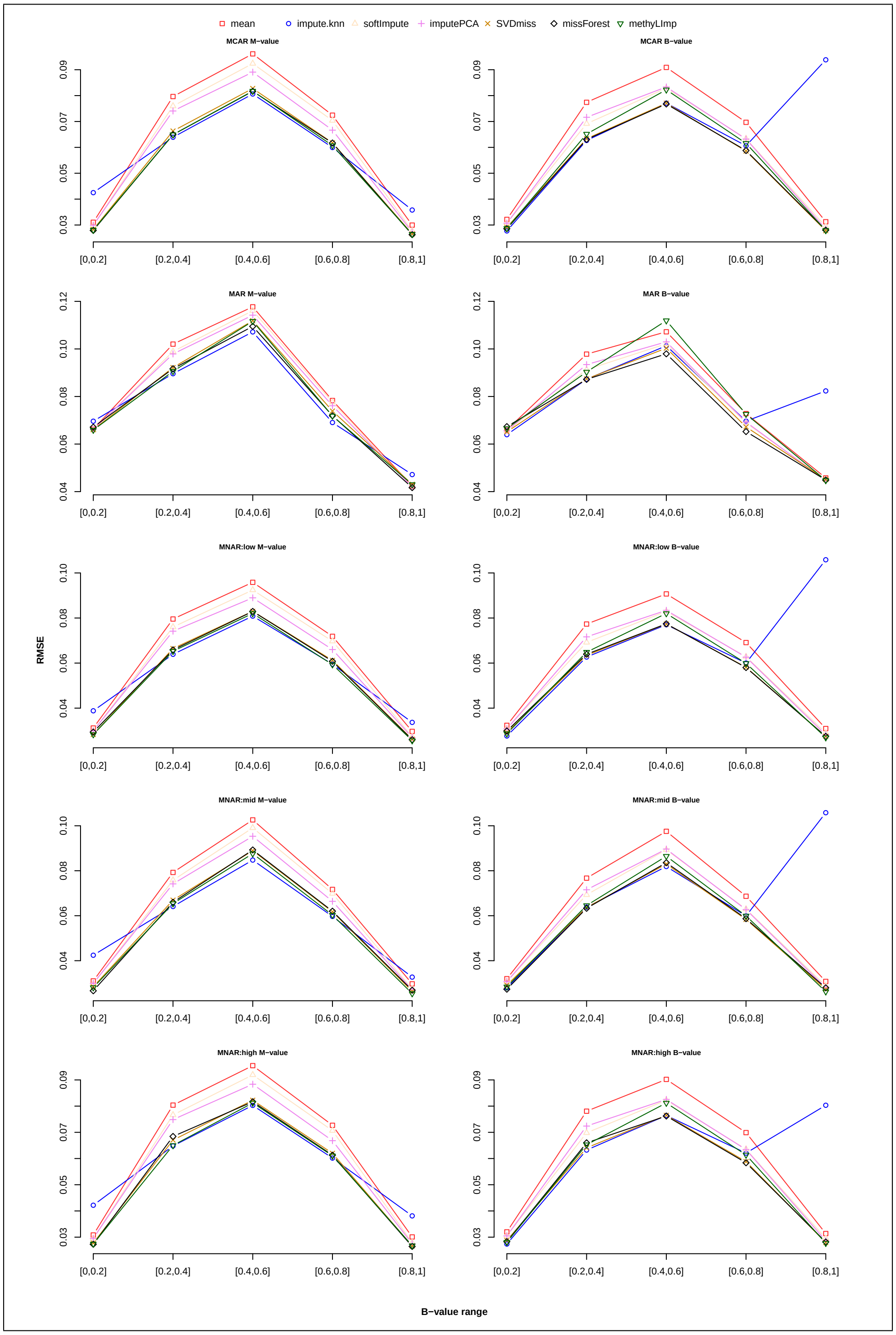

Figure 108: Dataset GSE89093 (D52). RMSE imputation performances with respect to B-value range.

2.53 GSE89472 (D53) - Blood - Normal - 5 samples

Method	Avg time (sec)	Avg RAM (Mb)
mean	< 1	4
softImpute	< 1	41
imputePCA	4	142
impute.knn	< 1	42
SVDmiss	10	3709
methyLImp	2	114
missForest	145	269

Table 327: Dataset GSE89472 (D53). Average time and memory usage.

Table 328: Dataset GSE89472 (D53). Imputation performance on **MCAR** type missing values.

	MAE		RMSE	
Method	M-value	B-value	M-value	B-value
mean	0.023±0.001	0.023±0.001*	0.046±0.003	0.045±0.003*
softImpute	0.025±0.001*	0.064±0.051	0.047±0.005*	0.145±0.126
impute.knn	0.091±0.029*	0.157±0.044	0.169±0.038*	0.244±0.047
imputePCA	0.023±0.001	0.023±0.001*	0.046±0.003	0.044±0.003*
SVDmiss	0.052±0.004	0.036±0.003*	0.085±0.005	0.069±0.007*
missForest	0.023±0.001	0.023±0.001*	0.046±0.003	0.045±0.003*
methyLImp	**0.022±0.001***	0.022±0.001	0.042±0.003	**0.042±0.003***

Table 329: Dataset GSE89472 (D53). Imputation performance on **MAR** type missing values.

	MAE		RMSE	
Method	M-value	B-value	M-value	B-value
mean	0.043±0.001	0.042±0.001*	0.089±0.004	0.084±0.004*
softImpute	0.044±0.011*	0.095±0.056	0.093±0.023*	0.205±0.118
impute.knn	0.089±0.017*	0.160±0.038	0.161±0.022*	0.242±0.038
imputePCA	0.043±0.001	0.041±0.001*	0.087±0.004	0.082±0.003*
SVDmiss	0.073±0.004	0.052±0.003*	0.119±0.005	0.107±0.008*
missForest	0.043±0.001	0.042±0.001*	0.088±0.004	0.083±0.004*
methyLImp	0.040±0.001	**0.038±0.001***	0.080±0.004	**0.076±0.004***

Table 330: Dataset GSE89472 (D53). Imputation performance on **MNAR:low** type missing values.

	MAE		RMSE	
Method	M-value	B-value	M-value	B-value
mean	0.019±0.001*	0.019±0.001	0.043±0.003*	0.044±0.003
softImpute	0.023±0.017	0.030±0.022	0.050±0.037	0.072±0.069
impute.knn	0.117±0.043	0.080±0.022*	0.213±0.051	0.172±0.034*
imputePCA	0.019±0.001*	0.019±0.001	0.042±0.003*	0.044±0.003
SVDmiss	0.035±0.001	0.030±0.002*	0.065±0.004	0.062±0.007*
missForest	0.019±0.001*	0.019±0.001	0.043±0.003*	0.044±0.003
methyLImp	**0.018±0.001***	0.018±0.001	0.038±0.003	**0.037±0.003***

Table 331: Dataset GSE89472 (D53). Imputation performance on **MNAR:mid** type missing values.

	MAE		RMSE	
Method	M-value	B-value	M-value	B-value
mean	0.055±0.001	0.051±0.001*	0.095±0.002	0.084±0.002*
softImpute	0.052±0.003*	0.087±0.035	**0.081±0.007***	0.158±0.069
impute.knn	**0.050±0.005***	0.088±0.018	**0.082±0.014***	0.157±0.031
imputePCA	0.054±0.001	0.050±0.001*	0.095±0.003	0.083±0.002*
SVDmiss	0.070±0.001	0.062±0.003*	0.110±0.003	0.107±0.007*
missForest	0.054±0.001	0.051±0.001*	0.096±0.003	0.084±0.002*
methyLImp	0.052±0.001	0.050±0.001*	0.088±0.003	0.082±0.002*

Table 332: Dataset GSE89472 (D53). Imputation performance on **MNAR:high** type missing values.

	MAE		RMSE	
Method	M-value	B-value	M-value	B-value
mean	0.020±0.001	0.020±0.001	0.039±0.003	0.038±0.003*
softImpute	0.023±0.002*	0.084±0.067	0.045±0.009*	0.185±0.153
impute.knn	0.144±0.037*	0.240±0.050	0.227±0.035*	0.310±0.040
imputePCA	0.020±0.001	0.020±0.001	0.039±0.003	0.038±0.003*
SVDmiss	0.100±0.010	0.033±0.003*	0.153±0.017	0.065±0.009*
missForest	0.020±0.001	0.020±0.001	0.039±0.003	0.038±0.003*
methyLImp	**0.020±0.001***	0.020±0.001	**0.037±0.003**	**0.037±0.003**

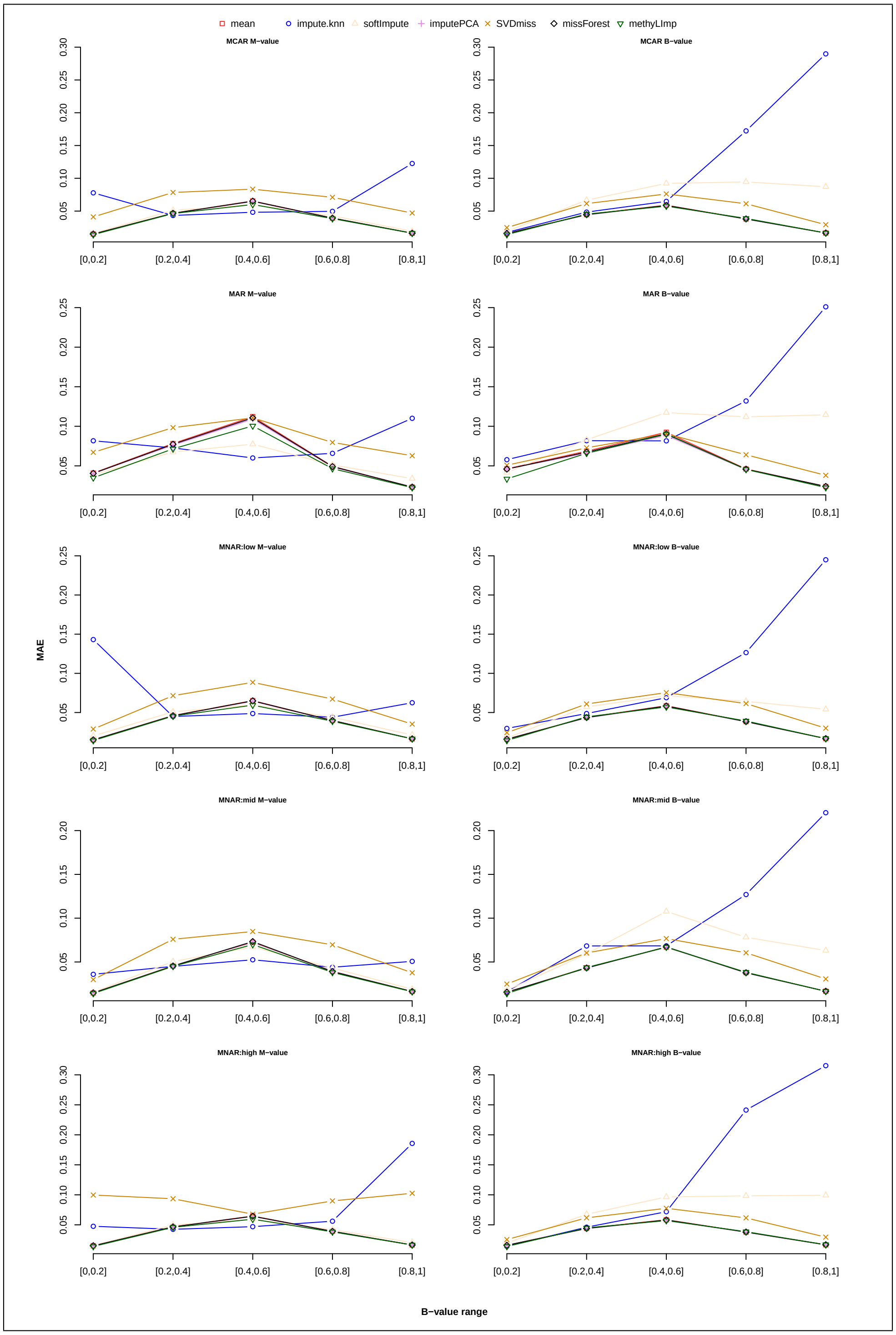

Figure 109: Dataset GSE89472 (D53). MAE imputation performances with respect to B-value range.

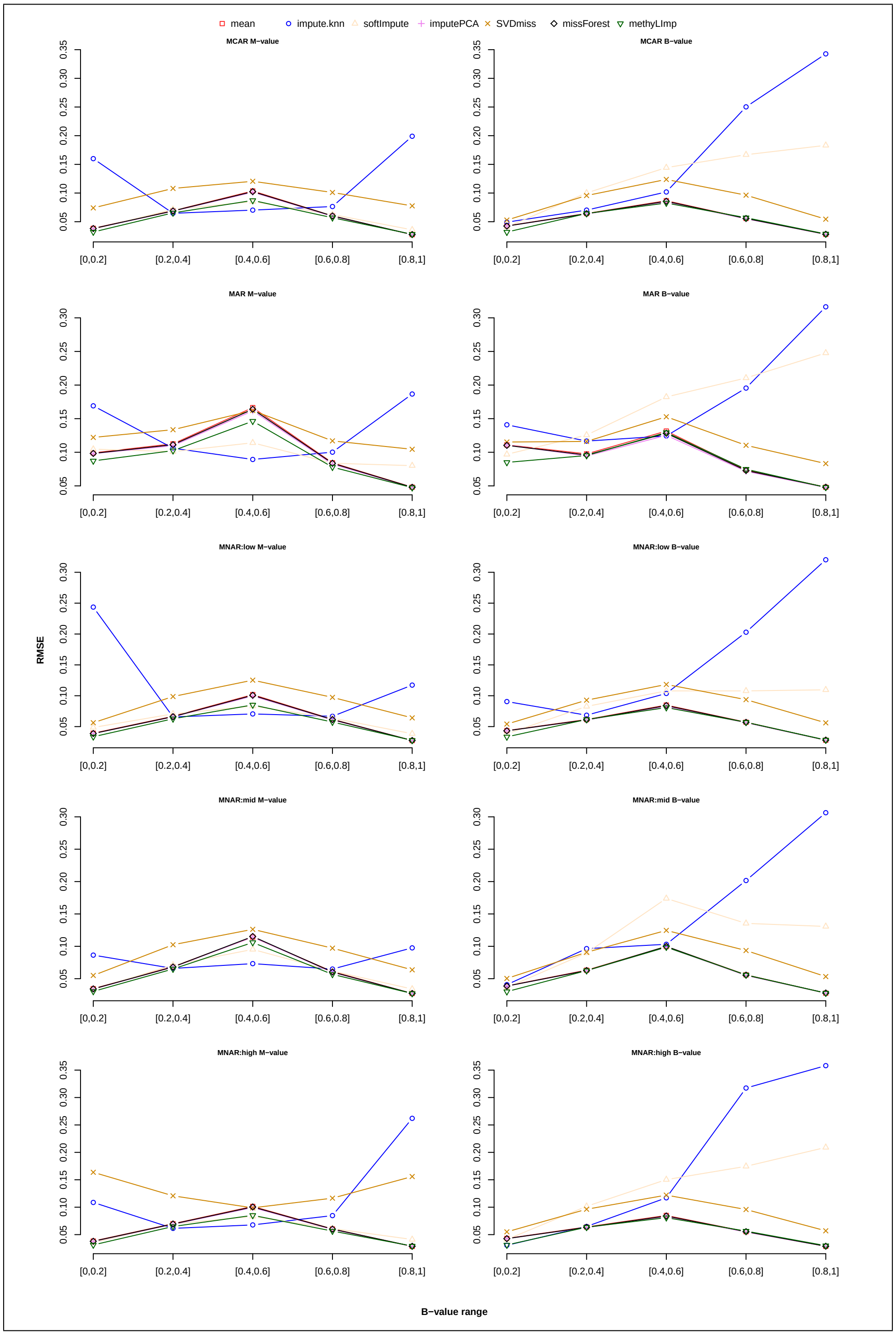

Figure 110: Dataset GSE89472 (D53). RMSE imputation performances with respect to B-value range.

2.54 GSE89702 (D54) - Cerebellum - Normal - 17 samples

Method	Avg time (sec)	Avg RAM (Mb)
mean	< 1	15
softImpute	< 1	48
imputePCA	10	170
impute.knn	< 1	54
SVDmiss	32	3232
methyLImp	31	88
missForest	5695	122

Table 333: Dataset GSE89702 (D54). Average time and memory usage.

Table 334: Dataset GSE89702 (D54). Imputation performance on **MCAR** type missing values.

	MAE		RMSE	
Method	M-value	B-value	M-value	B-value
mean	0.024±0.001*	0.024±0.001	0.038±0.001	0.038±0.001*
softImpute	0.024±0.001*	0.025±0.003	0.039±0.001	0.042±0.012
impute.knn	0.036±0.007*	0.045±0.010	0.078±0.018*	0.099±0.019
imputePCA	0.023±0.001*	0.023±0.001	0.037±0.001	0.036±0.001*
SVDmiss	0.026±0.001	0.026±0.001*	0.046±0.001	0.045±0.001*
missForest	0.023±0.001*	0.023±0.001	0.037±0.001	0.036±0.001*
methyLImp	**0.022±0.001***	0.022±0.001	**0.035±0.001***	0.035±0.001

Table 335: Dataset GSE89702 (D54). Imputation performance on **MAR** type missing values.

	MAE		RMSE	
Method	M-value	B-value	M-value	B-value
mean	0.026±0.001*	0.026±0.001	0.042±0.001	0.042±0.001
softImpute	0.026±0.001*	0.027±0.003	0.043±0.002*	0.047±0.011
impute.knn	0.031±0.004*	0.036±0.006	0.057±0.013*	0.073±0.018
imputePCA	0.025±0.001*	0.025±0.001	0.041±0.001	0.041±0.001
SVDmiss	0.029±0.001	0.028±0.001*	0.051±0.002	0.051±0.003*
missForest	0.025±0.001*	0.025±0.001	0.041±0.001	0.040±0.002
methyLImp	**0.024±0.001***	0.024±0.001	**0.039±0.001***	0.039±0.001

Table 336: Dataset GSE89702 (D54). Imputation performance on **MNAR:low** type missing values.

	MAE		RMSE	
Method	M-value	B-value	M-value	B-value
mean	0.019±0.001*	0.019±0.001	0.031±0.001*	0.031±0.001
softImpute	0.018±0.001*	0.019±0.001	0.032±0.001	0.032±0.004
impute.knn	0.036±0.010	0.026±0.005*	0.085±0.023	0.063±0.015*
imputePCA	0.017±0.001*	0.018±0.001	0.029±0.001*	0.030±0.001
SVDmiss	0.020±0.001	0.020±0.001*	0.037±0.001	0.037±0.002*
missForest	0.018±0.001*	0.018±0.001	0.029±0.001*	0.029±0.001
methyLImp	**0.017±0.001***	0.017±0.001	**0.028±0.001***	0.028±0.001

Table 337: Dataset GSE89702 (D54). Imputation performance on **MNAR:mid** type missing values.

	MAE		RMSE	
Method	M-value	B-value	M-value	B-value
mean	0.044±0.001	0.043±0.001*	0.065±0.001	0.063±0.001*
softImpute	0.043±0.001*	0.045±0.002	0.065±0.001*	0.069±0.006
impute.knn	0.044±0.002*	0.045±0.003	0.067±0.005*	0.072±0.007
imputePCA	0.042±0.001	0.042±0.001*	0.063±0.001	0.062±0.001*
SVDmiss	0.049±0.001	0.047±0.001*	0.077±0.001	0.077±0.001*
missForest	0.042±0.001	0.042±0.001*	0.063±0.001	0.062±0.001*
methyLImp	0.041±0.001	**0.040±0.001***	**0.061±0.001***	0.061±0.001

Table 338: Dataset GSE89702 (D54). Imputation performance on **MNAR:high** type missing values.

	MAE		RMSE	
Method	M-value	B-value	M-value	B-value
mean	0.022±0.001*	0.022±0.001	0.033±0.001*	0.033±0.001
softImpute	0.021±0.001*	0.023±0.004	0.034±0.001*	0.038±0.015
impute.knn	0.039±0.010*	0.073±0.020	0.089±0.025*	0.148±0.030
imputePCA	0.020±0.001*	0.021±0.001	0.031±0.001*	0.032±0.001
SVDmiss	0.024±0.001	0.023±0.001*	0.040±0.001	0.040±0.002*
missForest	0.021±0.001*	0.021±0.001	0.032±0.001*	0.032±0.001
methyLImp	**0.020±0.001***	0.020±0.001	**0.030±0.001***	0.031±0.001

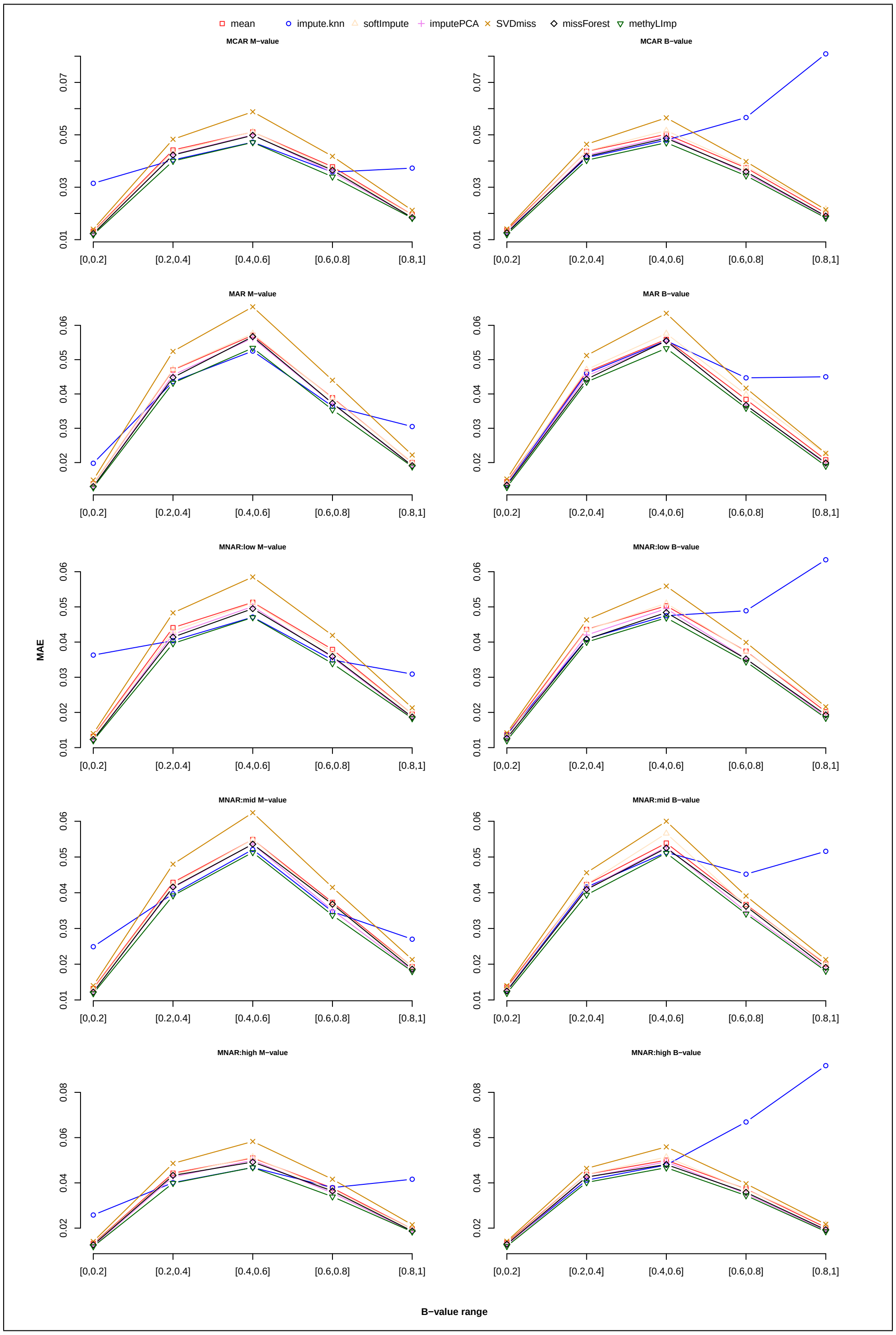

Figure 111: Dataset GSE89702 (D54). MAE imputation performances with respect to B-value range.

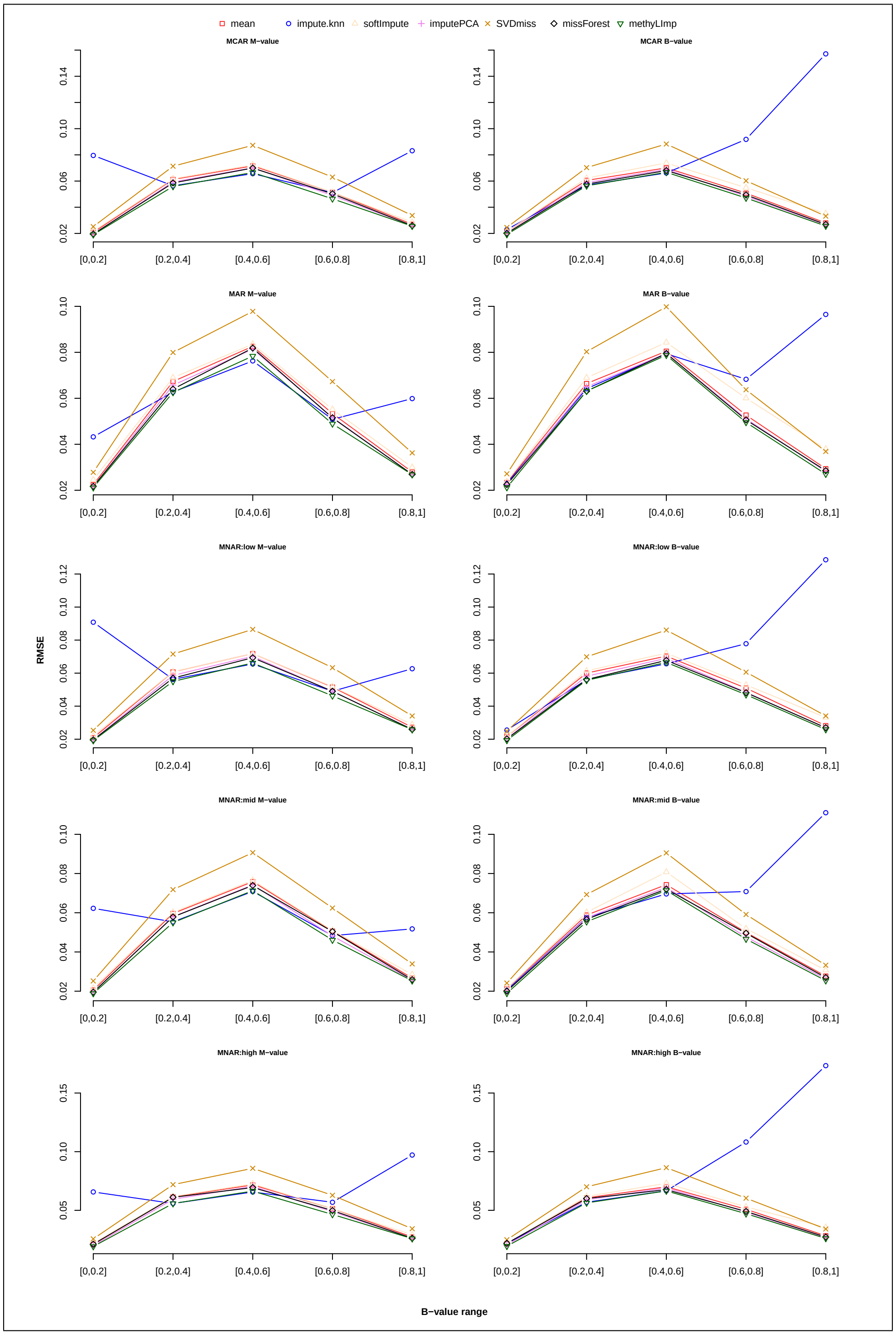

Figure 112: Dataset GSE89702 (D54). RMSE imputation performances with respect to B-value range.

2.55 GSE89703 (D55) - Hippocampus - Normal - 13 samples

Method	Avg time (sec)	Avg RAM (Mb)
mean	< 1	12
softImpute	< 1	42
imputePCA	6	154
impute.knn	< 1	49
SVDmiss	19	3099
methyLImp	13	98
missForest	4573	111

Table 339: Dataset GSE89703 (D55). Average time and memory usage.

Table 340: Dataset GSE89703 (D55). Imputation performance on **MCAR** type missing values.

	MAE		RMSE	
Method	M-value	B-value	M-value	B-value
mean	0.029±0.001*	0.029±0.001	0.042±0.001	0.042±0.001*
softImpute	0.027±0.001*	0.027±0.001	0.040±0.001	0.040±0.001*
impute.knn	0.040±0.009*	0.054±0.012	0.078±0.021*	0.105±0.022
imputePCA	0.026±0.001*	0.026±0.001	0.039±0.001*	0.039±0.001
SVDmiss	0.031±0.001	0.030±0.001*	0.050±0.001	0.049±0.001*
missForest	0.027±0.001*	0.027±0.001	0.039±0.001	0.039±0.001*
methyLImp	**0.025±0.001***	0.025±0.001	**0.037±0.001***	0.037±0.001

Table 341: Dataset GSE89703 (D55). Imputation performance on **MAR** type missing values.

	MAE		RMSE	
Method	M-value	B-value	M-value	B-value
mean	0.030±0.001*	0.030±0.001	0.044±0.001	0.044±0.001
softImpute	0.029±0.001*	0.029±0.001	0.043±0.001*	0.044±0.002
impute.knn	0.034±0.004*	0.042±0.007	0.057±0.012*	0.078±0.017
imputePCA	0.028±0.001*	0.028±0.001	0.041±0.001*	0.042±0.001
SVDmiss	0.033±0.001	0.033±0.001*	0.054±0.002	0.053±0.002*
missForest	0.028±0.001*	0.029±0.001	0.042±0.001	0.042±0.001*
methyLImp	**0.027±0.001***	0.027±0.001	**0.040±0.001***	0.041±0.001

Table 342: Dataset GSE89703 (D55). Imputation performance on **MNAR:low** type missing values.

	MAE		RMSE	
Method	M-value	B-value	M-value	B-value
mean	0.022±0.001*	0.022±0.001	0.033±0.001*	0.034±0.001
softImpute	0.020±0.001*	0.021±0.003	0.032±0.001*	0.033±0.011
impute.knn	0.044±0.015	0.028±0.005*	0.097±0.032	0.060±0.015*
imputePCA	0.020±0.001*	0.020±0.001	0.031±0.001*	0.031±0.001
SVDmiss	0.023±0.001*	0.023±0.001	0.040±0.001	0.039±0.001*
missForest	0.020±0.001*	0.020±0.001	0.031±0.001*	0.032±0.001
methyLImp	**0.019±0.001***	0.019±0.001	**0.030±0.001***	0.030±0.001

Table 343: Dataset GSE89703 (D55). Imputation performance on **MNAR:mid** type missing values.

	MAE		RMSE	
Method	M-value	B-value	M-value	B-value
mean	0.046±0.001	0.045±0.001*	0.064±0.001	0.063±0.001*
softImpute	0.042±0.001	0.042±0.001	0.059±0.001	0.059±0.001
impute.knn	0.043±0.002*	0.048±0.003	0.063±0.006*	0.076±0.009
imputePCA	0.042±0.001	0.041±0.001*	0.058±0.001	0.058±0.001*
SVDmiss	0.048±0.001	0.047±0.001*	0.070±0.001*	0.071±0.001
missForest	0.043±0.001	0.042±0.001*	0.060±0.001	0.059±0.001*
methyLImp	0.040±0.001	**0.040±0.001***	0.057±0.001	**0.056±0.001***

Table 344: Dataset GSE89703 (D55). Imputation performance on **MNAR:high** type missing values.

	MAE		RMSE	
Method	M-value	B-value	M-value	B-value
mean	0.025±0.001*	0.026±0.001	0.036±0.001*	0.036±0.001
softImpute	0.024±0.001*	0.026±0.010	0.035±0.001*	0.042±0.033
impute.knn	0.050±0.015*	0.088±0.023	0.104±0.030*	0.159±0.030
imputePCA	0.023±0.001*	0.023±0.001	0.033±0.001*	0.034±0.001
SVDmiss	0.027±0.001	0.027±0.001*	0.044±0.001	0.043±0.001*
missForest	0.023±0.001*	0.024±0.001	0.034±0.001*	0.034±0.001
methyLImp	**0.023±0.001***	0.023±0.001	**0.032±0.001***	0.033±0.001

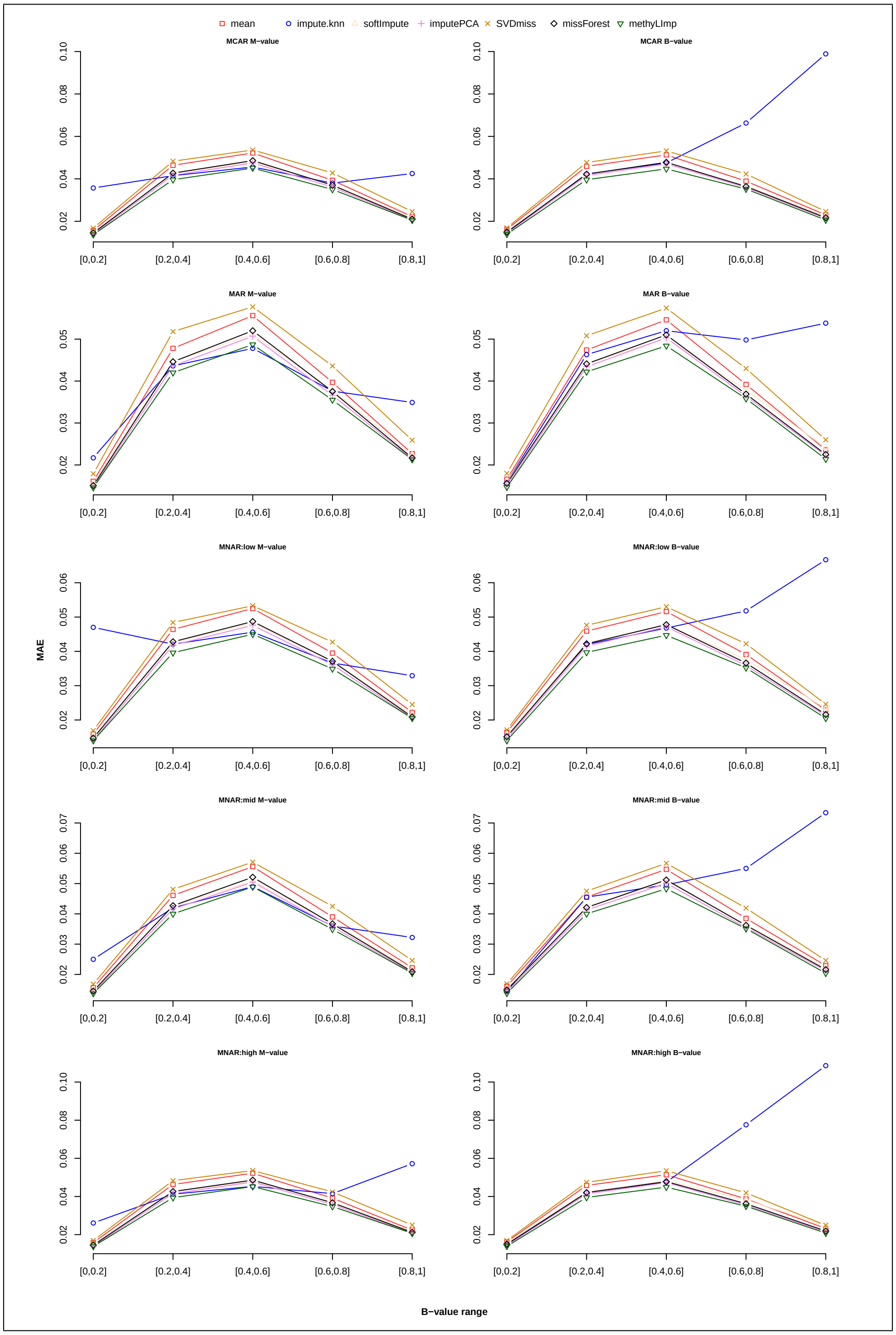

Figure 113: Dataset GSE89703 (D55). MAE imputation performances with respect to B-value range.

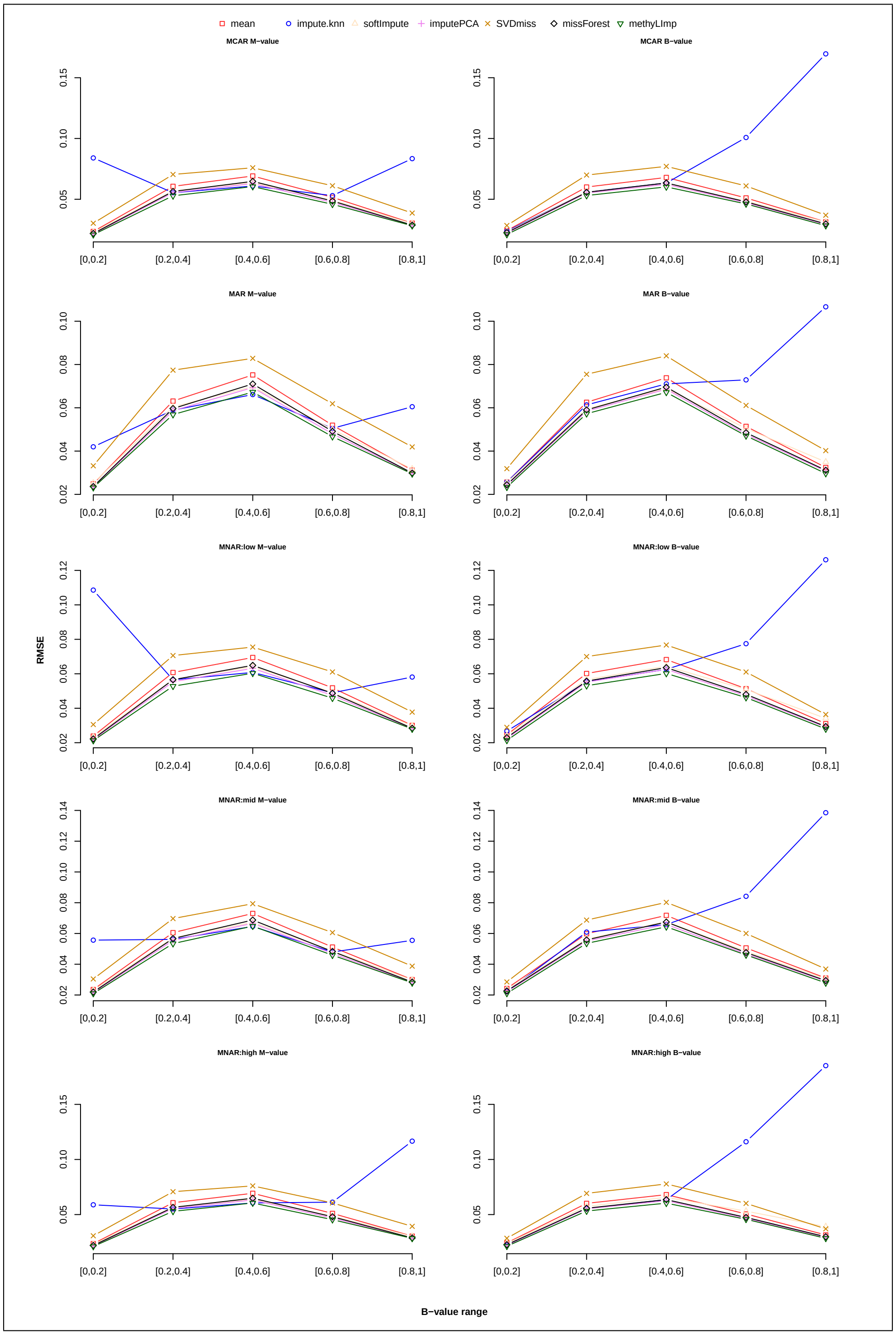

Figure 114: Dataset GSE89703 (D55). RMSE imputation performances with respect to B-value range.

2.56 GSE89705 (D56) - Putamen - Normal - 17 samples

Method	Avg time (sec)	Avg RAM (Mb)
mean	< 1	15
softImpute	< 1	49
imputePCA	9	168
impute.knn	< 1	53
SVDmiss	33	3238
methyLImp	32	89
missForest	8256	121

Table 345: Dataset GSE89705 (D56). Average time and memory usage.

Table 346: Dataset GSE89705 (D56). Imputation performance on **MCAR** type missing values.

	MAE		RMSE	
Method	M-value	B-value	M-value	B-value
mean	0.028±0.001*	0.028±0.001	0.043±0.001	0.043±0.001
softImpute	0.026±0.001*	0.026±0.001	0.041±0.001	0.041±0.001*
impute.knn	0.035±0.006*	0.046±0.009	0.069±0.017*	0.093±0.019
imputePCA	0.025±0.001*	0.026±0.001	0.039±0.001*	0.039±0.001
SVDmiss	0.032±0.001	0.031±0.001*	0.055±0.001	0.054±0.001*
missForest	0.026±0.001*	0.026±0.001	0.040±0.001	0.039±0.001*
methyLImp	**0.025±0.001***	0.025±0.001	**0.038±0.001***	0.038±0.001

Table 347: Dataset GSE89705 (D56). Imputation performance on **MAR** type missing values.

	MAE		RMSE	
Method	M-value	B-value	M-value	B-value
mean	0.029±0.001*	0.030±0.001	0.045±0.001	0.045±0.001
softImpute	0.028±0.001*	0.029±0.001	0.044±0.001*	0.045±0.002
impute.knn	0.032±0.003*	0.039±0.007	0.054±0.011*	0.072±0.018
imputePCA	0.027±0.001*	0.027±0.001	0.042±0.001*	0.042±0.001
SVDmiss	0.034±0.001	0.034±0.001*	0.060±0.002	0.058±0.003*
missForest	0.027±0.001*	0.028±0.001	0.042±0.002	0.042±0.002
methyLImp	**0.026±0.001***	0.026±0.001	**0.041±0.001***	0.041±0.001

Table 348: Dataset GSE89705 (D56). Imputation performance on **MNAR:low** type missing values.

	MAE		RMSE	
Method	M-value	B-value	M-value	B-value
mean	0.021±0.001*	0.022±0.001	0.034±0.001*	0.034±0.001
softImpute	0.020±0.001*	0.020±0.001	0.033±0.001	0.033±0.001
impute.knn	0.035±0.011	0.027±0.003*	0.082±0.026	0.059±0.011*
imputePCA	0.019±0.001*	0.019±0.001	0.031±0.001*	0.032±0.001
SVDmiss	0.024±0.001	0.024±0.001*	0.045±0.001	0.043±0.001*
missForest	0.019±0.001*	0.019±0.001	0.032±0.001*	0.032±0.001
methyLImp	**0.018±0.001***	0.018±0.001	**0.030±0.001***	0.031±0.001

Table 349: Dataset GSE89705 (D56). Imputation performance on **MNAR:mid** type missing values.

	MAE		RMSE	
Method	M-value	B-value	M-value	B-value
mean	0.044±0.001	0.044±0.001*	0.064±0.001	0.063±0.001*
softImpute	0.042±0.001*	0.042±0.003	0.062±0.001*	0.063±0.011
impute.knn	0.042±0.002*	0.044±0.003	0.063±0.005*	0.070±0.008
imputePCA	0.040±0.001	0.040±0.001*	0.059±0.001	0.059±0.001*
SVDmiss	0.050±0.001	0.050±0.001*	0.080±0.001*	0.081±0.001
missForest	0.041±0.001	0.040±0.001*	0.061±0.001	0.059±0.001*
methyLImp	**0.039±0.001***	0.039±0.001	**0.058±0.001***	0.058±0.001

Table 350: Dataset GSE89705 (D56). Imputation performance on **MNAR:high** type missing values.

	MAE		RMSE	
Method	M-value	B-value	M-value	B-value
mean	0.024±0.001*	0.025±0.001	0.036±0.001*	0.037±0.001
softImpute	0.023±0.001*	0.024±0.004	0.036±0.001*	0.038±0.018
impute.knn	0.038±0.009*	0.069±0.016	0.079±0.022*	0.136±0.023
imputePCA	0.022±0.001*	0.023±0.001	0.034±0.001*	0.034±0.001
SVDmiss	0.028±0.001	0.028±0.001*	0.049±0.001	0.047±0.001*
missForest	0.022±0.001*	0.023±0.001	0.034±0.001*	0.034±0.001
methyLImp	**0.022±0.001***	0.022±0.001	**0.033±0.001***	0.033±0.001

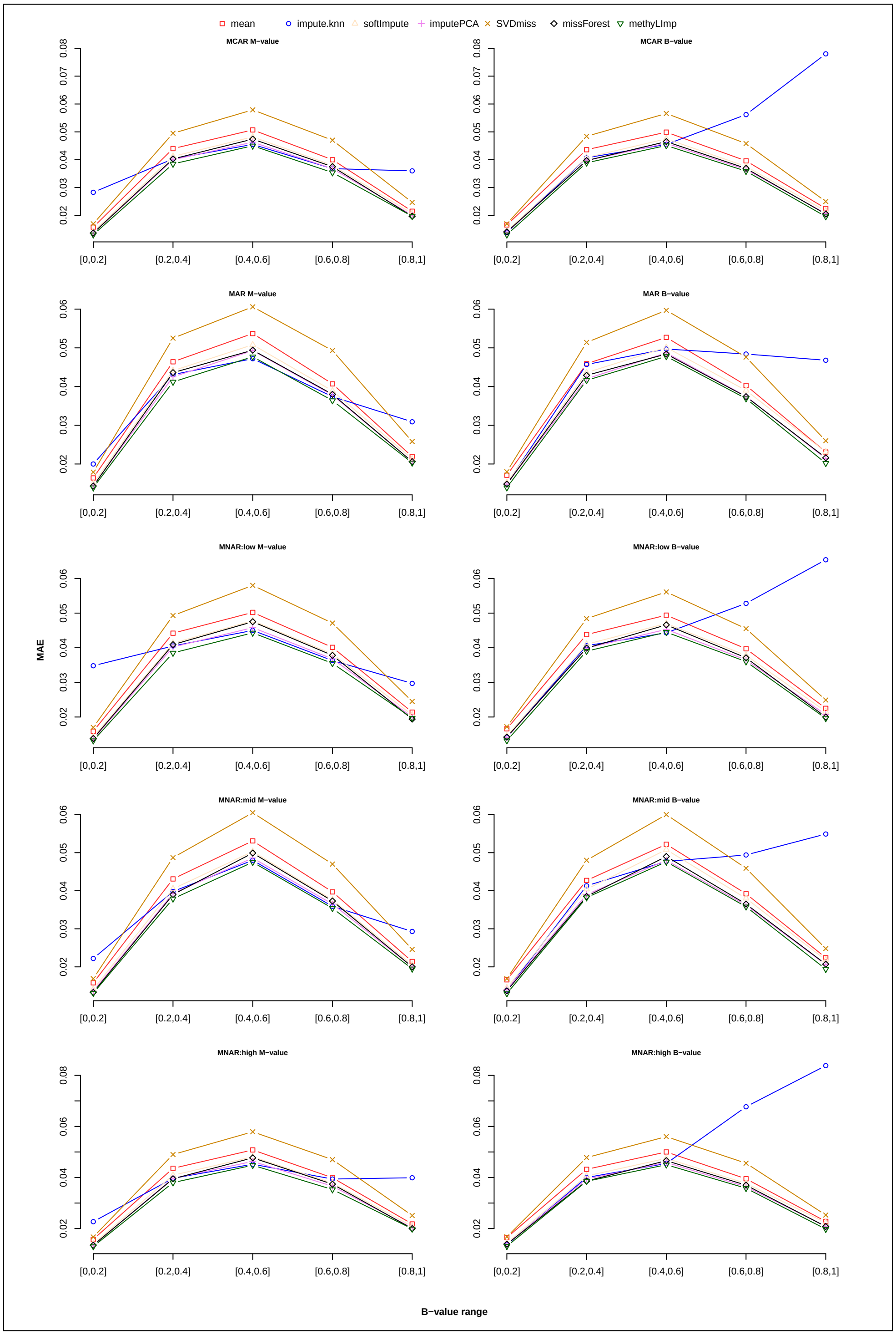

Figure 115: Dataset GSE89705 (D56). MAE imputation performances with respect to B-value range.

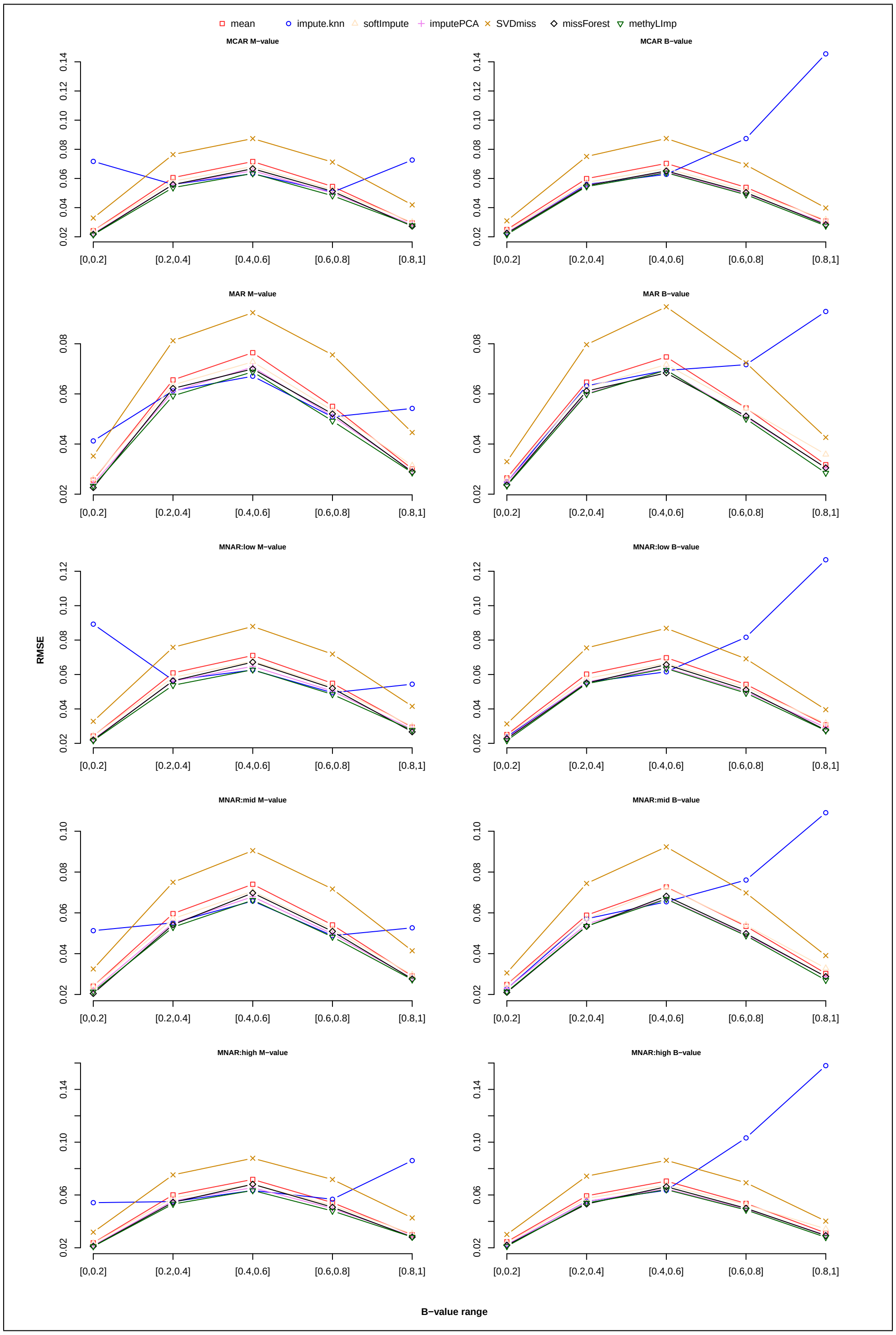

Figure 116: Dataset GSE89705 (D56). RMSE imputation performances with respect to B-value range.

2.57 GSE89706 (D57) - Putamen - Normal - 28 samples

Method	Avg time (sec)	Avg RAM (Mb)
mean	< 1	25
softImpute	< 1	66
imputePCA	8	188
impute.knn	1	77
SVDmiss	81	3280
methyLImp	154	99
missForest	18562	155

Table 351: Dataset GSE89706 (D57). Average time and memory usage.

Table 352: Dataset GSE89706 (D57). Imputation performance on **MCAR** type missing values.

	MAE		RMSE	
Method	M-value	B-value	M-value	B-value
mean	0.024±0.001*	0.024±0.001	0.036±0.001	0.036±0.001*
softImpute	0.022±0.001*	0.022±0.001	0.032±0.001*	0.032±0.001
impute.knn	0.026±0.003*	0.035±0.007	0.052±0.011*	0.074±0.016
imputePCA	0.021±0.001*	0.022±0.001	0.032±0.001	0.032±0.001
SVDmiss	0.021±0.001*	0.022±0.001	0.032±0.001	0.032±0.001*
missForest	0.022±0.001*	0.022±0.001	0.033±0.001	0.032±0.001
methyLImp	**0.020±0.001***	0.021±0.001	**0.031±0.001***	0.031±0.001

Table 353: Dataset GSE89706 (D57). Imputation performance on **MAR** type missing values.

	MAE		RMSE	
Method	M-value	B-value	M-value	B-value
mean	0.025±0.001*	0.025±0.001	0.038±0.001	0.038±0.001*
softImpute	0.023±0.001*	0.024±0.001	0.035±0.001*	0.035±0.001
impute.knn	0.026±0.002*	0.031±0.004	0.047±0.008*	0.061±0.013
imputePCA	0.023±0.001*	0.023±0.001	0.035±0.001	0.035±0.001
SVDmiss	0.023±0.001*	0.024±0.001	0.036±0.001*	0.036±0.001
missForest	0.023±0.001*	0.024±0.001	0.036±0.001	0.036±0.001
methyLImp	**0.022±0.001***	0.022±0.001	**0.034±0.001***	0.034±0.001

Table 354: Dataset GSE89706 (D57). Imputation performance on **MNAR:low** type missing values.

	MAE		RMSE	
Method	M-value	B-value	M-value	B-value
mean	0.018±0.001*	0.018±0.001	0.029±0.001*	0.029±0.001
softImpute	0.016±0.001*	0.017±0.001	0.026±0.001*	0.026±0.001
impute.knn	0.020±0.003*	0.022±0.003	0.046±0.014*	0.050±0.010
imputePCA	0.016±0.001*	0.016±0.001	0.026±0.001*	0.026±0.001
SVDmiss	0.016±0.001*	0.017±0.001	0.026±0.001*	0.026±0.001
missForest	0.016±0.001*	0.017±0.001	0.026±0.001*	0.026±0.001
methyLImp	**0.016±0.001***	0.016±0.001	**0.025±0.001***	0.025±0.001

Table 355: Dataset GSE89706 (D57). Imputation performance on **MNAR:mid** type missing values.

	MAE		RMSE	
Method	M-value	B-value	M-value	B-value
mean	0.039±0.001	0.039±0.001*	0.056±0.001	0.055±0.001*
softImpute	0.034±0.001	0.034±0.001*	0.048±0.001	0.048±0.001*
impute.knn	0.034±0.001*	0.036±0.002	0.052±0.003*	0.058±0.006
imputePCA	0.034±0.001	0.034±0.001*	0.048±0.001	0.048±0.001*
SVDmiss	0.033±0.001	0.033±0.001*	0.048±0.001	0.047±0.001*
missForest	0.034±0.001	0.034±0.001*	0.049±0.001	0.048±0.001*
methyLImp	0.032±0.001	**0.032±0.001***	0.046±0.001	**0.045±0.001***

Table 356: Dataset GSE89706 (D57). Imputation performance on **MNAR:high** type missing values.

	MAE		RMSE	
Method	M-value	B-value	M-value	B-value
mean	0.021±0.001*	0.021±0.001	0.031±0.001*	0.031±0.001
softImpute	0.019±0.001*	0.020±0.001	0.028±0.001*	0.029±0.001
impute.knn	0.025±0.004*	0.040±0.009	0.050±0.014*	0.089±0.020
imputePCA	0.019±0.001*	0.020±0.001	0.028±0.001*	0.028±0.001
SVDmiss	0.020±0.001*	0.020±0.001	0.028±0.001*	0.029±0.001
missForest	0.019±0.001*	0.020±0.001	0.028±0.001*	0.029±0.001
methyLImp	**0.019±0.001***	0.019±0.001	**0.027±0.001***	0.027±0.001

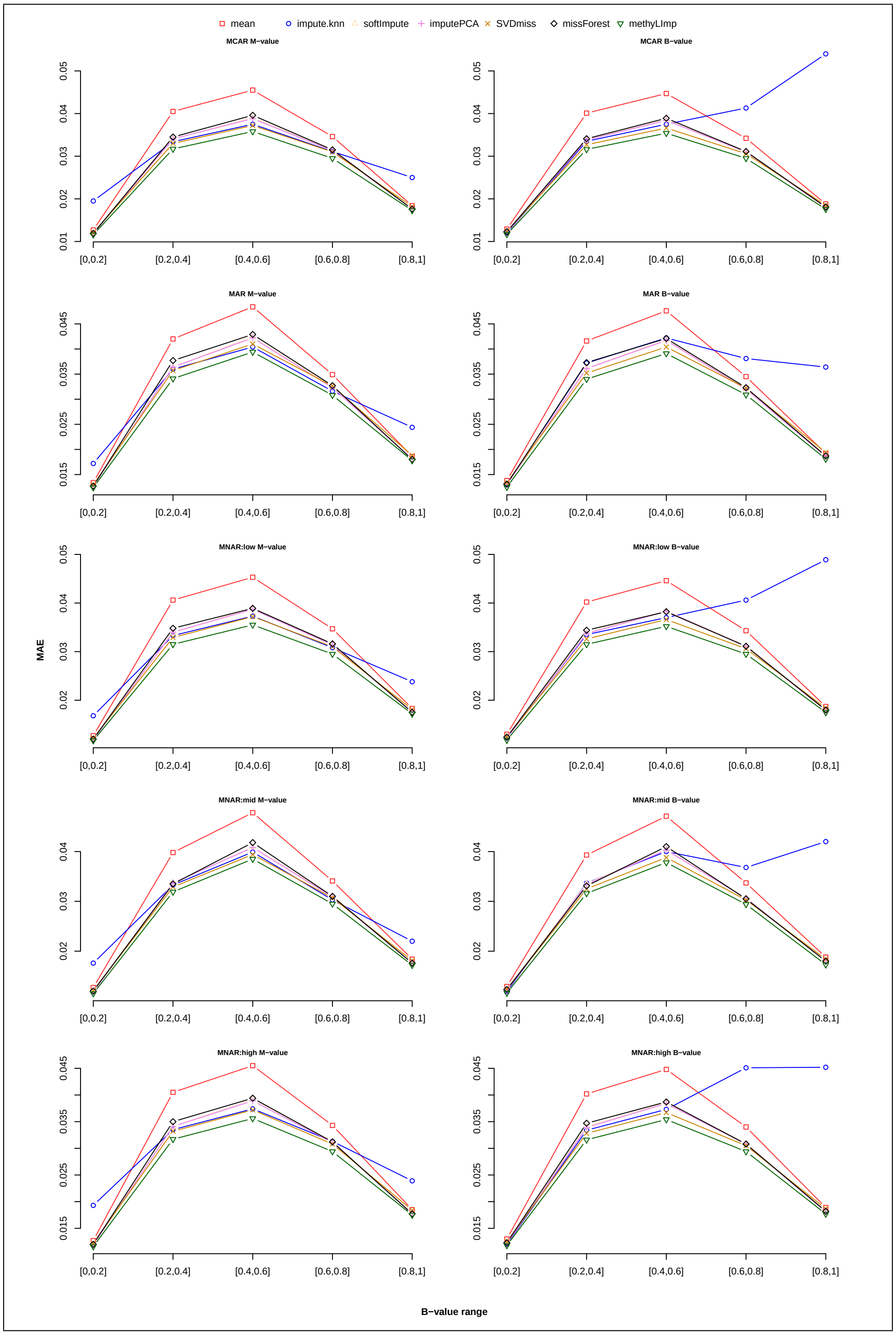

Figure 117: Dataset GSE89706 (D57). MAE imputation performances with respect to B-value range.

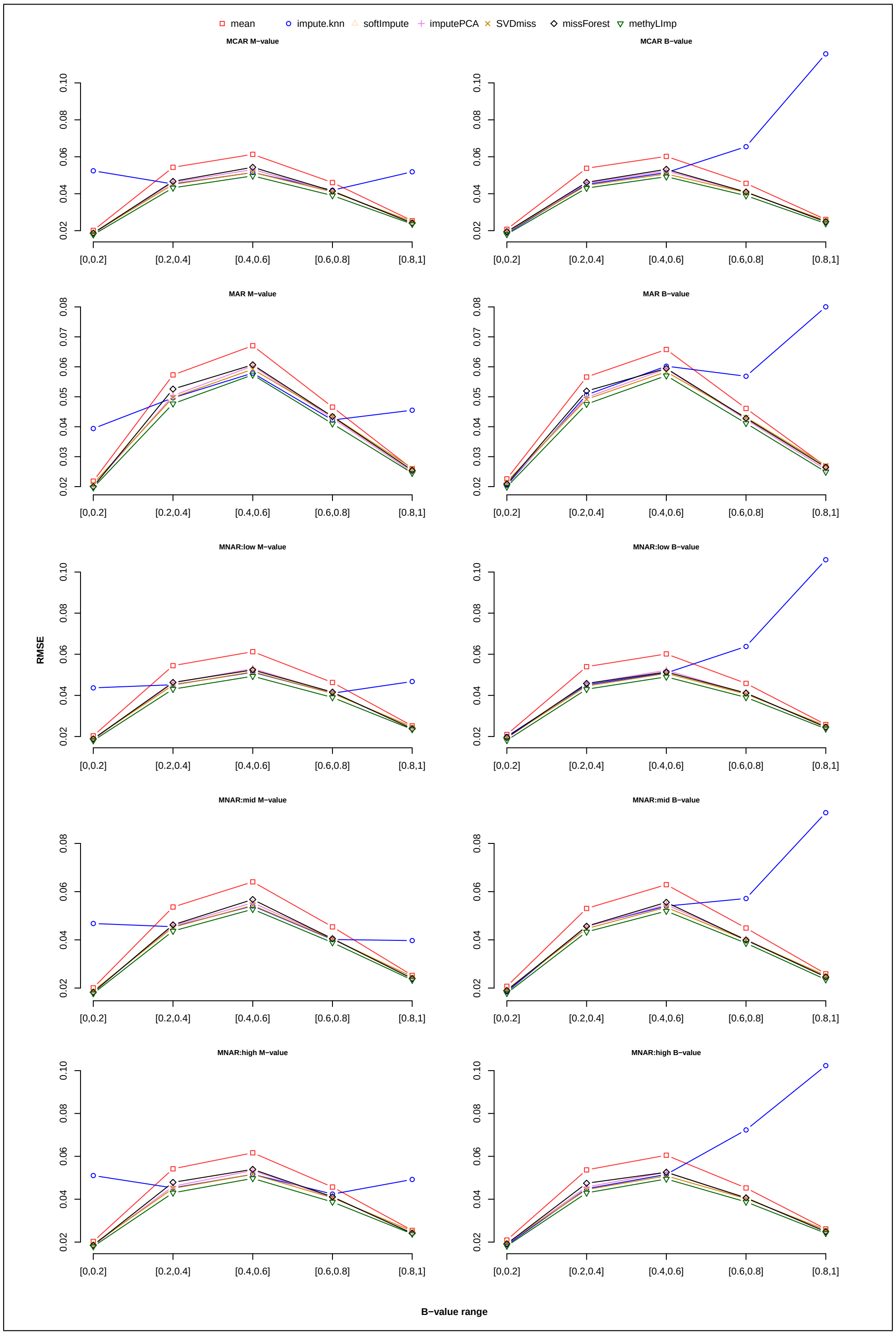

Figure 118: Dataset GSE89706 (D57). RMSE imputation performances with respect to B-value range.

2.58 GSE97362 (D58) - Blood - Normal - 123 samples

Method	Avg time (sec)	Avg RAM (Mb)
mean	< 1	99
softImpute	1	193
imputePCA	48	429
impute.knn	6	217
SVDmiss	535	5200
methyLImp	8430	202
missForest	508336	664

Table 357: Dataset GSE97362 (D58). Average time and memory usage.

Table 358: Dataset GSE97362 (D58). Imputation performance on **MCAR** type missing values.

	MAE		RMSE	
Method	M-value	B-value	M-value	B-value
mean	0.022±0.001	0.022±0.001*	0.044±0.001	0.042±0.001*
softImpute	0.022±0.001	0.020±0.001*	0.045±0.001	0.036±0.001*
impute.knn	0.018±0.001*	0.019±0.001	0.038±0.001*	0.042±0.005
imputePCA	0.020±0.001	0.020±0.001*	0.043±0.001	0.041±0.001*
SVDmiss	0.022±0.001	0.018±0.001*	0.045±0.001	0.033±0.001*
methyLImp	0.034±0.002	0.030±0.001*	0.094±0.004	0.088±0.002*
missForest	0.017±0.001	**0.017±0.001***	0.036±0.001	**0.033±0.001***

Table 359: Dataset GSE97362 (D58). Imputation performance on **MAR** type missing values.

	MAE		RMSE	
Method	M-value	B-value	M-value	B-value
mean	0.040±0.001*	0.041±0.001	0.086±0.001	0.079±0.001*
softImpute	0.040±0.001	0.029±0.001*	0.086±0.001	0.059±0.001*
impute.knn	0.031±0.001	0.031±0.002	0.073±0.002*	0.077±0.009
imputePCA	0.039±0.001*	0.039±0.001	0.086±0.001	0.078±0.001*
SVDmiss	0.041±0.001	0.026±0.001*	0.090±0.001	0.057±0.001*
methyLImp	0.050±0.001	0.033±0.001*	0.113±0.002	0.081±0.001*
missForest	0.028±0.001	**0.026±0.001***	0.067±0.001	**0.056±0.001***

Table 360: Dataset GSE97362 (D58). Imputation performance on **MNAR:low** type missing values.

	MAE		RMSE	
Method	M-value	B-value	M-value	B-value
mean	0.018±0.001*	0.018±0.001	0.038±0.001*	0.040±0.001
softImpute	0.018±0.001	0.016±0.001*	0.038±0.001	0.033±0.001*
impute.knn	0.014±0.001*	0.015±0.001	0.034±0.001*	0.040±0.004
imputePCA	0.017±0.001*	0.017±0.001	0.038±0.001*	0.039±0.001
SVDmiss	0.018±0.001	0.015±0.001*	0.043±0.001	0.032±0.001*
methyLImp	0.021±0.001*	0.022±0.001	0.064±0.004*	0.073±0.003
missForest	0.014±0.001	**0.013±0.001***	0.032±0.001	**0.031±0.001***

Table 361: Dataset GSE97362 (D58). Imputation performance on **MNAR:mid** type missing values.

	MAE		RMSE	
Method	M-value	B-value	M-value	B-value
mean	0.048±0.001	0.045±0.001*	0.088±0.001	0.077±0.001*
softImpute	0.048±0.001	0.037±0.001*	0.087±0.001	0.059±0.001*
impute.knn	0.035±0.001	0.034±0.001*	0.066±0.001	0.060±0.003*
imputePCA	0.045±0.001	0.042±0.001*	0.085±0.001	0.076±0.001*
SVDmiss	0.048±0.001	0.032±0.001*	0.087±0.001	0.054±0.001*
methyLImp	0.059±0.001	0.035±0.001*	0.103±0.003	0.067±0.001*
missForest	0.034±0.001	**0.031±0.001***	0.061±0.001	**0.053±0.001***

Table 362: Dataset GSE97362 (D58). Imputation performance on **MNAR:high** type missing values.

	MAE		RMSE	
Method	M-value	B-value	M-value	B-value
mean	0.019±0.001*	0.019±0.001	0.039±0.001	0.036±0.001*
softImpute	0.019±0.001	0.018±0.001*	0.039±0.001	0.032±0.001*
impute.knn	0.016±0.001*	0.018±0.001	0.034±0.001*	0.041±0.007
imputePCA	0.018±0.001*	0.018±0.001	0.037±0.001	0.035±0.001*
SVDmiss	0.019±0.001	0.016±0.001*	0.039±0.001	0.030±0.001*
methyLImp	0.038±0.001	0.020±0.001*	0.088±0.002	0.054±0.001*
missForest	0.016±0.001	**0.015±0.001***	0.032±0.001	**0.029±0.001***

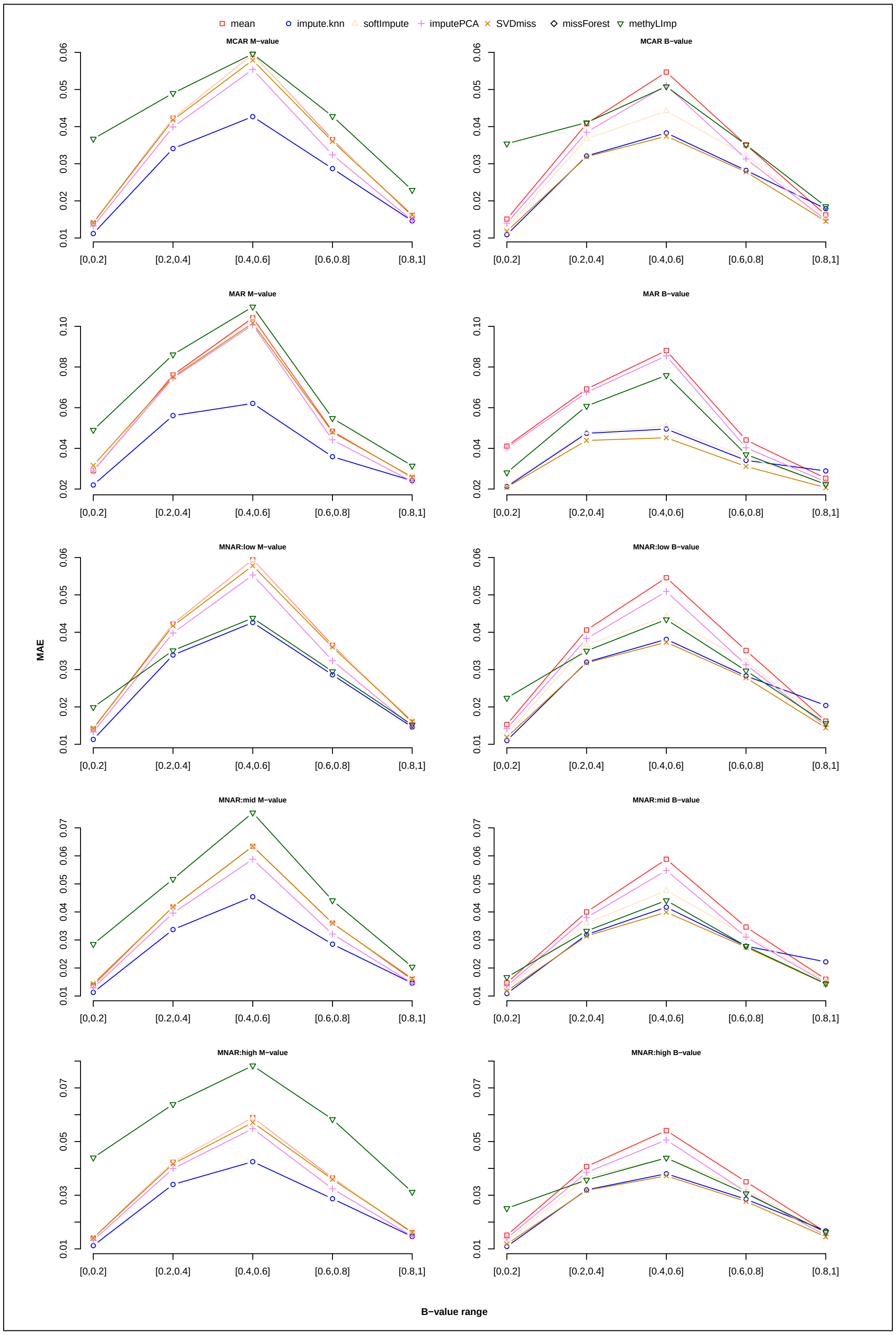

Figure 119: Dataset GSE97362 (D58). MAE imputation performances with respect to B-value range.

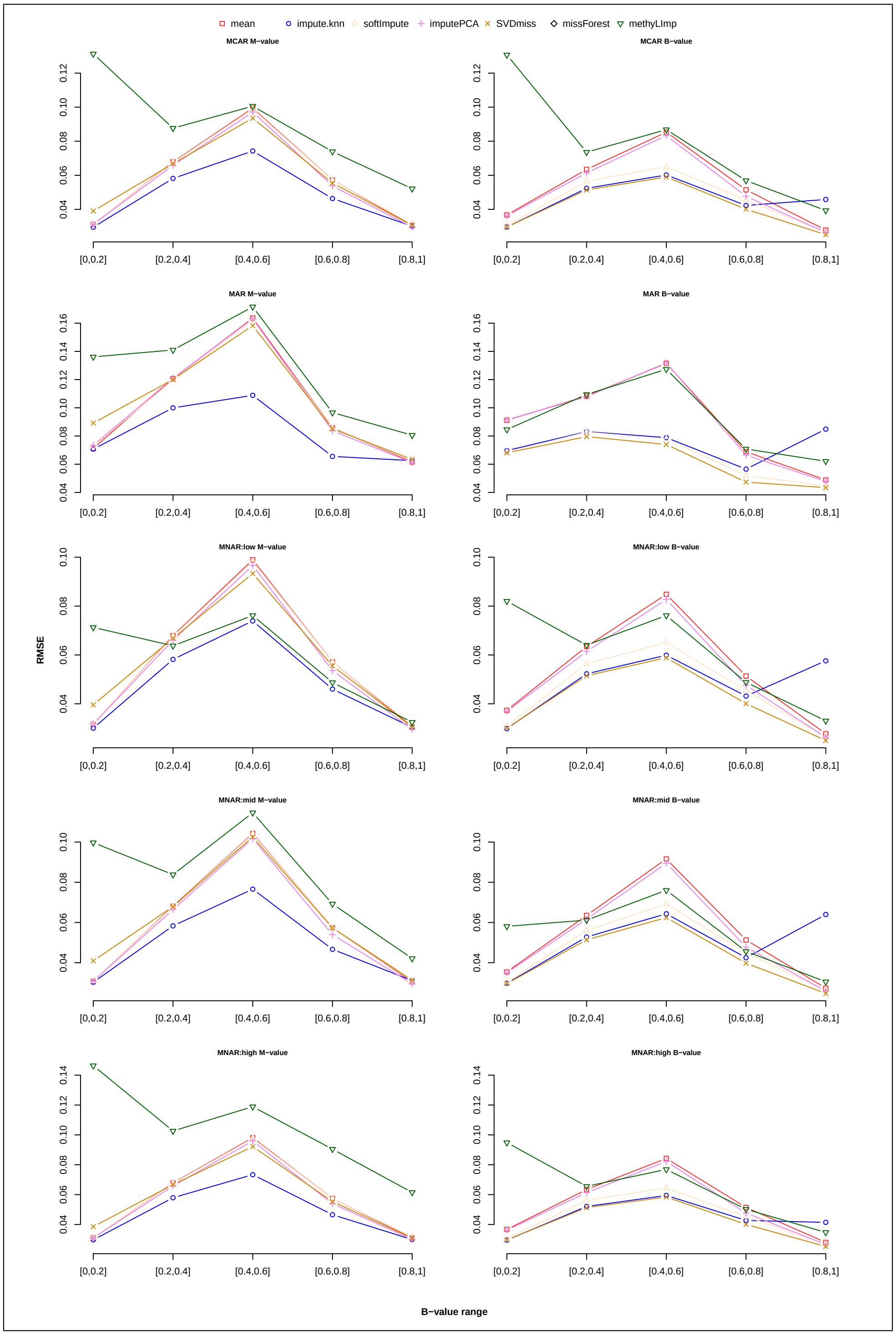

Figure 120: Dataset GSE97362 (D58). RMSE imputation performances with respect to B-value range.

List of Figures

1 Healthy samples. MAE imputation performances with respect to B-value range. 4
2 Healthy samples. RMSE imputation performances with respect to B-value range. 5
3 Disease samples. MAE imputation performances with respect to B-value range. 9
4 Disease samples. RMSE imputation performances with respect to B-value range. 10
5 Dataset GSE32146 (D1). MAE imputation performances with respect to B-value range. . . . 13
6 Dataset GSE32146 (D1). RMSE imputation performances with respect to B-value range. . . 14
7 Dataset GSE32146 (D2). MAE imputation performances with respect to B-value range. . . . 17
8 Dataset GSE32146 (D2). RMSE imputation performances with respect to B-value range. . . 18
9 Dataset GSE32146 (D3). MAE imputation performances with respect to B-value range. . . . 21
10 Dataset GSE32146 (D3). RMSE imputation performances with respect to B-value range. . . 22
11 Dataset GSE32148 (D4). MAE imputation performances with respect to B-value range. . . . 25
12 Dataset GSE32148 (D4). RMSE imputation performances with respect to B-value range. . . 26
13 Dataset GSE40005 (D5). MAE imputation performances with respect to B-value range. . . . 29
14 Dataset GSE40005 (D5). RMSE imputation performances with respect to B-value range. . . 30
15 Dataset GSE42921 (D6). MAE imputation performances with respect to B-value range. . . . 33
16 Dataset GSE42921 (D6). RMSE imputation performances with respect to B-value range. . . 34
17 Dataset GSE42921 (D7). MAE imputation performances with respect to B-value range. . . . 37
18 Dataset GSE42921 (D7). RMSE imputation performances with respect to B-value range. . . 38
19 Dataset GSE42921 (D8). MAE imputation performances with respect to B-value range. . . . 41
20 Dataset GSE42921 (D8). RMSE imputation performances with respect to B-value range. . . 42
21 Dataset GSE43091 (D9). MAE imputation performances with respect to B-value range. . . . 45
22 Dataset GSE43091 (D9). RMSE imputation performances with respect to B-value range. . . 46
23 Dataset GSE43091 (D10). MAE imputation performances with respect to B-value range. . . 49
24 Dataset GSE43091 (D10). RMSE imputation performances with respect to B-value range. . . 50
25 Dataset GSE44684 (D11). MAE imputation performances with respect to B-value range. . . 53
26 Dataset GSE44684 (D11). RMSE imputation performances with respect to B-value range. . . 54
27 Dataset GSE49393 (D12). MAE imputation performances with respect to B-value range. . . 57
28 Dataset GSE49393 (D12). RMSE imputation performances with respect to B-value range. . . 58
29 Dataset GSE51388 (D13). MAE imputation performances with respect to B-value range. . . 61
30 Dataset GSE51388 (D13). RMSE imputation performances with respect to B-value range. . . 62
31 Dataset GSE52113 (D14). MAE imputation performances with respect to B-value range. . . 65
32 Dataset GSE52113 (D14). RMSE imputation performances with respect to B-value range. . . 66
33 Dataset GSE53051 (D15). MAE imputation performances with respect to B-value range. . . 69
34 Dataset GSE53051 (D15). RMSE imputation performances with respect to B-value range. . . 70
35 Dataset GSE53051 (D16). MAE imputation performances with respect to B-value range. . . 73
36 Dataset GSE53051 (D16). RMSE imputation performances with respect to B-value range. . . 74
37 Dataset GSE53051 (D17). MAE imputation performances with respect to B-value range. . . 77
38 Dataset GSE53051 (D17). RMSE imputation performances with respect to B-value range. . . 78
39 Dataset GSE53051 (D18). MAE imputation performances with respect to B-value range. . . 81
40 Dataset GSE53051 (D18). RMSE imputation performances with respect to B-value range. . . 82
41 Dataset GSE53051 (D19). MAE imputation performances with respect to B-value range. . . 85
42 Dataset GSE53051 (D19). RMSE imputation performances with respect to B-value range. . . 86
43 Dataset GSE53051 (D20). MAE imputation performances with respect to B-value range. . . 89
44 Dataset GSE53051 (D20). RMSE imputation performances with respect to B-value range. . . 90
45 Dataset GSE53162 (D21). MAE imputation performances with respect to B-value range. . . 93

46 Dataset GSE53162 (D21). RMSE imputation performances with respect to B-value range. . . 94
47 Dataset GSE53740 (D22). MAE imputation performances with respect to B-value range. . . 97
48 Dataset GSE53740 (D22). RMSE imputation performances with respect to B-value range. . . 98
49 Dataset GSE57360 (D23). MAE imputation performances with respect to B-value range. . . 101
50 Dataset GSE57360 (D23). RMSE imputation performances with respect to B-value range. . . 102
51 Dataset GSE61151 (D24). MAE imputation performances with respect to B-value range. . . 105
52 Dataset GSE61151 (D24). RMSE imputation performances with respect to B-value range. . . 106
53 Dataset GSE61257 (D25). MAE imputation performances with respect to B-value range. . . 109
54 Dataset GSE61257 (D25). RMSE imputation performances with respect to B-value range. . . 110
55 Dataset GSE61257 (D26). MAE imputation performances with respect to B-value range. . . 113
56 Dataset GSE61257 (D26). RMSE imputation performances with respect to B-value range. . . 114
57 Dataset GSE61257 (D27). MAE imputation performances with respect to B-value range. . . 117
58 Dataset GSE61257 (D27). RMSE imputation performances with respect to B-value range. . . 118
59 Dataset GSE61258 (D28). MAE imputation performances with respect to B-value range. . . 121
60 Dataset GSE61258 (D28). RMSE imputation performances with respect to B-value range. . . 122
61 Dataset GSE61258 (D29). MAE imputation performances with respect to B-value range. . . 125
62 Dataset GSE61258 (D29). RMSE imputation performances with respect to B-value range. . . 126
63 Dataset GSE61258 (D30). MAE imputation performances with respect to B-value range. . . 129
64 Dataset GSE61258 (D30). RMSE imputation performances with respect to B-value range. . . 130
65 Dataset GSE61258 (D31). MAE imputation performances with respect to B-value range. . . 133
66 Dataset GSE61258 (D31). RMSE imputation performances with respect to B-value range. . . 134
67 Dataset GSE61258 (D32). MAE imputation performances with respect to B-value range. . . 137
68 Dataset GSE61258 (D32). RMSE imputation performances with respect to B-value range. . . 138
69 Dataset GSE61259 (D33). MAE imputation performances with respect to B-value range. . . 141
70 Dataset GSE61259 (D33). RMSE imputation performances with respect to B-value range. . . 142
71 Dataset GSE61259 (D34). MAE imputation performances with respect to B-value range. . . 145
72 Dataset GSE61259 (D34). RMSE imputation performances with respect to B-value range. . . 146
73 Dataset GSE61259 (D35). MAE imputation performances with respect to B-value range. . . 149
74 Dataset GSE61259 (D35). RMSE imputation performances with respect to B-value range. . . 150
75 Dataset GSE61380 (D36). MAE imputation performances with respect to B-value range. . . 153
76 Dataset GSE61380 (D36). RMSE imputation performances with respect to B-value range. . . 154
77 Dataset GSE62003 (D37). MAE imputation performances with respect to B-value range. . . 157
78 Dataset GSE62003 (D37). RMSE imputation performances with respect to B-value range. . . 158
79 Dataset GSE64495 (D38). MAE imputation performances with respect to B-value range. . . 161
80 Dataset GSE64495 (D38). RMSE imputation performances with respect to B-value range. . . 162
81 Dataset GSE67477 (D39). MAE imputation performances with respect to B-value range. . . 165
82 Dataset GSE67477 (D39). RMSE imputation performances with respect to B-value range. . . 166
83 Dataset GSE67484 (D40). MAE imputation performances with respect to B-value range. . . 169
84 Dataset GSE67484 (D40). RMSE imputation performances with respect to B-value range. . . 170
85 Dataset GSE69502 (D41). MAE imputation performances with respect to B-value range. . . 173
86 Dataset GSE69502 (D41). RMSE imputation performances with respect to B-value range. . . 174
87 Dataset GSE71955 (D42). MAE imputation performances with respect to B-value range. . . 177
88 Dataset GSE71955 (D42). RMSE imputation performances with respect to B-value range. . . 178
89 Dataset GSE73103 (D43). MAE imputation performances with respect to B-value range. . . 181
90 Dataset GSE73103 (D43). RMSE imputation performances with respect to B-value range. . . 182
91 Dataset GSE73747 (D44). MAE imputation performances with respect to B-value range. . . 185
92 Dataset GSE73747 (D44). RMSE imputation performances with respect to B-value range. . . 186

93 Dataset GSE79122 (D45). MAE imputation performances with respect to B-value range. . . 189
94 Dataset GSE79122 (D45). RMSE imputation performances with respect to B-value range. . . 190
95 Dataset GSE80970 (D46). MAE imputation performances with respect to B-value range. . . 193
96 Dataset GSE80970 (D46). RMSE imputation performances with respect to B-value range. . . 194
97 Dataset GSE82218 (D47). MAE imputation performances with respect to B-value range. . . 197
98 Dataset GSE82218 (D47). RMSE imputation performances with respect to B-value range. . . 198
99 Dataset GSE84003 (D48). MAE imputation performances with respect to B-value range. . . 201
100 Dataset GSE84003 (D48). RMSE imputation performances with respect to B-value range. . . 202
101 Dataset GSE88821 (D49). MAE imputation performances with respect to B-value range. . . 205
102 Dataset GSE88821 (D49). RMSE imputation performances with respect to B-value range. . . 206
103 Dataset GSE88821 (D50). MAE imputation performances with respect to B-value range. . . 209
104 Dataset GSE88821 (D50). RMSE imputation performances with respect to B-value range. . . 210
105 Dataset GSE88821 (D51). MAE imputation performances with respect to B-value range. . . 213
106 Dataset GSE88821 (D51). RMSE imputation performances with respect to B-value range. . . 214
107 Dataset GSE89093 (D52). MAE imputation performances with respect to B-value range. . . 217
108 Dataset GSE89093 (D52). RMSE imputation performances with respect to B-value range. . . 218
109 Dataset GSE89472 (D53). MAE imputation performances with respect to B-value range. . . 221
110 Dataset GSE89472 (D53). RMSE imputation performances with respect to B-value range. . . 222
111 Dataset GSE89702 (D54). MAE imputation performances with respect to B-value range. . . 225
112 Dataset GSE89702 (D54). RMSE imputation performances with respect to B-value range. . . 226
113 Dataset GSE89703 (D55). MAE imputation performances with respect to B-value range. . . 229
114 Dataset GSE89703 (D55). RMSE imputation performances with respect to B-value range. . . 230
115 Dataset GSE89705 (D56). MAE imputation performances with respect to B-value range. . . 233
116 Dataset GSE89705 (D56). RMSE imputation performances with respect to B-value range. . . 234
117 Dataset GSE89706 (D57). MAE imputation performances with respect to B-value range. . . 237
118 Dataset GSE89706 (D57). RMSE imputation performances with respect to B-value range. . . 238
119 Dataset GSE97362 (D58). MAE imputation performances with respect to B-value range. . . 241
120 Dataset GSE97362 (D58). RMSE imputation performances with respect to B-value range. . . 242

List of Tables

1 Healthy samples. Average time and memory usage. 1
2 Healthy samples. Global imputation performances. 1
3 Healthy samples. Imputation performance on **MCAR** type missing values. 2
4 Healthy samples. Imputation performance on **MAR** type missing values. 2
5 Healthy samples. Imputation performance on **MNAR:low** type missing values. 2
6 Healthy samples. Imputation performance on **MNAR:mid** type missing values. 3
7 Healthy samples. Imputation performance on **MNAR:high** type missing values. 3
8 Disease samples. Average time and memory usage. 6
9 Disease samples. Global imputation performances. 6
10 Disease samples. Imputation performance on **MCAR** type missing values. 6
11 Disease samples. Imputation performance on **MAR** type missing values. 7
12 Disease samples. Imputation performance on **MNAR:low** type missing values. 7
13 Disease samples. Imputation performance on **MNAR:mid** type missing values. 7
14 Disease samples. Imputation performance on **MNAR:high** type missing values. 8
15 Dataset GSE32146 (D1). Average time and memory usage. 11

16 Dataset GSE32146 (D1). Imputation performance on **MCAR** type missing values. 11
17 Dataset GSE32146 (D1). Imputation performance on **MAR** type missing values. 11
18 Dataset GSE32146 (D1). Imputation performance on **MNAR:low** type missing values. 12
19 Dataset GSE32146 (D1). Imputation performance on **MNAR:mid** type missing values. 12
20 Dataset GSE32146 (D1). Imputation performance on **MNAR:high** type missing values. 12
21 Dataset GSE32146 (D2). Average time and memory usage. 15
22 Dataset GSE32146 (D2). Imputation performance on **MCAR** type missing values. 15
23 Dataset GSE32146 (D2). Imputation performance on **MAR** type missing values. 15
24 Dataset GSE32146 (D2). Imputation performance on **MNAR:low** type missing values. 16
25 Dataset GSE32146 (D2). Imputation performance on **MNAR:mid** type missing values. 16
26 Dataset GSE32146 (D2). Imputation performance on **MNAR:high** type missing values. 16
27 Dataset GSE32146 (D3). Average time and memory usage. 19
28 Dataset GSE32146 (D3). Imputation performance on **MCAR** type missing values. 19
29 Dataset GSE32146 (D3). Imputation performance on **MAR** type missing values. 19
30 Dataset GSE32146 (D3). Imputation performance on **MNAR:low** type missing values. 20
31 Dataset GSE32146 (D3). Imputation performance on **MNAR:mid** type missing values. 20
32 Dataset GSE32146 (D3). Imputation performance on **MNAR:high** type missing values. 20
33 Dataset GSE32148 (D4). Average time and memory usage. 23
34 Dataset GSE32148 (D4). Imputation performance on **MCAR** type missing values. 23
35 Dataset GSE32148 (D4). Imputation performance on **MAR** type missing values. 23
36 Dataset GSE32148 (D4). Imputation performance on **MNAR:low** type missing values. 24
37 Dataset GSE32148 (D4). Imputation performance on **MNAR:mid** type missing values. 24
38 Dataset GSE32148 (D4). Imputation performance on **MNAR:high** type missing values. 24
39 Dataset GSE40005 (D5). Average time and memory usage. 27
40 Dataset GSE40005 (D5). Imputation performance on **MCAR** type missing values. 27
41 Dataset GSE40005 (D5). Imputation performance on **MAR** type missing values. 27
42 Dataset GSE40005 (D5). Imputation performance on **MNAR:low** type missing values. 28
43 Dataset GSE40005 (D5). Imputation performance on **MNAR:mid** type missing values. 28
44 Dataset GSE40005 (D5). Imputation performance on **MNAR:high** type missing values. 28
45 Dataset GSE42921 (D6). Average time and memory usage. 31
46 Dataset GSE42921 (D6). Imputation performance on **MCAR** type missing values. 31
47 Dataset GSE42921 (D6). Imputation performance on **MAR** type missing values. 31
48 Dataset GSE42921 (D6). Imputation performance on **MNAR:low** type missing values. 32
49 Dataset GSE42921 (D6). Imputation performance on **MNAR:mid** type missing values. 32
50 Dataset GSE42921 (D6). Imputation performance on **MNAR:high** type missing values. 32
51 Dataset GSE42921 (D7). Average time and memory usage. 35
52 Dataset GSE42921 (D7). Imputation performance on **MCAR** type missing values. 35
53 Dataset GSE42921 (D7). Imputation performance on **MAR** type missing values. 35
54 Dataset GSE42921 (D7). Imputation performance on **MNAR:low** type missing values. 36
55 Dataset GSE42921 (D7). Imputation performance on **MNAR:mid** type missing values. 36
56 Dataset GSE42921 (D7). Imputation performance on **MNAR:high** type missing values. 36
57 Dataset GSE42921 (D8). Average time and memory usage. 39
58 Dataset GSE42921 (D8). Imputation performance on **MCAR** type missing values. 39
59 Dataset GSE42921 (D8). Imputation performance on **MAR** type missing values. 39
60 Dataset GSE42921 (D8). Imputation performance on **MNAR:low** type missing values. 40
61 Dataset GSE42921 (D8). Imputation performance on **MNAR:mid** type missing values. 40
62 Dataset GSE42921 (D8). Imputation performance on **MNAR:high** type missing values. 40

63 Dataset GSE43091 (D9). Average time and memory usage. 43
64 Dataset GSE43091 (D9). Imputation performance on **MCAR** type missing values. 43
65 Dataset GSE43091 (D9). Imputation performance on **MAR** type missing values. 43
66 Dataset GSE43091 (D9). Imputation performance on **MNAR:low** type missing values. 44
67 Dataset GSE43091 (D9). Imputation performance on **MNAR:mid** type missing values. 44
68 Dataset GSE43091 (D9). Imputation performance on **MNAR:high** type missing values. 44
69 Dataset GSE43091 (D10). Average time and memory usage. 47
70 Dataset GSE43091 (D10). Imputation performance on **MCAR** type missing values. 47
71 Dataset GSE43091 (D10). Imputation performance on **MAR** type missing values. 47
72 Dataset GSE43091 (D10). Imputation performance on **MNAR:low** type missing values. 48
73 Dataset GSE43091 (D10). Imputation performance on **MNAR:mid** type missing values. 48
74 Dataset GSE43091 (D10). Imputation performance on **MNAR:high** type missing values. 48
75 Dataset GSE44684 (D11). Average time and memory usage. 51
76 Dataset GSE44684 (D11). Imputation performance on **MCAR** type missing values. 51
77 Dataset GSE44684 (D11). Imputation performance on **MAR** type missing values. 51
78 Dataset GSE44684 (D11). Imputation performance on **MNAR:low** type missing values. 52
79 Dataset GSE44684 (D11). Imputation performance on **MNAR:mid** type missing values. 52
80 Dataset GSE44684 (D11). Imputation performance on **MNAR:high** type missing values. 52
81 Dataset GSE49393 (D12). Average time and memory usage. 55
82 Dataset GSE49393 (D12). Imputation performance on **MCAR** type missing values. 55
83 Dataset GSE49393 (D12). Imputation performance on **MAR** type missing values. 55
84 Dataset GSE49393 (D12). Imputation performance on **MNAR:low** type missing values. 56
85 Dataset GSE49393 (D12). Imputation performance on **MNAR:mid** type missing values. 56
86 Dataset GSE49393 (D12). Imputation performance on **MNAR:high** type missing values. 56
87 Dataset GSE51388 (D13). Average time and memory usage. 59
88 Dataset GSE51388 (D13). Imputation performance on **MCAR** type missing values. 59
89 Dataset GSE51388 (D13). Imputation performance on **MAR** type missing values. 59
90 Dataset GSE51388 (D13). Imputation performance on **MNAR:low** type missing values. 60
91 Dataset GSE51388 (D13). Imputation performance on **MNAR:mid** type missing values. 60
92 Dataset GSE51388 (D13). Imputation performance on **MNAR:high** type missing values. 60
93 Dataset GSE52113 (D14). Average time and memory usage. 63
94 Dataset GSE52113 (D14). Imputation performance on **MCAR** type missing values. 63
95 Dataset GSE52113 (D14). Imputation performance on **MAR** type missing values. 63
96 Dataset GSE52113 (D14). Imputation performance on **MNAR:low** type missing values. 64
97 Dataset GSE52113 (D14). Imputation performance on **MNAR:mid** type missing values. 64
98 Dataset GSE52113 (D14). Imputation performance on **MNAR:high** type missing values. 64
99 Dataset GSE53051 (D15). Average time and memory usage. 67
100 Dataset GSE53051 (D15). Imputation performance on **MCAR** type missing values. 67
101 Dataset GSE53051 (D15). Imputation performance on **MAR** type missing values. 67
102 Dataset GSE53051 (D15). Imputation performance on **MNAR:low** type missing values. 68
103 Dataset GSE53051 (D15). Imputation performance on **MNAR:mid** type missing values. 68
104 Dataset GSE53051 (D15). Imputation performance on **MNAR:high** type missing values. 68
105 Dataset GSE53051 (D16). Average time and memory usage. 71
106 Dataset GSE53051 (D16). Imputation performance on **MCAR** type missing values. 71
107 Dataset GSE53051 (D16). Imputation performance on **MAR** type missing values. 71
108 Dataset GSE53051 (D16). Imputation performance on **MNAR:low** type missing values. 72
109 Dataset GSE53051 (D16). Imputation performance on **MNAR:mid** type missing values. 72

110 Dataset GSE53051 (D16). Imputation performance on **MNAR:high** type missing values. 72
111 Dataset GSE53051 (D17). Average time and memory usage. 75
112 Dataset GSE53051 (D17). Imputation performance on **MCAR** type missing values. 75
113 Dataset GSE53051 (D17). Imputation performance on **MAR** type missing values. 75
114 Dataset GSE53051 (D17). Imputation performance on **MNAR:low** type missing values. 76
115 Dataset GSE53051 (D17). Imputation performance on **MNAR:mid** type missing values. 76
116 Dataset GSE53051 (D17). Imputation performance on **MNAR:high** type missing values. 76
117 Dataset GSE53051 (D18). Average time and memory usage. 79
118 Dataset GSE53051 (D18). Imputation performance on **MCAR** type missing values. 79
119 Dataset GSE53051 (D18). Imputation performance on **MAR** type missing values. 79
120 Dataset GSE53051 (D18). Imputation performance on **MNAR:low** type missing values. 80
121 Dataset GSE53051 (D18). Imputation performance on **MNAR:mid** type missing values. 80
122 Dataset GSE53051 (D18). Imputation performance on **MNAR:high** type missing values. 80
123 Dataset GSE53051 (D19). Average time and memory usage. 83
124 Dataset GSE53051 (D19). Imputation performance on **MCAR** type missing values. 83
125 Dataset GSE53051 (D19). Imputation performance on **MAR** type missing values. 83
126 Dataset GSE53051 (D19). Imputation performance on **MNAR:low** type missing values. 84
127 Dataset GSE53051 (D19). Imputation performance on **MNAR:mid** type missing values. 84
128 Dataset GSE53051 (D19). Imputation performance on **MNAR:high** type missing values. 84
129 Dataset GSE53051 (D20). Average time and memory usage. 87
130 Dataset GSE53051 (D20). Imputation performance on **MCAR** type missing values. 87
131 Dataset GSE53051 (D20). Imputation performance on **MAR** type missing values. 87
132 Dataset GSE53051 (D20). Imputation performance on **MNAR:low** type missing values. 88
133 Dataset GSE53051 (D20). Imputation performance on **MNAR:mid** type missing values. 88
134 Dataset GSE53051 (D20). Imputation performance on **MNAR:high** type missing values. 88
135 Dataset GSE53162 (D21). Average time and memory usage. 91
136 Dataset GSE53162 (D21). Imputation performance on **MCAR** type missing values. 91
137 Dataset GSE53162 (D21). Imputation performance on **MAR** type missing values. 91
138 Dataset GSE53162 (D21). Imputation performance on **MNAR:low** type missing values. 92
139 Dataset GSE53162 (D21). Imputation performance on **MNAR:mid** type missing values. 92
140 Dataset GSE53162 (D21). Imputation performance on **MNAR:high** type missing values. 92
141 Dataset GSE53740 (D22). Average time and memory usage. 95
142 Dataset GSE53740 (D22). Imputation performance on **MCAR** type missing values. 95
143 Dataset GSE53740 (D22). Imputation performance on **MAR** type missing values. 95
144 Dataset GSE53740 (D22). Imputation performance on **MNAR:low** type missing values. 96
145 Dataset GSE53740 (D22). Imputation performance on **MNAR:mid** type missing values. 96
146 Dataset GSE53740 (D22). Imputation performance on **MNAR:high** type missing values. 96
147 Dataset GSE57360 (D23). Average time and memory usage. 99
148 Dataset GSE57360 (D23). Imputation performance on **MCAR** type missing values. 99
149 Dataset GSE57360 (D23). Imputation performance on **MAR** type missing values. 99
150 Dataset GSE57360 (D23). Imputation performance on **MNAR:low** type missing values. 100
151 Dataset GSE57360 (D23). Imputation performance on **MNAR:mid** type missing values. 100
152 Dataset GSE57360 (D23). Imputation performance on **MNAR:high** type missing values. 100
153 Dataset GSE61151 (D24). Average time and memory usage. 103
154 Dataset GSE61151 (D24). Imputation performance on **MCAR** type missing values. 103
155 Dataset GSE61151 (D24). Imputation performance on **MAR** type missing values. 103
156 Dataset GSE61151 (D24). Imputation performance on **MNAR:low** type missing values. 104

157 Dataset GSE61151 (D24). Imputation performance on **MNAR:mid** type missing values. 104
158 Dataset GSE61151 (D24). Imputation performance on **MNAR:high** type missing values. 104
159 Dataset GSE61257 (D25). Average time and memory usage. 107
160 Dataset GSE61257 (D25). Imputation performance on **MCAR** type missing values. 107
161 Dataset GSE61257 (D25). Imputation performance on **MAR** type missing values. 107
162 Dataset GSE61257 (D25). Imputation performance on **MNAR:low** type missing values. 108
163 Dataset GSE61257 (D25). Imputation performance on **MNAR:mid** type missing values. 108
164 Dataset GSE61257 (D25). Imputation performance on **MNAR:high** type missing values. 108
165 Dataset GSE61257 (D26). Average time and memory usage. 111
166 Dataset GSE61257 (D26). Imputation performance on **MCAR** type missing values. 111
167 Dataset GSE61257 (D26). Imputation performance on **MAR** type missing values. 111
168 Dataset GSE61257 (D26). Imputation performance on **MNAR:low** type missing values. 112
169 Dataset GSE61257 (D26). Imputation performance on **MNAR:mid** type missing values. 112
170 Dataset GSE61257 (D26). Imputation performance on **MNAR:high** type missing values. 112
171 Dataset GSE61257 (D27). Average time and memory usage. 115
172 Dataset GSE61257 (D27). Imputation performance on **MCAR** type missing values. 115
173 Dataset GSE61257 (D27). Imputation performance on **MAR** type missing values. 115
174 Dataset GSE61257 (D27). Imputation performance on **MNAR:low** type missing values. 116
175 Dataset GSE61257 (D27). Imputation performance on **MNAR:mid** type missing values. 116
176 Dataset GSE61257 (D27). Imputation performance on **MNAR:high** type missing values. 116
177 Dataset GSE61258 (D28). Average time and memory usage. 119
178 Dataset GSE61258 (D28). Imputation performance on **MCAR** type missing values. 119
179 Dataset GSE61258 (D28). Imputation performance on **MAR** type missing values. 119
180 Dataset GSE61258 (D28). Imputation performance on **MNAR:low** type missing values. 120
181 Dataset GSE61258 (D28). Imputation performance on **MNAR:mid** type missing values. 120
182 Dataset GSE61258 (D28). Imputation performance on **MNAR:high** type missing values. 120
183 Dataset GSE61258 (D29). Average time and memory usage. 123
184 Dataset GSE61258 (D29). Imputation performance on **MCAR** type missing values. 123
185 Dataset GSE61258 (D29). Imputation performance on **MAR** type missing values. 123
186 Dataset GSE61258 (D29). Imputation performance on **MNAR:low** type missing values. 124
187 Dataset GSE61258 (D29). Imputation performance on **MNAR:mid** type missing values. 124
188 Dataset GSE61258 (D29). Imputation performance on **MNAR:high** type missing values. 124
189 Dataset GSE61258 (D30). Average time and memory usage. 127
190 Dataset GSE61258 (D30). Imputation performance on **MCAR** type missing values. 127
191 Dataset GSE61258 (D30). Imputation performance on **MAR** type missing values. 127
192 Dataset GSE61258 (D30). Imputation performance on **MNAR:low** type missing values. 128
193 Dataset GSE61258 (D30). Imputation performance on **MNAR:mid** type missing values. 128
194 Dataset GSE61258 (D30). Imputation performance on **MNAR:high** type missing values. 128
195 Dataset GSE61258 (D31). Average time and memory usage. 131
196 Dataset GSE61258 (D31). Imputation performance on **MCAR** type missing values. 131
197 Dataset GSE61258 (D31). Imputation performance on **MAR** type missing values. 131
198 Dataset GSE61258 (D31). Imputation performance on **MNAR:low** type missing values. 132
199 Dataset GSE61258 (D31). Imputation performance on **MNAR:mid** type missing values. 132
200 Dataset GSE61258 (D31). Imputation performance on **MNAR:high** type missing values. 132
201 Dataset GSE61258 (D32). Average time and memory usage. 135
202 Dataset GSE61258 (D32). Imputation performance on **MCAR** type missing values. 135
203 Dataset GSE61258 (D32). Imputation performance on **MAR** type missing values. 135

204 Dataset GSE61258 (D32). Imputation performance on **MNAR:low** type missing values. 136
205 Dataset GSE61258 (D32). Imputation performance on **MNAR:mid** type missing values. 136
206 Dataset GSE61258 (D32). Imputation performance on **MNAR:high** type missing values. 136
207 Dataset GSE61259 (D33). Average time and memory usage. 139
208 Dataset GSE61259 (D33). Imputation performance on **MCAR** type missing values. 139
209 Dataset GSE61259 (D33). Imputation performance on **MAR** type missing values. 139
210 Dataset GSE61259 (D33). Imputation performance on **MNAR:low** type missing values. 140
211 Dataset GSE61259 (D33). Imputation performance on **MNAR:mid** type missing values. 140
212 Dataset GSE61259 (D33). Imputation performance on **MNAR:high** type missing values. 140
213 Dataset GSE61259 (D34). Average time and memory usage. 143
214 Dataset GSE61259 (D34). Imputation performance on **MCAR** type missing values. 143
215 Dataset GSE61259 (D34). Imputation performance on **MAR** type missing values. 143
216 Dataset GSE61259 (D34). Imputation performance on **MNAR:low** type missing values. 144
217 Dataset GSE61259 (D34). Imputation performance on **MNAR:mid** type missing values. 144
218 Dataset GSE61259 (D34). Imputation performance on **MNAR:high** type missing values. 144
219 Dataset GSE61259 (D35). Average time and memory usage. 147
220 Dataset GSE61259 (D35). Imputation performance on **MCAR** type missing values. 147
221 Dataset GSE61259 (D35). Imputation performance on **MAR** type missing values. 147
222 Dataset GSE61259 (D35). Imputation performance on **MNAR:low** type missing values. 148
223 Dataset GSE61259 (D35). Imputation performance on **MNAR:mid** type missing values. 148
224 Dataset GSE61259 (D35). Imputation performance on **MNAR:high** type missing values. 148
225 Dataset GSE61380 (D36). Average time and memory usage. 151
226 Dataset GSE61380 (D36). Imputation performance on **MCAR** type missing values. 151
227 Dataset GSE61380 (D36). Imputation performance on **MAR** type missing values. 151
228 Dataset GSE61380 (D36). Imputation performance on **MNAR:low** type missing values. 152
229 Dataset GSE61380 (D36). Imputation performance on **MNAR:mid** type missing values. 152
230 Dataset GSE61380 (D36). Imputation performance on **MNAR:high** type missing values. 152
231 Dataset GSE62003 (D37). Average time and memory usage. 155
232 Dataset GSE62003 (D37). Imputation performance on **MCAR** type missing values. 155
233 Dataset GSE62003 (D37). Imputation performance on **MAR** type missing values. 155
234 Dataset GSE62003 (D37). Imputation performance on **MNAR:low** type missing values. 156
235 Dataset GSE62003 (D37). Imputation performance on **MNAR:mid** type missing values. 156
236 Dataset GSE62003 (D37). Imputation performance on **MNAR:high** type missing values. 156
237 Dataset GSE64495 (D38). Average time and memory usage. 159
238 Dataset GSE64495 (D38). Imputation performance on **MCAR** type missing values. 159
239 Dataset GSE64495 (D38). Imputation performance on **MAR** type missing values. 159
240 Dataset GSE64495 (D38). Imputation performance on **MNAR:low** type missing values. 160
241 Dataset GSE64495 (D38). Imputation performance on **MNAR:mid** type missing values. 160
242 Dataset GSE64495 (D38). Imputation performance on **MNAR:high** type missing values. 160
243 Dataset GSE67477 (D39). Average time and memory usage. 163
244 Dataset GSE67477 (D39). Imputation performance on **MCAR** type missing values. 163
245 Dataset GSE67477 (D39). Imputation performance on **MAR** type missing values. 163
246 Dataset GSE67477 (D39). Imputation performance on **MNAR:low** type missing values. 164
247 Dataset GSE67477 (D39). Imputation performance on **MNAR:mid** type missing values. 164
248 Dataset GSE67477 (D39). Imputation performance on **MNAR:high** type missing values. 164
249 Dataset GSE67484 (D40). Average time and memory usage. 167
250 Dataset GSE67484 (D40). Imputation performance on **MCAR** type missing values. 167

251 Dataset GSE67484 (D40). Imputation performance on **MAR** type missing values. 167
252 Dataset GSE67484 (D40). Imputation performance on **MNAR:low** type missing values. 168
253 Dataset GSE67484 (D40). Imputation performance on **MNAR:mid** type missing values. 168
254 Dataset GSE67484 (D40). Imputation performance on **MNAR:high** type missing values. 168
255 Dataset GSE69502 (D41). Average time and memory usage. 171
256 Dataset GSE69502 (D41). Imputation performance on **MCAR** type missing values. 171
257 Dataset GSE69502 (D41). Imputation performance on **MAR** type missing values. 171
258 Dataset GSE69502 (D41). Imputation performance on **MNAR:low** type missing values. 172
259 Dataset GSE69502 (D41). Imputation performance on **MNAR:mid** type missing values. 172
260 Dataset GSE69502 (D41). Imputation performance on **MNAR:high** type missing values. 172
261 Dataset GSE71955 (D42). Average time and memory usage. 175
262 Dataset GSE71955 (D42). Imputation performance on **MCAR** type missing values. 175
263 Dataset GSE71955 (D42). Imputation performance on **MAR** type missing values. 175
264 Dataset GSE71955 (D42). Imputation performance on **MNAR:low** type missing values. 176
265 Dataset GSE71955 (D42). Imputation performance on **MNAR:mid** type missing values. 176
266 Dataset GSE71955 (D42). Imputation performance on **MNAR:high** type missing values. 176
267 Dataset GSE73103 (D43). Average time and memory usage. 179
268 Dataset GSE73103 (D43). Imputation performance on **MCAR** type missing values. 179
269 Dataset GSE73103 (D43). Imputation performance on **MAR** type missing values. 179
270 Dataset GSE73103 (D43). Imputation performance on **MNAR:low** type missing values. 180
271 Dataset GSE73103 (D43). Imputation performance on **MNAR:mid** type missing values. 180
272 Dataset GSE73103 (D43). Imputation performance on **MNAR:high** type missing values. 180
273 Dataset GSE73747 (D44). Average time and memory usage. 183
274 Dataset GSE73747 (D44). Imputation performance on **MCAR** type missing values. 183
275 Dataset GSE73747 (D44). Imputation performance on **MAR** type missing values. 183
276 Dataset GSE73747 (D44). Imputation performance on **MNAR:low** type missing values. 184
277 Dataset GSE73747 (D44). Imputation performance on **MNAR:mid** type missing values. 184
278 Dataset GSE73747 (D44). Imputation performance on **MNAR:high** type missing values. 184
279 Dataset GSE79122 (D45). Average time and memory usage. 187
280 Dataset GSE79122 (D45). Imputation performance on **MCAR** type missing values. 187
281 Dataset GSE79122 (D45). Imputation performance on **MAR** type missing values. 187
282 Dataset GSE79122 (D45). Imputation performance on **MNAR:low** type missing values. 188
283 Dataset GSE79122 (D45). Imputation performance on **MNAR:mid** type missing values. 188
284 Dataset GSE79122 (D45). Imputation performance on **MNAR:high** type missing values. 188
285 Dataset GSE80970 (D46). Average time and memory usage. 191
286 Dataset GSE80970 (D46). Imputation performance on **MCAR** type missing values. 191
287 Dataset GSE80970 (D46). Imputation performance on **MAR** type missing values. 191
288 Dataset GSE80970 (D46). Imputation performance on **MNAR:low** type missing values. 192
289 Dataset GSE80970 (D46). Imputation performance on **MNAR:mid** type missing values. 192
290 Dataset GSE80970 (D46). Imputation performance on **MNAR:high** type missing values. 192
291 Dataset GSE82218 (D47). Average time and memory usage. 195
292 Dataset GSE82218 (D47). Imputation performance on **MCAR** type missing values. 195
293 Dataset GSE82218 (D47). Imputation performance on **MAR** type missing values. 195
294 Dataset GSE82218 (D47). Imputation performance on **MNAR:low** type missing values. 196
295 Dataset GSE82218 (D47). Imputation performance on **MNAR:mid** type missing values. 196
296 Dataset GSE82218 (D47). Imputation performance on **MNAR:high** type missing values. 196
297 Dataset GSE84003 (D48). Average time and memory usage. 199

298 Dataset GSE84003 (D48). Imputation performance on **MCAR** type missing values. 199
299 Dataset GSE84003 (D48). Imputation performance on **MAR** type missing values. 199
300 Dataset GSE84003 (D48). Imputation performance on **MNAR:low** type missing values. 200
301 Dataset GSE84003 (D48). Imputation performance on **MNAR:mid** type missing values. 200
302 Dataset GSE84003 (D48). Imputation performance on **MNAR:high** type missing values. 200
303 Dataset GSE88821 (D49). Average time and memory usage. 203
304 Dataset GSE88821 (D49). Imputation performance on **MCAR** type missing values. 203
305 Dataset GSE88821 (D49). Imputation performance on **MAR** type missing values. 203
306 Dataset GSE88821 (D49). Imputation performance on **MNAR:low** type missing values. 204
307 Dataset GSE88821 (D49). Imputation performance on **MNAR:mid** type missing values. 204
308 Dataset GSE88821 (D49). Imputation performance on **MNAR:high** type missing values. 204
309 Dataset GSE88821 (D50). Average time and memory usage. 207
310 Dataset GSE88821 (D50). Imputation performance on **MCAR** type missing values. 207
311 Dataset GSE88821 (D50). Imputation performance on **MAR** type missing values. 207
312 Dataset GSE88821 (D50). Imputation performance on **MNAR:low** type missing values. 208
313 Dataset GSE88821 (D50). Imputation performance on **MNAR:mid** type missing values. 208
314 Dataset GSE88821 (D50). Imputation performance on **MNAR:high** type missing values. 208
315 Dataset GSE88821 (D51). Average time and memory usage. 211
316 Dataset GSE88821 (D51). Imputation performance on **MCAR** type missing values. 211
317 Dataset GSE88821 (D51). Imputation performance on **MAR** type missing values. 211
318 Dataset GSE88821 (D51). Imputation performance on **MNAR:low** type missing values. 212
319 Dataset GSE88821 (D51). Imputation performance on **MNAR:mid** type missing values. 212
320 Dataset GSE88821 (D51). Imputation performance on **MNAR:high** type missing values. 212
321 Dataset GSE89093 (D52). Average time and memory usage. 215
322 Dataset GSE89093 (D52). Imputation performance on **MCAR** type missing values. 215
323 Dataset GSE89093 (D52). Imputation performance on **MAR** type missing values. 215
324 Dataset GSE89093 (D52). Imputation performance on **MNAR:low** type missing values. 216
325 Dataset GSE89093 (D52). Imputation performance on **MNAR:mid** type missing values. 216
326 Dataset GSE89093 (D52). Imputation performance on **MNAR:high** type missing values. 216
327 Dataset GSE89472 (D53). Average time and memory usage. 219
328 Dataset GSE89472 (D53). Imputation performance on **MCAR** type missing values. 219
329 Dataset GSE89472 (D53). Imputation performance on **MAR** type missing values. 219
330 Dataset GSE89472 (D53). Imputation performance on **MNAR:low** type missing values. 220
331 Dataset GSE89472 (D53). Imputation performance on **MNAR:mid** type missing values. 220
332 Dataset GSE89472 (D53). Imputation performance on **MNAR:high** type missing values. 220
333 Dataset GSE89702 (D54). Average time and memory usage. 223
334 Dataset GSE89702 (D54). Imputation performance on **MCAR** type missing values. 223
335 Dataset GSE89702 (D54). Imputation performance on **MAR** type missing values. 223
336 Dataset GSE89702 (D54). Imputation performance on **MNAR:low** type missing values. 224
337 Dataset GSE89702 (D54). Imputation performance on **MNAR:mid** type missing values. 224
338 Dataset GSE89702 (D54). Imputation performance on **MNAR:high** type missing values. 224
339 Dataset GSE89703 (D55). Average time and memory usage. 227
340 Dataset GSE89703 (D55). Imputation performance on **MCAR** type missing values. 227
341 Dataset GSE89703 (D55). Imputation performance on **MAR** type missing values. 227
342 Dataset GSE89703 (D55). Imputation performance on **MNAR:low** type missing values. 228
343 Dataset GSE89703 (D55). Imputation performance on **MNAR:mid** type missing values. 228
344 Dataset GSE89703 (D55). Imputation performance on **MNAR:high** type missing values. 228

345 Dataset GSE89705 (D56). Average time and memory usage. 231
346 Dataset GSE89705 (D56). Imputation performance on **MCAR** type missing values. 231
347 Dataset GSE89705 (D56). Imputation performance on **MAR** type missing values. 231
348 Dataset GSE89705 (D56). Imputation performance on **MNAR:low** type missing values. 232
349 Dataset GSE89705 (D56). Imputation performance on **MNAR:mid** type missing values. 232
350 Dataset GSE89705 (D56). Imputation performance on **MNAR:high** type missing values. 232
351 Dataset GSE89706 (D57). Average time and memory usage. 235
352 Dataset GSE89706 (D57). Imputation performance on **MCAR** type missing values. 235
353 Dataset GSE89706 (D57). Imputation performance on **MAR** type missing values. 235
354 Dataset GSE89706 (D57). Imputation performance on **MNAR:low** type missing values. 236
355 Dataset GSE89706 (D57). Imputation performance on **MNAR:mid** type missing values. 236
356 Dataset GSE89706 (D57). Imputation performance on **MNAR:high** type missing values. 236
357 Dataset GSE97362 (D58). Average time and memory usage. 239
358 Dataset GSE97362 (D58). Imputation performance on **MCAR** type missing values. 239
359 Dataset GSE97362 (D58). Imputation performance on **MAR** type missing values. 239
360 Dataset GSE97362 (D58). Imputation performance on **MNAR:low** type missing values. 240
361 Dataset GSE97362 (D58). Imputation performance on **MNAR:mid** type missing values. 240
362 Dataset GSE97362 (D58). Imputation performance on **MNAR:high** type missing values. 240
363 Healthy samples. Average time and memory usage performance comparison between complete/restricted datasets 244
364 Healthy samples. **MCAR** type missing values. Performance comparison between complete/restricted datasets 244
365 Healthy samples. **MAR** type missing values. Performance comparison between complete/restricted datasets 244
366 Healthy samples. **MNAR:low** type missing values. Performance comparison between complete/restricted datasets 245
367 Healthy samples. **MNAR:mid** type missing values. Performance comparison between complete/restricted datasets 245
368 Healthy samples. **MNAR:high** type missing values. Performance comparison between complete/restricted datasets 245
369 Disease samples. Average time and memory usage performance comparison between complete/restricted datasets 246
370 Disease samples. **MCAR** type missing values. Performance comparison between complete/restricted datasets 246
371 Disease samples. **MAR** type missing values. Performance comparison between complete/restricted datasets 246
372 Disease samples. **MNAR:low** type missing values. Performance comparison between complete/restricted datasets 247
373 Disease samples. **MNAR:mid** type missing values. Performance comparison between complete/restricted datasets 247
374 Disease samples. **MNAR:high** type missing values. Performance comparison between complete/restricted datasets 247
