# Supplementary material for: Methylation data imputation performances under different representations and missingness patterns
Source: BMC Bioinformatics. 2020 Jun 29;21:268. doi: 10.1186/s12859-020-03592-5 (PMC7325236; doi:10.1186/s12859-020-03592-5)
Supplement: Supplementary file 2 — Additional file 2 Dataset statistics. [file 12859_2020_3592_MOESM2_ESM.pdf]

Dataset statistics

1 Dataset statistics

Table 1: Benchmark datasets

ID	GEO ID	Tissue	Disease status	# Samples	# Missing values (21k)	% Missing values (21k)
D1	GSE32146	Colon mucosa	Crohn's disease	10	175	0.08%
D2	GSE32146	Colon mucosa	Ulcerative colitis	5	161	0.15%
D3	GSE32146	Colon	Normal	10	171	0.08%
D4	GSE32148	Blood	Normal	19	325	0.08%
D5	GSE40005	Blood	Normal	12	324	0.13%
D6	GSE42921	Colon mucosa	Crohn's disease	5	192	0.18%
D7	GSE42921	Colon mucosa	Ulcerative colitis	6	331	0.26%
D8	GSE42921	Colon	Normal	12	874	0.34%
D9	GSE43091	Liver	Cancer	50	1,980	0.19%
D10	GSE43091	Liver	Normal	4	125	0.15%
D11	GSE44684	Cerebellum	Normal	6	67	0.05%
D12	GSE49393	Prefrontal Cortex	Normal	25	54,000	10.11%
D13	GSE51388	Blood	Normal	60	292,200	22.79%
D14	GSE52113	Blood	Normal	24	0	0.00%
D15	GSE53051	Breast	Cancer	14	0	0.00%
D16	GSE53051	Colon	Cancer	35	0	0.00%
D17	GSE53051	Colon, Pancreas	Normal	9	0	0.00%
D18	GSE53051	Lung	Cancer	9	0	0.00%
D19	GSE53051	Pancreas	Cancer	29	0	0.00%
D20	GSE53051	Thyroid	Cancer	70	0	0.00%
D21	GSE53162	Brain, Cerebellum, Prefrontal Cortex	Normal	21	0	0.00%
D22	GSE53740	Blood	Normal	165	0	0.00%
D23	GSE57360	Brain	Normal	5	0	0.00%
D24	GSE61151	Blood	Normal	184	7,544	0.19%
D25	GSE61257	Adipose	Non-alcoholic fatty liver disease (NAFLD)	8	88	0.05%
D26	GSE61257	Adipose	Non-alcoholic steatohepatitis (NASH)	9	142	0.07%
D27	GSE61257	Adipose	Normal	15	241	0.08%
D28	GSE61258	Liver	Non-alcoholic fatty liver disease (NAFLD)	14	370	0.12%
D29	GSE61258	Liver	Non-alcoholic steatohepatitis (NASH)	7	218	0.15%
D30	GSE61258	Liver	Normal	32	966	0.14%
D31	GSE61258	Liver	Primary biliary cholangitis (PBC)	12	251	0.10%
D32	GSE61258	Liver	Primary sclerosing cholangitis (PSC)	14	352	0.12%
D33	GSE61259	Muscle	Non-alcoholic fatty liver disease (NAFLD)	9	90	0.05%
D34	GSE61259	Muscle	Non-alcoholic steatohepatitis (NASH)	7	49	0.03%
D35	GSE61259	Muscle	Normal	10	96	0.04%
D36	GSE61380	Brain	Normal	15	2,4671	7.70%
D37	GSE62003	Blood	Normal	35	0	0.00%
D38	GSE64495	Blood	Normal	106	32	0.00%
D39	GSE67477	Liver	Cancer	6	461	0.36%
D40	GSE67484	Liver, Intestine-Small	Normal	4	45	0.05%
D41	GSE69502	Brain, Spinal Cord	Normal	20	37,781	8.84%
D42	GSE71955	Blood	Normal	62	260,245	19.64%
D43	GSE73103	Blood	Normal	268	1,005,268	17.55%
D44	GSE73747	Brain	Normal	9	7,069	3.68%
D45	GSE79122	Brain	Normal	7	99	0.07%
D46	GSE80970	Prefrontal Cortex	Normal	68	1,324	0.09%
D47	GSE82218	Blood	Normal	25	398	0.07%
D48	GSE84003	Blood	Normal	6	275	0.21%
D49	GSE88821	Colon, Rectum	Cancer	63	36,995	2.75%
D50	GSE88821	Colon, Rectum	Normal	8	4,680	2.74%
D51	GSE88821	Liver	Cancer	4	2,349	2.75%
D52	GSE89093	Blood	Normal	46	65,044	6.62%
D53	GSE89472	Blood	Normal	5	245	0.23%
D54	GSE89702	Cerebellum	Normal	17	49,572	13.65%
D55	GSE89703	Hippocampus	Normal	13	37,557	13.52%
D56	GSE89705	Putamen	Normal	17	49,215	13.55%
D57	GSE89706	Putamen	Normal	28	78,736	13.16%
D58	GSE97362	Blood	Normal	123	2,333	0.09%
				32	34,926	2.81%

2 Statistics on completely observed data

Table 2: Percentage of completely observed CpGs per dataset with respect to MCAR, MAR, MNAR simulated missing data

		% Completely observed CpGs (21k)					
ID	# Samples	Original	MCAR	MAR	MNAR low	MNAR mid	MNAR high
D1	10	99.30	73.15	80.29	74.97	77.74	75.76
D2	5	99.50	85.26	87.34	85.73	86.44	85.95
D3	10	98.96	73.16	80.34	74.93	78.29	75.85
D4	19	98.79	55.21	72.67	59.77	70.52	59.12
D5	12	98.90	68.41	77.89	70.88	76.25	70.36
D6	5	99.32	85.15	87.26	85.60	86.53	85.70
D7	6	98.61	82.08	85.04	82.81	83.86	82.96
D8	12	97.59	67.08	76.40	69.59	73.95	69.81
D9	50	94.11	20.21	59.19	31.25	35.84	32.78
D10	4	99.57	88.01	89.39	88.35	89.19	88.27
D11	6	99.74	83.04	86.03	83.55	85.13	83.68
D12	25	97.05	41.99	68.82	45.69	55.69	48.20
D13	60	83.24	12.14	57.60	16.63	27.24	13.40
D14	24	100.00	48.12	71.14	54.48	67.86	52.28
D15	14	100.00	65.28	77.18	68.53	69.39	68.10
D16	35	100.00	34.42	67.10	44.12	45.69	45.33
D17	9	100.00	76.02	82.10	77.52	79.30	77.44
D18	9	100.00	76.02	82.10	77.51	77.92	77.59
D19	29	100.00	41.35	69.08	49.44	50.63	49.51
D20	70	100.00	11.86	59.90	24.11	30.87	21.16
D21	21	100.00	52.78	72.62	58.43	65.66	58.39
D22	165	100.00	0.66	52.50	6.12	12.02	3.38
D23	5	100.00	85.89	87.99	86.34	87.23	86.32
D24	184	100.00	0.37	51.70	4.79	11.17	2.20
D25	8	99.65	78.07	83.04	79.19	81.54	80.03
D26	9	99.49	75.51	81.56	76.92	79.60	77.95
D27	15	99.00	62.63	75.65	65.85	71.06	68.29
D28	14	98.36	64.30	76.11	67.31	69.97	68.48
D29	7	99.30	80.10	84.10	80.94	81.50	81.43
D30	32	96.41	36.34	65.82	45.28	53.30	47.32
D31	12	99.01	68.65	78.10	71.21	74.86	71.79
D32	14	98.41	64.26	75.99	67.31	72.59	68.10
D33	9	99.54	75.71	81.78	77.06	79.14	78.83
D34	7	99.73	80.63	84.57	81.48	83.13	82.74
D35	10	99.59	73.41	80.58	74.99	77.37	76.63
D36	15	97.95	58.38	73.37	59.60	66.24	60.93
D37	35	100.00	34.42	67.10	45.32	56.98	44.86
D38	106	99.97	3.96	56.06	14.50	26.29	10.33
D39	6	98.36	81.71	84.70	82.11	82.57	82.10
D40	4	99.71	88.33	89.70	88.57	88.94	88.66
D41	20	98.10	48.13	69.80	49.35	59.65	49.08
D42	62	93.96	12.14	58.95	18.38	27.59	16.86
D43	268	92.39	0.03	48.94	0.39	1.40	0.19
D44	9	99.88	73.18	81.27	73.90	77.97	73.27
D45	7	99.61	80.45	84.39	81.43	83.18	81.07
D46	68	95.76	11.99	57.58	26.45	35.57	26.80
D47	25	98.36	46.02	69.81	53.13	65.51	51.77
D48	6	97.87	82.25	85.21	82.86	85.84	82.64
D49	63	95.37	13.60	58.36	23.46	27.64	27.57
D50	8	99.33	75.84	82.30	76.65	78.81	77.09
D51	4	99.57	85.85	88.32	85.73	86.42	85.94
D52	46	99.78	23.02	64.05	30.77	46.19	26.22
D53	5	99.99	85.69	87.90	86.06	88.49	85.86
D54	17	98.86	51.47	72.62	50.26	60.92	51.77
D55	13	98.92	58.19	74.90	57.48	63.34	58.09
D56	17	98.91	51.53	72.66	51.68	59.27	52.08
D57	28	99.02	37.02	68.38	40.22	51.45	40.30
D58	123	95.10	2.18	51.73	10.34	20.59	7.40
