# Supplementary material for: Methylation data imputation performances under different representations and missingness patterns
Source: BMC Bioinformatics. 2020 Jun 29;21:268. doi: 10.1186/s12859-020-03592-5 (PMC7325236; doi:10.1186/s12859-020-03592-5)
Supplement: Supplementary file 3 — Additional file 3 Performance comparison between complete (450k) and restricted (21k) datasets. [file 12859_2020_3592_MOESM3_ESM.pdf]

# Performance comparison between complete (450k) and restricted (21k) datasets

## 1 Description

We test whether there is a significant difference between imputation performances on complete datasets, consisting of 450k CpGs, and their restriction to 21k CpGs.

Due to the large computational times required to perform tests on complete datasets we restrict them in three ways: i) we do not consider performance assessment for *missForest* and *SVDmiss*, since these two tools are too much demanding in terms of computational time and memory requirements, respectively; ii) we do not consider benchmark datasets containing more than 20 samples, for a total of 37 (out of 58) datasets (21 healthy and 16 disease); iii) we compute only 10 sampling replications for each missingness mechanism instead of 100 as in the main paper, since the sampling procedure itself is computational intensive for the MAR and the MNAR models (it requires several days of computation also on medium-size datasets).

In the following sections we report the average MAE and RMSE performances for healthy and disease datasets, with respect to the 5 missingness models MCAR, MAR, low-range MNAR, mid-range MNAR and high-range MNAR. The result tables directly compare the average performances obtained on the 450k datasets considered in this section with those obtained on the their 21k restriction. For each method, we use the Wilcoxon's test to highlight statistically significantly differences between imputation accuracy on complete versus restricted datasets. These performances are marked with the $^{*}$, $^{**}$ and $^{***}$ symbols, which indicate Wilcoxon's test p-value $< 0.05$, $< 0.01$ and $< 0.001$, respectively. The overall best imputation performances are highlighted in bold, by using the same approach described in the "Wilcoxon-testing procedure to assess statistically significantly better performances" section in the main paper. Here, the paired samples in the Wilcoxon test are the average performances per dataset, while in the main paper we use the (paired) average performances per sampling (for performance comparison, all the methods have been are run on the same sets of artificially introduced NAs). This approach was necessary here since we cannot directly compare (i.e pair) a sampling on complete data (i.e. a set of artificially introduced NAs) with one on restricted data, while we can pair/compare the average performances on complete versus restricted datasets.

We can identify some clearly visible trends in these tests:

- *impute.knn* performances are significantly lower on the complete datasets, irrespectively of the missingness model (MCAR, MAR, low/mid/high-range MNAR), data representation ($M$-value or $\beta$-value) and metric used for performance assessment (MAE or RMSE).

- For the remaining methods, the MAE and RMSE absolute difference between imputations on complete and restricted datasets is overall quite small (on the order of $10^{-3}$).

- Although in absolute terms we observe small differences, the Wilcoxon's test detects some statistically significantly differences between imputations on complete and restricted datasets. Overall (excluding *impute.knn*), MAR missing value imputations are more accurate on the complete datasets, while mid-range MNAR missing value imputations are more accurate on the restricted datasets. For the remaining missingness models there is no significant difference. However, few cases are statistically significant at p-value threshold equal to 0.001 (i.e. performances marked with $^{***}$).

- The performance rankings reflect those reported in the main paper, where the *methyLImp* method is overall best performing. In particular, *methyLImp*'s performances are generally slightly better (bold values) on complete datasets. This is not surprising, since *methyLImp* exploits inter-sample correlations and we can expect to find stronger correlations on complete data.

As a general conclusion, these comparison tests indicate that the imputation performances on the restricted datasets can be considered as representative of the imputation performances on the complete datasets.

## 2 Healthy control samples

| Method | Avg time (sec) | Avg RAM (Mb) |
|---|---|---|
| mean | 2/1 | 167/9 |
| impute.knn | 40/1 | 480/48 |
| softImpute | 26/1 | 411/44 |
| imputePCA | 549/8 | 1039/153 |
| methyLImp | 3049/15 | 37650/110 |

Table 1: Healthy samples. Average time and memory usage performance comparison between complete/restricted datasets

Table 2: Healthy samples. **MCAR** type missing values. Performance comparison between complete/restricted datasets

| | MAE | | RMSE | |
|---|---|---|---|---|
| Method | M-value | B-value | M-value | B-value |
| mean | 0.032*/0.032 | 0.032/0.032 | 0.055/0.055 | 0.054/0.054 |
| impute.knn | 0.209/0.051*** | 0.199/0.083*** | 0.273/0.100*** | 0.266/0.148*** |
| softImpute | 0.038/0.037 | 0.038/0.039 | 0.067/0.065 | 0.073/0.073 |
| imputePCA | 0.027/0.027 | 0.027/0.027 | 0.048/0.048 | **0.046**/0.046 |
| methyLImp | 0.031**/0.031 | **0.026**/0.026 | 0.049/0.049 | **0.046**/0.045 |

Comparison between complete/restricted datasets: *** $p < 0.001$, ** $p < 0.01$ *: $p < 0.05$
Best overall performances per metric are highlighted in bold.

Table 3: Healthy samples. **MAR** type missing values. Performance comparison between complete/restricted datasets

| | MAE | | RMSE | |
|---|---|---|---|---|
| Method | M-value | B-value | M-value | B-value |
| mean | 0.042***/0.043 | 0.041***/0.043 | 0.074**/0.076 | 0.071**/0.073 |
| impute.knn | 0.154/0.053*** | 0.168/0.081*** | 0.222/0.098*** | 0.238/0.138*** |
| softImpute | 0.048**/0.049 | 0.053/0.053 | 0.086/0.087 | 0.104/0.101 |
| imputePCA | 0.038**/0.039 | 0.037***/0.038 | 0.070*/0.072 | **0.067****/0.068 |
| methyLImp | 0.040***/0.042 | **0.035****/0.036 | 0.070**/0.071 | **0.067***/0.068 |

Comparison between complete/restricted datasets: *** $p < 0.001$, ** $p < 0.01$ *: $p < 0.05$
Best overall performances per metric are highlighted in bold.

Table 4: Healthy samples. **MNAR:low** type missing values. Performance comparison between complete/restricted datasets

| | MAE | | RMSE | |
|---|---|---|---|---|
| Method | M-value | B-value | M-value | B-value |
| mean | 0.024/0.024 | 0.025/0.025 | 0.046/0.046 | 0.047/0.047 |
| impute.knn | 0.233/0.058*** | 0.173/0.043*** | 0.288/0.117*** | 0.254/0.098*** |
| softImpute | 0.032/0.032 | 0.026/0.026 | 0.065/0.063 | 0.053/0.054 |
| imputePCA | 0.021/0.021 | 0.021/0.021 | **0.042**/0.042 | 0.041/0.041 |
| methyLImp | 0.025/0.025 | **0.020**/0.020 | **0.042**/0.042 | **0.042**/0.042 |

Comparison between complete/restricted datasets: *** $p < 0.001$, ** $p < 0.01$ *: $p < 0.05$

Best overall performances per metric are highlighted in bold.

Table 5: Healthy samples. **MNAR:mid** type missing values. Performance comparison between complete/restricted datasets

| | MAE | | RMSE | |
|---|---|---|---|---|
| Method | M-value | B-value | M-value | B-value |
| mean | 0.056/0.056* | 0.054/0.053*** | 0.084/0.084 | 0.079/0.078*** |
| impute.knn | 0.089/0.045*** | 0.112/0.059*** | 0.151/0.070*** | 0.177/0.100*** |
| softImpute | 0.056/0.056 | 0.058/0.057 | 0.086/0.084 | 0.095/0.094 |
| imputePCA | 0.047/0.047* | 0.045/0.045*** | 0.074/0.074 | 0.069/0.068*** |
| methyLImp | 0.047/0.047 | 0.043/0.042*** | 0.071/0.071 | 0.067/0.066* |

Comparison between complete/restricted datasets: *** $p < 0.001$, ** $p < 0.01$ *: $p < 0.05$

Best overall performances per metric are highlighted in bold.

Table 6: Healthy samples. **MNAR:high** type missing values. Performance comparison between complete/restricted datasets

| | MAE | | RMSE | |
|---|---|---|---|---|
| Method | M-value | B-value | M-value | B-value |
| mean | 0.028/0.028 | 0.029/0.029 | 0.049/0.048 | 0.048/0.048 |
| impute.knn | 0.265/0.079*** | 0.270/0.137*** | 0.330/0.146*** | 0.326/0.211*** |
| softImpute | 0.034/0.034 | 0.042/0.043 | 0.058/0.058 | 0.082/0.084 |
| imputePCA | 0.024/0.024 | 0.024/0.024 | 0.042/0.042 | 0.041/0.041 |
| methyLImp | 0.030/0.030 | **0.023**/0.023 | 0.046/0.046 | **0.040**/0.040 |

Comparison between complete/restricted datasets: *** $p < 0.001$, ** $p < 0.01$ *: $p < 0.05$

Best overall performances per metric are highlighted in bold.

# 3 Disease case samples

| Method | Avg time (sec) | Avg RAM (Mb) |
|---|---|---|
| mean | 2/1 | 137/7 |
| impute.knn | 44/1 | 409/45 |
| softImpute | 26/1 | 353/42 |
| imputePCA | 598/7 | 957/150 |
| methyLImp | 1277/7 | 44425/120 |

Table 7: Disease samples. Average time and memory usage performance comparison between complete/restricted datasets

Table 8: Disease samples. **MCAR** type missing values. Performance comparison between complete/restricted datasets

| | MAE | | RMSE | |
|---|---|---|---|---|
| Method | M-value | B-value | M-value | B-value |
| mean | 0.044**/0.044 | 0.044*/0.044 | 0.074/0.074 | 0.073/0.073 |
| impute.knn | 0.203/0.062*** | 0.211/0.091*** | 0.261/0.114*** | 0.276/0.158*** |
| softImpute | 0.046/0.046 | 0.051/0.049 | 0.079/0.078 | 0.096/0.091 |
| imputePCA | 0.037/0.037 | 0.037/0.037 | 0.065/0.065 | 0.064/0.064 |
| methyLImp | 0.036*/0.037 | **0.034**/0.034 | 0.062/0.062 | **0.061**/0.061 |

Comparison between complete/restricted datasets: *** $p < 0.001$, ** $p < 0.01$ *: $p < 0.05$

Best overall performances per metric are highlighted in bold.

Table 9: Disease samples. **MAR** type missing values. Performance comparison between complete/restricted datasets

| | MAE | | RMSE | |
|---|---|---|---|---|
| Method | M-value | B-value | M-value | B-value |
| mean | 0.054**/0.056 | 0.054**/0.056 | 0.093**/0.095 | 0.090**/0.093 |
| impute.knn | 0.162/0.069*** | 0.186/0.095*** | 0.225/0.119*** | 0.255/0.157*** |
| softImpute | 0.059/0.058 | 0.059***/0.065 | 0.103/0.101 | 0.111***/0.121 |
| imputePCA | 0.048***/0.050 | 0.048**/0.050 | 0.087**/0.089 | **0.084****/0.087 |
| methyLImp | 0.047***/0.048 | **0.044****/0.046 | **0.083****/0.085 | **0.083****/0.085 |

Comparison between complete/restricted datasets: *** $p < 0.001$, ** $p < 0.01$ *: $p < 0.05$

Best overall performances per metric are highlighted in bold.

Table 10: Disease samples. **MNAR:low** type missing values. Performance comparison between complete/restricted datasets

| | MAE | | RMSE | |
|---|---|---|---|---|
| Method | M-value | B-value | M-value | B-value |
| mean | 0.033/0.033 | 0.034/0.034 | 0.063/0.063 | 0.064/0.064 |
| impute.knn | 0.236/0.072*** | 0.190/0.051*** | 0.284/0.136*** | 0.265/0.111*** |
| softImpute | 0.039/0.038 | 0.035/0.034 | 0.075/0.072 | 0.071/0.071 |
| imputePCA | 0.028/0.028 | 0.029/0.029 | 0.057/0.056 | 0.057/0.057 |
| methyLImp | 0.028/0.028 | **0.026**/0.026 | **0.053**/0.053 | 0.053/0.053 |

Comparison between complete/restricted datasets: *** $p < 0.001$, ** $p < 0.01$ *: $p < 0.05$

Best overall performances per metric are highlighted in bold.

Table 11: Disease samples. **MNAR:mid** type missing values. Performance comparison between complete/restricted datasets

| | MAE | | RMSE | |
|---|---|---|---|---|
| Method | M-value | B-value | M-value | B-value |
| mean | 0.069/0.069* | 0.066/0.066* | 0.100/0.099*** | 0.095/0.094*** |
| impute.knn | 0.092/0.054*** | 0.131/0.073*** | 0.150/0.084*** | 0.195/0.118*** |
| softImpute | 0.069/0.068* | 0.069/0.069 | 0.100/0.098 | 0.112/0.110 |
| imputePCA | 0.059/0.059*** | 0.057/0.056*** | 0.090/0.089* | 0.085/0.084*** |
| methyLImp | 0.056/0.055*** | 0.052/0.052* | 0.084/0.083*** | 0.082/0.081*** |

Comparison between complete/restricted datasets: *** $p < 0.001$, ** $p < 0.01$ *: $p < 0.05$

Best overall performances per metric are highlighted in bold.

Table 12: Disease samples. **MNAR:high** type missing values. Performance comparison between complete/restricted datasets

| | MAE | | RMSE | |
|---|---|---|---|---|
| Method | M-value | B-value | M-value | B-value |
| mean | 0.038/0.038 | 0.040/0.040 | 0.064/0.064 | 0.065/0.064 |
| impute.knn | 0.275/0.098*** | 0.299/0.160*** | 0.334/0.170*** | 0.350/0.237*** |
| softImpute | 0.043/0.041 | 0.059/0.054 | 0.074/0.071 | 0.117/0.105 |
| imputePCA | 0.032/0.032 | 0.033/0.033* | 0.057/0.056 | 0.057/0.056 |
| methyLImp | 0.033/0.033 | **0.030**/0.030 | 0.056/0.056 | 0.054/0.053 |

Comparison between complete/restricted datasets: *** $p < 0.001$, ** $p < 0.01$ *: $p < 0.05$

Best overall performances per metric are highlighted in bold.
